# Efficacy of Xiaoyao-san preparations in treating Hashimoto’s thyroiditis: a meta-analysis and systematic review

**DOI:** 10.3389/fphar.2025.1528506

**Published:** 2025-06-13

**Authors:** Chengna Wang, Wenxin Ma, Lingling Qin, Lili Wu, Tonghua Liu

**Affiliations:** ^1^ The Second Clinical Medical College of Beijing University of Chinese Medicine, Dongfang Hospital of Beijing University of Chinese Medicine, Beijing, China; ^2^ Key Laboratory of Health Cultivation of Traditional Chinese Medicine, the Ministry of Education, Beijing University of Chinese Medicine, Beijing, China; ^3^ Centre for Evidence-Based Chinese Medicine, School of Traditional Chinese Medicine, Beijing University of Chinese Medicine, Beijing, China; ^4^ Department of Technology, Beijing University of Chinese Medicine, Beijing, China

**Keywords:** Hashimoto’s thyroiditis, Xiaoyao-san, randomized controlled trial, systematic review, meta-analysis

## Abstract

**Objective:**

To assess Xiaoyao-san (XYS) preparations’ effectiveness in treating Hashimoto’s thyroiditis.

**Methods:**

Eight databases were searched for randomized controlled trials (RCTs) comparing XYS preparations to a low-iodine diet (LID), selenium yeast (SY), levothyroxine (LT4), or *Ophiocordyceps sinensis* (OS) for HT treatment. The datasets from inception to September 2024. Two reviewers independently evaluated literature and research biases. Meta-analysis was done with Revman 5.4. The GRADE technique assessed evidence reliability. Robustness was assessed using sensitivity and trial sequential analysis (TSA).

**Results:**

This study analyzed seven randomized controlled trials involving 612 patients. Meta-analysis demonstrated that XYS preparations significantly reduced TPOAb levels [SMD = −0.74, 95% CI (−1.02, −0.46), *p* < 0.00001]. Combining LT4 with XYS preparations resulted in greater TPOAb reduction compared to LT4 alone [SMD = −0.77, 95% CI (−1.06, −0.47), *p* < 0.00001] and was more effective in lowering TgAb levels [SMD = −0.66, 95% CI (−1.05, −0.26), *p* = 0.001]. XYSJW outperformed OS in reducing TgAb [SMD = −0.35, 95% CI (−0.58, −0.10), *p* = 0.005]. Four XYS preparations (XY, HHXY, GQXY, and DZXY) increased FT3 and FT4 levels [SMD = 0.13, 95% CI (0.01, 0.61), *p* = 0.04; SMD = 0.58, 95% CI (0.12, 1.04), *p* = 0.01] and decreased TSH [SMD = −0.76, 95% CI (−0.98, −0.54), *p* < 0.00001]. Subgroup analysis indicated XY significantly improved FT3 and FT4 levels, but XYS preparations combined with LT4 did not enhance FT3/FT4 restoration. XYSJW also did not reduce TSH more effectively than OS. Evidence quality was low or very low. TSA confirmed the pooled effect estimates, with cumulative z-curves for TPOAb, TgAb, and TSH surpassing the benefit threshold.

**Conclusion:**

Combination therapy with XYS preparations and LT4 may reduce TPOAb, TgAb, and TSH levels in HT patients. XY combined with LID or SY therapy is more effective at restoring FT3 and FT4 levels. While XYSJW outperforms OS preparations in lowering TgAb levels, it may not surpass OS in restoring thyroid hormone levels. Most studies reviewed are of low quality. XYS preparations appear to modulate cytokines by targeting immune markers and reducing inflammation, but their safety profile remains unclear, requiring further robust evidence.

**Systematic Review Registration:**

clinicaltrials.gov, identifier CRD42023472233.

## 1 Introduction

Hashimoto’s thyroiditis (HT) is a prevalent autoimmune thyroid condition that frequently leads to hypothyroidism. According to a recent meta-analysis, the worldwide occurrence of HT in adults is around 7.5%. The prevalence of HT in females is roughly four times more than in males (Hu et al., 2022). Viral infections have been linked to the development of HT (Lim et al., 2023). Therefore, in the current neocoronavirus pandemic, the occurrence of HT has been seen to be notably higher in those who are infected (Mohammadi et al., 2023). Studies have shown that HT is associated with an elevated risk of developing systemic lupus erythematosus (Lin et al., 2023), polycystic ovary syndrome (Ho et al., 2020), and malignant tumors (Feldt-Rasmussen, 2020, Hu et al., 2020). The available clinical interventions for HT are somewhat restricted. Low iodine intake and selenium supplementation as common trace element adjuvant therapy, as well as thyroid hormone replacement therapy, have been used to treat patients with hypothyroidism. However, for individuals with raised antibodies and normal thyroid function, it is advised to merely do follow-up observations without any specific treatment.

Extracted from the medical compendium “Taiping Huimin Heji Jufang” of the Song Dynasty. Xiaoyao-san (XYS) is a traditional Chinese medicinal formula listed in the 2020 edition of the Chinese National Pharmacopoeia (Chinese Pharmacopoeia Commission, 2020). It is effective in treating various symptoms associated with the syndrome of liver-depression and spleen-deficiency. The composition has eight botanical ingredients, namely, Bupleuri radix [Chaihu, *Bupleurum Chinese* DC.], Paeoniae radix alba [Shaoyao, *Paeonia lactiflora* Pall.], Atractylodis macrocephalae rhizome [Baizhu, *Atractylodes macrocephala* Koidz.], Angelicae sinensis radix [Danggui, *Angelica sinensis (Oliv.)* Diels.], Poria cocos [Fuling, *Poria cocos (Schw.)* Wolf.], Glycyrrhizae radix et rhizome [Gancao, *Glycyrrhiza glabra* L.], Menthae haplocalycis herba [Bohe, *Mentha canadensis* L.], and Zingiberis rhizoma recens [Shengjiang, *Zingiber officinale* Rosc.]. Table 1 displays the botanical names of the botanical drugs together with their primary ingredients.

**TABLE 1:** Composition of Xiaoyao-San.

Non-scientific names (Chinese name)	Class of name	Part(s) of plant used	Name as published	Medicinal source	Maior bioactive metabolites
Bupleuri radix (Chaihu)	Pharmaceutical	Root	*Bupleurum Chinese* DC.	Pharmacop. of China (2015) WHODrug Herbal Substances 2024 (WHO-UMC, 2024)	Saikosaponins A, B, and D (Lin et al., 2005; Li et al., 2018b)
Paeoniae radix alba (Shaoyao)	Pharmaceutical	Root	*Paeonia lactiflora* Pall	Pharmacop. of China (2015) WHODrug Herbal Substances 2024 (WHO-UMC, 2024)	Total glycoside of paeony (Pae, hydroxyl-paeoniflorin, paeonin, albiflorin and benzoylpaeoniflorin, etc.) (Zhang and Wei, 2020)
Atractylodis macrocephalae rhizome (Baizhu)	Pharmaceutical	Rhizome	*Atractylodes macrocephala* Koidz	Pharmacop. of China (2015) WHODrug Herbal Substances 2024 (WHO-UMC, 2024)	Atractylenolide I, Ⅱ, Ⅲ, Ⅳ and V, etc. (Zhu et al., 2018)
Angelicae sinensis radix (Danggui)	Pharmaceutical	Root	*Angelica sinensis (Oliv.)* Diels	Pharmacop. Of China (2015) WHODrug Herbal Substances 2024 (WHO-UMC, 2024)	Z-ligustilide, Senkyunolide A and Ferulic acid, etc. (Wei et al., 2016)
Poria cocos (Fuling)	Other	Sclerotia	*Poria cocos (Schw.)* Wolf *Poria cocos* F.A.Wolf	Pharmacop. of China (2015) WHODrug Herbal Substances 2024 (WHO-UMC, 2024) https://www.gbif.org/	Poricoic acid A, B, and dehydrotumulosic acid, etc. (Yang et al., 2022)
Glycyrrhizae radix et rhizome (Gancao)	Pharmaceutical	Rhizome; Root	*Glycyrrhiza glabra* L	Pharmacop. of China (2015) WHODrug Herbal Substances 2024 (WHO-UMC, 2024)	Glycyrrhiza glabra, glycyrrhizin, etc. (Wang et al., 2020)
Menthae haplocalycis herba (Bohe)	Pharmaceutical	Aerial parts	*Mentha canadensis* L	Pharmacop. of China (2015) WHODrug Herbal Substances 2024 (WHO-UMC, 2024)	Hesperidin, rosmarinic acid, diosmin, etc. (Zhao et al., 2018)
Zingiberis rhizoma recens (Shengjiang)	Pharmaceutical	Rhizome	*Zingiber officinale* Rosc	Pharmacop. of China (2015) WHODrug Herbal Substances 2024 (WHO-UMC, 2024)	Diarylheptanoids, linear diarylheptanoids and ring diarylheptanoids, etc. (Lu et al., 2022)

Traditional Chinese medicine asserts that the thyroid gland is linked to the liver meridian of the foot-jueyin. This meridian is profoundly influenced by emotions, and specific thyroid-related symptoms may manifest when liver qi is impeded and blood stagnation develops in the neck. The five components hypothesis of Traditional Chinese Medicine asserts a substantial connection between the liver and spleen. Liver depression leads to reduced splenic function, resulting in symptoms like dyspepsia, constipation, and other manifestations similar to thyroid hormone deficiency. Traditional Chinese medicine asserts that the spleen governs blood regulation. Given the increased incidence of thyroid diseases in women, patients exhibit a greater vulnerability to menstrual irregularities. XYS is considered a reliable and effective TCM substance, recognized for its capacity to detoxify the liver, strengthen the spleen, and enrich the blood. Following the principles of Traditional Chinese Medicine (TCM), the therapeutic effects are primarily attributed to the five botanical drugs components: Bupleuri radix [Chaihu, *Bupleurum Chinese* DC.], Paeoniae radix alba [Shaoyao, *P. lactiflora* Pall.], Atractylodis macrocephalae rhizome [Baizhu, *Atractylodes macrocephala* Koidz.], Angelicae sinensis radix [Danggui, *A. sinensis* (Oliv.) Diels.], and Poria cocos [Fuling, *P. cocos* (Schw.) Wolf.]. These five botanical drugs are also recognized as the main components for the identification of XYS in the Chinese Pharmacopoeia, as detailed in the Supplementary Table S4, S6. It is frequently employed to treat several conditions related to mood, digestion, and menstruation in women, including mixed anxiety-depression (Li et al., 2022), sleep and mood problems (Yang et al., 2022), and functional dyspepsia in perimenopausal depression (Du et al., 2014). The efficacy of XYS preparations has been demonstrated in multiple thyroid disorders, including thyroid nodules (Bai et al., 2019), hyperthyroidism (Shi et al., 2020), thyroid-associated ophthalmopathy (Wang et al., 2014; Huang, 2021), and Hashimoto’s thyroiditis (Du JH. et al., 2020; Zhang et al., 2022).

In clinical practice, the prescription is adjusted based on these five botanical drugs according to the various TCM symptoms presented by the patients. Danzhi Xiaoyao Powder (DZXY) is the most often recommended therapy for hyperthyroidism patients in Taiwan (Chang et al., 2022). Honghua Xiaoyao pill (HHXY) and DZXY are often utilized Chinese polyherbal preparation (CCPP). Both have demonstrated success in treating irregular menstruation (Pei et al., 2016), melasma (Liu et al., 2017), mammary hyperplasia (Meng et al., 2014), and H-type hypertension (Li et al., 2020). It may enhance the efficacy of antithyroid medications (ATDs) in managing hyperthyroidism (Ma et al., 2023). Multiple clinical investigations indicate that XYS drugs, including XY, HHXY, and DZXY, can diminish TPOAb and TgAb levels (Atkins et al., 2004; Zhang and Zhu, 2018), hence improving thyroid function in patients with hypothyroidism produced by Hashimoto’s thyroiditis (Gai et al., 2023; Du J. et al., 2020; Chen et al., 2021).

*Ophiocordyceps sinensis* (OS, also called *Cordyceps sinensis*), acknowledged for its varied biological impacts on the circulatory, pulmonary, renal, and urinary systems (Booi et al., 2022), has been employed in China for millennia and signifies a prospective herbal treatment for investigation and application. OS exhibits unique immunological properties (Rong et al., 2021) and has been widely employed in therapeutic investigations for many autoimmune diseases, such as experimental autoimmune thyroiditis (EAT) (Xiao et al., 2018). OS may significantly reduce blood concentrations of TSH, TPOAb, and TgAb, while improving the histological irregularities of the thyroid. Clinically, OS can significantly reduce TPOAb levels in HT patients while altering the balance of helper T cells and cytotoxic T cells (He et al., 2016). The recent meta-analysis demonstrated that Cordyceps sinensis significantly decreased TPOAb and TgAb levels in the treatment of Hashimoto’s thyroiditis (Wei et al., 2023). This study utilizes current research, employing OS preparations in the comparative analysis to provide more robust and comprehensive data for therapeutic applications. The bulk of these studies had a limited participant pool and were conducted at a single institution. This study rigorously evaluated the efficacy of XYS preparations, formulated with the five main botanical drugs, in the treatment of HT, as compared to a LID, SY, LT4 and OS.

## 2 Materials and methods

The meta-analysis was reported in full adherence to the PRISMA guidelines, which are the recommended reporting standards for systematic reviews and meta-analyses (Supplementary Table S1). The study protocol was registered with the International Prospective Systematic Review Registry (PROSPERO 2023 CRD42023472233) (Moher et al., 2009). All the botanical drugs and CCPP involved are listed in Supplementary Table S4-S10. All general rules and patent contents can be viewed in Supplementary Material S2. The PDF file generated by the evaluation tool of ConPhYMP guidelines can be viewed in Supplementary Material S1. Note: For the parts of the article that do not involve chemical components, the Chinese names of the herbal medicines will be used for a more concise and accurate expression.

### 2.1 Inclusion criteria

#### 2.1.1 Type of studies

Randomized controlled trials (RCTs).

#### 2.1.2 Participants

The study included adult participants aged 18 years or older who had received a diagnosis of HT based on the criteria of a recognized professional organization, with or without hypothyroidism. The study did not consider factors such as sex, ethnicity, race, or duration of HT.

#### 2.1.3 Interventions and comparisons

The intervention group was administered a low-iodine diet (LID), selenium yeast supplements (SY), or levothyroxine (LT4), alongside oral drugs (e.g., DZXY, HHXY, etc.). The control group was administered LID, SY, LT4, or OS therapy. The types and dosage forms of LID, SY, or LT4 were the same in both groups. The XYS preparation is CCPP with the five main botanical drugs of XYS as the main ingredient, and the specific form, quantity, or duration are not limited. The intervention method employed oral administration.

#### 2.1.4 Outcomes

The principal outcomes of this study were TPOAb and TgAb, while the secondary outcomes included free triiodothyronine (FT3), free thyroxine (FT4), thyroid stimulating hormone (TSH), and traditional Chinese medicine symptom score (TCMS). The additional outcomes included tumor necrosis factor-α (TNF-α), interleukin-2 (IL-2), interleukin-6 (IL-6), and adverse reactions.

### 2.2 Exclusion criteria

1) Full text is not available. 2) Reviews, case reports, reviews, animal experiments and other non-RCT studies. 3) The language is not Chinese or English. 4) No required outcome data. 5) Missing or erroneous data.

### 2.3 Search strategy

Eight databases, including PubMed, Web of Science, Cochrane Library, Embase, SinoMed, CNKI (China National Knowledge Infrastructure), Chongqing Chinese Science and Technology Journal Database (VIP), and Wanfang Data, were searched for publications published prior to September 2024. After consulting the MeSH related terms provided by PubMed, the search terms were identified as follows: Disease, Hashimoto, Hashimoto Struma, Hashimoto Thyroiditis, Hashimoto Thyroiditides, Thyroiditides, Hashimoto, Thyroiditis, hashimoto, thyroiditis, Hashimoto, Hashimoto’s Syndrome, Hashimoto’s Syndrome, Hashimoto’s Syndromes, Hashimotos Syndrome, Syndrome, Hashimoto’s syndrome, Hashimoto’s Struma, Hashimoto’s struma, Chronic Lymphocytic Thyroiditis, also known as Hashimoto’s Disease, is a condition characterized by inflammation of the thyroid gland. It is a chronic autoimmune disorder that affects the lymphocytes in the thyroid. The condition is often referred to as Lymphocytic Thyroiditis or simply Thyroiditis. The several names for this substance include xiaoyao, danzhi xiaoyao, kamisyoyo san, XYDN (xiaoyao), xiao-yao, kami-shoyo-san, kami-soyo-san, TJ-24, TJ24, jiawei-xiaoyao-san, jia-wei-xiao-yao-san, and kamisyoyo-san. Systematic retrieval was conducted by selecting various combinations and matches, such as subject words, free words, and abstracts, based on the retrieval characteristics of distinct databases. The Supplementary Table S2 provides a comprehensive overview of the specific retrieval procedures.

### 2.4 Study selection and data extraction

The EndNote 20 software was utilized for the purpose of organizing and categorizing literature. An initial screening was performed by reading the title and abstract. If the necessary information for inclusion and exclusion cannot be obtained, we proceed to download the complete text. Upon completion of reading the entire text, the literature underwent a rigorous rescreening process that adhered strictly to the predetermined criteria for inclusion and exclusion. We utilized Excel tables for the extraction of literature. The pertinent information from the studies included was extracted, including 1) the name and publication year of the primary author; 2) the sample size and age range of both the intervention and control groups; 3) the method used to generate the random sequence; 4) the various intervention methods and duration for both XYS preparations and comparator drugs; 5) the outcome indicators. The systematic review included a summary of the following items from the included studies: 1) The initial author’s name and the year of publication; 2) Modifications in the composition, forms, and dosage of XYS preparations; 3) Indicators used for observation; 4) Factors contributing to efficacy and the underlying mechanisms. The process of selecting and extracting data was carried out by two raters in an independent manner. In case of any disagreements, a third rater resolved them through collaborative conversation.

### 2.5 Risk of bias assessment

The investigators utilized the Cochrane Risk of Bias tool in RevMan 5.4 software (Higgins et al., 2011) to assess the quality of the included studies in the following dimensions: a) Random sequence generation, b) Concealment of allocation, c) Blinding of participants and personnel, d) Blinding of outcome assessment, e) Incomplete outcome data, f) Selective reporting, and g) Other biases. The risk outcomes were categorized into three levels: low, unclear, and high. Two reviewers conducted separate evaluations to determine the potential for bias in the studies that were included. Any conflicts were resolved through collaborative deliberation involving a third reviewer.

### 2.6 Statistical analysis and publication bias

Data analysis was conducted using Review Manager software (version 5.4, Copenhagen: Nordic Cochrane Centre, The Cochrane Collaboration, 2014). All outcomes examined in this study were characterized as continuous variables. For the treatment of various units or measurements of continuous variables, study results articulated in interquartile ranges were transformed into means and standard deviations using the method outlined in Section 6.5.2.5 of the Cochrane Handbook. When dealing with various units or measurement methods for continuous variables, the standardized mean difference (SMD) was employed as the combined effect indicator instead of the mean difference (MD). For each effect magnitude, we computed point estimates and 95% confidence intervals (CI).

The Cochrane Q test was employed to examine the heterogeneity among the findings of the studies that were included, while I^2^ was utilized to measure the extent of heterogeneity (Higgins and Thompson, 2002). When the value of *p* ≥ 0.1 and the value of I^2^ < 50%, there was no statistically significant variation in each outcome. Therefore, the fixed effect model was employed for Meta-analysis. When the *p* < 0.1 and the I^2^ > 50%, the results exhibit statistical heterogeneity, and the cause of this heterogeneity was further investigated. Only methods such as subgroup analysis or descriptive analysis were employed to identify the causes of considerable clinical heterogeneity. Following the clarification of the impact of substantial clinical heterogeneity, a random-effects model was employed for the meta-analysis. A meta-analysis was conducted using a significance threshold α = 0.05. The robustness of the pooled results was assessed using TSA and sensitivity analysis (leave-one-out approach). The objective of this study was to investigate XYS preparations with different treatment regimens (including combinations with LT4, SY, etc.) and formulation compositions (such as variations in the original formula, additives, and subtractions). The Egger’s test was used to evaluate the presence of publication bias in the meta-analysis when there were 10 or more papers included. If the value of *p* < 0.05, it suggests that the study exhibited publication bias. Sensitivity analyses and Egger’s test were conducted using Stata software (version 18, Stata Corporation, College Station, Texas, United States). Finally, for results that cannot be enhanced by the above methods, the Rosenthal fail-safe N is employed to measure the number of non-significant studies that need to be published to invalidate the meta-analysis findings (Becker, 2005), thereby increasing the certainty of the results. The calculation of Rosenthal fail-safe N is conducted using R software (version 4.3.0; R Core Team, 2023).

### 2.7 Trial sequential analysis

To determine whether the meta-analysis contained the necessary data and to address potential problems that could have led to false positive (type I error) or false negative (type II error) results due to insufficient power to detect an intervention effect, TSA software (0.9.5.10 Beta, Copenhagen Trial Unit, Denmark) was used (Brok et al., 2008). The continuous data type was chosen, and the cumulative z-curve was built using a random effects model. The hypothesis test was conducted using a two-sided test. The type I error was determined to be 5%, and the penalty value was set at 2. The aggregated sample size was chosen as the information axis, and the statistical power was set at 80%. The necessary information size to detect or reject the impact of the intervention was determined by utilizing estimations for the quantity of information, which was derived from empirical data for both the mean difference and variance, and model-based variance for correcting heterogeneity.

### 2.8 Certainty assessment

The quality of evidence for outcomes was assessed using the Grading of Recommendations, Assessment, Development, and Evaluation (GRADE) approach (Atkins et al., 2004). The evidence was assessed based on criteria such as bias, inconsistency, indirectness, imprecision, and publication bias. The evidentiary quality was categorized into four tiers: high, medium, low, and very low.

## 3 Results

### 3.1 Study selection

**FIGURE 1:**
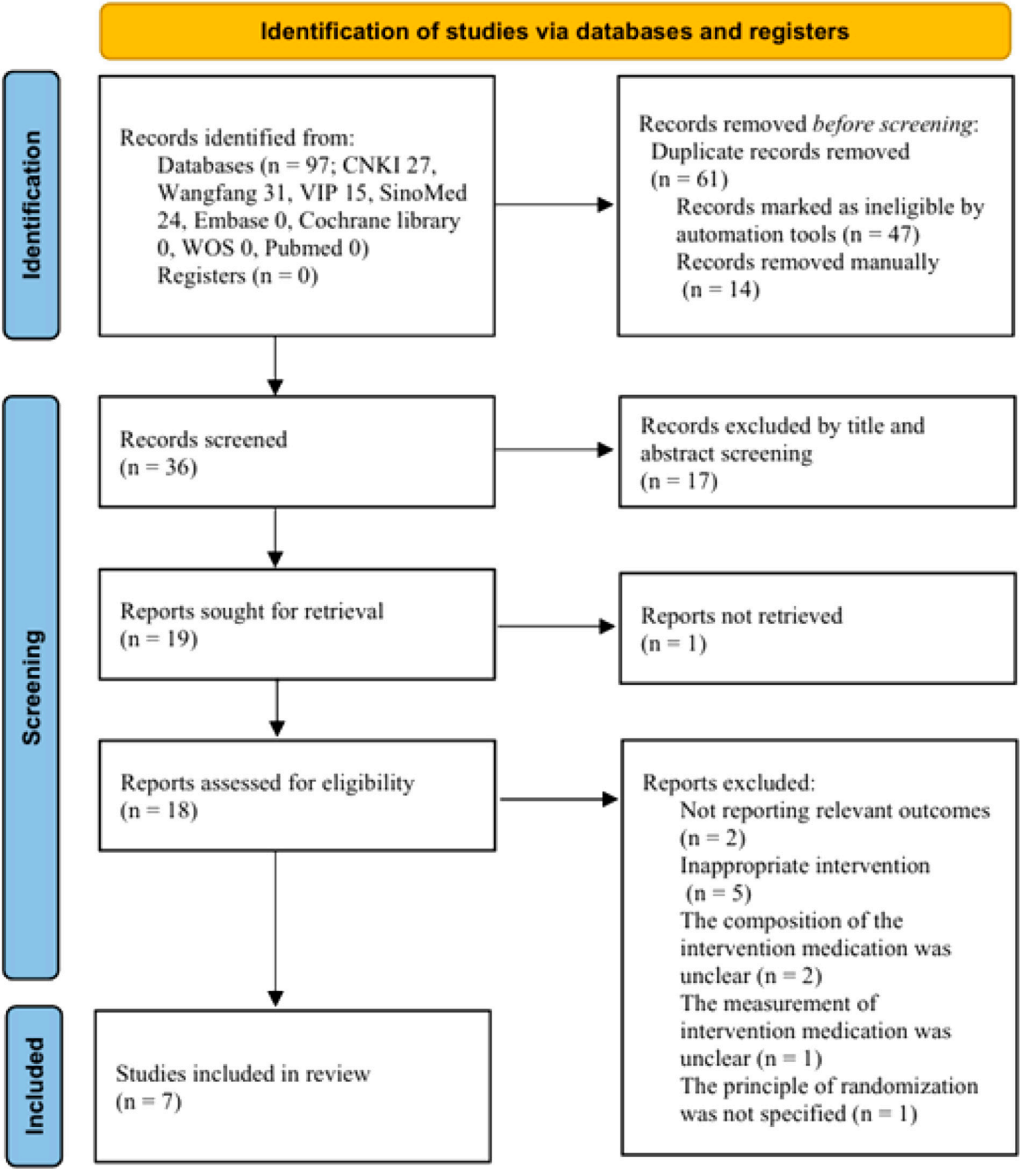
PRISMA Flow diagram of the record selection process.

A total of 97 articles were included, all written in Chinese. Employing EndNote 20 software to systematically eliminate duplicates from 47 documents. Fourteen elements were manually eliminated, resulting in thirty-six remaining for further study. Seventeen papers were rejected for various reasons following the evaluation of titles and abstracts: Two studies were network pharmacology analyses, one was animal research, one was a review, one was a retrospective study, and twelve were non-randomized controlled trials. A study was retracted due to its inaccessibility for download. Eighteen articles were retrieved, of which eight satisfied the inclusion criteria. Ten publications were discarded for several reasons: insufficient explanation of significant outcomes (*n* = 2), inappropriate intervention (*n* = 5), ambiguous formulation of intervention drugs (*n* = 2), and vague evaluation of intervention pharmaceuticals (*n* = 1). A total of eight articles were incorporated for systematic review and meta-analysis. Figure 1 illustrates the literature screening process, while Supplementary Table S3 offers detailed annotations of the publications excluded following the removal of duplicate data.

### 3.2 Study characteristics in the meta-analysis

This meta-analysis encompassed seven studies with a total of 612 participants with HT. 420 cases of normal thyroid function and 192 cases of hypothyroidism. The trials were conducted in mainland China from 2018 to 2023 and subsequently published in Chinese. Intervention group: 307 patients; control group: 305 cases. The experiment requires a minimum sample size of 60 patients and a maximum of 162 persons. Enumerate five types of CCPP: Xiaoyao Pills (XY), Danzhi-Xiaoyao Granules (DZXY), Honghua-Xiaoyao Tablets (HHXY), Guiqi-Xiaoyao Mixture (GQXY), and Xiaoyao-San-Jiawei (XYSJW). Consult Supplementary Table S4-S10 for further details on these preparations. In four trials, the XYS preparations were solely used with the LID intervention, whereas all other studies utilized LT4 or SY combinations as the intervention drugs. Six studies had a duration of 3 months, one research had a duration of 6 months, and another study had a duration of 1 month. Consult Table 2 for further information.

**TABLE 2:** Basic characteristics of the included studies.

Study ID	Sample size (E/C)	Age (year)		Male/Female		Intervention(s)	Comparators	Treatment duration (week)	With hypothyroidism	Outcomes
		E	C	E	C					
Chen et al. (2021)	34/34	37.8 ± 3.95	38.4 ± 4.57	6/28	5/29	XY 1.6 g tid + SY	SY	24	no	1,2,3,4,5
Cheng, J. (2021)	30/30	36.03 ± 9.94	36.87 ± 11.36	5/25	4/26	GQXY 0.2 g bid + LT4	LT4	12	yes	1,2,3,4,5,6
Du et al. (2020)	30/30	39.29 ± 11.68	37.54 ± 12.11	2/28	3/27	(DZXY 9 g + XKC 9 g) bid + LT4	LT4	12	yes	1,2,3,4,5,10
Gai et al. (2023)	36/36	40.14 ± 10.01	41.92 ± 7.66	11/25	14/22	HHXY 0.78 g tid + LT4	LT4	12	yes	1,2,3,4,5,6,9 10
Zhang et al. (2019)	40/40	36.2 ± 6.4	35.6 ± 6.8	0/40	0/40	XY 2.4 g tid + LID	LID	12	no	1,2,3,4,5,10
Li (2021b)	55/55	43.56 ± 11.41	42.82 ± 12.34	7/48	5/50	XYSJW 30 g tid + LID	OS 1.68 g tid	12	no	1,2,3,4,5,6
Zhang et al. (2022)	82/80	38.18 ± 10.41	40.28 ± 10.519	24/58	21/59	XYSJW 30 g tid + LID	OS 1.68 g tid	12	no	1,2,3,4,5,6,9

E, experimental group; C, control group; XY, xiaoyao pill; GQXY, guiqi xiaoyao mixture; DZXY, danzhi xiaoyao granules; XKC, xiakucao gao; HHXY, honghua xiaoyao tablet; XYSJW, xiaoyao san jiawei granules; LID, low-iodine diet; SY, selenium yeast tablets; OS, Ophiocordyceps sinensis (Jinshuibao tablets); LT4, levothyroxine; 1: thyroid peroxidase antibody (TPOAb); 2: thyroglobulin antibody (TgAb); 3: free triiodothyronine (FT3); 4: free thyroxine (FT4); 5: thyroid stimulating hormone (TSH); 6: TCM, symptom score (TCMS); 7: tumor necrosis factor alpha (TNF-α); 8: interleukin-2 (IL-2); 9: interleukin-6 (IL-6); 10: adverse events.

### 3.3 Quality assessment of included studies

**FIGURE 2:**
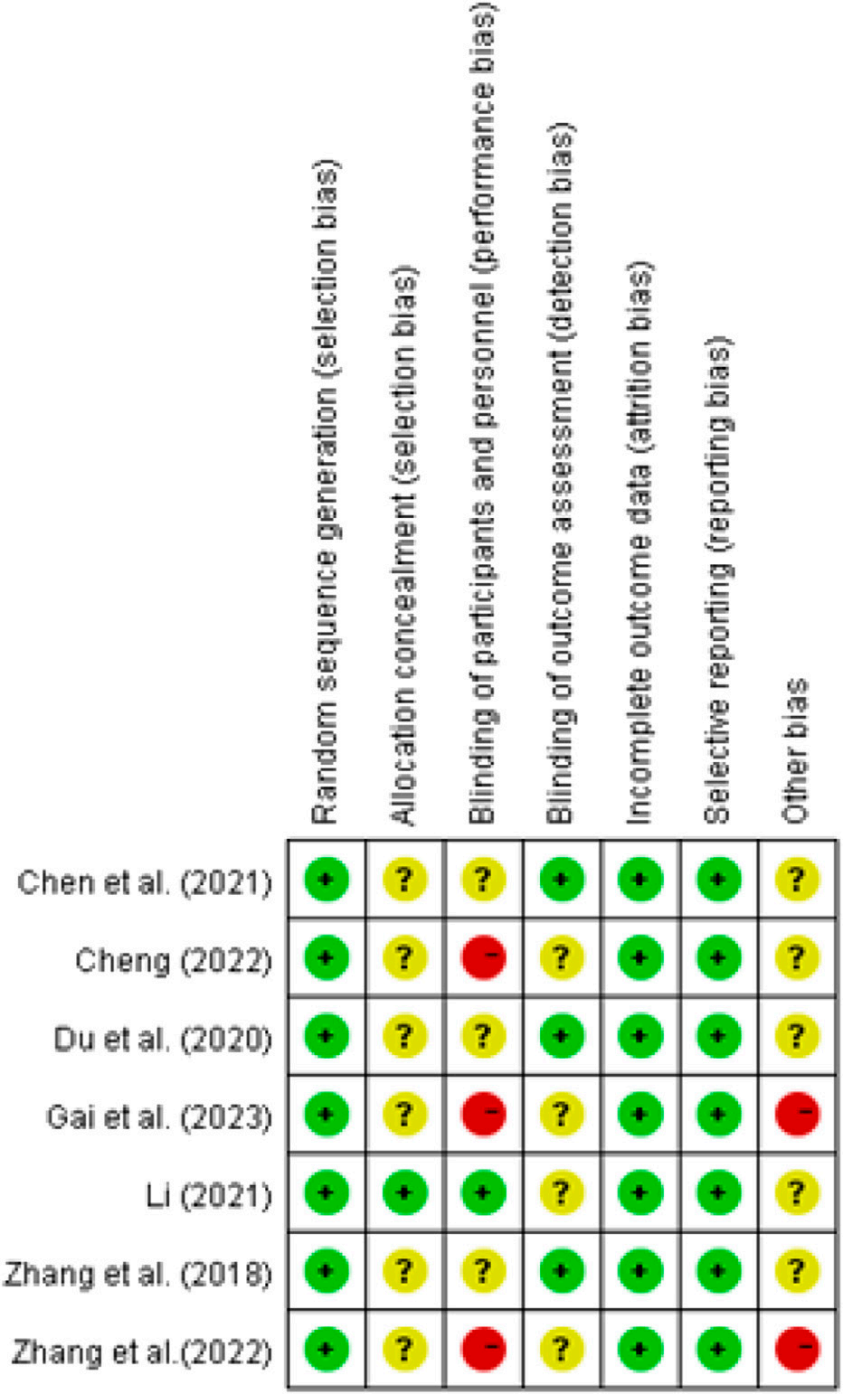
Risk of bias summary.

Of the seven studies analyzed, all of them employed the random number table method for group allocation (Gai et al., 2023; Du JH. et al., 2020; Zhang and Zhu, 2018; Chen et al., 2021; Cheng, 2021; Zhang et al., 2022; Li Yinan., 2021), Hence, the generation of random sequences is considered low risk. Only one study was deemed to have a low operational risk (Li YN., 2021), while the rest of the studies that did not mention blinding were considered to have a high risk of concealment bias (Cheng, 2021; Du J. et al., 2020; Zhang et al., 2022). Three studies that only described physiological indicators were considered to have an unclear operational risk, while the detection bias was considered low (Cheng, 2021; Gai et al., 2023; Zhang and Zhu, 2018). The detection bias was unclear in the remaining studies. All study data has been finalized, and the attrition rate is below 10%. The absence of pre-registration for the study protocols introduces an ambiguous risk of first-reporting bias for all examined investigations. Consequently, it is unfeasible to ascertain if all anticipated effects have been documented. In two investigations, the gender distribution of male and female patients diverged from the actual clinical demographics (Gai et al., 2023; Zhang et al., 2022), indicating the potential presence of additional biases. Figure 2 depicts the evaluation of probable bias in the reviewed literature.

### 3.4 Effectiveness of interventions

#### 3.4.1 Primary outcomes and subgroup analysis

**FIGURE 3:**
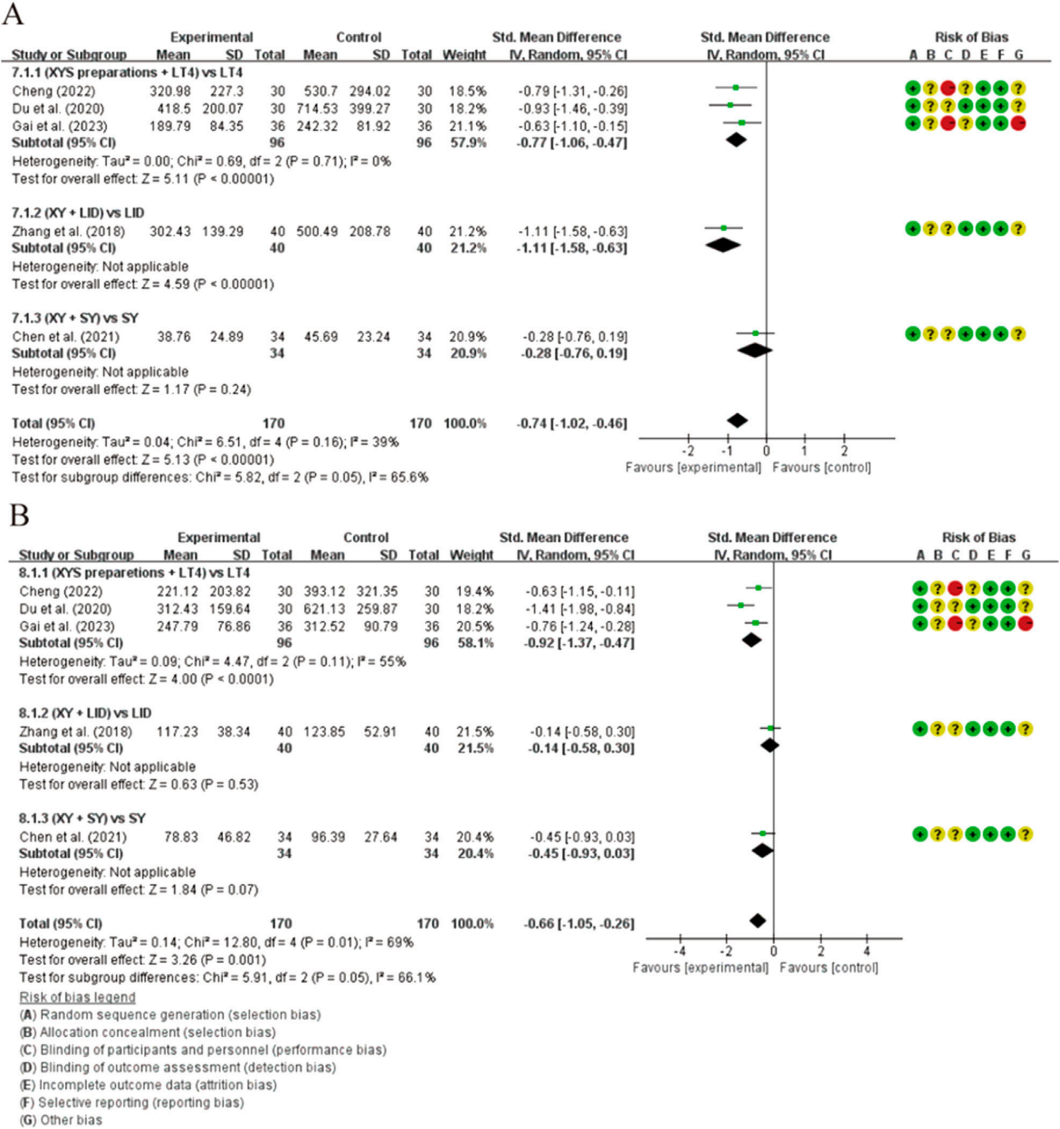
Forest plots of the effectiveness comparison of autoantibodies (XYS preparations vs LT4, LID, and SY): **(A)** TPOAb; **(B)** TgAb.

**FIGURE 4:**
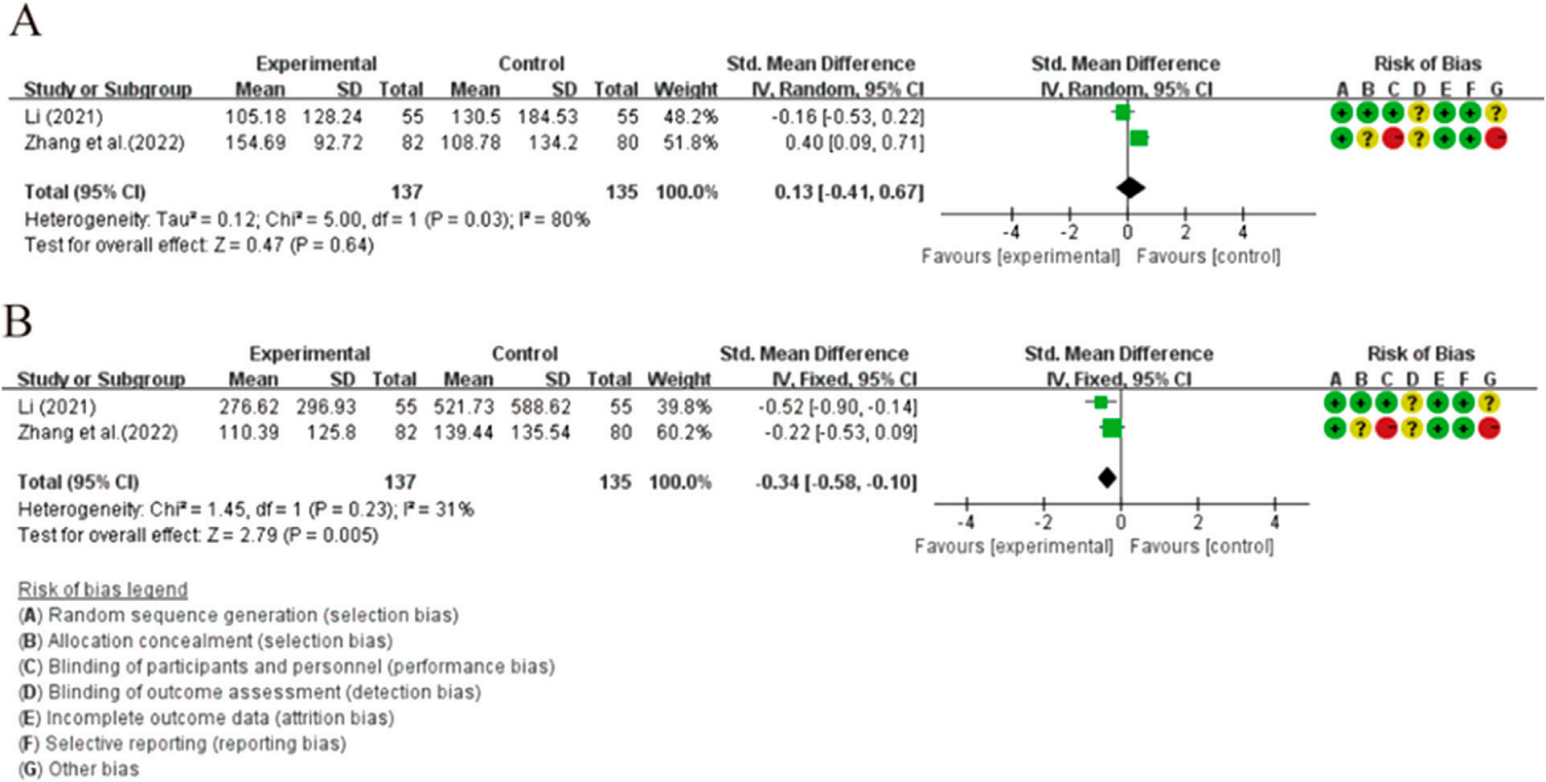
Forest plots of the effectiveness comparison of autoantibodies (XYSJW vs OS): **(A)** TPOAb; **(B)** TgAb.

As shown in Figure 3, thyroid autoantibodies were detected in all evaluations. Since OS is an extract of CCPP, to ensure more rigorous control of intergroup differences, we conducted separate comparisons between XYSJW and OS trials. Compared to the control group, XYS preparations exhibited a greater impact on TPOAb levels [SMD = −0.74, 95% CI (−1.02, −0.46), *p* < 0.00001]. The overall difference across the entire investigation was not significant (*p* < 0.16, I^2^ = 39%). A subgroup analysis was conducted to investigate potential reasons for the discrepancies. The subgroup analysis of combination therapy with XYS preparations indicated that the combination of XYS and LT4 was significantly more effective in reducing TPOAb levels compared to LT4 monotherapy [SMD = −0.77, 95% CI (−1.06, −0.47), *p* < 0.00001], without heterogeneity. Due to the availability of only one study on the combination of XY with LID and SY, their results were described: XY combined with LID could reduce TPOAb levels, while the combination of XY and SY showed no statistical significance in reducing TPOAb levels. Significant statistical differences were observed among subgroups with different treatment regimens (*p* = 0.05, I^2^ = 65.5%), suggesting that variations in treatment regimens might be the primary source of heterogeneity (Supplementary Table S11). Additionally, there was no statistically significant difference in the reduction of TPOAb levels between XYSJW and OS preparations [SMD = 0.13, 95% CI (−0.41, −0.67), *p* = 0.64]. (Figure 4).

The XYS preparations intervention group was superior to the control group in reducing TgAb levels, showing a significant statistical difference [SMD = −0.66, 95% CI (−1.05, −0.26), *p* = 0.001], while exhibiting considerable heterogeneity (*p* = 0.01, I^2^ = 69%). Subgroup analysis indicated that the combination of XYS preparations and LT4 was more effective than LT4 monotherapy [SMD = −0.92, 95% CI (−1.37, −0.47), *p* < 0.0001], without demonstrating significant heterogeneity (*p* = 0.11, I^2^ = 55%). Whether XY was used in combination with LID or SY, it did not show a better effect in reducing TgAb compared to LID or SY monotherapy. Significant statistical differences were observed among subgroups with different treatment regimens, which may be the primary source of heterogeneity (*p* = 0.05, I^2^ = 66.1%) (Figure 3; Supplementary Table S11). Additionally, there was a significant statistical difference between XYSJW and OS preparations in reducing TgAb levels [SMD = −0.35, 95% CI (−0.58, −0.10), *p* = 0.005], with no significant heterogeneity observed between groups (*p* = 0.23, I^2^ = 31%). (Figure 4).

#### 3.4.2 Secondary outcomes and subgroup analysis

**FIGURE 5:**
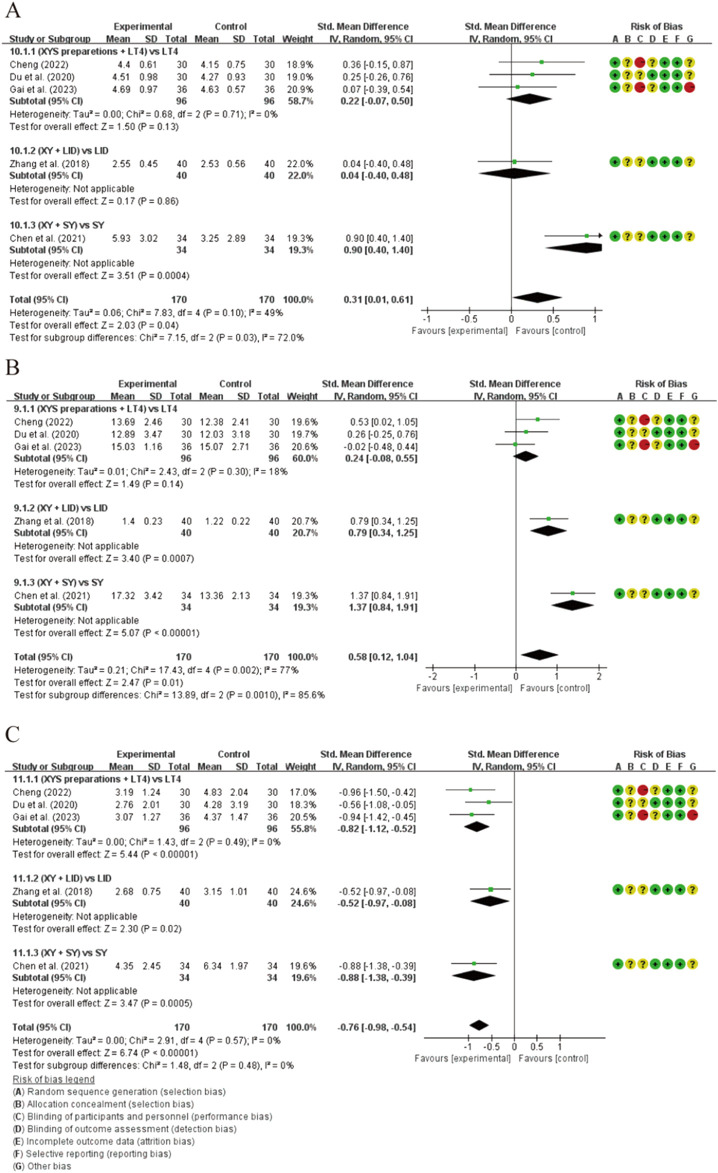
Forest plots of the effectiveness comparisons for thyroid function (XYS preparations vs LT4, LID, and SY): **(A)** FT3, **(B)** FT4, and **(C)** TSH.

**FIGURE 6:**
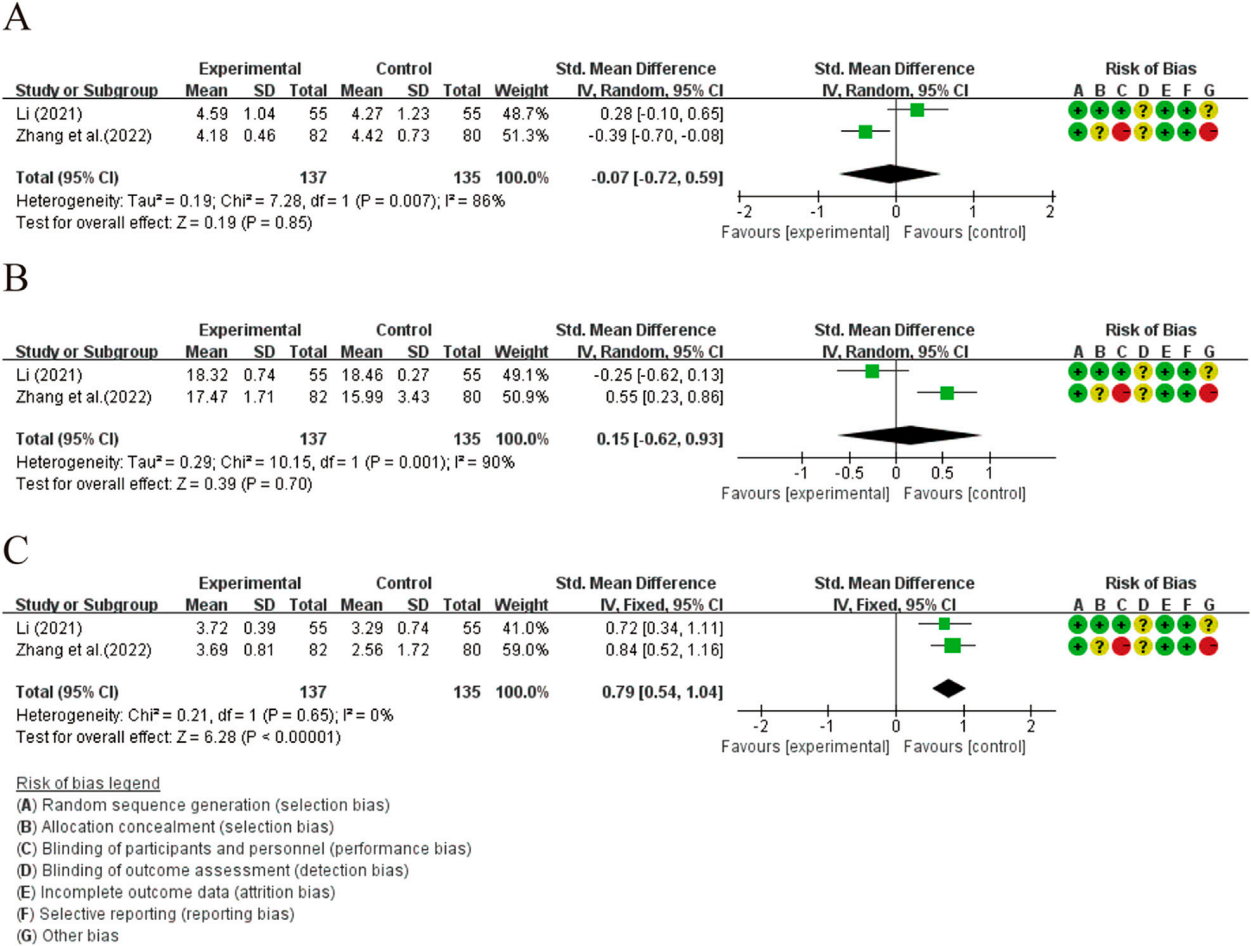
Forest plots of the effectiveness comparisons for thyroid function (XYSJW vs OS): **(A)** FT3, **(B)** FT4, and **(C)** TSH.

All seven studies evaluated thyroid function (Figure 5). The studies demonstrated a statistically significant difference between the two groups in terms of elevated FT3 levels [SMD = 0.13, 95% CI (0.01, 0.61), *p* = 0.04]. The heterogeneity was not significantly altered (*p* = 0.10, I^2^ = 49%). Subgroup analysis revealed significant differences among groups receiving different treatment methods (*p* = 0.03, I^2^ = 72%). Combination therapy with XYS preparations and LT4 did not show a significant difference compared to LT4 monotherapy in elevating FT3 levels [SMD = 0.22, 95% CI (−0.07, 0.50), *p* = 0.13], and no heterogeneity was detected (*p* = 0.71, I^2^ = 0%). Combination therapy of XY and LID did not demonstrate a better ability to elevate serum FT3 levels compared to LID intervention alone. However, combination therapy of XY and SY showed significantly better efficacy in elevating FT3 levels compared to SY monotherapy, suggesting that this treatment regimen may be the primary source of heterogeneity among subgroups (*p* = 0.03, I^2^ = 72%). (Supplementary Table S11). Additionally, combination therapy with XYSJW and OS preparations did not exhibit a better ability to restore FT3 levels compared to OS monotherapy. (Figure 6).

The results showed a statistically significant difference in FT4 levels between the two groups [SMD = 0.58, 95% CI (0.12, 1.04), *p* = 0.01], accompanied by considerable heterogeneity (*p* = 0.002, I^2^ = 77%). In the subgroup analysis, the combination of XYS preparations with LT4 did not demonstrate a superior ability to restore FT4 levels compared to LT4 alone [SMD = 0.24, 95% CI (−0.08, 0.55), *p* = 0.14], and the heterogeneity was not significant (*p* = 0.30, I^2^ = 18%). However, when XY was combined with LID or SY, it showed a better ability to restore FT4 levels than LID or SY monotherapy, which may be the main reason for the heterogeneity. Significant heterogeneity among subgroup analyses supported this view (*p* = 0.001, I^2^ = 85.6%). (Supplementary Table S4, S11). Additionally, there was no statistically significant difference between XYSJW and OS preparations in restoring serum FT4 levels. (Figure 6).

The results of the study demonstrate that XYS preparations has statistically significant effects on reducing TSH levels [SMD = −0.76, 95% CI (−0.98, −0.54), p < 0.00001], and the findings are robust in terms of heterogeneity (*p* = 0.57, I^2^ = 0%). Subgroup analysis reveals that combinations of XYS preparations with LT4 therapy, XY with LID treatment, and XY with SY treatment all exhibit significant abilities to restore TSH levels, and the results are relatively consistent across groups (*p* = 0.48, I^2^ = 0%). (Supplementary Table S11). Compared to OS, XYSJW shows a statistically significant increase in TSH levels [SMD = 0.79, 95% CI (0.54, 1.04), *p* < 0.00001], with minimal heterogeneity (*p* = 0.65, I^2^ = 0%) (Figure 6).

**FIGURE 7:**
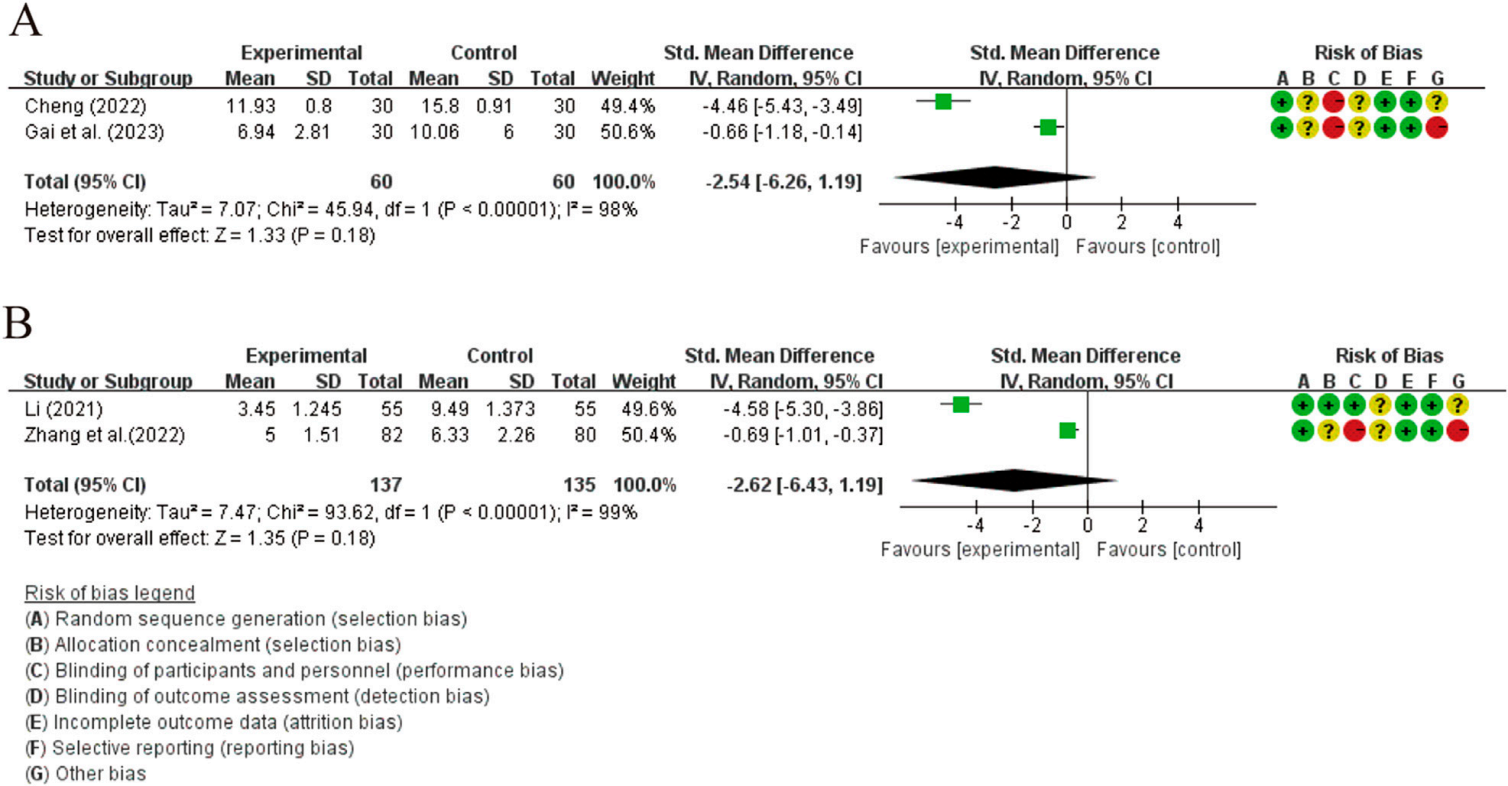
Forest plots of the effectiveness comparison of TCMs: **(A)** XYS preparations vs LT4 and SY, **(B)** XYSJW vs OS.

Four studies evaluated the effect of XYS preparations on TCMs (Gai et al., 2023; Cheng, 2021; Li Yinan., 2021; Zhang et al., 2022). The results indicated no evidence that the involvement of XYS preparations in treatment significantly reduced TCMs [SMD = −2.54, 95% CI (−6.26, 1.19), *p* = 0.18]. The same conclusion was reached in the comparison between XYSJW and OS. [SMD = −2.62, 95% CI (−6.43, 1.19), *p* = 0.18]. However, both comparisons showed considerable heterogeneity (*p* < 0.00001, I^2^ = 98%; *p* < 0.00001, I^2^ = 99%). The source of this heterogeneity could not be determined through subgroup analysis. Upon reviewing the studies, we believe this heterogeneity was primarily due to the gender ratio in two studies not aligning with the clinical characteristics of HT (Gai et al., 2023; Zhang et al., 2022) (Figure 7).

#### 3.4.3 Additional outcomes

**FIGURE 8:**
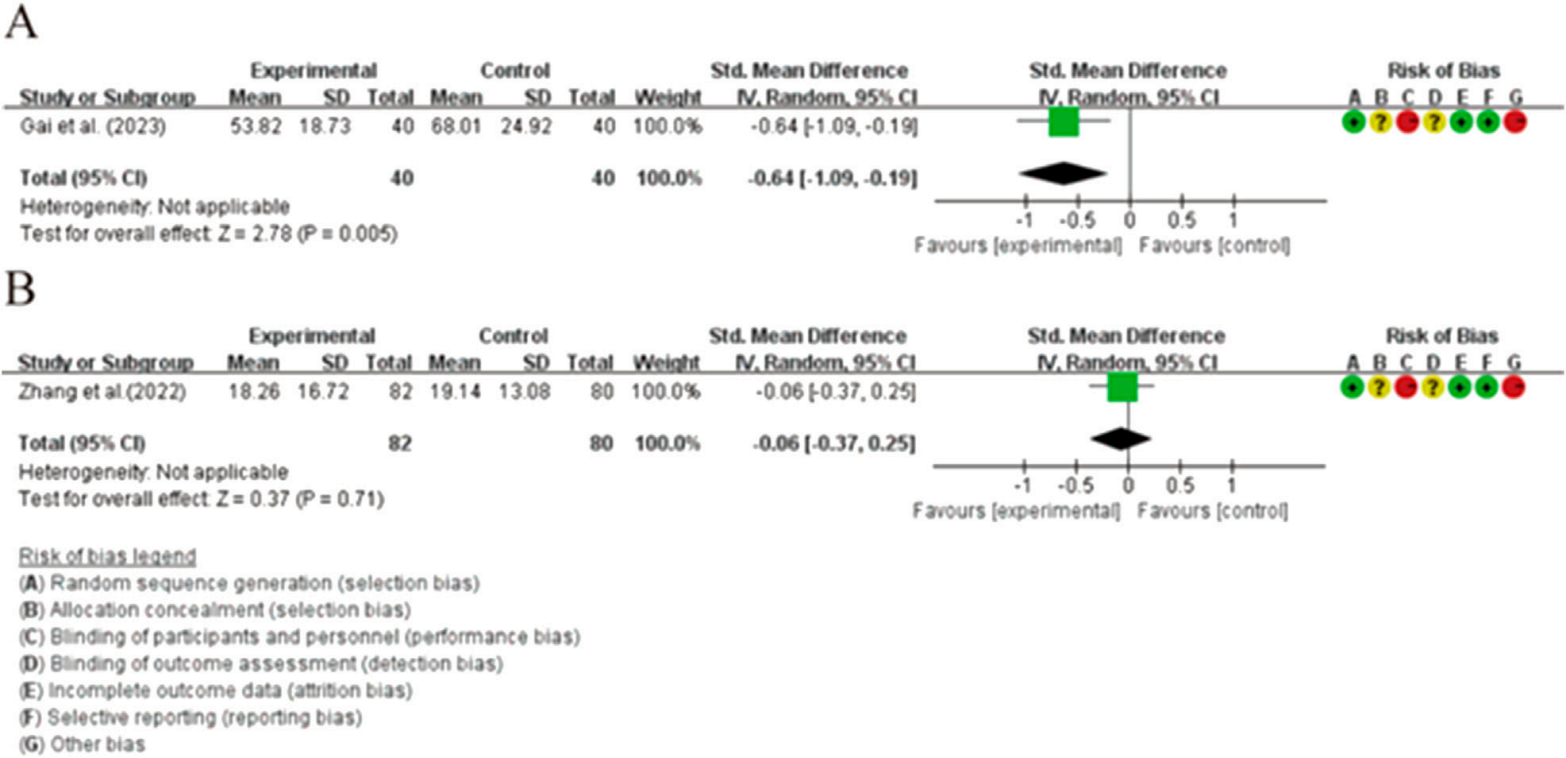
Forest plots of the effectiveness comparison of IL-6: **(A)** HHXY + LT4 vs LT4, **(B)** XYSJW vs OS.

A descriptive evaluation of serum IL-6 levels in two experiments revealed a more significant decrease in blood IL-6 levels in the HHXY combined with LT4 intervention group compared to the LT4 group [SMD = −0.64, 95% CI (−1.09, −0.19), *p* = 0.005]. When comparing XYSJW with OS preparations, no statistically significant difference was observed [SMD = −0.06, 95% CI (−0.37, 0.25), *p* = 0.71] (Figure 8). However, no studies measured serum IL-2 and TNF-α concentrations. Three studies documented negative reactions (Gai et al., 2023; Du JH. et al., 2020; Zhang and Zhu, 2018), although no adverse effects were identified.

#### 3.4.4 Sensitivity analysis

**FIGURE 9:**
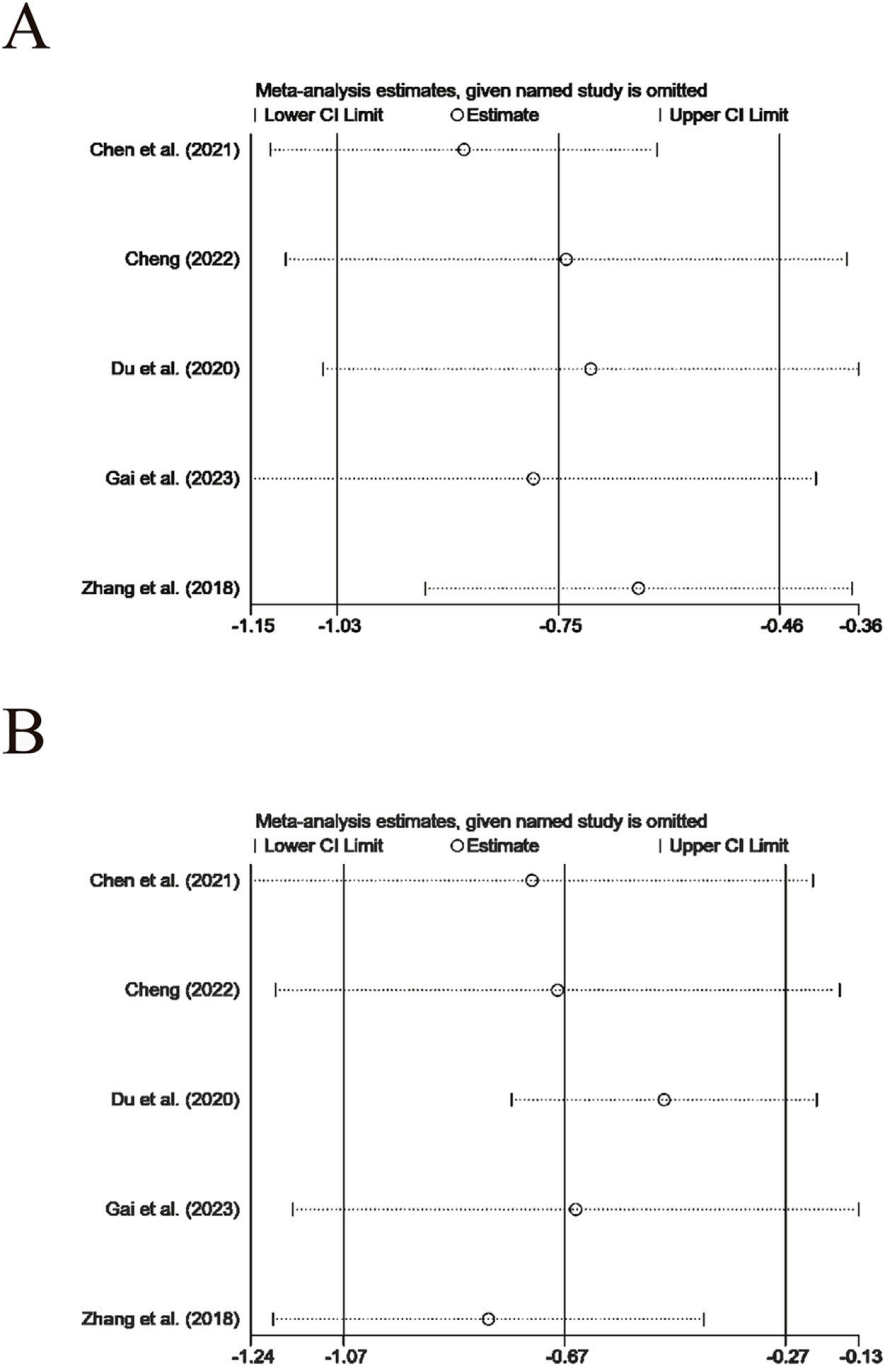
Sensitivity tests of primary outcomes: **(A)** TPOAb, **(B)** TgAb.

**FIGURE 10:**
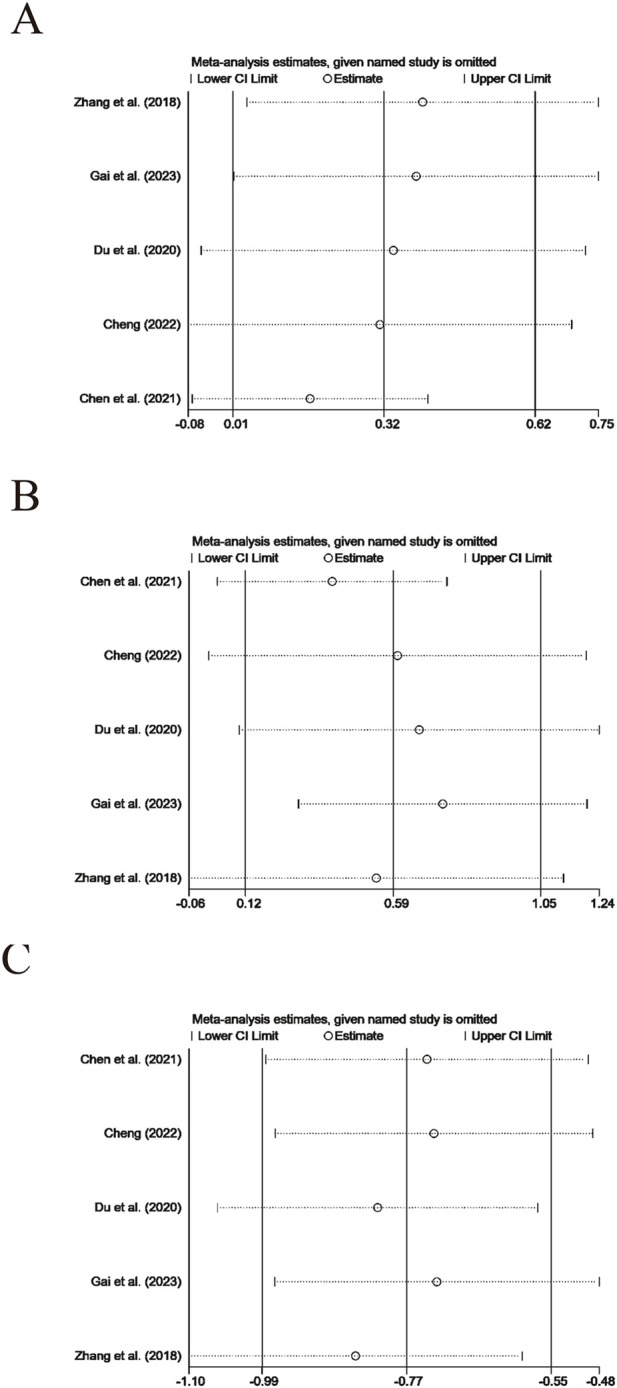
Sensitivity tests of secondary outcomes: **(A)** FT3, **(B)** FT4, and **(C)** TSH.

Systematic exclusion for sensitivity analysis of each trial indicated that, when comparing the intervention group receiving XYS preparations with the control group, apart from TCMs, and the comparison between XYSJW and OS preparations which involved only two trials and could not undergo leave-one-out analysis, the test results for other primary and secondary outcomes were mainly reliable (Figures 9, 10).

### 3.5 Trial sequential analysis

**FIGURE 11:**
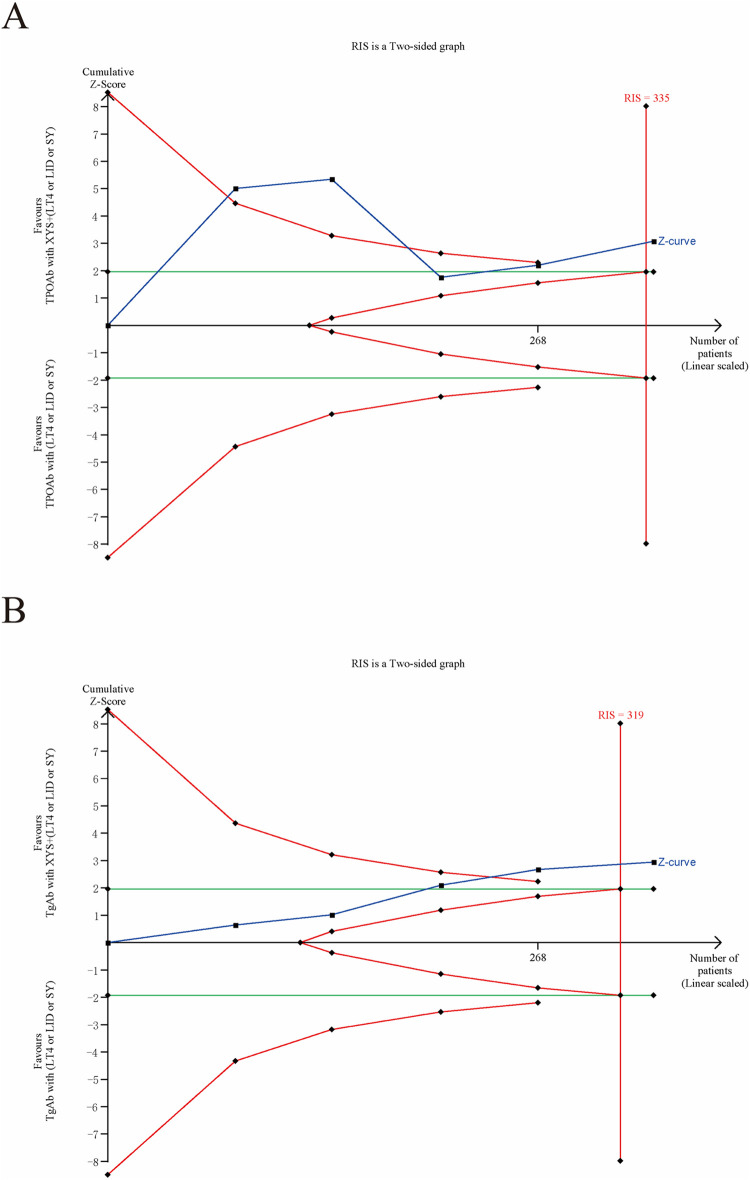
Trial Sequential Analysis of primary outcomes: **(A)** TPOAb, **(B)** TgAb.

**FIGURE 12:**
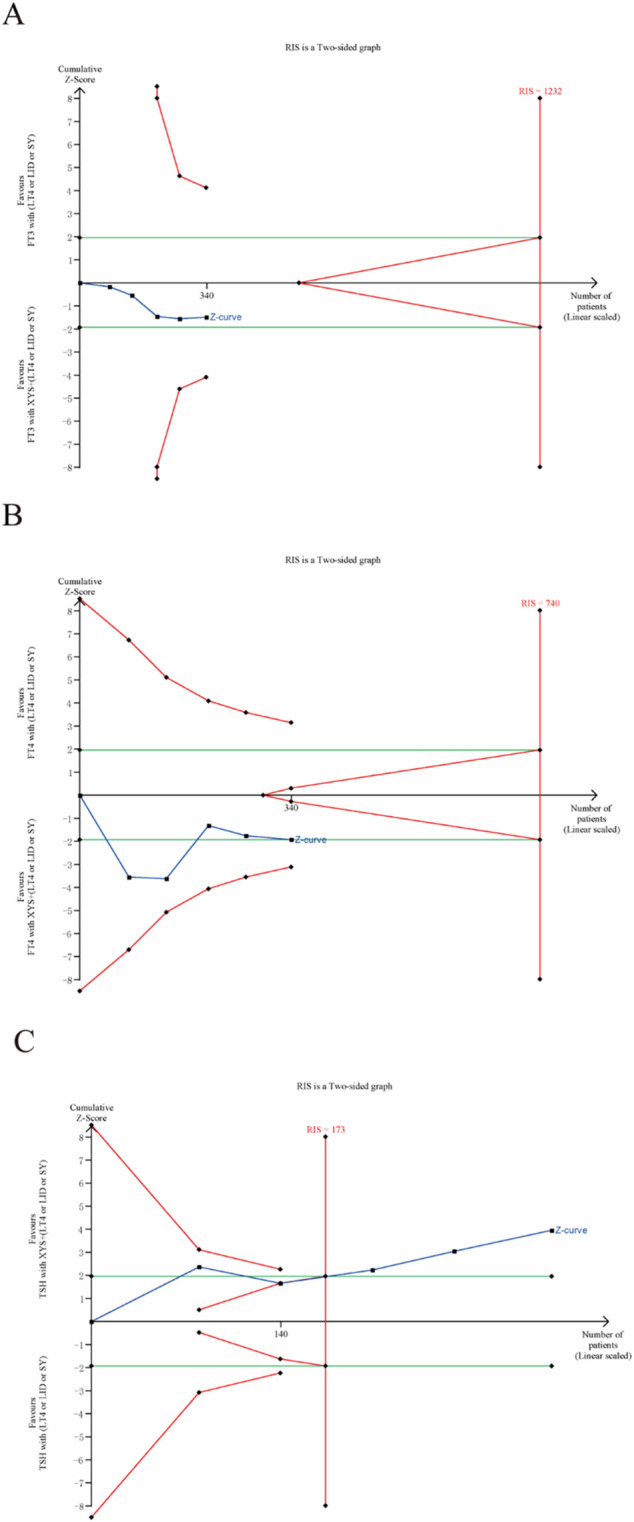
Trial Sequential Analysis of secondary outcomes: **(A)** FT3, **(B)** FT4 and **(C)** TSH.

The TSA of the primary and secondary outcomes in this study indicated that the efficacy of TPOAb, TgAb, and TSH exceeded the established thresholds for benefit and harm, reaching the required information size (Figure 11). This provides reliable evidence of the treatment’s impact. The cumulative z-curve for FT4 corresponded with alpha-spending and standard testing thresholds, approaching but not reaching the required information size, while the cumulative z-curve for FT3 did not reach the required information size (Figure 12).

### 3.6 Publication bias assessment

Due to the limited number of trials, which was fewer than 10, Egger’s test was not employed to evaluate publication bias in the meta-analysis. This study conducted a thorough and exhaustive search in both Chinese and English databases to reduce the likelihood of overlooking any tests. However, it is important to note that unpublished studies with negative results may not have been included. Due to the high heterogeneity within the TgAb subgroup (I^2^ = 55), a further Rosenthal fail-safe number N analysis was conducted on the TgAb results. The analysis indicated that 57 non-significant studies would need to be published to negate the meta-analysis findings (N/5k+10 > 1, N = 57, k = 5). This suggests that the weight of evidence for TgAb appears to be sufficiently robust to accommodate future results (Becker, 2005), and it is unlikely to be altered by publication bias.

### 3.7 Certainty of evidence

We evaluated the comprehensive evidence for primary and secondary outcomes using the GRADE system. The quality of evidence for all outcomes was rated as low or very low. Due to methodological flaws in the study results and the limited number of studies, the quality of evidence for all outcomes was downgraded by two levels. Additionally, considerable heterogeneity contributed to the downgrade of the quality of evidence for the outcomes. Please refer to Supplementary Table S12 for more information.

## 4 Discussion

### 4.1 Summary of evidence

XYS preparations (HHXY, DZXY, and GQXY) combined with LT4 significantly reduced TPOAb and TgAb levels in patients with HT, demonstrating superior efficacy compared to LT4 monotherapy. The combination of XY and LID therapy was beneficial in reducing TPOAb levels. However, the study did not observe a significant effect of XY combined with SY treatment on the recovery of TgAb. Compared to the novel OS preparations, XYSJW showed significantly enhanced efficacy in reducing TgAb levels, making it a potential therapeutic agent for HT patients with elevated TgAb. Treatments with XY, HHXY, GQXY, and DZXY facilitated the recovery of thyroid function in HT patients, primarily by reducing TSH levels, which is beneficial for patients with subclinical hypothyroidism characterized by elevated TSH. The study indicated that XY might be a superior choice for restoring thyroid hormone levels, particularly when combined with SY, which simultaneously aided in the recovery of serum FT3, FT4, and TSH. The combination of XY and LID also facilitated the recovery of serum FT4 and TSH. Interestingly, the combination therapy of the three XYS preparations with LT4 did not significantly increase FT3 and FT4 levels, suggesting that exogenous thyroid hormone supplementation may not be conducive to the natural recovery of thyroid function in HT patients. A noteworthy finding was that XYSJW was less effective in restoring TSH levels in HT patients compared to the OS preparations. Additionally, XYS preparations did not demonstrate significant effects in improving the symptoms of HT patients from the perspective of traditional Chinese medicine, which may be related to the disproportionate male-to-female ratio in the study population compared to the clinical characteristics of HT. HHXY was effective in reducing serum IL-6 levels, while XYSJW did not show significant benefits in reducing IL-6 when compared to the OS preparations.

### 4.2 Autoantibodies and thyroid function in Hashimoto’s thyroiditis patients

Hashimoto’s thyroiditis (HT) is an autoimmune disease characterized by thyroid-specific damage. The immune response targeting the thyroid gland utilizes thyroid peroxidase (TPO) and thyroglobulin (Tg) as the primary antigens for humoral and cytotoxic reactions (Ehlers and Schott, 2014). The concentrations of TPOAb and TgAb in the bloodstream are crucial to disease progression. Previous studies have indicated that elevated levels of TPOAb and TgAb in patients with HT are associated with a poorer quality of life (Djurovic et al., 2018). Hypothyroidism is a subsequent outcome of thyroid degeneration. The immune response in HT leads to the destruction of normal thyroid cell structure and impairs the stroma surrounding the affected thyroid follicles. In typical HT patients, the stroma is infiltrated by hematopoietic mononuclear cells, including lymphocytes, plasma cells, and macrophages. This group of lymphocytes continuously damages thyroid cells through direct contact (Caturegli et al., 2014). Consequently, hypothyroidism and subclinical hypothyroidism, characterized by elevated TSH levels, are often documented as responses to HT disease and may be associated with more harmful outcomes. Studies have shown that elevated TSH levels are correlated with increased risks of fatal stroke, heart failure, coronary events, and coronary mortality (Rodondi et al., 2010; Gencer et al., 2012; Chaker et al., 2015; Baumgartner et al., 2017). Pregnant women with subclinical hypothyroidism or hypothyroxinemia have an increased risk of premature birth (Korevaar et al., 2019). Additionally, infants born to these mothers have a significant association with reduced IQ, language impairments, or overall developmental abnormalities (Thompson et al., 2018). Research suggests that selenium supplementation and metformin may successfully reduce TPOAb and TgAb levels. However, reliable studies indicate that various treatment methods have drawbacks, including a significantly increased possibility of negative side effects (Wichman et al., 2016; Jia et al., 2020).

Thyroid hormone replacement therapy (THR) is an economically effective treatment for hypothyroidism caused by HT. However, it has specific limitations, including the inability of HT patients to fully restore physical activity (Lankhaar et al., 2021), the risk of overtreatment (Somwaru et al., 2009; Taylor et al., 2014), and the increased likelihood of atrial fibrillation, osteoporosis, and fractures due to overtreatment (Flynn et al., 2010). Therefore, it is imperative to explore innovative methods to reduce autoantibodies and restore thyroid function in patients with Hashimoto’s thyroiditis. The evaluation of this meta-analysis also includes the development of OS preparations. The results of this study indicate that XYS preparations, as adjunctive therapy for HT patients, may reduce TPOAb, TgAb, and TSH levels while potentially increasing FT3 and FT4 levels. Combination therapy with XYS preparations and LT4 may be more beneficial for reducing serum TPOAb and TgAb levels but less favorable for restoring thyroid function. Combination therapy with XY and LID or SY is more adept at restoring thyroid function but weaker in reducing thyroid autoantibodies compared to combination therapy with LT4. Compared to OS, XYSJW enhances the efficacy of reducing TgAb in HT patients but may be less effective in restoring TSH. However, currently, only XYSJW has been compared to OS, and more high-quality randomized controlled studies are needed to confirm these findings.

### 4.3 Cytokines in Hashimoto’s thyroiditis

The etiology of HT remains ambiguous. Autoreactive lymphocytes or external stimuli may potentially induce the premature demise of thyroid cells. Researchers have identified that the cytokines implicated in this pathway facilitate the advancement of HT. Research indicates that the dysregulation of chemokines and cytokines in the thyroid tissue and peripheral blood of individuals with Hashimoto’s thyroiditis, including pro-inflammatory agents such as interferon-gamma (IFN-g), interferon-alpha (IFN-a), and tumor necrosis factor-alpha (TNF-α), can aggravate thyroid damage and sustain chronic inflammation (Kemp et al., 2003; Domberg et al., 2008; Akeno et al., 2011; Rebuffat et al., 2013; Salzano et al., 2012). Individuals with autoimmune thyroiditis (AIT) and hypothyroidism demonstrate increased concentrations of IFN-γ-dependent chemokines, along with significant lympho-mononuclear cell infiltration, indicating that these chemokines may serve as more reliable indicators of severe thyroid inflammation (Antonelli et al., 2011a; Antonelli et al., 2011b). Research indicates that many cytokines, such as IL-1α, IL-1β, IL-2, IL-4, IL-6, IL-8, IL-10, IL-12, IL-13, IL-14, TNF-α, and IFN-γ, are found in thyroid follicular cells, enhancing the inflammatory response to nitric oxide and prostaglandins (Khan et al., 2015). Additionally, increased concentrations of IFN-γ and IL-2 in hypertensive pregnant women correlate with poorer pregnancy outcomes (Zhong et al., 2023).

The incidence of HT among COVID-19 patients has sparked renewed interest among researchers, as these patients produce an excess of inflammatory cytokines (IL-6, IL-1β, IFN-γ, TNF-α) (Mohammadi et al., 2023). Our meta-analysis uncovered limited but suggestive evidence of XYS preparations modulating cytokines. For instance, HHXY can reduce IL-6 levels, while XYSJW demonstrates a comparable ability to lower IL-6 levels as OS preparations. Results based on real-world studies also indicate that XYSJW can decrease the levels of proinflammatory cytokines IFN-γ, IL-2, and IL-6, while simultaneously increasing the concentration of the anti-inflammatory cytokine IL-10 in the serum of HT patients (Wu, 2023). This aligns with preclinical studies showing that XYS can inhibit IL-6 and TNF-α (Tan et al., 2015; Wan et al., 2020; Fang et al., 2020), DZXY can suppress IL-6 and TNF-α (Zhu et al., 2015), and HHXY can reduce IL-6, TNF-α, and IL-1β (Chen et al., 2024). Animal models further suggest that XYS can restore the Th1/Th2 balance through the upregulation of IL-10 and downregulation of IFN-γ/IL-2, thereby exerting an immunomodulatory effect (Zhang Y. et al., 2021). Regrettably, only two included studies reported IL-6 data, and none measured key cytokines such as TNF-α, IL-1β, or IFN-γ. This gap prevents a definitive conclusion on the immune mechanism of XYS. Existing evidence indicates that different XYS preparations do not significantly exhibit distinct effects on cytokines, possibly due to their shared five main herbal ingredients. However, variations in auxiliary components are bound to lead to differences in active metabolites, which is a worthy area for further exploration. Standardized cytokine analysis, including measurements at multiple time points, is necessary in future trials to capture dynamic immune responses. Integrating transcriptomic or proteomic analyses could further elucidate the specific pathways of the preparations.

### 4.4 Xiaoyao-san preparations: active metabolites and mechanism of action

#### 4.4.1 Inflammatory mechanisms in autoimmune thyroid disorders

The inflammatory immune response significantly influences the advancement of autoimmune thyroid disorders. Upon activation by inflammatory stimuli, CD4^+^ T cells, which are significant immune cells, differentiate into many Th cell subtypes, including Th1, Th2, Th17, follicular helper T (Tfh) cells, and regulatory T (Treg) cells. The disparity between Th1/Th2 and Trg17/Treg influences the pathogenesis of thyroid disorders and regulates immune inflammatory responses (Zhang L. et al., 2021). XYSJW may enhance the synthesis of the target protein ADAM17 by suppressing miR-326 expression in the thyroid tissue of rats with experimental autoimmune thyroiditis, hence hindering Th17 cell development and diminishing IL-17 mRNA expression (Li YN. et al., 2023). In clinical studies, XYSJW has demonstrated a significant ability to reduce the levels of IFN-γ, IL-2, and IL-6 while increasing IL-10 levels in patients with Hashimoto’s thyroiditis and chronic lymphocytic thyroiditis, thereby regulating the Th1/Th2 balance (Zhang Y. et al., 2021). This mechanism may underlie the immunomodulatory effects of XYSJW; however, it does not encompass all XYS preparations. This highlights a critical challenge in the research of traditional Chinese medicine: although all formulations share common core herbs (Chaihu, Shaoyao, Baizhu, Danggui and Fuling), variations in auxiliary ingredients and processing methods lead to distinct metabolite profiles.

#### 4.4.2 Core active metabolites in XYS preparations

Pharmacological investigations indicate that ferulic acid, atractylodin III (Geng et al., 2013), paeoniflorin (C_23_H_28_O_11_), glycyrrhizic acid (C_21_H_22_O_9_), glycyrrhizin (C_42_H_62_O_16_), and imperatorin (C_12_H_14_O_2_) (Shi et al., 2019) are the principal bioactive metabolites present in XYS. The HPLC-MS/MS study determined the principal active metabolites of DZXY as geniposide, ginsenoside Rg1, paeoniflorin, glycyrrhizin, saikosaponin a, saikosaponin d, saikosaponin b2, paeonol, and atractyloside III (Zhu et al., 2020; Lin, 2021). Volatile metabolites like menthone, menthol, 2-methoxy-4-vinylphenol, n-butylphenylpeptide, atractylodes, and costunolide have been recognized as possible active metabolites of HHXY (Huang et al., 2023). The chemical components mentioned above primarily originate from the herbal medicines commonly found in XYS preparations. Considering the addition of auxiliary components, in the *in vitro* component study of HHXY, 17 chemical components from Honghua and nine chemical components from Zaojiaoci were identified (Chen et al., 2024). Among the blood components of DZXY, six chemical components derived from Zhizi and one from Mudanpi were also discovered (Li M. et al., 2023).

#### 4.4.3 Mechanistic insights from metabolomics and proteomics

Moreover, metabolomics research indicates that XYS may mitigate liver damage by neutralizing free radicals, diminishing lipid peroxidation, and restoring metabolic pathways, including amino acid metabolism, the tricarboxylic acid cycle, and the urea cycle (Zhang et al., 2014). DZXY may augment excitability and demonstrate antidepressant effects by modulating several metabolic pathways, including phenylalanine, arachidonic acid, porphyrin, d-arginine, d-ornithine metabolism, steroid hormone biosynthesis, unsaturated fatty acid biosynthesis, and steroid biosynthesis (Chen, 2019). Proteomic analyses demonstrate that DZXY modulates the β-arr2-mediated signaling cascade by enhancing β-arr2 expression, hence contributing to the prevention and management of polycystic ovarian syndrome (Zhang et al., 2013). Both XYS and DZXY can exert their effects by modulating amino acid metabolism and lipid metabolism-related pathways. However, based on XYS’s regulation of amino acid metabolism, DZXY extends its regulatory scope to include aromatic amino acids (phenylalanine) and precursors of steroid hormones. This expansion potentially explains its functional evolution from basic metabolism towards neuroendocrine regulation. DZXY’s unique ability to regulate β-arr2 (Zhang et al., 2013) suggests that it may achieve multi-system regulation through GPCR signal reprogramming, a level of protein regulation that has not been reported in XYS research. In addition to restoring basic metabolic cycles (such as the TCA cycle and urea cycle), XYS also possesses the ability to neutralize free radicals, exerting a direct antioxidant effect that may benefit the treatment of organic metabolic injuries (such as chemical liver damage). Due to its dual functions in metabolic regulation and signal transduction modulation, DZXY may have advantages in the treatment of psychosomatic comorbidities (such as metabolic abnormalities concomitant with depression). However, the existing fingerprint evidence cannot cover all XYS preparations, and there is a lack of omics evidence related to the immunomodulatory effects of treating HT. Therefore, it is practically meaningful to continue exploring the mechanisms through which the common and specific components of XYS preparations may play a role in the treatment of HT.

#### 4.4.4 Mechanistic studies on shared active metabolites

We have observed that there are significantly more mechanistic studies on the monomeric active metabolites shared among different XYS preparations for the treatment of HT than there are on compound formulations.

Total glucosides of paeony (TGP) can regulate the expression of SOCS1 in rats with autoimmune thyroiditis, enhance the expression of JAK1, JAK2, STAT3, and STAT5, thereby affecting the JAK/STAT signaling pathway. This inhibits excessive inflammatory responses, reduces the levels of TGAb and TPOAb in rat serum, and alleviates inflammatory damage to thyroid tissue (Yu et al., 2023). Simultaneously, TGP can regulate the composition and species of intestinal microbiota in AIT rats, enhance the integrity of the intestinal mucosal barrier, perform intestinal-immune axis regulation (Mu et al., 2021), reduce the expression of Fas and FasL in rat thyroid tissue (inhibiting apoptosis), and regulate the immunological balance of Treg/Th17 (Tong, 2018; Tong et al., 2019; Mu, 2018). This may be achieved by promoting the differentiation of Treg cells and increasing the proportion of CD4^+^ CD25^+^ Foxp3^+^ Treg cells (Yu et al., 2017).

The active ingredient of Chaihu, saikosaponin-D, can regulate the imbalance of M1/M2 (promoting M2 macrophage polarization) in the spleen of mice with Hashimoto’s thyroiditis. It inhibits the expression of Th1-type cytokine IFN-γ and local Th17-type cytokine IL-17, corrects the imbalance of Th1/Th2 and Th17/Treg, and reduces the severity of Hashimoto’s thyroiditis in mice (Du et al., 2022). Saikosaponin-A can significantly reduce the levels of TgAb, TPOAb, TSH, and T3 in the blood of AIT rats, as well as the protein concentration of TNF-α and IL-6 in thyroid tissue. It inhibits the NLRP3 inflammasome, reduces the expression of NLRP3, ASC, and Cleaved-Caspase-1, and blocks the mature release of IL-1β (Pan et al., 2022). The flavonoid compound quercetin in Bupleurum can enhance the AP-1 activity of rat thyroid follicular epithelial cells (FRTL-5 cells) *in vitro* (Giuliani, 2019), possibly reducing oxidative damage through antioxidant mechanisms, thereby playing a protective role in thyroid tissue. *In vivo*, it can significantly reduce the absorption of radioactive iodine by Sprague-Dawley rats (Giuliani et al., 2014).

Ferulic acid, a biologically active component of Dangui, exerts a regulatory effect on propylthiouracil-induced hypothyroidism in rats by reducing IL-6 levels, inhibiting inflammatory cascade reactions, improving thyroid function (increasing T3 and T4 while decreasing TSH), and modulating lipid metabolism (levels of total cholesterol, triglycerides, high-density lipoprotein/low-density lipoprotein). Simultaneously, ferulic acid has been found to lower aspartate aminotransferase (AST), alanine aminotransferase (ALT), uric acid, and creatinine levels, demonstrating its ability to improve liver and kidney function as well as metabolic disorders (Rongala S et al., 2024).

On the other hand, Beta-elemene, an active ingredient in Baizhu, exhibits antitumor and immune microenvironment-modulating properties. It enhances the cleaved expression of Caspase-9, reduces the production of bcl-2, induces tumor cell apoptosis, and decreases the expression of vascular endothelial growth factor to reduce tumor angiogenesis. Beta-elemene significantly inhibits the proliferation of DTC (differentiated thyroid cancer) cells, lowers OCR, ECAR, maximal glycolytic capacity, maximal respiratory capacity, and ATP production, restricts the invasive capacity of DTC cells, indirectly suppresses the immunosuppressive microenvironment, and reduces immune evasion (Zhao et al., 2020).

The water-soluble polysaccharide from Poria cocos (PCP) has demonstrated significant effects in reducing the levels of proinflammatory cytokines such as TNF-α, IL-1β, IL-6, and IL-8 in animal models of prostatic inflammation. It also inhibits the expression of nitric oxide synthase (iNOS) and malondialdehyde (MDA), thereby mitigating oxidative stress damage and protecting the rat prostate from oxidative-inflammatory cascade disruption (Liu et al., 2020). Interestingly, *in vitro* experiments using macrophages (RAW264.7) have shown that PCP-2, a component of Poria cocos polysaccharides, activates innate immunity via the TLR4/MyD88/NF-κB pathway, promoting the secretion of NO, TNF-α, IL-6, and IL-17A, and enhancing immune surveillance function. In an immunosuppression model induced by cyclophosphamide, PCP-2 supports the recovery of systemic immune homeostasis by enhancing humoral immunity (increasing serum IgG, IgA, and IgM levels), regulating adaptive immunity (increasing the proportion of CD3^+^ CD4^+^ T cells), and promoting thymic/spleen development (Lv et al., 2024). Current research indicates that PCP possesses a multi-target immunomodulatory mechanism and contradictory bidirectional immunomodulatory properties. However, the direction and specific role of its dynamic regulation in the treatment of HT remain unclear.

#### 4.4.5 Specific components and mechanisms in different XYS preparations

In addition to shared components, various XYS preparations contain specific ingredients optimized for treating other symptoms that patients may experience. Under the guidance of traditional Chinese medicine theory, HHXY is effective for HT patients with thyroid nodules, DZXY is more suitable for HT patients with transient hypermetabolism and psychiatric symptoms, GQXY is beneficial for HT patients with hypometabolism and psychiatric symptoms, and XYSJW is adept at treating HT patients with thyroid enlargement. Due to a lack of evidence, this study was unable to verify differences in these concomitant symptoms, and fingerprint or omics methods were not feasible. Therefore, we attempt to explain such differences by reviewing representative specific active ingredients in different XYS preparations.

##### 4.4.5.1 DZXY-specific components

Mudanpi is a key specific component in DZXY, and its active ingredient, paeonol (PAE), has been proven to promote the Th17/Treg balance in AIT rats by inhibiting the HMGB1/RAGE pathway, thereby protecting against pathological damage in autoimmune thyroid rats (Zhang et al., 2025). Zhizi is another specific herbal ingredient in DZXY. Its main components, geniposide and crocin, can reduce levels of proinflammatory factors (TNF-α, IL-1β, IL-6, IL-18) and alleviate inflammatory damage by inhibiting the NF-κB pathway and NLRP3 inflammasome (Bu and Fan, 2019; Tang and Shen, 2019; Sun et al., 2019; Zhang L. et al., 2021). Simultaneously, geniposide activates the AMPK pathway, inhibits hepatic gluconeogenesis (via FOXO1), lowers blood glucose levels, and regulates energy metabolism pathways to improve abnormal metabolic hyperactivity (Guo et al., 2016; Yang et al., 2018). Furthermore, the iridoid glycosides in Gardenia jasminoides enhance synaptic plasticity by regulating the AMPAR-mTOR pathway, promote hippocampal neuron regeneration, downregulate excessive activation of the glutamate receptor (GluA1), reduce neuronal excitability toxicity, protect nerve function, and thereby improve depressive symptoms (Xiao et al., 2018).

##### 4.4.5.2 HHXY-specific components

Honghua is the primary specific plant component of HHXY, and its main active ingredient, hydroxyl safflor yellow A (HSYA), significantly reduces TNF-α and IL-17 levels in experimental autoimmune hepatitis mouse models (Peng et al., 2024), thereby alleviating autoimmune attacks. In animal models of alcoholic liver disease, HSYA regulates the STAT3/NF-κB signaling pathway, inhibits inflammatory cascade reactions, reduces lymphocyte infiltration and inflammatory damage, activates the Keap1/Nrf2 pathway, and reduces oxidative damage (Wang et al., 2024). Additionally, safflower extracts (including HSYA, HSYB, etc.) can inhibit the proliferation pathway (p38 MAPK), reducing cancer cell proliferation (Zhang et al., 2019). They also downregulate the expression of VEGF and MMP-9 (Kaçaroğlu et al., 2023; Chen et al., 2020), inhibiting angiogenesis and tumor metastasis. By inhibiting the PD-1/PD-L1 axis and chemokine secretion (Ji et al., 2024), these extracts restore CD8^+^T cell function and enhance immune surveillance, thereby modulating the tumor microenvironment. Activation of Caspase-3/9 and upregulation of the Bax/Bcl-2 ratio (Wang et al., 2022) promote mitochondrial-dependent cancer cell apoptosis. Finally, inhibition of the HIF-1α/SLC7A11 pathway (Zhu et al., 2024) blocks the Warburg effect and iron metabolism imbalance, inducing ferroptosis in cancer cells.

Zaojiaoci is another specific herbal component in HHXY. Studies have demonstrated that the water extract of Gleditsia Spina significantly reduces the production of inflammatory cytokines such as TNF-α, IL-1β, and IL-6, and inhibits their mRNA expression (Li et al., 2016). Relevant mechanistic studies have shown that it indirectly alleviates inflammatory responses by blocking the STAT3/STAT6 signaling pathway and reducing the generation of IL-4/IL-13-induced mucin protein MUC5AC (Jung et al., 2023). Simultaneously, evidence suggests that the water extract of Gleditsia Spina can significantly inhibit tumor size in prostate cancer (PC-3 cell xenografts) (Ryu et al., 2016), block the AGE-RAGE signaling pathway, and induce apoptosis in pancreatic cancer cells (Duan et al., 2023). The ethanol extract of Gleditsia Spina exhibits inhibitory effects on breast cancer (MCF7) and gastric cancer (SNU-5) cell lines (Yu et al., 2019; Lee et al., 2013). In animal experiments, it can induce cancer cell apoptosis and significantly protect the liver morphology in rat hepatocellular carcinoma (Cai et al., 2019). Among them, the monomeric compounds p-hydroxyl cinnamicaldehyde, trans-coniferyl aldehyde, and sinapaldehyde may be the main components inhibiting the growth of hepatoma cell line (SK-hep1) (Yu et al., 2015). Due to the specific efficacy of Honghua and Zaojiaoci, HHXY is also commonly used in the treatment of various cancers.

##### 4.4.5.3 GQXY-specific components

Huangqi is the main specific ingredient of GQXY. Its active component, Astragalus polysaccharides (APS), significantly reduces the levels of serum proinflammatory cytokines IL-6, IL-12, and IL-17, while enhancing the expression of anti-inflammatory cytokines IL-10 and TGF-β1. This helps to inhibit thyroid lymphocyte infiltration and alleviate pathological damage in autoimmune thyroiditis (AIT) (Qiu et al., 2024). Astragaloside IV, on the other hand, can suppress the NLRP3 inflammasome and Caspase-1 pathway, reducing the release of inflammatory cytokines and mitigating the occurrence of chronic inflammation (Yuan et al., 2022). Furthermore, APS can activate the AMPK/mTOR pathway, enhancing insulin sensitivity, improving glucose and lipid metabolism, and relieving symptoms of hypometabolism (Ye et al., 2022).

Roucongrong is another specific component of GQXY, whose active ingredient, Cistanche deserticola polysaccharide, reduces inflammatory responses by inhibiting the release of proinflammatory factors such as IL-6 and TNF-α from macrophages, while simultaneously promoting the proliferation of macrophages (RAW264.7) and enhancing immune regulatory capabilities (Ailadan et al., 2021). Additionally, echinacoside decreases the release of neuroinflammatory factors by inhibiting the NLRP3/Caspase-1/IL-1β pathway (Gao et al., 2020). Both Cistanche deserticola polysaccharide and total glycosides upregulate the expression of synapsin, growth-associated protein 43 (GAP43), PSD-95, and BDNF, enhancing synaptic plasticity in the hippocampal region and improving learning and memory impairments (Yin et al., 2014; Xing et al., 2019; Liu et al., 2022). Together, these components exert neuroprotective effects, enhance synaptic plasticity, and alleviate psychiatric symptoms. As such, Cistanche deserticola has also been proven effective in ameliorating symptoms associated with neurodegenerative diseases, such as AD (Qiu and Liu, 2022) and PD (Gao et al., 2020).

##### 4.4.5.4 XYSJW-specific components

Kunbu and Haizao are specific auxiliary components in XYSJW, and these two traditional Chinese medicines have a history of treating goiter that can be traced back to the Ming Dynasty’s specialized book on surgical medicine, “Wai Ke Zheng Zong.” Components in kunbu, such as squalene, δ-tocopherol, and phytol, alleviate inflammatory responses by inhibiting the release of proinflammatory factors like NO, PGE2, IL-6, IL-1β, and TNF-α from LPS-induced RAW264.7 macrophages (Shao, 2024). Kombu polysaccharide (LJP61A) suppresses macrophage foam cell formation and reduces the release of inflammatory mediators by regulating lipid metabolism and inflammatory signaling pathways (Li Q. M. et al., 2018). Additionally, various kombu polysaccharide components, including fucoidan, WPS-two to one, and others, exhibit inhibitory effects on abnormal cell proliferation (Peng et al., 2013; Teruya et al., 2019; Niyonizigiye et al., 2019; Lu et al., 2018; Xue et al., 2018).

Trehalose, a component found in haizao, can activate the nuclear translocation of Transcription Factor EB (TFEB) by slightly increasing lysosomal pH. This process promotes autophagosome biogenesis and facilitates the clearance of damaged organelles and inflammatory mediators (Jeong et al., 2021). In a model of viral myocarditis, trehalose was observed to induce autophagy in B-cells through the AMPK/ULK1 signaling pathway, thereby reducing inflammatory damage (Wei et al., 2023). Additionally, trehalose promotes the polarization of macrophages towards the anti-inflammatory M2 phenotype through energy metabolism reprogramming, restoring immune homeostasis (Yu et al., 2023). Furthermore, in a model of silicosis, trehalose alleviated lysosomal dysfunction and reduced tissue structural damage by activating the TFEB-mediated autophagy-lysosome system (He et al., 2016; Tan et al., 2015).

Presently, numerous papers examine the active metabolites of individual formulations; however, there is a deficiency in research about the synergistic effects between each botanical drug metabolite in XYS preparations. Assessing whether these botanical drug metabolites exhibit identical mechanisms of anti-immunoinflammatory effects as evidenced by their combined application is challenging. The investigation on the inconsistencies of active metabolites among various XYS preparations is insufficient. Omics research primarily concentrates on specific dose forms; however, there is a significant deficiency of papers regarding the treatment of thyroid disorders with XYS preparations. This domain warrants additional scrutiny.

### 4.5 Strengths and limitations

This study represents a preliminary evaluation and meta-analysis of the efficacy of XYS preparations in the treatment of Hashimoto’s thyroiditis. We conducted a comprehensive search across eight databases to ensure the inclusion of all relevant randomized controlled trials (RCTs). A comprehensive report detailing our methodology has been provided to enable others to reproduce our results. We evaluated thyroid function (FT3, FT4, and TSH) and thyroid autoantibodies (TPOAb and TgAb) in patients with Hashimoto’s thyroiditis. This may offer a unique alternative treatment option for patients diagnosed with HT, regardless of their hypothyroid status, particularly for those with elevated TSH levels. Additionally, we examined the effects of XYS preparations on cytokines. We summarized and analyzed the potential pharmacological effects of XYS on HT and its associated inflammatory markers. This study aims to provide new and effective treatment options for HT through traditional Chinese medicine.

However, our study is subject to several specific limitations. Initially, language constraints of the researchers limited our search to Chinese and English databases. All studies included in the analysis were conducted in China and published in Chinese. Therefore, the findings cannot be extrapolated to the global population. Secondly, it must be emphasized that all studies included in this analysis have limited sample sizes, with participant numbers ranging from a minimum of 60 to a maximum of 162 in single-center randomized controlled trials. Only one study elucidated the criteria for determining the sample size (Li YN., 2021). The methodological rigor of the publications included in the study is somewhat lacking. Only one trial employed a double-blind strategy, and none of the studies adequately disclosed trial registration, conflicts of interest, or other relevant information. The internal validity of the study results may be influenced by potential biases and confounding variables related to participant selection. Due to the limited number of studies included in the meta-analysis (only seven studies), the use of Egger’s test to evaluate publication bias was not feasible, and the fail-safe number N could only serve as an auxiliary analysis, thus complicating the identification of publication bias. TSA was used to evaluate the effectiveness of primary and secondary outcomes. However, it does not acknowledge the scientific rigor deficiencies of the experiments and does not address errors that may arise from reporting biases.

Three trials aimed at identifying adverse events failed to assess the safety of the XYS preparations. The duration of medication administration in this trial ranged from 12 to 24 weeks. Without long-term post-treatment monitoring, it will be impossible to determine whether the XYS preparations have a lasting impact on HT after discontinuation. In numerous RCTs, XYS preparations, as part of combination therapy, have demonstrated a significant reduction in the incidence of adverse reactions. The “Clinical Application Guidelines for Chinese Patent Medicines in the Treatment of Depressive Disorders,” published in 2022, recommends the use of XY in combination with antidepressants (such as venlafaxine and paroxetine) for the treatment of mild to moderate depressive disorders to improve depressive and anxiety symptoms (recommendation evidence level: 1C) (Standardization Project Team, 2023). For patients with depressive disorders, compared to antidepressants alone, the combination of XY and antidepressants can reduce adverse reactions in the central and peripheral nervous systems (including dizziness, headache, insomnia, and lethargy) as well as the sympathetic/parasympathetic nervous system (including dry mouth and excessive sweating). However, this does not aid in evaluating the adverse reactions caused by the use of XYS preparations alone. No adverse reactions were reported in this study, leading to an extended discussion. We found that in the treatment of hyperplasia of mammary glands (HMG), HHXY has a lower incidence of adverse reactions compared to tamoxifen. It is recommended by the standardized project team of the “Clinical Application Guidelines for Chinese Patent Medicines in the Treatment of Dominant Diseases” and included in the “Guidelines for the Application of Chinese Patent Medicines in the Treatment of Hyperplasia of Mammary Glands (2021)” as “Level C” evidence. The adverse reactions mainly involve gastrointestinal discomfort and increased menstrual flow (Standardization Project Team, 2022). XY is also recommended by the guidelines as “Level D″ evidence, and no authoritative reports of adverse reactions have been found in expert consensus opinions. However, we discovered three case reports of adverse reactions caused by XY. All patients were female and took XY for gynecological diseases, gastrointestinal discomfort, and gastrointestinal discomfort after a cold. After taking XY, they experienced reactions such as dizziness, nausea, vomiting, palpitations, profuse sweating, and elevated blood pressure. No abnormalities were found during hospital visits and examinations, and they recovered after anti-allergic treatment (Li and Zhu, 2000). From the perspective of traditional Chinese medicine, these three patients may have experienced adverse reactions due to medication inconsistency and individual physical differences. Therefore, despite the preliminary safety data of XYS preparations in the treatment of HT being favorable, existing studies have not incorporated an active reporting mechanism for adverse events, and the follow-up period has been relatively short. Systematic long-term evaluation is still needed to support clinical promotion. It must be acknowledged that only two studies evaluated cytokines (such as IL-6), which limited our ability to delve deeper into the immune mechanism. There was significant heterogeneity among XYS preparations, and the limited number of included studies precluded more meaningful subgroup analyses. Therefore, caution is advised when evaluating the outcomes derived from these meta-analyses.

### 4.6 Implications

Since its discovery by Japanese researchers, Hashimoto’s thyroiditis (HT) has remained a significant therapeutic challenge in thyroid diseases. The primary clinical approaches to managing hypothyroidism include adhering to a low-iodine diet and using levothyroxine (LT4) replacement therapy. Additionally, selenium supplementation has recently emerged as a potentially beneficial strategy. However, these methods have limitations in treatment efficacy and may be associated with certain side effects. Prolonged LT4 replacement therapy may lead to persistent cognitive impairment and decreased overall wellbeing in HT patients (Djurovic et al., 2018). Furthermore, selenium supplementation might increase the incidence of adverse reactions (Korevaar et al., 2019).

Our comprehensive survey and meta-analysis indicate that compared to a low-iodine diet, selenium supplementation, and LT4 replacement therapy, the concomitant administration of XYS preparations can more effectively reduce TPOAb and TgAb levels in individuals with HT. When compared to the novel OS preparation, XYS maintains its efficacy in lowering TgAb levels in patients with Hashimoto’s thyroiditis. Apart from the comparison with OS, all treatment regimens using XYS preparations significantly reduced blood TSH levels. The results of this study suggest that XYS preparations have a certain restorative effect on thyroid function in patients with Hashimoto’s thyroiditis. The TSA results demonstrate that XYS preparations can significantly reduce TPOAb, TgAb, and TSH, providing strong evidence for its efficacy. This can serve as a reference for treatment plans; however, clinicians must exercise caution based on the specific conditions of the patients, considering the low methodological quality of the studies, insufficient internal validity of the data, and unclear safety impacts. Follow-up studies should employ randomized and double-blind methods to confirm these findings. The structure of the randomized controlled trial (RCT) must align with the CONSORT guidelines (Schulz et al., 2010). Furthermore, further research is needed to investigate the effects of XYS preparations on FT3, FT4, and cytokines and to increase the sample size to evaluate adverse reactions. Analyzing differences in botanical drug metabolites is crucial for future pharmacological process research.

## 5 Conclusion

Recent studies have suggested that the combination of XYS preparations and LT4 in the treatment of HT may be beneficial in reducing TPOAb, TgAb, and TSH levels, while XY combined with LID or SY therapy is more adept at restoring thyroid function in HT patients. XYSJW has demonstrated greater efficacy compared to OS preparations in addressing elevated TgAb levels. However, the benefits of XYSJW in restoring thyroid hormone levels in HT patients may not exceed those of OS preparations. It is important to note that our analysis is constrained by the limited number of included studies and their methodological quality. To further validate the findings of this study, it is necessary to conduct large-sample, multi-center, high-quality RCT research.

## Data Availability

The original contributions presented in the study are included in the article/Supplementary Material, further inquiries can be directed to the corresponding author.
