# Supplementary material for: Efficacy of Xiaoyao-san preparations in treating Hashimoto’s thyroiditis: a meta-analysis and systematic review
Source: Front Pharmacol. 2025 Jun 13;16:1528506. doi: 10.3389/fphar.2025.1528506 (PMC12202410; doi:10.3389/fphar.2025.1528506)
Supplement: Supplementary file 1 [file Supplementaryfile3.docx]

**Efficacy of Xiao-yao San preparations in treating Hashimoto's thyroiditis: a meta-analysis and systematic review**

**Supplementary Files 1**

[Table S1 PRISMA 2020 Checklist 1](#_Toc187760359)

[Table S2 Systematic search detail (record number: 2024.09.30) 6](#_Toc187760360)

[Table S3 Specific review annotations for the 29 deleted articles after duplication. 10](#_Toc187760361)

[Table S4. Summary of Botanical Drugs Information 12](#_Toc187760362)

[Table S5 Details of the *Xiao-yao San* preparations used in the included studies 15](#_Toc187760363)

[Table S6 Ingredients and Preparation methods of Chinese polyherbal preparation^*^ 19](#_Toc187760364)

[Table S7 Identification, Inspection and Content Determination of Chinese polyherbal preparation* 22](#_Toc187760365)

[Table S8 Botanical drugs of Xiao-yao San preparations and daily intake measurement* 28](#_Toc187760366)

[Table S9 Subgroup analysis of TPOAb, TgAb, FT3, FT4 and TSH 32](#_Toc187760367)

[Table S10 GRADE Summary of Outcomes for *Xiao-yao San* preparations Combined with LID/SY/LT4 compared to LID/SY/LT4/OS for HT 33](#_Toc187760368)

# Table S1 PRISMA 2020 Checklist

| **Section and Topic** | **Item #** | **Checklist item** | **Location where item is reported** |
| --- | --- | --- | --- |
| **TITLE** | | |  |
| Title | 1 | Identify the report as a systematic review. | Title division |
| **ABSTRACT** | | |  |
| Abstract | 2 | See the PRISMA 2020 for Abstracts checklist. | The Abstract part |
| **INTRODUCTION** | | |  |
| Rationale | 3 | Describe the rationale for the review in the context of existing knowledge. | 1 Introduction |
| Objectives | 4 | Provide an explicit statement of the objective(s) or question(s) the review addresses. | 1 Introduction |
| **METHODS** | | |  |
| Eligibility criteria | 5 | Specify the inclusion and exclusion criteria for the review and how studies were grouped for the syntheses. | 2 Materials and methods  2.1-2.2 |
| Information sources | 6 | Specify all databases, registers, websites, organisations, reference lists and other sources searched or consulted to identify studies. Specify the date when each source was last searched or consulted. | 2 Materials and methods 2.3 |
| Search strategy | 7 | Present the full search strategies for all databases, registers and websites, including any filters and limits used. | 2 Materials and methods 2.3 |
| Selection process | 8 | Specify the methods used to decide whether a study met the inclusion criteria of the review, including how many reviewers screened each record and each report retrieved, whether they worked independently, and if applicable, details of automation tools used in the process. | 2 Materials and methods 2.4 |
| Data collection process | 9 | Specify the methods used to collect data from reports, including how many reviewers collected data from each report, whether they worked independently, any processes for obtaining or confirming data from study investigators, and if applicable, details of automation tools used in the process. | 2 Materials and methods 2.4 |
| Data items | 10a | List and define all outcomes for which data were sought. Specify whether all results that were compatible with each outcome domain in each study were sought (e.g. for all measures, time points, analyses), and if not, the methods used to decide which results to collect. | 2 Materials and methods 2.4 |
|  | 10b | List and define all other variables for which data were sought (e.g. participant and intervention characteristics, funding sources). Describe any assumptions made about any missing or unclear information. | 2 Materials and methods 2.4 |
| Study risk of bias assessment | 11 | Specify the methods used to assess risk of bias in the included studies, including details of the tool(s) used, how many reviewers assessed each study and whether they worked independently, and if applicable, details of automation tools used in the process. | 2 Materials and methods 2.5 |
| Effect measures | 12 | Specify for each outcome the effect measure(s) (e.g. risk ratio, mean difference) used in the synthesis or presentation of results. | 2 Materials and methods 2.6 |
| Synthesis methods | 13a | Describe the processes used to decide which studies were eligible for each synthesis (e.g. tabulating the study intervention characteristics and comparing against the planned groups for each synthesis (item #5)). | 2 Materials and methods 2.6 |
|  | 13b | Describe any methods required to prepare the data for presentation or synthesis, such as handling of missing summary statistics, or data conversions. | 2 Materials and methods 2.6 |
|  | 13c | Describe any methods used to tabulate or visually display results of individual studies and syntheses. | 2 Materials and methods 2.6 |
|  | 13d | Describe any methods used to synthesize results and provide a rationale for the choice(s). If meta-analysis was performed, describe the model(s), method(s) to identify the presence and extent of statistical heterogeneity, and software package(s) used. | 2 Materials and methods 2.6 |
|  | 13e | Describe any methods used to explore possible causes of heterogeneity among study results (e.g. subgroup analysis, meta-regression). | 2 Materials and methods 2.6 |
|  | 13f | Describe any sensitivity analyses conducted to assess robustness of the synthesized results. | 2 Materials and methods 2.6 |
| Reporting bias assessment | 14 | Describe any methods used to assess risk of bias due to missing results in a synthesis (arising from reporting biases). | 2 Materials and methods 2.6 |
| Certainty assessment | 15 | Describe any methods used to assess certainty (or confidence) in the body of evidence for an outcome. | 2 Materials and methods 2.7-2.8 |
| **RESULTS** | | |  |
| Study selection | 16a | Describe the results of the search and selection process, from the number of records identified in the search to the number of studies included in the review, ideally using a flow diagram. | 3 Results 3.1 Figure 1 |
|  | 16b | Cite studies that might appear to meet the inclusion criteria, but which were excluded, and explain why they were excluded. | 3 Results 3.1 Table S3 |
| Study characteristics | 17 | Cite each included study and present its characteristics. | 3 Results 3.2 Table 2. |
| Risk of bias in studies | 18 | Present assessments of risk of bias for each included study. | 3 Results 3.3 Figure 2 |
| Results of individual studies | 19 | For all outcomes, present, for each study: (a) summary statistics for each group (where appropriate) and (b) an effect estimate and its precision (e.g. confidence/credible interval), ideally using structured tables or plots. | Figure 3-8. |
| Results of syntheses | 20a | For each synthesis, briefly summarize the characteristics and risk of bias among contributing studies. | 3 Results 3.4 |
|  | 20b | Present results of all statistical syntheses conducted. If meta-analysis was done, present for each the summary estimate and its precision (e.g. confidence/credible interval) and measures of statistical heterogeneity. If comparing groups, describe the direction of the effect. | 3 Results 3.4 Figure 3-5 |
|  | 20c | Present results of all investigations of possible causes of heterogeneity among study results. | 3 Results 3.4 Figure 3-5.  Table S9. |
|  | 20d | Present results of all sensitivity analyses conducted to assess the robustness of the synthesized results. | 3 Results 3.4 Figure 9-10 |
| Reporting biases | 21 | Present assessments of risk of bias due to missing results (arising from reporting biases) for each synthesis assessed. | 3 Results 3.5-3.6  Figure 11-12 |
| Certainty of evidence | 22 | Present assessments of certainty (or confidence) in the body of evidence for each outcome assessed. | 3 Results 3.7 Figure 11-12 |
| **DISCUSSION** | | |  |
| Discussion | 23a | Provide a general interpretation of the results in the context of other evidence. | 4 Discussion 4.1-4.4 |
|  | 23b | Discuss any limitations of the evidence included in the review. | 4 Discussion 4.5 |
|  | 23c | Discuss any limitations of the review processes used. | 4 Discussion 4.5 |
|  | 23d | Discuss implications of the results for practice, policy, and future research. | 4 Discussion 4.6 |
| **OTHER INFORMATION** | | |  |
| Registration and protocol | 24a | Provide registration information for the review, including register name and registration number, or state that the review was not registered. | Systematic Review Registration |
|  | 24b | Indicate where the review protocol can be accessed, or state that a protocol was not prepared. | Systematic Review Registration |
|  | 24c | Describe and explain any amendments to information provided at registration or in the protocol. | 2 Materials and methods |
| Support | 25 | Describe sources of financial or non-financial support for the review, and the role of the funders or sponsors in the review. | Funding |
| Competing interests | 26 | Declare any competing interests of review authors. | Acknowledgements |
| Availability of data, code and other materials | 27 | Report which of the following are publicly available and where they can be found: template data collection forms; data extracted from included studies; data used for all analyses; analytic code; any other materials used in the review. | Supplementary material |

# Table S2 Systematic search detail (record number: 2024.09.30)

| **The search strategy for PubMed** | | |
| --- | --- | --- |
| **Sequence** | **Search terms** | **Hits** |
| #1 | ((((((((((((((((((((((Hashimoto Disease[MeSH Terms]) OR (Disease, Hashimoto[Title/Abstract])) OR (Hashimoto Struma[Title/Abstract])) OR (Hashimoto Thyroiditis[Title/Abstract])) OR (Hashimoto Thyroiditides[Title/Abstract])) OR (Thyroiditides, Hashimoto[Title/Abstract])) OR (Thyroiditis, Hashimoto[Title/Abstract])) OR (Hashimoto's Syndrome[Title/Abstract])) OR (Hashimoto Syndrome[Title/Abstract])) OR (Hashimoto's Syndromes[Title/Abstract])) OR (Hashimotos Syndrome[Title/Abstract])) OR (Syndrome, Hashimoto's[Title/Abstract])) OR (Syndromes, Hashimoto's[Title/Abstract])) OR (Hashimoto's Struma[Title/Abstract])) OR (Chronic Lymphocytic Thyroiditis[Title/Abstract])) OR (Chronic Lymphocytic Thyroiditides[Title/Abstract])) OR (Lymphocytic Thyroiditides, Chronic[Title/Abstract])) OR (Lymphocytic Thyroiditis, Chronic[Title/Abstract])) OR (Thyroiditides, Chronic Lymphocytic[Title/Abstract])) OR (Thyroiditis, Chronic Lymphocytic[Title/Abstract])) OR (Hashimoto's Disease[Title/Abstract])) OR (Disease, Hashimoto's[Title/Abstract])) OR (Hashimotos Disease[Title/Abstract]) | 13,649 |
| #2 | (((((((((((xiaoyao[Supplementary Concept]) OR (danzhi xiaoyao[Supplementary Concept])) OR (kamisyoyo san[Supplementary Concept])) OR (XYDN[Title/Abstract])) OR (xiao-yao[Title/Abstract])) OR (kami-shoyo-san[Title/Abstract])) OR (kami-soyo-san[Title/Abstract])) OR (TJ-24[Title/Abstract])) OR (TJ24[Title/Abstract])) OR (jiawei-xiaoyao-san[Title/Abstract])) OR (jia-wei-xiao-yao-san[Title/Abstract])) OR (kamisyoyo-san[Title/Abstract]) | 258 |
| #3 | #1 AND #2 | 0 |
| **The search strategy for Web of science** | | |
| **Sequence** | **Search terms** | **Hits** |
| #1 | TS=(Hashimoto Disease) | 3,506 |
| #2 | **(((((((((((((((((((((AB=(Disease, Hashimoto)) OR AB=(Hashimoto Struma)) OR AB=(Hashimoto Thyroiditis)) OR AB=(Hashimoto Thyroiditides)) OR AB=(Thyroiditides, Hashimoto)) OR AB=(Thyroiditis, Hashimoto)) OR AB=(Hashimoto's Syndrome)) OR AB=(Hashimoto Syndrome)) OR AB=(Hashimoto's Syndromes)) OR AB=(Hashimotos Syndrome)) OR AB=(Syndrome, Hashimoto's)) OR AB=(Syndromes, Hashimoto's)) OR AB=(Hashimoto's Struma)) OR AB=(Chronic Lymphocytic Thyroiditis)) OR AB=(Chronic Lymphocytic Thyroiditides)) OR AB=(Lymphocytic Thyroiditides, Chronic)) OR AB=(Lymphocytic Thyroiditis, Chronic)) OR AB=(Thyroiditides, Chronic Lymphocytic)) OR AB=(Thyroiditis, Chronic Lymphocytic)) OR AB=(Hashimoto's Disease)) OR AB=(Disease, Hashimoto's)) OR AB=(Hashimotos Disease)** | 4,035 |
| #3 | #1 OR #2 | 4,535 |
| #4 | **((TS=(xiaoyao)) OR TS=(danzhi xiaoyao)) OR TS=(kamisyoyo san)** | 130 |
| #5 | **((((((((AB=(XYDN)) OR AB=(xiao-yao)) OR AB=(kami-shoyo-san)) OR AB=(kami-soyo-san)) OR AB=(TJ-24)) OR AB=(TJ24)) OR AB=(jiawei-xiaoyao-san)) OR AB=(jia-wei-xiao-yao-san)) OR AB=(kamisyoyo-san)** | 121 |
| #6 | #4 OR #5 | 248 |
| #7 | #3 AND #6 | 0 |
| **The search strategy for Cochrane Library** | | |
| **Sequence** | **Search terms** | **Hits** |
| #1 | MeSH descriptor:[Hashimoto Disease] explode all trees | 101 |
| #2 | (Disease, Hashimoto or Hashimoto Struma or Hashimoto Thyroiditis or Hashimoto Thyroiditides or Thyroiditides, Hashimoto or Thyroiditis, Hashimoto or Hashimoto's Syndrome or Hashimoto Syndrome or Hashimoto's Syndromes or Hashimotos Syndrome or Syndrome, Hashimoto's or Syndromes, Hashimoto's or Hashimoto's Struma or Chronic Lymphocytic Thyroiditis or Chronic Lymphocytic Thyroiditides or Lymphocytic Thyroiditides, Chronic or Lymphocytic Thyroiditis, Chronic or Thyroiditides, Chronic Lymphocytic or Thyroiditis, Chronic Lymphocytic or Hashimoto's Disease or Disease, Hashimoto's or Hashimotos Disease):ti,ab,kw | 322 |
| #3 | #1 OR #2 | 322 |
| #4 | MeSH descriptor:[Xiaoyao] explode all trees | 0 |
| #5 | xiaoyao or danzhi xiaoyao or kamisyoyo san or XYDN or xiao-yao or kami-shoyo-san or kami-soyo-san or TJ-24 or TJ24 or jiawei-xiaoyao-san or jia-wei-xiao-yao-san or kamisyoyo-san):ti,ab,kw | 135 |
| #6 | #4 OR #5 | 135 |
| #7 | #3 AND #6 | 0 |
| **The search strategy for EMBASE** | | |
| **Sequence** | **Search terms** | **Hits** |
| #1 | 'hashimoto disease'/exp | 16,220 |
| #2 | ‘Disease, Hashimoto’:ab,ti OR ‘Hashimoto Struma’:ab,ti OR ‘Hashimoto Thyroiditis’:ab,ti OR ‘Hashimoto Thyroiditides’:ab,ti OR ‘Thyroiditides, Hashimoto’:ab,ti OR ‘Thyroiditis, Hashimoto’:ab,ti OR ‘Hashimoto Syndrome’:ab,ti OR ‘Hashimotos Syndrome’:ab,ti OR ‘Hashimotos Struma’:ab,ti OR ‘Chronic Lymphocytic Thyroiditis’:ab,ti OR ‘Chronic Lymphocytic Thyroiditides’:ab,ti OR ‘Lymphocytic Thyroiditides, Chronic’:ab,ti OR ‘Lymphocytic Thyroiditis, Chronic’:ab,ti OR ‘Thyroiditides, Chronic Lymphocytic’:ab,ti OR ‘Thyroiditis, Chronic Lymphocytic’:ab,ti OR ‘Hashimotos Disease’:ab,ti | 2,968 |
| #3 | #1 OR #2 | 16,787 |
| #4 | 'xiaoyao'/exp OR 'xiaoyao san'/exp OR 'xiaoyaosan'/exp OR 'xiaoyao powder'/exp OR 'xiaoyao pill'/exp OR 'danzhi xiaoyao' OR 'kamisyoyo san' | 273 |
| #5 | 'XYDN':ab,ti OR 'xiao-yao':ab,ti OR 'kami-shoyo-san':ab,ti OR 'kami-soyo-san':ab,ti OR 'TJ-24':ab,ti OR 'TJ24':ab,ti OR 'jiawei-xiaoyao-san':ab,ti OR 'jia-wei-xiao-yao-san':ab,ti OR 'kamisyoyo-san':ab,ti | 161 |
| #6 | #4 OR #5 | 431 |
| #7 | #3 AND #6 | 0 |
| **The search strategy for CWKI** | | |
| **Sequence** | **Search terms** | **Hits** |
| #1 | SU%=桥本 or 桥本氏病 or Hashimotos病 or Hashimoto综合征 or 桥本综合征 or Hashimotos综合征 or 桥本甲状腺炎 or 桥本甲状腺肿 or 慢性淋巴细胞性甲状腺炎 | 8,278 |
| #2 | SU%=逍遥 or 逍遥丸 or 逍遥片 or 逍遥散 or 逍遥合剂 or 逍遥胶囊 or 逍遥颗粒 or 黑逍遥汤 or 黑逍遥散 or 逍遥软胶囊 or 加味逍遥散 or 八味逍遥散 or 加味逍遥汤 or 加味逍遥丸 or 加味逍遥片 or 丹栀逍遥丸 or 丹栀逍遥片 or 丹栀逍遥汤 or 丹栀逍遥散 or 逍遥丸水丸 or 加味逍遥颗粒 | 12,564 |
| #3 | #1 AND #2 | 27 |
| **The search strategy for Wanfang Data** | | |
| **Sequence** | **Search terms** | **Hits** |
| #1 | Theme=桥本 or 桥本氏病 or Hashimotos病 or Hashimoto综合征 or 桥本综合征 or Hashimotos综合征 or 桥本甲状腺炎 or 桥本甲状腺肿 or 慢性淋巴细胞性甲状腺炎 | 9,490 |
| #2 | Theme=逍遥 or 逍遥丸 or 逍遥片 or 逍遥散 or 逍遥合剂 or 逍遥胶囊 or 逍遥颗粒 or 黑逍遥汤 or 黑逍遥散 or 逍遥软胶囊 or 加味逍遥散 or 八味逍遥散 or 加味逍遥汤 or 加味逍遥丸 or 加味逍遥片 or 丹栀逍遥丸 or 丹栀逍遥片 or 丹栀逍遥汤 or 丹栀逍遥散 or 逍遥丸水丸 or 加味逍遥颗粒 | 20,252 |
| #3 | #1 AND #2 | 31 |
| **The search strategy for SinoMed** | | |
| **Sequence** | **Search terms** | **Hits** |
| #1 | "桥本"[常用字段:智能] OR "桥本氏病"[常用字段:智能] OR "hashimotos病"[常用字段:智能] OR "hashimoto综合征"[常用字段:智能] OR "桥本综合征"[常用字段:智能] OR "hashimotos综合征"[常用字段:智能] OR "桥本甲状腺炎"[常用字段:智能] OR "桥本甲状腺肿"[常用字段:智能] OR "慢性淋巴细胞性甲状腺炎"[常用字段:智能] | 6,232 |
| #2 | "逍遥"[常用字段:智能] OR "逍遥丸"[常用字段:智能] OR "逍遥片"[常用字段:智能] OR "逍遥散"[常用字段:智能] OR "逍遥合剂"[常用字段:智能] OR "逍遥胶囊"[常用字段:智能] OR "逍遥颗粒"[常用字段:智能] OR "黑逍遥汤"[常用字段:智能] OR "逍遥软胶囊"[常用字段:智能] OR "加味逍遥散"[常用字段:智能] OR "八味逍遥散"[常用字段:智能] OR "加味逍遥丸"[常用字段:智能] OR "加味逍遥片"[常用字段:智能] OR "丹栀逍遥丸"[常用字段:智能] OR "丹栀逍遥片"[常用字段:智能] OR "丹栀逍遥散"[常用字段:智能] OR "逍遥丸水丸"[常用字段:智能] OR "加味逍遥颗粒"[常用字段:智能] | 7,376 |
| #3 | #1 AND #2 | 24 |
| The common fields consist of four search terms: Chinese title, abstract, keywords, and theme words. | | |
| **The search strategy for VIP** | | |
| **Sequence** | **Search terms** | **Hits** |
| #1 | M=桥本+桥本氏病 + Hashimotos病 + Hashimoto综合征 + 桥本综合征 + Hashimotos综合征 + 桥本甲状腺炎 + 桥本甲状腺肿 + 慢性淋巴细胞性甲状腺炎 | 4,287 |
| #2 | M=逍遥 + 逍遥丸 + 逍遥片 + 逍遥散 + 逍遥合剂 + 逍遥胶囊 + 逍遥颗粒 + 黑逍遥汤 + 黑逍遥散 + 逍遥软胶囊 + 加味逍遥散 + 八味逍遥散 + 加味逍遥汤 + 加味逍遥丸 + 加味逍遥片 + 丹栀逍遥丸 + 丹栀逍遥片 + 丹栀逍遥汤 + 丹栀逍遥散 + 逍遥丸水丸 + 加味逍遥颗粒 | 8,572 |
| #3 | #1 AND #2 | 15 |

# Table S3 Specific review annotations for the 29 deleted articles after duplication.

| Reviewed Papers（29） | Review Annotations | Reviewer |
| --- | --- | --- |
| 1. Professor Chen Jun 's experience in the treatment of modified Danzhi Xiaoyao Powder | Non-RCT | CW/WM |
| 1. Based on ' preventive treatment of disease ', the mechanism of Xiaoyaosan Jiawei Granules on Hashimoto 's thyroiditis was discussed | Non-RCT | CW/WM |
| 1. Cui Yingmin 's experience in treating gynecological diseases with modified Xiaoyaosan | Non-RCT | CW/WM |
| 1. To explore the mechanism of Jiawei Xiaoyao Powder in the treatment of Hashimoto 's thyroiditis with qi stagnation and phlegm obstruction based on network pharmacology. | Network pharmacology | CW/WM |
| 1. Zhang Lan 's experience in treating Hashimoto 's thyroiditis with ointment | Non-RCT | CW/WM |
| 1. Study on the correlation between the curative effect of Chinese and western medicine and glucocorticoid receptor in rats with autoimmune thyroiditis | Animal experiments | CW/WM |
| 1. Clinical Summary of Treatment of Hashimoto 's Thyroiditis from Spleen | Non-RCT | CW/WM |
| 1. The mechanism of Xiaoyao Bushen Recipe in the treatment of Hashimoto 's thyroiditis based on network pharmacology and metabolomics | Network pharmacology | CW/WM |
| 1. Clinical Experience of Sun Tongjiao Using Xiaoyao Powder | Non-RCT | CW/WM |
| 1. Professor Zhang Lan 's experience in treating Hashimoto 's thyroiditis from phlegm | Non-RCT | CW/WM |
| 1. WANG Jianhua 's experience in treating gall disease from liver | Non-RCT | CW/WM |
| 1. Analysis of TCM Syndrome Distribution and Medication Rules of Hashimoto 's Thyroiditis | Retrospective study | CW/WM |
| 1. Professor Wang Dong 's experience in treating Hashimoto 's thyroiditis from liver, spleen and kidney | Non-RCT | CW/WM |
| 1. Discussion on the treatment of benign thyroid nodules by Xiaoyao Powder from ' powder ' to ' pill ' | Non-RCT | CW/WM |
| 1. Analysis of Professor Liu Surong 's Thoughts on Treating Hashimoto 's Thyroiditis | Non-RCT | CW/WM |
| 1. Guo Junjie 's experience in treating Hashimoto 's thyroiditis with hypothyroidism | Non-RCT | CW/WM |
| 1. Application of Chinese patent medicine in Hashimoto 's thyroiditis | Reviews | CW/WM |
| 1. Clinical Observation of Xiaochaihu Granules Combined with Xiaoyao Pills in the Treatment of Hashimoto 's Disease | Not reporting relevant outcomes | CW/LW |
| 1. Clinical Observation of Yupingfeng Granules Combined with Xiaoyao Pills in the Treatment of Hashimoto 's Thyroiditis | Not reporting relevant outcomes | CW/LW |
| 1. Clinical effect of modified Xiaoyao Powder combined with external application of traditional Chinese medicine collapse stains on hashimoto thyroiditis | Inappropriate intervention | CW/LW |
| 1. Regulating effect of Xiaoyao Powder combined with Euthyrox on serum index levels in patients with hypothyroidism caused by Hashimoto 's thyroiditis | Inappropriate intervention | CW/LW |
| 1. Clinical Observation of Xiaoyaosan in the Treatment of Hashimoto 's Hypothyroidism | Inappropriate intervention | CW/LW |
| 1. Clinical Observation of Xiaoyaosan Combined with Euthyrox in the Treatment of Hashimoto 's Hypothyroidism | Inappropriate intervention | CW/LW |
| 1. Efficacy observation of Jiawei Xiaoyaosan in the treatment of Hashimoto 's thyroiditis with liver depression and spleen deficiency syndrome and its effect on IFN-γ, IL-2,6,10 | The composition of the intervention medication was unclear | CW/LQ |
| 1. Clinical Observation on Modified Xiaoyao Powder in the Treatment of 76 Cases of Chronic Lymphocytic Thyroiditis | The composition of the intervention medication was unclear | CW/LQ |
| 1. Clinical Observation on Modified Xiaoyao Powder in the Treatment of 76 Cases of Chronic Lymphocytic Thyroiditis | Inappropriate intervention | CW/LQ |
| 1. Clinical intervention and research of Danzhi Xiaoyao Powder on liver depression and spleen deficiency with heat syndrome of Hashimoto 's hyperthyroidism | Inappropriate intervention | CW/LQ |
| 1. Clinical study on Xiakucao Granules combined with Danzhi Xiaoyao Powder in treatment of Hashimoto 's thyroiditis under different thyroid function states | Reports not retrieved | CW/LQ |
| 1. Clinical observation of Xiaoyao powder Jiawei granule in the treatment of early Hashimoto's thyroiditis | The principle of randomization was not specified | CW/WM |

# Table S4 Summary of Botanical Drugs Information

| **Non-scientific names (Chinese name)** | **Class of name** | **Part(s) of plant used** | **Name as published** | **Medicinal source** |
| --- | --- | --- | --- | --- |
| Carthami flos (Honghua) | Pharmaceutical | Flower | *Carthamus tinctorius* L. | [Pharmacop. of China (2015)](https://mpns.science.kew.org/mpns-portal/reference?reference=100050&query=Paeonia+Lactiflora&filter=&fuzzy=false&nameType=all)  [WHODrug Herbal Substances 2024 (WHO-UMC, 2024)](https://mpns.science.kew.org/mpns-portal/reference?reference=100469&query=Paeonia+Lactiflora&filter=&fuzzy=false&nameType=all) |
| Gleditsiae spina (Zaojiaoci) | Pharmaceutical | Fruit | *Gleditsia sinensis* Lam. | [Pharmacop. of China (2015)](https://mpns.science.kew.org/mpns-portal/reference?reference=100050&query=Paeonia+Lactiflora&filter=&fuzzy=false&nameType=all)  [WHODrug Herbal Substances 2024 (WHO-UMC, 2024)](https://mpns.science.kew.org/mpns-portal/reference?reference=100469&query=Paeonia+Lactiflora&filter=&fuzzy=false&nameType=all) |
| Moutan cortex (Mudanpi) | Pharmaceutical | Root bark | *Paeonia suffruticosa* Andr. | [Pharmacop. of China (2015)](https://mpns.science.kew.org/mpns-portal/reference?reference=100050&query=Paeonia+Lactiflora&filter=&fuzzy=false&nameType=all)  [WHODrug Herbal Substances 2024 (WHO-UMC, 2024)](https://mpns.science.kew.org/mpns-portal/reference?reference=100469&query=Paeonia+Lactiflora&filter=&fuzzy=false&nameType=all) |
| Gardeniae fructus (Zhizi) | Pharmaceutical | Fruit | *Gardenia jasminoides* Ellis. | [Pharmacop. of China (2015)](https://mpns.science.kew.org/mpns-portal/reference?reference=100050&query=Paeonia+Lactiflora&filter=&fuzzy=false&nameType=all)  [WHODrug Herbal Substances 2024 (WHO-UMC, 2024)](https://mpns.science.kew.org/mpns-portal/reference?reference=100469&query=Paeonia+Lactiflora&filter=&fuzzy=false&nameType=all) |
| Astragali radix (Huangqi) | Pharmaceutical | Root | *Astragalus membranaceus (Fisch.)* Bge. | [Pharmacop. of China (2015)](https://mpns.science.kew.org/mpns-portal/reference?reference=100050&query=Paeonia+Lactiflora&filter=&fuzzy=false&nameType=all)  [WHODrug Herbal Substances 2024 (WHO-UMC, 2024)](https://mpns.science.kew.org/mpns-portal/reference?reference=100469&query=Paeonia+Lactiflora&filter=&fuzzy=false&nameType=all) |
| Curcumae radix (Yujin) | Pharmaceutical | Root tuber | *Curcuma wenyujin* Y. H. Chen et C. Ling | [Pharmacop. of China (2015)](https://mpns.science.kew.org/mpns-portal/reference?reference=100050&query=Paeonia+Lactiflora&filter=&fuzzy=false&nameType=all)  [WHODrug Herbal Substances 2024 (WHO-UMC, 2024)](https://mpns.science.kew.org/mpns-portal/reference?reference=100469&query=Paeonia+Lactiflora&filter=&fuzzy=false&nameType=all) |
| Cistanches herba (Roucongrong) | Pharmaceutical | Stem | *Cistanche deserticola* Y. C. Ma | [Pharmacop. of China (2015)](https://mpns.science.kew.org/mpns-portal/reference?reference=100050&query=Paeonia+Lactiflora&filter=&fuzzy=false&nameType=all)  [WHODrug Herbal Substances 2024 (WHO-UMC, 2024)](https://mpns.science.kew.org/mpns-portal/reference?reference=100469&query=Paeonia+Lactiflora&filter=&fuzzy=false&nameType=all) |
| Ligustri lucidi fructus (Nuzhenzi) | Pharmaceutical | Fruit | *Ligustrum lucidum* Ait. | [Pharmacop. of China (2015)](https://mpns.science.kew.org/mpns-portal/reference?reference=100050&query=Paeonia+Lactiflora&filter=&fuzzy=false&nameType=all)  [WHODrug Herbal Substances 2024 (WHO-UMC, 2024)](https://mpns.science.kew.org/mpns-portal/reference?reference=100469&query=Paeonia+Lactiflora&filter=&fuzzy=false&nameType=all) |
| Chuanxiong rhizome (Chuanxiong) | Pharmaceutical | rhizome | *Ligusticum chuanxiong* Hort. | [Pharmacop. of China (2015)](https://mpns.science.kew.org/mpns-portal/reference?reference=100050&query=Paeonia+Lactiflora&filter=&fuzzy=false&nameType=all)  [WHODrug Herbal Substances 2024 (WHO-UMC, 2024)](https://mpns.science.kew.org/mpns-portal/reference?reference=100469&query=Paeonia+Lactiflora&filter=&fuzzy=false&nameType=all) |
| Pseudostellariae radix (Taizishen) | Pharmaceutical | root tuber | *Pseudostellaria heterophylla (Miq.)* Pax ex Pax et Hoffm. | [Pharmacop. of China (2015)](https://mpns.science.kew.org/mpns-portal/reference?reference=100050&query=Paeonia+Lactiflora&filter=&fuzzy=false&nameType=all)  [WHODrug Herbal Substances 2024 (WHO-UMC, 2024)](https://mpns.science.kew.org/mpns-portal/reference?reference=100469&query=Paeonia+Lactiflora&filter=&fuzzy=false&nameType=all) |
| Sargassum (Haizao) | Other | Dried algal body | *Sargassum pallidum（Turn.）*C.Ag  *Sargassum*C.Agardh | [Pharmacop. of China (2015)](https://mpns.science.kew.org/mpns-portal/reference?reference=100050&query=Paeonia+Lactiflora&filter=&fuzzy=false&nameType=all)  [WHODrug Herbal Substances 2024 (WHO-UMC, 2024)](https://mpns.science.kew.org/mpns-portal/reference?reference=100469&query=Paeonia+Lactiflora&filter=&fuzzy=false&nameType=all)  <https://www.gbif.org/> |
| Laminaria japonica (Kunbu) | Other | Dried thallus | *Laminaria japonica* Aresch.  *Laminaria japonica*J.E.Areschoug | [Pharmacop. of China (2015)](https://mpns.science.kew.org/mpns-portal/reference?reference=100050&query=Paeonia+Lactiflora&filter=&fuzzy=false&nameType=all)  [WHODrug Herbal Substances 2024 (WHO-UMC, 2024)](https://mpns.science.kew.org/mpns-portal/reference?reference=100469&query=Paeonia+Lactiflora&filter=&fuzzy=false&nameType=all)  <https://www.gbif.org/> |
| Dioscoreae rhizome (Shanyao) | Pharmaceutical | rhizome | *Dioscorea opposita* Thunb. | [Pharmacop. of China (2015)](https://mpns.science.kew.org/mpns-portal/reference?reference=100050&query=Paeonia+Lactiflora&filter=&fuzzy=false&nameType=all)  [WHODrug Herbal Substances 2024 (WHO-UMC, 2024)](https://mpns.science.kew.org/mpns-portal/reference?reference=100469&query=Paeonia+Lactiflora&filter=&fuzzy=false&nameType=all) |
| Rehmanniae radix (Shudihuang) | Pharmaceutical | root tuber | *Rehmannia glutinosa* Libosch. | [Pharmacop. of China (2015)](https://mpns.science.kew.org/mpns-portal/reference?reference=100050&query=Paeonia+Lactiflora&filter=&fuzzy=false&nameType=all)  [WHODrug Herbal Substances 2024 (WHO-UMC, 2024)](https://mpns.science.kew.org/mpns-portal/reference?reference=100469&query=Paeonia+Lactiflora&filter=&fuzzy=false&nameType=all) |
| Corni fructus (Shanzhuyu) | Pharmaceutical | sarcocarp | *Cornus officinalis* Sieb. et Zucc. | [Pharmacop. of China (2015)](https://mpns.science.kew.org/mpns-portal/reference?reference=100050&query=Paeonia+Lactiflora&filter=&fuzzy=false&nameType=all)  [WHODrug Herbal Substances 2024 (WHO-UMC, 2024)](https://mpns.science.kew.org/mpns-portal/reference?reference=100469&query=Paeonia+Lactiflora&filter=&fuzzy=false&nameType=all) |
| Citri reticulatae pericarpium (Chenpi) | Pharmaceutical | pericarp | *Citrus reticulata Blanco* | [Pharmacop. of China (2015)](https://mpns.science.kew.org/mpns-portal/reference?reference=100050&query=Paeonia+Lactiflora&filter=&fuzzy=false&nameType=all)  [WHODrug Herbal Substances 2024 (WHO-UMC, 2024)](https://mpns.science.kew.org/mpns-portal/reference?reference=100469&query=Paeonia+Lactiflora&filter=&fuzzy=false&nameType=all) |
| Pinelliae rhizome (Banxia) | Pharmaceutical | tuber | *Pinellia ternata (Thunb.)* Breit. | [Pharmacop. of China (2015)](https://mpns.science.kew.org/mpns-portal/reference?reference=100050&query=Paeonia+Lactiflora&filter=&fuzzy=false&nameType=all)  [WHODrug Herbal Substances 2024 (WHO-UMC, 2024)](https://mpns.science.kew.org/mpns-portal/reference?reference=100469&query=Paeonia+Lactiflora&filter=&fuzzy=false&nameType=all) |
| Citri fructus (Xiangyuan) | Pharmaceutical | Fruit | *Citrus medica* L. | [Pharmacop. of China (2015)](https://mpns.science.kew.org/mpns-portal/reference?reference=100050&query=Paeonia+Lactiflora&filter=&fuzzy=false&nameType=all)  [WHODrug Herbal Substances 2024 (WHO-UMC, 2024)](https://mpns.science.kew.org/mpns-portal/reference?reference=100469&query=Paeonia+Lactiflora&filter=&fuzzy=false&nameType=all) |
| Citri sarcodactylis fructus (Foshou) | Pharmaceutical | Fruit | *Citrus medica L. var. sarcodactylis Swingle* | [Pharmacop. of China (2015)](https://mpns.science.kew.org/mpns-portal/reference?reference=100050&query=Paeonia+Lactiflora&filter=&fuzzy=false&nameType=all)  [WHODrug Herbal Substances 2024 (WHO-UMC, 2024)](https://mpns.science.kew.org/mpns-portal/reference?reference=100469&query=Paeonia+Lactiflora&filter=&fuzzy=false&nameType=all) |
| Prunellae spica (Xiakucao) | Pharmaceutical | Dried fruitspike | *Prunella vulgaris* L. | [Pharmacop. of China (2015)](https://mpns.science.kew.org/mpns-portal/reference?reference=100050&query=Paeonia+Lactiflora&filter=&fuzzy=false&nameType=all)  [WHODrug Herbal Substances 2024 (WHO-UMC, 2024)](https://mpns.science.kew.org/mpns-portal/reference?reference=100469&query=Paeonia+Lactiflora&filter=&fuzzy=false&nameType=all) |
| Cordyceps sinensis (Dongchongxiacao)  Ophiocordyceps sinensis | Other | Dried complex | *Cordyceps sinensis（BerK.）*Sacc. | [Pharmacop. of China (2015)](https://mpns.science.kew.org/mpns-portal/reference?reference=100050&query=Paeonia+Lactiflora&filter=&fuzzy=false&nameType=all)  [WHODrug Herbal Substances 2024 (WHO-UMC, 2024)](https://mpns.science.kew.org/mpns-portal/reference?reference=100469&query=Paeonia+Lactiflora&filter=&fuzzy=false&nameType=all)  <https://www.gbif.org/> |

# Table S5 Details of the Chinese polyherbal preparation used in the included studies

| **Preparation** | **Dosage Form** | **Specifica-tion** | **Approval Number** | **Manufacturer** | **Dose** | **Adverse Reactions** | **Contraindication** |
| --- | --- | --- | --- | --- | --- | --- | --- |
| Xiaoyao Pill | Pill | 0.2g | National medicine permission number Z43020469 | Jiu Zhitang Co. LTD | 9g/time; 2 times/day | Changes of menstrual cycle or menstrual volume in some patients. | Prohibited for those who are allergic to this product. People with allergic constitutions should use it with caution. |
| Guiqi Xiaoyao Mixture | Mixture | 200g | - | Pharmacy of Anhui Provincial Hospital of Traditional Chinese Medicine | 200g/time; 2 times/day | Unclear | Unclear |
| Xiakucao Gao | Soft extract | 9g | National medicine permission number Z20050593 | Shandong Xianhe Pharmaceutical Co. LTD | 9g/time; 2 times/day | Unclear | Pregnant women should avoid taking these medications. |
| Danzhi Xiaoyao Granules | Granules | 11.1g | - | Beijing Kang Ren Tang Pharmaceutical Co. LTD | 11.1g/time; 2 times/day | Unclear | Unclear |
| Xiaoyao San Jiawei Granules | Granules | 15g | - | Jiangyin Tianjiang Pharmaceutical Co. LTD | 30g/time; 3 times/day | Unclear | Unclear |
| Honghua Xiaoyao Tablet | Tablet | 0.39g | National medicine permission number Z20080299 | Jiangxi Puzheng Pharmaceutical Co. LTD | 0.78-1.56g/time; 3 times/ day | Some patients have nausea, abdominal pain, vomiting, dizziness, rash, pruritus, etc. | Prohibited for Pregnant women and those who are allergic to this product. |
| Jinshuibao Tablet | Tablet | 0.42g | National medicine permission number Z20163112 | Jiangxi Jimin Xinke Pharmaceutical Co., Ltd. | 1.68g/times; 3 times/day | Unclear | Not suitable for patients with cold and fever; Patients with severe chronic diseases such as hypertension, heart disease, liver disease, diabetes, and kidney disease should take it under the guidance of a physician; Children, pregnant women, and breastfeeding women should take it under the guidance of a physician; This product is contraindicated for those who are allergic to it and should be used with caution by those with allergic constitutions; Do not use if the physical appearance of the product changes. |

# Table S6 Composition and standardization information of *Xiao-yao San* preparations

| **Preparation** | **Ingredients^1^** | **Approval Number^2^** | **Preparation methods^3^** | **Standardized indicators^4^** | **Quality control^5^** |
| --- | --- | --- | --- | --- | --- |
| Xiaoyao Pill  (XY) | Chaihu 100g, Danggui 100g, Shaoyao 100g, Baizhu 100g, Gancao 80g, Bohe 20g, Shengjiang 100g | National medicine permission number Z43020469 | Soxhlet extraction; HWE; Precipitation; Filtration; Direct powder mixing; Preparation of Pills (General Rules 0108) | TLC: Chaihu, Shaoyao, Fuling, Danggui, Baizhu;  HPLC: Shaoyao (Ch.P 2020) | TLC (General Rules 0502); HPLC (General Rule 0512) |
| Guiqi Xiaoyao Mixture  (GQXY) | Dangui 20g, Huangqi 20g, Chaihu 15g, Fuling 15g, Baizhu 15g, Shaoyao 15g, Yujin 15g, Roucongrong 15g, Nuzhenzi 15g, Chuanxiong 10g | - | HWE; Preparation of Mixtures (General Rules 0181) | Unclear | Unclear |
| Danzhi Xiaoyao Granules  (DZXY)^6^ | Mudanpi 15g, Zhizi 15g, Shaoyao 15g, Danggui 15g, Chaihu 9g, Fuling 15g, Baizhu 15g, Bohe 6g, Gancao 6g | Market Authorization Record (NMPA) | HWE; Precipitation; Drying; Preparation of Granules (General Rules 0104) | Zhizi, Shaoyao, Danggui, Chaihu, Baizhu, Bohe, Gancao (CPC); Mudanpi, Fuling (BJMMPA) | HPLC (General Rule 0512) |
| Xiaoyao San Jiawei Granules  (XYSJW)^6^ | Danggui 10g, Shaoyao 15g, Chaihu 6g, Fuling 10g, Thaizishen 15g, Baizhu 6g, Haizao 8g, Kunbu 8g, Shanzhuyu 10g, Shanyao 10g, Shudihuang 20g, Chenpi 8g, Banxia 6g, Xiangyuan 6g, Foshou 6g | Market Authorization Record (NMPA) | HWE; Precipitation; Drying; Preparation of Granules (General Rules 0104) | Danggui, Shaoyao, Chaihu, Baizhu, Taizishen, Shanzhuyu, Shudihuang, Chenpi, Xiangyuan, Foshou (CPC); Fuling, Haizao, Kunbu, Shanyao, Banxia (JSMPA) | HPLC (General Rule 0512) |
| Honghua Xiaoyao Tablet  (HHXY) | Chaihu 260g, Danggui 260g, Shaoyao 260g, Baizhu 260g, Fuling 260g, Bohe 40g, Gancao 195g, Honghua 50g, Zaojiaoci 80g | National medicine permission number Z20080299 | Soxhlet extraction; UAE; Preparation of Tablets (General Rules 0101) | TLC: Chaihu, Baizhu, Honghua;  HPLC: Shaoyao (Ch.P 2020) | TLC (General Rules 0502); HPLC (General Rule 0512) |

**NMPA**, National Medical Products Administration; **Ch.P 2020**, Pharmacopoeia of the People's Republic of China 2020; **CPC**, Chinese Pharmacopoeia Commission; **BJMMPA**, Beijing Municipal Medical Products Administration; **JSMPA**, Jiangsu Medical Products Administration; **HWE**, Hot water extraction; **UAE**, ultrasound-assisted extraction; **TLC**, Thin layer chromatography; **HPLC**, Performance Liquid Chromatography; 1. The Chinese names of herbal medicines are used to present the table content concisely. For specific plant name comparisons and calculations of patients' daily intake, refer to **Table S9**; 2. The NMPA approval numbers for traditional Chinese medicines and the listing numbers for herbal medicinal granules are obtained from the NMPA website, which can be accessed at https://www.nmpa.gov.cn/datasearch/home-index.html#category=yp for relevant information; 3. The general preparation methods are derived from the Ch.P 2020. Detailed content can be found in **Table S7** and **Supplementary Material 2**; 4. Standardization indicators are sourced from the Ch.P 2020 and the CPC. Detailed indicators are provided in **Table S8** and **Supplementary Material 2**; 5. Quality control methods are based on the Ch.P 2020 and the guidelines of the CPC. Detailed methodologies are outlined in **Table S8** and **Supplementary Material 2**; 6. DZXY and XYSJW adhere to the quality standards for herbal medicinal granules. For detailed information, refer to **Table S10** and **Supplementary Material 2**.

# Table S7 Ingredients and Preparation methods of Chinese polyherbal preparation^*^

| Preparation | Ingredients | Preparation methods |
| --- | --- | --- |
| Xiaoyao Pill | Chaihu 100g, Danggui 100g, Shaoyao 100g, Baizhu 100g, Gancao 80g, Bohe 20g, Shengjiang 100g | The above seven herbs are processed as follows: 100g of Chaihu, 50g of Danggui, 20g of Boheand, 100g of Shengjiang are used to extract volatile oil. The residue is then boiled twice with Baizhu and Fuling for 2 hours each time. The decoctions are combined, filtered, and the filtrate is concentrated into a thick paste. Shaoyao and the remaining Danggui are pulverized into fine powder. 20g of Gancao is also pulverized into fine powder, while the remaining Gancao is boiled three times for 2 hours each. The decoctions are combined, filtered, and either used immediately or left overnight. The filtrate or supernatant is concentrated to an appropriate volume, mixed evenly with the aforementioned thick paste, fine powder, volatile oil, and an appropriate amount of malt sugar. The mixture is then shaped into pills, dried, and polished to obtain the final product. |
| Guiqi Xiaoyao Mixture | Dangui 20g, Huangqi 20g, Chaihu 15g, Fuling 15g, Baizhu 15g, Shaoyao 15g, Yujin 15g, Roucongrong 15g, Nuzhenzi 15g, Chuanxiong 10g | It should comply with the relevant provisions under the mixture item (General Rule 0181). |
| Xiakucao Gao | Xiakucao 2500g | Take Xiakucao, boil it in water three times, each time for 2 hours, combine the decoctions, filter, and concentrate the filtrate into a clear paste with a relative density of 1.21-1.25 (80-85℃). Add 200g of refined honey or 200g of sucrose to every 100g of clear paste, heat to dissolve, mix well, concentrate, and make 1000g of the final product. |
| Danzhi Xiaoyao Granules | Mudanpi 15g, Zhizi 15g, Shaoyao 15g, Danggui 15g, Chaihu 9g, Fuling 15g, Baizhu 15g, Bohe 6g, Gancao 6g | It should comply with the relevant provisions under the granules item (General Rule 0104). In accordance with the standards for formula granule medications (CPC; BJMMPA). |
| Xiaoyao San Jiawei Granules | Danggui 10g, Shaoyao 15g, Chaihu 6g, Fuling 10g, Thaizishen 15g, Baizhu 6g, Haizao 8g,Kunbu 8g, Shanzhuyu 10g, Shanyao 10g, Shudihuang 20g, Chenpi 8g, Banxia 6g, Xiangyuan 6g, Foshou 6g | It should comply with the relevant provisions under the granules item (General Rule 0104). In accordance with the standards for formula granule medications (CPC; JSMPA). |
| Honghua Xiaoyao Tablet | Chaihu 260g, Danggui 260g, Shaoyao 260g, Baizhu 260g, Fuling 260g, Bohe 40g, Gancao 195g, Honghua 50g, Zaojiaoci 80g | 1. First, crush the three herbal ingredients: Danggui, Baizhu, and Bohe them in water for 1.5-3 hours, then use a volatile oil distiller for water distillation until the volatile oil in the oil-water separator no longer increases. 2. Take the drug residue after extracting volatile oil, and extract it together with Shaoyao, Fuling, Honghua, Zaojiaoci, Chaihu and Gancao using 8-12 times the amount of water as the solvent. Use an ultrasonic extractor to extract 2-3 times, each time for 30-50 minutes. After filtering, the water extract is concentrated under reduced pressure and dried to obtain a water extract. 3. Under the condition that the mass ratio of β-cyclodextrin to the volatile oil is 0.5-1.5:4-8, use the saturated aqueous solution method, stir and encapsulate at 30°C-50°C for 20-40 minutes, then precipitate at low temperature, filter and dry to obtain the product. The preferred volume ratio of volatile oil to β-cyclodextrin is 1:5, the preferred encapsulation reaction temperature is 40°C, and the preferred encapsulation time is 30 minutes. The volume unit of the volatile oil is milliliters, and the mass unit of β-cyclodextrin is grams. 4. Add starch to the water extract obtained in step 2 and the volatile oil β-cyclodextrin inclusion compound obtained in step 3, granulate and compress into tablets, and coat with a film to obtain the final product. |
| Jinshui Bao Tablet | Fermented *Cordyceps sinensis* Mycelia Powder (Cs-4) 500g | Take Fermented *Cordyceps sinensis* Mycelia Powder (Cs-4), add appropriate excipients, mix well, make into granules, dry, and compress into 1000 tablets. Coat with sugar or film coating to obtain the final product. |

* Using the Chinese names of botanical drugs provides a more concise and accurate representation of the table content. The official names of the herbs can be cross-referenced in the **Table 1 and S4**. For more detailed information on the formula granules, refer to **Table S10** and **Supplementary Material 2**. Further patent details and general rules can be found in **Supplementary Material 2**. **CPC**, Chinese Pharmacopoeia Commission; **BJMMPA**, Beijing Municipal Medical Products Administration; **JSMPA**, Jiangsu Medical Products Administration;

# Table S8 Identification, Inspection and Content Determination of Chinese polyherbal preparation*

| **Preparation** | **Identification** | **Inspection** | **Content Determination** |
| --- | --- | --- | --- |
| Xiaoyao Pill | (1) Take this product and observe it under a microscope: The oil tubes contain yellow or tan secretions, with a diameter of 8 to 25 micrometers (Chaihu). The calcium oxalate cluster crystals have a diameter of 18 to 32 micrometers and exist in parenchyma cells, often arranged in rows, or several cluster crystals may exist in one cell (Shaoyao). The calcium oxalate needle crystals are fine, with a length of 10 to 32 micrometers, irregularly filling the parenchyma cells (Baizhu). The parenchyma cells are spindle-shaped, with slightly thick walls and a very fine oblique interlaced texture on the surface (Danggui). Take this product and observe it under a microscope: The irregular branched mass is colorless and dissolves in chloral hydrate test solution; the hyphae are colorless or light brown, with a diameter of 4 to 6 micrometers (Fuling).  (2) Perform TLC (General Rule 0502) test. In the chromatography of the test product, spots of the same color appear at the corresponding positions in the chromatography of the control medicinal material under sunlight; fluorescent spots of the same color appear under UV light. | It should comply with the relevant provisions under the pill item (General Rule 0108). | Determination is performed according to the HPLC (General Rule 0512).  Chromatographic Conditions and System Suitability Test: Use octadecylsilane chemically bonded silica as the filler, and acetonitrile-0.1% phosphoric acid solution (15:85) as the mobile phase. The detection wavelength is set to 230nm. The theoretical plate number, calculated based on the paeoniflorin peak, should be no less than 2000. Preparation of Reference Solution: Take an appropriate amount of paeoniflorin reference substance, accurately weigh it, and add diluted ethanol to prepare a solution containing 60μg per 1ml. Preparation of Test Solution: Take an appropriate amount of the product, grind it finely, take approximately 0.4g, accurately weigh it, place it in a stoppered conical flask, accurately add 25ml of diluted ethanol, tightly stopper the flask, weigh it, and then subject it to ultrasonic treatment (power 250W, frequency 33kHz) for 30 minutes. Allow it to cool, reweigh it, make up any lost weight with diluted ethanol, shake well, filter, and collect the subsequent filtrate. Determination Method: Precisely pipette 10μl of each of the above two solutions, inject them into the liquid chromatograph, and perform the measurement.  The content of paeoniflorin (C_23_H_28_O_11_) in each 1g of the product should not be less than 4.0mg |
| Guiqi Xiaoyao Mixture | No publicly available data were found | It should comply with the relevant provisions under the mixture item (General Rule 0181). | No publicly available data were found |
| Xiakucao Gao | Take 2g of this product, add 25ml of water to dissolve, adjust the pH to 2-3 with dilute hydrochloric acid, filter, and extract the filtrate three times with ether by shaking, 25ml each time. Combine the ether extracts, evaporate to dryness, dissolve the residue in 1ml of absolute ethanol as the test solution. Separately, take 0.5g of reference medicinal material of Prunella vulgaris, add 25ml of water, boil for 30 minutes, let cool, filter, take the filtrate, and prepare a reference medicinal solution using the same method from "adjust the pH to 2-3 with dilute hydrochloric acid". Conduct TLC (General Rule 0502), pipette 5μl of the above two solutions, spot them separately on the same silica gel G thin-layer plate, use chloroform-methanol-glacial acetic acid-water (7:2:0.5:0.3) as the developing solvent, develop, remove, dry, spray with 2% ferric chloride ethanol solution, and heat until the spots are clearly visible. In the chromatogram of the tested sample, spots of the same color appear at the corresponding positions as in the chromatogram of the reference medicinal materials. | The relative density should be 1.40-1.46 (General Rule 0183).  Others should comply with the relevant regulations under the item of decoction (General Rule 0183). | No publicly available data were found |
| Danzhi Xiaoyao Granules | In accordance with the standards for formula granule medications (CPC; BJMMPA). | It should comply with the relevant provisions under the granules item (General Rule 0104). | In accordance with the standards for formula granule medications (CPC; BJMMPA). |
| Xiaoyao San Jiawei Granules | In accordance with the standards for formula granule medications (CPC; JSMPA). | It should comply with the relevant provisions under the granules item (General Rule 0104). | In accordance with the standards for formula granule medications (CPC; JSMPA). |
| Honghua Xiaoyao Tablet | Perform TLC (General Rule 0502) test. In the chromatography of the test product, spots of the same color appear at the corresponding positions in the chromatography of the control medicinal material under sunlight; fluorescent spots of the same color appear under UV light. (Chaihu, Baizhu, Honghua) | It should comply with the relevant provisions under the tablet item (General Rule 0101). | HPLC: Paeoniflorin (C_23_H_28_O_11_) was used as the reference peak for standard fingerprint determination. |
| Jinshui Bao Tablet | Perform TLC (General Rule 0502) test. In the chromatography of the test product, spots of the same color appear at the corresponding positions in the chromatography of the control medicinal material under sunlight; fluorescent spots of the same color appear under UV light. (ergosterol (C_28_H_44_O), uridine (C_9_H_12_N_2_O_6_), guanosine (C_10_H_13_N_5_O_5_), and adenosine (C_10_H_13_N_5_O_4_)) | It should comply with the relevant provisionss under the tablet section (General Rule 0101). | Determination of Uridine, Guanosine, and Adenosine by HPLC (General Rule 0512).  Chromatographic conditions and system suitability test: Use octadecylsilane chemically bonded silica as the filling agent; use 0.05mol/L potassium dihydrogen phosphate aqueous solution as mobile phase A and methanol as mobile phase B. Perform gradient elution as specified in the table below; the detection wavelength is set at 260nm. The theoretical plate count should be not less than 5000, calculated based on the adenosine peak. Preparation of reference standard solution: Take appropriate amounts of uridine, guanosine, and adenosine reference standards, accurately weigh them, and dissolve in water to prepare a mixed solution containing 30μg of uridine, 20μg of guanosine, and 20μg of adenosine per 1ml. Preparation of test solution: Take 20 tablets of the product, remove the coating, accurately weigh, and grind into a fine powder. Take approximately 0.5g, accurately weigh, place in a stoppered conical flask, accurately add 50ml of 70% methanol, weigh the flask, and subject it to ultrasonic treatment (power 500W, frequency 40kHz) for 20 minutes. Remove, allow to cool, weigh again, make up the lost weight with 70% methanol, mix well, filter, and collect 25ml of the subsequent filtrate into an evaporating dish. Recover the solvent to dryness, dissolve the residue in water, dilute to volume in a 25ml volumetric flask, filter, and collect the subsequent filtrate as the test solution. Assay method: Accurately pipette 20μl each of the reference standard solution and the test solution, inject into the HPLC system, and measure.  The total content of uridine (C_9_H_12_N_2_O_6_), guanosine (C_10_H_13_N_5_O_5_), and adenosine (C_10_H_13_N_5_O_4_) per tablet should not be less than 2.4mg for specification.  Determination of Ergosterol by HPLC (General Rule 0512).  Chromatographic conditions and system suitability test: Use octadecylsilane chemically bonded silica as the filling agent; use methanol-water (98:2) as the mobile phase; the detection wavelength is set at 283nm. The theoretical plate count should be not less than 5000, calculated based on the ergosterol peak. Preparation of reference standard solution: Take an appropriate amount of ergosterol reference standard, accurately weigh, and dissolve in methanol to prepare a solution containing 40μg per 1ml as the reference standard solution. Preparation of test solution: Take 20 tablets of the product, remove the coating, accurately weigh, and grind into a fine powder. Take approximately 0.5g, accurately weigh, place in a stoppered conical flask, accurately add 25ml of methanol, weigh the flask, and subject it to ultrasonic treatment (power 500W, frequency 40kHz) for 1 hour. Remove, allow to cool, weigh again, make up the lost weight with methanol, filter, and collect the subsequent filtrate as the test solution. Assay method: Accurately pipette 10μl each of the reference standard solution and the test solution, inject into the HPLC system, and measure.  The content of ergosterol (C_28_H_44_O) per tablet should not be less than 1.0mg for specification (3). |

* Using the Chinese names of botanical drugs provides a more concise and accurate representation of the table content. The official names of the herbs can be cross-referenced in the **Table1** and **S4**. **CPC**, Chinese Pharmacopoeia Commission; **BJMMPA**, Beijing Municipal Medical Products Administration; **JSMPA**, Jiangsu Medical Products Administration; **TLC**, Thin layer chromatography; **HPLC**, Performance Liquid Chromatography. For more detailed information on the formula granules, refer to **Table S10** and **Supplementary Material 2**. Further patent details and general rules can be found in **Supplementary Material 2**.

# Table S9 Botanical drugs of Xiao-yao San preparations and daily intake measurement*

| **Name as published** | **Nomenclature of TCM** | | **XY** | **HHXY** | | **DZXY** | **GQXY** | **XYSJW** |
| --- | --- | --- | --- | --- | --- | --- | --- | --- |
| *Bupleurum Chinese* DC. | Chaihu | | 10g | 15g | | 9g | 15g | 6g |
| *Paeonia lactiflora* Pall. | Shaoyao | | 10g | 15g | | 15g | 15g | 15g |
| *Atractylodes macrocephala* Koidz. | Baizhu | | 10g | 15g | | 15g | 15g | 6g |
| *Angelica sinensis (Oliv.)* Diels. | Danggui | | 10g | 15g | | 15g | 20g | 10g |
| *Poria cocos (Schw.)* Wolf.  *Poria cocos* F.A.Wolf | Fuling | | 10g | 15g | | 15g | 15g | 10g |
| *Glycyrrhiza glabra* L. | Gancao | | 8g | 12g | | 6g | - | ^-^ |
| *Mentha canadensis* L. | Bohe | | 2g | 2g | | 6g | - | - |
| *Zingiber officinale* Rosc. | Shengjiang | | 10g | - | | - | - | - |
| *Carthamus tinctorius* L. | Honghua | | - | 3g | | - | - | - |
| *Gleditsia sinensis* Lam. | Zaojiaoci | | - | 5g | | - | - | - |
| *Paeonia suffruticosa* Andr. | Mudanpi | | - | - | | 15g | - | - |
| *Gardenia jasminoides* Ellis. | Zhizi | | - | - | | 15g | - | - |
| *Astragalus membranaceus (Fisch.)* Bge. | Huangqi | | - | - | | - | 20g | - |
| *Curcuma wenyujin* Y. H. Chen et C. Ling | Yujin | | - | - | | - | 15g | - |
| *Cistanche deserticola* Y. C. Ma | Rongcongrong | | - | - | | - | 15g | - |
| *Ligustrum lucidum* Ait. | Nuzhenzi | | - | - | | - | 15g | - |
| *Ligusticum chuanxiong* Hort. | Chuanxiong | | - | - | | - | 10g | - |
| *Pseudostellaria heterophylla (Miq.)* Pax ex Pax et Hoffm. | Taizishen | | - | - | | - | - | 15g |
| *Sargassum pallidum（Turn.）*C.Ag  *Sargassum*C.Agardh | Haizao | | - | - | | - | - | 8g |
| *Laminaria japonica* Aresch.  *Laminaria japonica*J.E.Areschoug | Kunbu | - | | | - | - | - | 8g |
| *Dioscorea opposita* Thunb. | Shanyao | - | | | - | - | - | 10g |
| *Rehmannia glutinosa* Libosch. | Shudihuang | - | | | - | - | - | 20g |
| *Cornus officinalis* Sieb. et Zucc. | Shanzhuyu | - | | | - | - | - | 10g |
| *Citrus reticulata Blanco* | Chenpi | - | | | - | - | - | 8g |
| *Pinellia ternata (Thunb.)* Breit. | Banxia | - | | | - | - | - | 6g |
| *Citrus medica* L. | Xiangyuan | - | | | - | - | - | 6g |
| *Citrus medica L. var. sarcodactylis Swingle* | Foshou | - | | | - | - | - | 6g |

*Daily intake measurement = conversion of dry weight of botanical drugs preparations × number of daily doses; **TCM**, Traditional Chinese Medicine; **XY**, Xiaoyao Pill; **HHXY**, Honghua Xiaoyao Tablet; **DZXY**, Danzhi Xiaoyao Granules; **GQXY**, Guiqi Xiaoyao Mixture; **XYSJW**, Xiaoyao San Jiawei Granules

# Table S10 Summary of standards for formulated granule drugs

| **Nomenclature of TCM** | **Market Authorization** | **Standard-Setting Authority** | **Standard No.** | **Determination of content (HPLC)** |
| --- | --- | --- | --- | --- |
| Chaihu | 1121000108001 | CPC | YBZ-PFKL-2021011 | Each 1g of saikosaponin a (C_42_H_68_O_13_) should be 1.60 mg ~ 5.00 mg. |
| Shaoyao | 3221000035000 | CPC | YBZ-PFKL-2021002 | Each 1g of paeoniflorin (C_23_H_28_O_11_) should be 65.0 mg ~ 137.0 mg. |
| Baizhu | 3222000022000 | CPC | YBZ-PFKL-2021161 | The total amount of neochlorogenic acid (C_16_H_18_O_9_), chlorogenic acid (C_16_H_18_O_9_) and cryptochlorogenic acid (C_16_H1_8_O_9_) per 1g of this product should be 0.12 mg ~ 0.95 mg. The total amount of fructose (C_6_H_12_O_6_) and sucrose (C_12_H_22_O_11_) per 1g of this product should be 30.0 mg ~ 155.0 mg. |
| Danggui | 1121000016000 | CPC | YBZ-PFKL-2021037 | Each 1g of ferulic acid (C_10_H_10_O_4_) should be 0.70 mg~1.80 mg. |
| Fuling | 1122000440000 | BJMMPA | BJ-PFKL-2022044 | Each 1g of this product containing pachymic acid B (C_30_H_44_O_5_) should be 0.10mg ~ 0.40mg; pachymic acid A (C_31_H_46_O_5_) should be 0.08 mg ~ 0.30 mg. |
|  | 3222000446000 | JSMPA | JS-YBZ-2022216 | Each 1g of this product containing pachymic acid B (C_30_H_44_O_5_) should be 0.10mg ~ 0.70mg; pachymic acid A (C_31_H_46_O_5_) should be 0.09 mg ~ 0.60 mg. |
| Gancao | 1121000222000 | CPC | YBZ-PFKL-2021049 | Each 1g of this product containing liquiritin (C_21_H_22_O_9_) should be 15.0 mg ~ 35.0 mg; glycyrrhizic acid (C_42_H_62_O_16_) should be 29.0 mg ~ 80.0 mg. |
| Bohe | 1122000054001 | CPC | YBZ-PFKL-2021010 | Each 1g of rosmarinic acid (C_18_H_16_O_8_) should be 1.0 mg ~ 4.0 mg. |
| Mudanpi | 1122000466000 | BJMMPA | BJ-PFKL-2022067 | Each 1g of paeonol (C_9_H_10_O_3_) should be 2.4 mg ~ 6.6 mg. |
| Zhizi | 1121000028001 | CPC | YBZ-PFKL-2021150 | Each 1g of geniposide (C_17_H_24_O_10_) should be 64.0mg ~ 184.0mg. |
| Taizishen | 3223000062000 | CPC | YBZ-PFKL-2022046 | Each 1g of this product containing ginsenoside Rg1 (C_42_H_72_O_14_) should be 6.0 mg ~ 21.0 mg; pseudostellarin B (C_40_H_58_N_8_O_8_) should be 0.23 mg ~ 0.65 mg. |
| Haizao | 3223000037000 | JSMPA | JS-YBZ-2022317 | Each 1g of uridine (C_9_H_12_N_2_O_6_) should be 0.15 mg ~ 0.35 mg. |
| Kunbu | 3225000004000 | JSMPA | JS-YBZ-2024401 | Each 1g of galactose (C_6_H_12_O_6_) should be 4.0 mg ~ 12.0 mg. |
| Shanyao | 3222000059000 | JSMPA | JS-YBZ-2021095 | Each 1g of adenosine (C_10_H_13_N_5_O_4_) should be 0.30 mg ~ 1.50 mg; allantoin (C_4_H_6_N_4_O_3_) should be 7.0 mg ~ 42.0 mg. |
| Shudihuang | 3221000056000 | CPC | YBZ-PFKL-2021118 | Each 1g of rehmannioside D (C_27_H_42_O_20_) should be 0.70 mg ~ 2.70 mg. |
| Shanzhuyu | 3221000143000 | CPC | YBZ-PFKL-2021080 | The total amount of morroniside (C_17_H_26_O_11_) and loganin (C_17_H_26_O_10_) per 1g of this product should be 16.0mg ~ 31.0 mg. |
| Chenpi | 3221000074000 | CPC | YBZ-PFKL-2021025 | Each of 1g hesperidin (C_28_H_34_O_15_) should be 6.5mg ~ 14.5mg. |
| Banxia | 3222000186000 | JSMPA | JS-YBZ-2021082 | The number of theoretical plates should not be less than 3000 according to the peak of triglochinic acid. |
| Xiangyuan | 3221000095000 | CPC | YBZ-PFKL-2021129 | Each 1g of naringin (C_27_H_32_O_14_) should be 30.0 mg ~ 90.0 mg. |
| Foshou | 3222000094000 | CPC | YBZ-PFKL-2021044 | Each 1g of hesperidin (C_28_H_34_O_15_) should be 0.15 mg ~ 0.50 mg. |

**CPC**, Chinese Pharmacopoeia Commission; **BJMMPA**, Beijing Municipal Medical Products Administration; **JSMPA**, Jiangsu Medical Products Administration; **HPLC**, Performance Liquid Chromatography. For more detailed information on the formula granules, refer to **Table S10** and **Supplementary Material 2**.

# Table S11 Subgroup analysis of TPOAb, TgAb, FT3, FT4 and TSH

| Outcomes | Subgroup Comparison | n | SMD/MD (95%CI) | *I^2^*(%) | *^1^P* | *^2^P* | *^3^P* |
| --- | --- | --- | --- | --- | --- | --- | --- |
| TPOAb | Total | 5 | -0.74 [-1.02, -0.46] | 39 | <0.00001 | 0.16 | - |
|  | (XYS preparations + LT4) vs LT4 | 3 | -0.77 [-1.06, -0.47] | 0 | <0.00001 | 0.71 | 0.05 |
|  | (XY + LID) vs LID | 1 | -1.11 [-1.58, -0.63] | - | <0.00001 | - |  |
|  | (XY + SY) vs SY | 1 | -0.28 [-0.76, 0.19] | - | 0.24 | - |  |
| TgAb | Total | 5 | -0.66 [-1.05, -0.26] | 69 | 0.001 | 0.01 | - |
|  | (XYS preparations + LT4) vs LT4 | 3 | -0.92 [-1.37, -0.47] | 55 | <0.0001 | 0.11 | 0.05 |
|  | (XY + LID) vs LID | 1 | -0.14 [-0.58, 0.30] | - | 0.53 | - |  |
|  | (XY + SY) vs SY | 1 | -0.45 [-0.93, 0.03] | - | 0.07 | - |  |
| FT3 | Total | 5 | 0.31 [0.01, 0.61] | 49 | 0.04 | 0.10 | - |
|  | (XYS preparations + LT4) vs LT4 | 3 | 0.22 [-0.07, 0.50] | 0 | 0.13 | 0.71 | 0.03 |
|  | (XY + LID) vs LID | 1 | 0.04 [-0.40, 0.48] | - | 0.86 | - |  |
|  | (XY + SY) vs SY | 1 | 0.90 [0.40, 1.40] | - | 0.0004 | - |  |
| FT4 | Total | 5 | 0.58 [0.12, 1.04] | 77 | 0.01 | 0.002 | - |
|  | (XYS preparations + LT4) vs LT4 | 3 | 0.24 [-0.08, 0.55] | 18 | 0.14 | 0.30 | 0.001 |
|  | (XY + LID) vs LID | 1 | 0.79 [0.34, 1.25] | - | 0.0007 | - |  |
|  | (XY + SY) vs SY | 1 | 1.37 [0.84, 1.91] | - | <0.00001 | - |  |
| TSH | Total | 5 | -0.76 [-0.98, -0.54] | 0 | <0.00001 | 0.57 | - |
|  | (XYS preparations + LT4) vs LT4 | 3 | -0.82 [-1.12, -0.52] | 0 | <0.00001 | 0.49 | 0.48 |
|  | (XY + LID) vs LID | 1 | -0.52 [-0.97, -0.08] | - | 0.02 | - |  |
|  | (XY + SY) vs SY | 1 | -0.88 [-1.38, -0.39] | - | 0.0005 | - |  |

^1^p values for effect size; ^2^p values for heterogeneity; ^3^p values for between subgroup.

# Table S12 GRADE Summary of Outcomes for *Xiao-yao San* preparations Combined with LID/SY/LT4 compared to LID/SY/LT4/OS for HT

| **Outcomes** | **No. of studies** | **No. of participants** | **I² (%), Effect model (R/F)** | **Type of effect size** | **Effect size [95%CI]** | **Quality of evidence(GRADE)** |
| --- | --- | --- | --- | --- | --- | --- |
| TPOAb | 5 | 340 | 39, R | SMD | - 0.74 [-1.02, -0.46] | Low^a,d^ |
|  | 2 | 272 | 80, R | SMD | 0.13 [-0.41, 0.67] | Very low^a,b,d^ |
| TgAb | 5 | 340 | 69, R | SMD | - 0.66 [-1.05, -0.26] | Very low^a,b,d^ |
|  | 2 | 272 | 31, F | SMD | - 0.34 [-0.58, -0.10] | Low^a,d^ |
| FT3 | 5 | 340 | 49, R | SMD | 0.31 [0.01, 0.61] | Low^a,d^ |
|  | 2 | 272 | 86, R | SMD | - 0.07 [-0.72, 0.59] | Very low^a,b,d^ |
| FT4 | 5 | 340 | 77, R | SMD | 0.58 [0.12, 1.04] | Very low^a,b,d^ |
|  | 2 | 272 | 90, R | SMD | 0.15 [-0.62, 0.93] | Very low^a,b,d^ |
| TSH | 5 | 340 | 0, R | SMD | - 0.76 [-0.98, -0.54] | Low^a,d^ |
|  | 2 | 272 | 0, F | SMD | 0.79 [0.54, 1.04] | Low^a,d^ |
| TCMs | 2 | 120 | 98, R | SMD | - 2.54 [-6.62, 1.19] | Very low^a,b,d^ |
|  | 2 | 272 | 99, R | SMD | - 2.62 [-6.43, 1.19] | Very low^a,b,d^ |
| IL-6 | 1 | 80 | N, R | SMD | - 0.64 [-1.09, -0.19] | Very low^a,b,d^ |
|  | 1 | 162 | N, R | SMD | - 0.06 [-0.37, 0.25] | Very low^a,b,d^ |

a. The risk of bias is decreased by one level: There are some high risks and unclear risk bias, poor description of methodology including random sequence generation, allocation concealment, blinding and others.

b. The inconsistency is reduced by one level: I^2^ ≥ 50% for heterogeneity.

c. The inaccuracy is decreased by one level: Small sample sizes.

d. Too few studies
