# Supplementary material for: Efficacy of Xiaoyao-san preparations in treating Hashimoto’s thyroiditis: a meta-analysis and systematic review
Source: Front Pharmacol. 2025 Jun 13;16:1528506. doi: 10.3389/fphar.2025.1528506 (PMC12202410; doi:10.3389/fphar.2025.1528506)
Supplement: Supplementary file 2 [file Supplementaryfile2.zip › Supplementary Files 2/Detection Method 1 for Honghua Xiaoyao Tablets - Invention Patent Application Specification CN201210124256.4.pdf]

(19)中华人民共和国国家知识产权局

(12)发明专利申请

(10)申请公布号 CN 102944634 A
(43)申请公布日 2013.02.27

(21)申请号 201210124256.4

(22)申请日 2012.04.26

(71)申请人 江西普正制药有限公司
地址 331400 江西省吉安市峡江县工业园区

(72)发明人 肖军平　饶毅　吴永忠　余宝平
邹贵阳

(74)专利代理机构 南昌佳诚专利事务所 36117
代理人 文珊　闵蓉

(51)Int.Cl.
G01N 30/88(2006.01)

权利要求书 2 页　说明书 4 页　附图 1 页

(54)发明名称

一种红花逍遥片的质量检测方法

(57)摘要

一种红花逍遥片的质量检测方法，属于药物质量控制技术领域。包括如下步骤：对照品溶液的制备、供试品溶液的制备、色谱条件、以芍药苷为参照峰的标准指纹图谱的制定、指纹图谱的质量控制。本发明采用高效液相建立标准指纹图谱，通过指纹图谱得到量化参数，更有效的控制制剂的质量，精密度较高、稳定性、重复性均良好。

CN 102944634 A

1. 一种红花逍遥片的质量检测方法，其特征在于，包括如下步骤：

（1）对照品溶液的制备

取芍药苷对照品，置于容量瓶中，加甲醇溶解，摇匀，制成对照品溶液；

（2）供试品溶液的制备

取红花逍遥片制剂粉末，置锥形瓶中，加甲醇，密塞，称定重量，超声处理，放冷，再称定重量，用甲醇补足减失的重量，摇匀，经微孔滤膜滤过，滤液作为供试品溶液；

（3）色谱条件

色谱柱用十八烷基硅烷键合硅胶为填充剂，规格为：4.6mm×250mm，5μm；流动相：A 为乙腈，B 为 0.1% 磷酸水溶液；

采用梯度洗脱，洗脱程序如下：

0 分钟时，9% 流动相 A、91% 流动相 B；

5 分钟时，12% 流动相 A、88% 流动相 B；

25 分钟时，12% 流动相 A、88% 流动相 B；

30 分钟时，15% 流动相 A、85% 流动相 B；

65 分钟时，45% 流动相 A、55% 流动相 B；

85 分钟时，45% 流动相 A、55% 流动相 B；

流速：1.0mL/min；检测波长：230nm；柱温：25℃；

（4）以芍药苷为参照峰的标准指纹图谱的制定

吸取上述对照品溶液和供试品溶液各 8 批，每批 10μL，分别注入高效液相色谱仪中，按高效液相色谱法测定，记录色谱图，依据所得的 8 批对照品的指纹图谱，制定标准指纹图谱；

所述红花逍遥片的标准指纹图谱，各共有峰以芍药苷为参照峰，计算相对保留时间和相对峰面积；

（5）指纹图谱的质量控制

将红花逍遥片的供试品溶液指纹图谱与制定的标准指纹图谱进行比较，计算相似度，识别两者所具有的共同吸收峰的数量，确定相似度。

2. 根据权利要求 1 所述的一种红花逍遥片的质量检测方法，其特征在于：所述步骤（2）中超声处理时间为 25-35min。

3. 根据权利要求 1 所述的一种红花逍遥片的质量检测方法，其特征在于：所述步骤（2）中微孔滤膜的孔径为 0.45μm。

4. 根据权利要求 1 所述的一种红花逍遥片的质量检测方法，其特征在于：所述步骤（4）中相对保留时间的计算公式为：

相对保留时间 = 其它各组分峰的保留时间 / 参照峰的保留时间；

相对峰面积的计算公式为：

相对峰面积 = 其它各组分峰的峰面积 / 参照峰的峰面积；

所述参照峰为芍药苷峰。

5. 根据权利要求 1 所述的一种红花逍遥片的质量检测方法，其特征在于：步骤（4）中所述相对保留时间和相对峰面积分别为：

相对保留时间：1（0.192 ～ 0.193），2（0.579 ～ 0.591），3（0.795），4（1.000），5

(1.317 ～ 1.324),6(1.590 ～ 1.602),7(1.701 ～ 1.711),8(1.777 ～ 1.793),9(1.807 ～ 1.822),10(1.992 ～ 2.014);

相对峰面积:1(0.6296 ～ 0.7345),2(0.8666 ～ 1.1740),3(0.7374 ～ 0.8077),4(1.000),5(0.2065 ～ 0.2473),6(0.0378 ～ 0.0440),7(0.0485 ～ 0.0514),8(0.0532 ～ 0.0601),9(0.0314 ～ 0.0501),10(0.0614 ～ 0.0706)。

6. 根据权利要求 1 所述的一种红花逍遥片的质量检测方法,其特征在于:步骤(5)中所述确定相似度是指将红花逍遥片的供试品溶液指纹图谱与标准图谱比对,其相似度应为 0.90 ～ 1.00。

7. 根据权利要求 1 所述的一种红花逍遥片的质量检测方法,其特征在于:所述指纹图谱的质量控制,运用国家药典委员会制定的《中药指纹图谱相似度计算软件》(2004),经过多点校正,色谱峰的匹配,计算供试品溶液指纹图谱与制定的标准指纹图谱的相似度;指纹图谱相似度计算参数设置为:时间宽度为 0.2 秒;校正方式采用多点校正,校正色谱峰的匹配点为共有峰,峰 1(0.192 ～ 0.193),峰 2(0.579 ～ 0.591),峰 3(0.795),峰 4(1.000),峰 10(1.992 ～ 2.014)。

一种红花逍遥片的质量检测方法

技术领域

[0001]　本发明属于药物质量控制技术领域，特别涉及一种红花逍遥片的指纹图谱检测方法。

背景技术

[0002]　红花逍遥片由当归、白芍、白术、茯苓、红花、皂角刺、竹叶柴胡、薄荷、甘草九味药组成，功能主治为舒肝、理气、活血，用于肝气不舒，胸胁胀痛，头晕目眩，食欲减退，月经不调，乳房胀痛或伴见颜面黄褐斑。

[0003]　由于中药具有成分复杂多变、活性成分多、药效成分和毒性成分不甚明确等特点，红花逍遥片做为中成药，其药效不是来自任何单一的活性成分，其药效作用基本上是多种活性成分共同协作的结果。然而，目前对红花逍遥片的研究仅针对处方中当归、白芍、甘草、竹叶柴胡、薄荷的薄层鉴别方法和白芍的高效液相色谱含量测定。显然，对于红花逍遥片这样一个由 9 味中药组成的复方制剂，其质量控制检测方法过于简单，不能较准确全面所映产品的内在质量，也无法用于生产过程中、以及产品质量的有效控制。

[0004]　中药指纹图谱是指某些中药材或中药制剂经适当处理后，采用一定的分析手段，得到的能够标示其化学特征的色谱图。采用中药指纹图谱方式，一方面可以通过指纹图谱的特征性，有效的鉴别样品的真伪和产地；另一方面通过对主要指纹图谱特征峰的面积或比例的控制，能有效控制产品的质量，确保产品质量的稳定一致，从而保证产品安全有效。因此建立红花逍遥片的指纹图谱势在必行，建立一种能全面检测红花逍遥片中有效成分的质量检测方法具有重要意义。

发明内容

[0005]　本发明的目的是针对上面所述红花逍遥片的质量控制现状，提供一种红花逍遥片的质量检测方法，该方法提供一种基于高效液相色谱法的红花肖遥片的指纹图谱检测方法，可行到红花逍遥片的 HPLC（高效液相色谱）指纹图谱，可有效控制红花逍遥片的质量及保证其临床疗效。

[0006]　本发明的目的是通过以下技术方案予以实现的。

[0007]　一种红花逍遥片的质量检测方法，其特征在于，包括如下步骤：

（1）对照品溶液的制备

取芍药苷对照品，置于容量瓶中，加甲醇溶解，摇匀，制成对照品溶液；

（2）供试品溶液的制备

取红花逍遥片制剂粉末，置锥形瓶中，加入甲醇，密塞，称定重量，超声处理，放冷，再称定重量，用甲醇补足减失的重量，摇匀，经微孔滤膜滤过，滤液作为供试品溶液；

（3）色谱条件

色谱柱用十八烷基硅烷键合硅胶为填充剂，规格为：4.6mm×250mm，5μm；流动相：A 为乙腈，B 为 0.1% 磷酸水溶液；

采用梯度洗脱，洗脱程序如下：

0 分钟时，9% 流动相 A、91% 流动相 B；

5 分钟时，12% 流动相 A、88% 流动相 B；

25 分钟时，12% 流动相 A、88% 流动相 B；

30 分钟时，15% 流动相 A、85% 流动相 B；

65 分钟时，45% 流动相 A、55% 流动相 B；

85 分钟时，45% 流动相 A、55% 流动相 B；

流速：1.0mL/min；检测波长：230nm；柱温：25℃；

（4）以芍药苷为参照峰的标准指纹图谱的制定

吸取上述对照品溶液和供试品溶液各 8 批，每批 10μL，分别注入高效液相色谱仪中，按高效液相色谱法测定，记录色谱图，依据所得的 8 批对照品的指纹图谱，制定标准指纹图谱；

所述红花逍遥片的标准指纹图谱，各共有峰以芍药苷为参照峰，计算相对保留时间和相对峰面积；

（5）指纹图谱的质量控制

将红花逍遥片的供试品溶液指纹图谱与制定的标准指纹图谱进行比较，计算相似度，识别两者所具有的共同吸收峰的数量，确定相似度。

[0008] 所述步骤（2）中超声处理时间为 25 ～ 35min。

[0009] 所述步骤（2）中微孔滤膜的孔径为 0.45μm。

[0010] 所述步骤（4）中相对保留时间的计算公式为：相对保留时间＝其它各组分峰的保留时间／参照峰的保留时间。

[0011] 相对峰面积的计算公式为：相对峰面积＝其它各组分峰的峰面积／参照峰的峰面积。

[0012] 所述参照峰为芍药苷峰。

[0013] 步骤（4）中所述相对保留时间和相对峰面积分别为：

相对保留时间：1（0.192 ～ 0.193），2（0.579 ～ 0.591），3（0.795），4（1.000），5（1.317 ～ 1.324），6（1.590 ～ 1.602），7（1.701 ～ 1.711），8（1.777 ～ 1.793），9（1.807 ～ 1.822），10（1.992 ～ 2.014）；

相对峰面积：1（0.6296 ～ 0.7345），2（0.8666 ～ 1.1740），3（0.7374 ～ 0.8077），4（1.000），5（0.2065 ～ 0.2473），6（0.0378 ～ 0.0440），7（0.0485 ～ 0.0514），8（0.0532 ～ 0.0601），9（0.0314 ～ 0.0501），10（0.0614 ～ 0.0706）；

步骤（5）中所述确定相似度是指将红花逍遥片的供试品溶液指纹图谱与标准图谱比对，其相似度应为 0.90 ～ 1.00。

[0014] 本发明所述指纹图谱的质量控制，运用国家药典委员会制定的《中药指纹图谱相似度计算软件》（2004），经过多点校正，色谱峰的匹配，计算供试品溶液指纹图谱与制定的标准指纹图谱的相似度；指纹图谱相似度计算参数设置为：时间宽度为 0.2 秒；校正方式采用多点校正，校正色谱峰的匹配点为共有峰，峰 1（0.192 ～ 0.193），峰 2（0.579 ～ 0.591），峰 3（0.795），峰 4（1.000），峰 10（1.992 ～ 2.014）。

[0015] 与现有技术相比，本发明的有益效果是：a. 本发明采用高效液相建立标准指纹图

谱，通过指纹图谱得到量化参数，更有效的控制制剂的质量。b. 本发明采用高效液相色谱指纹图谱，以芍药药材中的有效成分（芍药苷）特征为主的指纹图谱来全面控制红花逍遥片的质量，更加完善了制剂的质量控制。c. 通过实验证明，本发明质量控制方法对红花逍遥片的质量控制更为有效，方法精密度较高、稳定性、重复性均良好。能有效控制产品的质量，确保产品质量稳定可靠，药性和疗效基本一致，从而保证产品的安全有效。

附图说明

[0016] 图 1 为本发明所述以芍药苷为参照制得的标准指纹图谱。

[0017] 图 2 为本发明所述红花逍遥片制得的 8 批样品指纹图谱。

具体实施方式

[0018] 实施例 1：红花逍遥片标准指纹图谱建立。

[0019] （1）仪器及试剂：高效液相色谱仪：岛津 LC － 10ATvp 高效液相色谱仪（SPD － M10Avp 检测器，CLASS-VP 软件）；数控超声波清洗器：KQ-250DB 型，昆山市超声仪器有限公司；十万分之一天平：岛津 AUW220；万分之一天平：AB104 － N 型 ，梅特勒－托利多；Millipore 超纯水仪。

[0020] 乙腈：山东禹王实验有限公司化工分公司；甲醇：上海振兴化工一厂；磷酸（优级纯）：天津精细化学品开发有限公司；超纯水。

[0021] 芍药苷对照品：中国药品生物制品检定所；红花逍遥片：江西普正制药有限公司。

[0022] （2）对照品溶液的制备。

[0023] 取芍药苷约 23mg，精密称定，置 25mL 量瓶中，用甲醇定容至刻度，摇匀，精密吸取 2mL，置 25mL 量瓶中，用甲醇定容至刻度，摇匀即得。

[0024] （3）供试品溶液的制备。

[0025] 取本品 20 片，除去薄膜衣，研细，取约 0.35g，精密称定，置锥形瓶中，精密加入甲醇 25mL，密塞，称定重量，超声处理 25min，放冷，再称定重量，用甲醇补足减失的重量，摇匀，经微孔滤膜（0.45μm）滤过，即得。

[0026] （4）色谱条件。

[0027] 色谱柱用十八烷基硅烷键合硅胶为填充剂（4.6 mm×250 mm，5μm）；流动相：A 为乙腈，B 为 0.1% 磷酸水溶液，梯度洗脱（见表 1）；进样量：10μL；检测波长：230nm；流速：1.0mL/min；柱温：25℃。

[0028] 表 1 液相系统洗脱程序。

时间（min）	乙腈	0.1%磷酸水溶液
0	9	91
5	12	88
25	12	88
30	15	85
65	45	55
85	45	55

[0029] （5）方法学考察。

[0030] 精密度试验：取同一份供试品溶液，在上述液相色谱条件下，重复进样 6 次，每次

进样 10μL，以芍药苷峰为参照峰，考察主要色谱峰的相对保留时间、峰面积比值的一致性。结果单峰面积大于或等于 5% 以上的主要色谱峰，其相对保留时间和相对峰面积的 RSD 小于 3.32%，表明精密度良好。

[0031]　溶液稳定性试验：取同一份供试品溶液，在上述液相色谱条件下，分别在 0、2、4、8、12、24 小时检测指纹图谱，每次进样 10μL，考察主要色谱峰的相对保留时间、峰面积比值的一致性。结果单峰面积大于或等于 5% 以上的主要色谱峰，其相对保留时间和相对峰面积的 RSD 小于 3.08%，表明供试品溶液 24 小时内相对稳定。

[0032]　重复性试验：取同一批样品，按供试品制备方法制备 6 份供试品溶液，在上述液相色谱条件下，进样分析，考察主要色谱峰的相对保留时间、峰面积比值的一致性。结果单峰面积大于或等于 5% 以上的主要色谱峰，其相对保留时间和相对峰面积的 RSD 小于 3.37%，表明该方法重复性好。

[0033]　(6) 以芍药苷为参照峰的标准指纹图谱的制定。

[0034]　精密吸取上述对照品和供试品溶液各 10μL，分别注入高效液相色谱仪中，照高效液相色谱法测定，记录色谱图；依据所得的 8 批对照品的指纹图谱，制定标准指纹图谱，见图 1。

[0035]　照上述液相色谱条件，将 8 批红花逍遥片的供试品溶液进行测定，色谱图见图 2。比较对照品的色谱图和计算相对保留时间，其中有 10 个峰确定为共有峰，其中 4 号峰为芍药苷峰。依据《中药注射剂指纹图谱研究的技术要求》，制定了红花逍遥片的标准指纹图谱技术参数。以芍药苷峰为内参照峰，计算各共有峰的相对保留时间、相对峰面积。

[0036]　相对保留时间：1 (0.192 ～ 0.193)，2 (0.579 ～ 0.591)，3 (0.795)，4 (1.000)，5 (1.317 ～ 1.324)，6 (1.590 ～ 1.602)，7 (1.701 ～ 1.711)，8 (1.777 ～ 1.793)，9 (1.807 ～ 1.822)，10 (1.992 ～ 2.014)；

相对峰面积：1 (0.6296 ～ 0.7345)，2 (0.8666 ～ 1.1740)，3 (0.7374 ～ 0.8077)，4 (1.000)，5 (0.2065 ～ 0.2473)，6 (0.0378 ～ 0.0440)，7 (0.0485 ～ 0.0514)，8 (0.0532 ～ 0.0601)，9 (0.0314 ～ 0.0501)，10 (0.0614 ～ 0.0706)。

[0037]　(7) 相似度评价。

[0038]　运用国家药典委员会制定的用于生成共有模式的《中药指纹图谱相似度计算软件》A 版 (2004)，经过多点校正，色谱峰的匹配，计算 8 批红花逍遥片的相似度，结果见表 2。

[0039]　表 2　8 批红花逍遥片 HPLC 指纹图谱相似度结果表。

批号	100101	100102	100103	100104	100105	100106	100107	100108	对照指纹图谱
100101	1.000	0.985	0.995	0.998	0.991	0.992	0.999	0.983	0.995
100102	0.985	1.000	0.997	0.992	0.999	0.998	0.990	0.999	0.997
100103	0.995	0.997	1.000	0.998	0.999	0.999	0.998	0.996	1.000
100104	0.998	0.992	0.998	1.000	0.996	0.997	1.000	0.991	0.999
100105	0.991	0.999	0.999	0.996	1.000	0.999	0.995	0.998	0.999
100106	0.992	0.998	0.999	0.997	0.999	1.000	0.996	0.998	0.999
100107	0.999	0.990	0.998	1.000	0.995	0.996	1.000	0.989	0.998
100108	0.983	0.999	0.996	0.991	0.998	0.998	0.989	1.000	0.996
对照指纹图谱	0.995	0.997	1.000	0.999	0.999	0.999	0.998	0.996	1.000

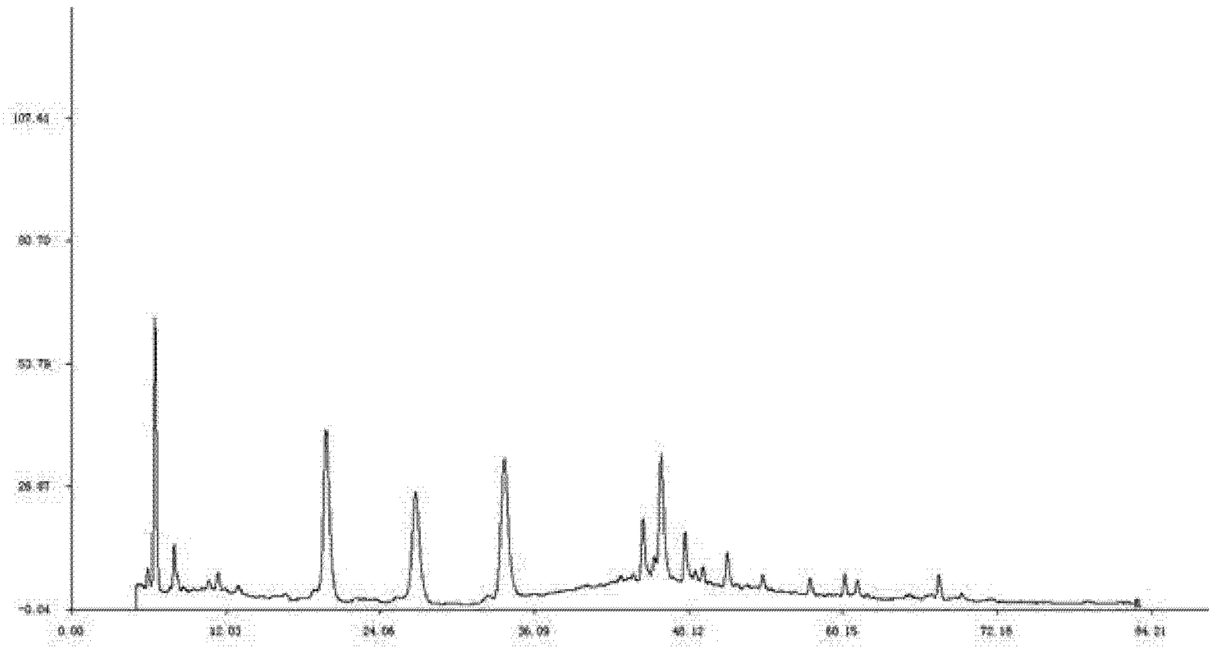
107.61
80.70
53.78
26.87
-0.04
0.00
12.03
24.06
36.09
48.12
60.15
72.18
84.21

图 1

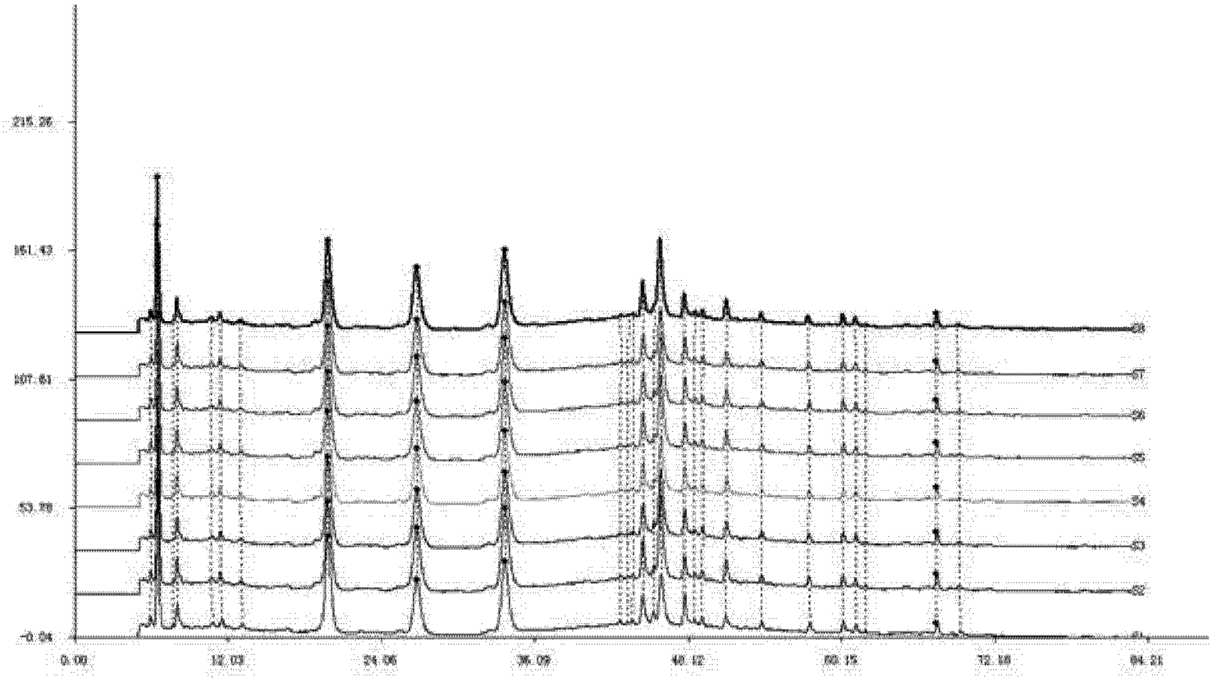
215.26
161.43
107.61
53.78
-0.04
0.00
12.03
24.06
36.09
48.12
60.15
72.18
84.21

图 2
