# Supplementary material for: Efficacy of Xiaoyao-san preparations in treating Hashimoto’s thyroiditis: a meta-analysis and systematic review
Source: Front Pharmacol. 2025 Jun 13;16:1528506. doi: 10.3389/fphar.2025.1528506 (PMC12202410; doi:10.3389/fphar.2025.1528506)
Supplement: Supplementary file 2 [file Supplementaryfile2.zip › Supplementary Files 2/Detection Method 2 for Honghua Xiaoyao Tablets - Invention Patent Application Specification CN201811237371.6.pdf]

(19)中华人民共和国国家知识产权局

(12)发明专利申请

(10)申请公布号 CN 109682918 A
(43)申请公布日 2019.04.26

(21)申请号 201811237371.6

(22)申请日 2018.10.23

(71)申请人 江西普正制药有限公司
地址 331409 江西省吉安市峡江县工业园区

(72)发明人 梁佳 李小锋 康兴东 杜剑松 毛金娣 刘厚权

(74)专利代理机构 南昌佳诚专利事务所 36117
代理人 吕道锋

(51)Int.Cl.
G01N 30/90(2006.01)

权利要求书1页 说明书7页

(54)发明名称

一种红花逍遥片的质量检测方法

(57)摘要

一种红花逍遥片的质量检测方法，属于药物的技术领域。现有技术仅限对当归、白芍、甘草、竹叶柴胡、薄荷薄层鉴别和芍药苷含量测定进行质量控制，该方法新增采用薄层色谱法对茯苓进行鉴别，并用高效液相色谱法对甘草苷进行含量测定。本发明质量控制方法对产品质量控制更有效，方法精密度、灵敏度、稳定性都较高。

CN 109682918 A

1.一种红花逍遥片的质量检测方法,其特征在于该方法包括以下步骤:

鉴别:

取本品5片,除去薄膜衣,研细,加甲醇30ml,超声处理30分钟,滤过,滤液蒸干,残渣加三氯甲烷5ml振摇溶解5分钟,倾出三氯甲烷液,浓缩至0.5ml,作为供试品溶液;另取茯苓对照药材1g,同法制成对照药材溶液;照薄层色谱法;试验,吸取对照品溶液、供试品溶液各10μl,分别点于同一硅胶G薄层板上,以环已烷-乙醚为展开剂,展开,取出,晾干,置紫外光灯下检视;供试品色谱中,在与对照品色谱相应的位置上,显相同颜色的荧光斑点;

含量测定:

色谱条件与系统适用性试验 以十八烷基硅烷键合硅胶为填充剂;以乙腈-0.1%磷酸溶液(15 : 85)为流动相;检测波长为230nm;理论板数按芍药苷峰计算应不低于2000;

对照品溶液的制备取甘草苷对照品适量,精密称定,加甲醇制成每1ml含20μg的溶液,即得;

供试品溶液的制备取本品20片,除去薄膜衣,精密称定,研细,取约0.25g,精密称定,置25ml量瓶中,加甲醇至近刻度,超声处理30分钟,放冷,加甲醇至刻度,摇匀,滤过,取续滤液,即得;

测定法分别精密吸取对照品溶液与供试品溶液各10μl,注入液相色谱仪,测定,即得。

2.根据权利要求1所述的一种红花逍遥片的质量检测方法,其特征在于其配方中药物配比包含:当归240~280份、白芍240~280份、白术240~280份、茯苓240~280份、红花30~70份、皂角刺60~100份、竹叶柴胡240~280份、薄荷20~60份、甘草170~210份。

3.根据权利要求1所述的一种红花逍遥片的质量检测方法,其特征在于:鉴别步骤中所述环已烷与乙醚的体积比为11 : 6。

4.根据权利要求1所述的一种红花逍遥片的质量检测方法,其特征在于:鉴别步骤中所述紫外光灯的波长为365nm。

5.根据权利要求1所述的一种红花逍遥片的质量检测方法,其特征在于:含量测定步骤中所述乙腈与0.1%磷酸溶液的体积比为15 : 85。

6.根据权利要求1所述的一种红花逍遥片的质量检测方法,其特征在于:含量测定步骤中所述超声处理的功率为90W,频率为59kHz。

一种红花逍遥片的质量检测方法

[0001] 技术领域：

本发明属于药物的技术领域，具体属于一种红花逍遥片的质量检测方法。

[0002] 背景技术：

红花逍遥片由当归、白芍、白术、茯苓、红花、皂角刺、竹叶柴胡、薄荷、甘草九味药组成，诸药合用，共奏舒肝、理气、活血的功效。用于肝气不舒，胸胁胀痛，头晕目眩，食欲减退，月经不调，乳房胀痛或伴见颜面黄褐斑。

[0003] 红花逍遥片功能主治作用明显，用之疗效较好，但红花逍遥片目前的质量控制方法存在一些不足，仅限当归、白芍、甘草、竹叶柴胡、薄荷薄层鉴别和芍药苷含量测定予以质量控制，难以对该制剂进行更有效的质量控制。

[0004] 发明内容：

本发明的目的在于提供一种红花逍遥片的质量检测方法，该方法通过对茯苓薄层鉴别和甘草苷含量测定加以质量控制，使之对产品的质量控制更加有效。

[0005] 本发明是通过下述的技术方案来实现的。

[0006] 本发明所述的红花逍遥片是由各药材按以下配比(以重量计)组成：当归240～280份、白芍240～280份、白术240～280份、茯苓240～280份、红花30～70份、皂角刺60～100份、竹叶柴胡240～280份、薄荷20～60份、甘草170～210份等组成。

[0007] 本发明质量控制方法包括以下鉴别和/或含量测定方法：

鉴别方法包括以下方法中的一种或几种：

取本品5片，除去薄膜衣，研细，加甲醇30ml，超声处理30分钟，滤过，滤液蒸干，残渣加三氯甲烷5ml振摇溶解5分钟，倾出三氯甲烷液，浓缩至0.5ml，作为供试品溶液。另取茯苓对照药材1g，同法制成对照药材溶液。照薄层色谱法(中国药典2015年版通则0502)试验，吸取对照品溶液、供试品溶液各10μl，分别点于同一硅胶G薄层板上，以环已烷-乙醚(体积比为11 ： 6)为展开剂，展开，取出，晾干，置紫外光灯(365nm)下检视。供试品色谱中，在与对照品色谱相应的位置上，显相同颜色的荧光斑点。

[0008] 含量测定：

色谱条件与系统适用性试验：以十八烷基硅烷键合硅胶为填充剂；以乙腈-0.1%磷酸溶液(体积比为15 ： 85)为流动相；检测波长为230nm。理论板数按芍药苷峰计算应不低于2000。

[0009] 对照品溶液的制备：取甘草苷对照品适量，精密称定，加甲醇制成每1ml含20μg的溶液，即得。

[0010] 供试品溶液的制备：取本品20片，除去薄膜衣，精密称定，研细，取约0.25g，精密称定，置25ml量瓶中，加甲醇至近刻度，超声处理(功率90W，频率59kHz)30分钟，放冷，加甲醇至刻度，摇匀，滤过，取续滤液，即得。

[0011] 测定法：分别精密吸取对照溶液与供试品溶液各10μl，注入液相色谱仪，测定，即得。

[0012] 本发明质量控制方法在与现有技术对比下，新增采用薄层色谱法对茯苓进行鉴

别，并采用高效液相色谱法测定红花逍遥片中的甘草苷的含量进行实验研究。茯苓和甘草为处方中的主药，用药量较大，故对其制定了薄层鉴别方法和含量测定方法以控制产品质量，可以达到对产品质量的有效控制，并通过对各方法筛选，使得方法精密度、灵敏度、稳定性都较高。

具体实施方式

[0013] 为了更好的解释本发明的实施，提供下述测定方法的实施实例。这些实施实例仅仅是解释、而不是限制本发明的范围。下面通过典型的实例对本发明做进一步的说明。

[0014] 实施例1：茯苓的薄层鉴别方法考察。

[0015] 茯苓为处方中主药之一，用药量较大，茯苓含多种三萜类物质如茯苓酸等，故对其制定了薄层鉴别方法以控制产品质量。经实验，阴性无干扰，方法如下：

1.1 提取条件的选择：由于制剂中茯苓为水煎煮提取入药，因此，参照药典及文献提取方法分别比较采用乙酸乙酯超声处理和采用甲醇超声提取后以三氯甲烷萃取，结果以后者提取效果为佳，因此选择甲醇超声提取后以三氯甲烷萃取的处理方法。

[0016] 1.2 薄层板：0.5%羧甲基纤维素钠溶液制备的硅胶G板，厚度为500μm。

[0017] 1.3 显色剂的选择：分别采用喷以10%硫酸乙醇显色和置365nm紫外光灯下检视，结果发现后者显色效果更佳，故选用置365nm紫外光灯下检视。

[0018] 1.4 展开剂的选择：参照中国药典分别选用环已烷-乙酸乙酯-甲酸(19 ：5 ：0.5)、石油醚(30-60℃)-乙醚(6 ：4)、环已烷-乙酸乙酯(11 ：6)、环已烷-乙醚(11 ：6)为展开剂，结果以后者分离效果为佳，且Rf值适中，同时阴性样品无干扰，故选用环已烷-乙醚(11 ：6)为展开剂。

[0019] 实施例2：甘草苷含量测定方法的考察。

[0020] 白芍与甘草是中医临床上常用的药材，现代研究证明显示，甘草的解痉作用与白芍有协同作用，合用比两者单用显著增强。且中国药典2015年版所收载的白芍和甘草标准中均建立了其含量测定方法，原标准中已建立了制剂中芍药苷的含量测定方法，本次质量标准提高研究中，对制剂中甘草苷的含量测定方法进行了研究，并建立了其含量测定方法。

[0021] 仪器与试药 HP-1100高效液相色谱仪，德国：包括1322A在线脱气机、G1311A四元梯度泵、G1315A二极管阵列检测器、HP化学工作站；AG-135电子天平，梅特勒--托利多；CQ-250超声波清洗器，上海船舶电子设备研究所；SimplicityTM个人型超纯水系统，Millipore公司；78X -2型片剂四用测定仪，上海黄海药检仪器厂；甘草苷对照品，供含量测定用，批号：111610-201005，中国食品药品检定研究院；红花逍遥片，批号：150201、150401、150501、150601、150901、150902、151001、151101、151201、151202、160101、160102、160201、160301、160302，色谱纯乙腈，其它试剂均为分析纯。

[0022] 色谱条件的选择中国药典2015年版一部甘草项下采用了高效液相色谱法测定甘草苷的含量，因此，我们以上述条件为基础，采用高效液相色谱法测定红花逍遥片中的甘草苷的含量进行实验研究，结果确定了以下色谱条件：

色谱柱：Dikma Diamonsil C18(2)柱(4.6mm×250mm 5μm)，流动相：乙腈-0.1%磷酸(15:85)，流速：1ml/min，检测波长230nm，柱温：30℃。在此条件下，甘草苷对照品及供试品均在16min相同时间有一色谱峰。

[0023] 甘草阴性对照试验 按处方取不含甘草的其他药材，照本制剂的制备工艺制成阴性对照样品，再按含量测定供试品溶液制备方法制成阴性对照品溶液，取阴性对照溶液10μl，按样品测定方法注入液相色谱仪，结果在对照品甘草苷色谱峰相应的位置上，无色谱峰出现，甘草阴性对照对样品对甘草苷测定无干扰。

[0024] 提取溶剂的选择甘草苷为黄酮类化合物，中国药典2015年版一部“甘草”药材含量测定项下以70%乙醇提取，文献中采用16%乙腈提取，原标准中芍药苷的含量测定方法中采用甲醇提取，因此，我们分别选择甲醇、70%乙醇、16%乙腈为提取溶剂，取红花逍遥片适量，研细，分别精密称约0.25g共3份，置25ml量瓶中，分别加入上述溶剂至近刻度，超声处理30分钟，放冷，分别用上述溶剂加至刻度，混匀，滤过。吸取续滤液，用微孔滤膜(0.45μm)滤过，取滤液，即得。分别精密吸取上述供试品溶液和对照品溶液各10μl，分别注入液相仪中，测得甘草苷含量结果见表1。

[0025] 表1 不同溶剂提取甘草苷含量

提取溶剂	甲醇	70%乙醇	16%乙腈
含量（mg/片）	0.67	0.64	0.62

结果显示，三者含量无明显差异，但甲醇提取液中甘草苷含较高，且原标准中芍药苷采用甲醇提取，故采用甲醇作为提取溶剂。

[0026] 提取时间的考察 取本品适量，研细，称取约0.25g，共4份，精密称定，置25ml量瓶中，分别加甲醇至近刻度，分别超声处理15、30、45、60分钟，放冷，用甲醇加至刻度，混匀，滤过，吸取续滤液，用微孔滤膜(0.45μm)滤过，即得。分别精密吸取对照品溶液、供试品溶液各10μl，注入液相色谱仪，测定，结果见表2。

[0027] 表2 不同提取时间对测定结果的影响

提取时间(分钟)	15	30	45	60
含量（mg/片）	0.58	0.65	0.66	0.63

上述结果显示，超声处理45分钟与30分钟无明显差异，为方便操作，故选用超声处理30分钟。

[0028] 提取溶剂用量的考察 取本品适量，研细，称取约0.25g，共3份，精密称定，置100ml具塞三角烧瓶中，精密加入甲醇25ml、50ml、75ml，称定重量，超声处理30分钟，放冷，用甲醇补足减失的重量，混匀，滤过，吸取续滤液，用微孔滤膜(0.45μm)滤过，即得。分别精密吸取对照品溶液、供试品溶液各10μl，注入液相色谱仪，测定。结果见表3。

[0029] 表3 不同提取溶剂量对测定结果的影响

提取溶剂量（ml）	25	50	75
含量（mg/片）	0.66	0.62	0.68

上述结果显示，三者提取效果无明显差异，为方便操作、节省溶剂，宜选用25ml进行提

取。

[0030] 线性关系的考察 精密称取甘草苷对照品6.60mg，置25ml量瓶中，加甲醇至刻度，摇匀，即得(每1ml含甘草苷0.264mg)。分别精密吸取适量，加甲醇稀释成以下浓度：0.132mg/ml、0.0528mg /ml、0.0264mg /ml、0.0132mg/ml、0.01056mg /ml、0.00528mg /ml。分别吸取10μl，注入液相色谱仪中，按上述色谱条件测定对照品峰面积，将样品浓度与峰面积进行回归处理，回归方程为：Y=15046.27X－1.295，r=0.9999，芍药苷对照品在0.0189～0.2268mg/ml范围内进样量与峰面积呈良好的线性关系，结果见表4。

[0031] 表4 甘草苷对照品线性关系测定结果

样号	1	2	3	4	5	6
浓度 mg/ml	0.132	0.0528	0.0264	0.0132	0.01056	0.00528
峰面积	4809.85	1927.7	956.35	478.75	385.1	184.9
回归方程：	Y=15046.27X－1.295		r=1.0			
线性范围：0.02985～0.2985mg/ml						

2.8 精密度试验 精密吸取供试品溶液10μl，按上述色谱条件，重复进样5次，测定供试品溶液中甘草苷峰面积值，经统计学处理RSD为0.53%。结果见表5。

[0032] 表5 精密度试验结果

样号	1	2	3	4	5	平均	RSD（%）
供试品峰面积	1290.4	1297.5	1287.1	1305.1	1295.1	1295.04	0.53

2.9 稳定性试验 精密吸取供试品溶液10μl，按上述色谱条件，每隔一定时间，测定供试品溶液中甘草苷峰面积值，经测定10小时内供试品溶液中甘草苷峰面积值基本保持不变；经统计学处理RSD为0.74%。结果见表6。

[0033] 表6 稳定性试验结果

时间	0h	1.0h	2.0h	4.0h	8.0h	12h	平均	RSD（%）
峰面积	1290.4	1287.1	1295.1	1282.9	1310.7	1294.2	1293.4	0.74

2.10重现性试验 取本品适量，研细，称取约0.25g，共6份，精密称定，置25ml量瓶中，照含量测定项下实验方法制成供试品溶液，按上述色谱条件进行测定，测定结果经统计学处理，RSD为2.11%。结果见表7。

[0034] 表7 重现性试验结果

样号	1	2	3	4	5	6	平均	RSD(%)
含量 mg/片	0.65	0.63	0.64	0.67	0.64	0.65	0.647	2.11

2.11 加样回收率试验 取本品适量，研细，称取约0.15g，共6份，精密称定，置25ml量瓶

中，各精密加入甘草苷对照品溶液(0.264mg/ml)1ml，加甲醇至近刻度，超声处理30分钟，放冷，用甲醇加至刻度。摇匀，滤过，取适量用微孔滤膜(0.45μm)滤过，吸取10μl续滤液注入液相色谱仪中进行含量测定，计算回收率。结果见表8。

$$回收率(\%)=\frac{测出甘草苷总量-供试品中甘草苷量}{加入甘草苷量}\times 100\%$$

[0035] 表8 回收率测定结果

样号	样品中甘草苷量（mg）	加入甘草苷量（mg）	测出甘草苷量（mg）	回收率（%）
1	0.2501	0.264	0.5122	99.28
2	0.2492	0.264	0.5005	95.19
3	0.2486	0.264	0.5064	97.65
4	0.2475	0.264	0.5119	100.15
5	0.2463	0.264	0.5085	99.32
6	0.2514	0.264	0.5068	96.74

平均回收率98.055% RSD=1.91%

2.12 样品测定 取本品适量，研细，取约0.25g，精密称定，置25ml量瓶中，加甲醇至近刻度，超声(功率250w，频率33kHz)处理30分钟。放冷，用甲醇加至刻度，摇匀，滤过，取续滤液通过微孔滤膜(0.45μm)，吸取10μl续滤注入液相色谱仪中进行含量测定即得。结果见表9。

[0036] 表9 红花逍遥片中甘草苷含量

批号	芍药苷含量(mg/片)	甘草苷含量(mg/片)
150501	1.72	0.55
150601	1.88	0.59
150901	2.09	0.63
151001	1.91	0.70
151101	1.96	0.68
151201	2.17	0.65
160101	1.65	0.68
160201	1.80	0.69
160301	1.73	0.70
160302	1.82	0.69

根据表9测定的结果，平均每粒含甘草苷0.66mg，考虑药材来源、操作过程等因素的差异，因此，甘草苷按45%转移率计算，根据中国药典中甘草药材中甘草苷的含量(≥0.5%)，则本品中甘草苷的含量暂规定为：195×45%×0.5%/1000=0.44mg/片，即规定本品每粒含甘草以甘草苷($C_{21}H_{22}O_9$)计，不得少于0.44mg。

[0037] 实施例3：红花逍遥片的质量控制方法

鉴别：

取本品5片，除去薄膜衣，研细，加甲醇30ml，超声处理30分钟，滤过，滤液蒸干，残渣加三氯甲烷5ml振摇溶解5分钟，倾出三氯甲烷液，浓缩至0.5ml，作为供试品溶液。另取茯苓对照药材1g，同法制成对照药材溶液。照薄层色谱法(中国药典2015年版通则0502)试验，吸取对照品溶液、供试品溶液各10μl，分别点于同一硅胶G薄层板上，以环已烷-乙醚(11 : 6)为展开剂，展开，取出，晾干，置紫外光灯(365nm)下检视。供试品色谱中，在与对照品色谱相应的位置上，显相同颜色的荧光斑点。

[0038] 含量测定。

[0039] 照高效液相色谱法(中国药典2015年版通则0512)测定。

[0040] 色谱条件与系统适用性试验以十八烷基硅烷键合硅胶为填充剂；以乙腈-0.1%磷酸溶液(15 : 85)为流动相；检测波长为230nm。理论板数按芍药苷峰计算应不低于2000。

[0041] 对照品溶液的制备取甘草苷对照品适量，精密称定，加甲醇制成每1ml含20μg的溶液，即得。

[0042] 供试品溶液的制备取本品20片，除去薄膜衣，精密称定，研细，取约0.25g，精密称定，置25ml量瓶中，加甲醇至近刻度，超声处理(功率90W，频率59kHz)30分钟，放冷，加甲醇至刻度，摇匀，滤过，取续滤液，即得。

[0043] 测定法分别精密吸取对照溶液与供试品溶液各10μl，注入液相色谱仪，测定，即得。

[0044] 本品每片含甘草以甘草苷($C_{21}H_{22}O_9$)计，不得少于0.44mg。
