# Supplementary material for: Efficacy of Xiaoyao-san preparations in treating Hashimoto’s thyroiditis: a meta-analysis and systematic review
Source: Front Pharmacol. 2025 Jun 13;16:1528506. doi: 10.3389/fphar.2025.1528506 (PMC12202410; doi:10.3389/fphar.2025.1528506)
Supplement: Supplementary file 2 [file Supplementaryfile2.zip › Supplementary Files 2/Preparation of Honghua Xiaoyao Tablets - Invention Patent Application Specification CN201010596946.0.pdf]

(19) 中华人民共和国国家知识产权局

(12) 发明专利申请

(10) 申请公布号 CN 102204967 A
(43) 申请公布日 2011.10.05

(21) 申请号 201010596946.0
(22) 申请日 2010.12.21
(71) 申请人 江西普正制药有限公司
地址 331409 江西省吉安市峡江县普正工业园区
(72) 发明人 肖军平 吴永忠 李旭 刘立鼎
(74) 专利代理机构 南昌佳诚专利事务所 36117
代理人 闵蓉 张建新
(51) Int.Cl.
A61K 36/71 (2006.01)
A61K 47/40 (2006.01)
A61K 9/30 (2006.01)
A61K 9/16 (2006.01)
A61K 9/48 (2006.01)
A61P 1/16 (2006.01)
A61P 7/00 (2006.01)
A61P 29/00 (2006.01)
A61P 25/00 (2006.01)
A61P 1/14 (2006.01)
A61P 15/00 (2006.01)
A61P 17/00 (2006.01)

权利要求书 1 页 说明书 6 页

(54) 发明名称

一种红花逍遥片的制备方法

(57) 摘要

一种红花逍遥片的制备方法，涉及一种中药制剂的制备方法。依次包括如下步骤：①采用水蒸汽蒸馏的方法，提取出当归、白术、薄荷三味中药的挥发油；②提取挥发油后的药渣与白芍、茯苓、红花、皂角刺、竹叶柴胡、甘草一起以水为溶剂，用超声波提取得水提取物；③将提取的挥发油用 β-环糊精包结，即得挥发油 β-环糊精包结物；④将步骤②所得水提取物和步骤③所得挥发油 β-环糊精包结物加入淀粉，制粒压制成片，包薄膜衣，即得。本发明能够使药材细胞内的有效成份迅速溶出，且不破坏有效成份的化学结构，提取率高。在挥发油加入方法上采用 β-环糊精包结技术，有效地增加中药挥发性成分在制剂中的保留指数。

CN 102204967 A

1. 一种红花逍遥片的制备方法，包括下列重量份的原料药：当归 220g-300g、白芍 220g-300g、白术 220g-300g、茯苓 220g-300g、红花 30g-70g、皂角刺 60g-100g、竹叶柴胡 220g-300g、薄荷 20g-60g、甘草 160g-240g，其特征在于：依次包括如下步骤：①、采用水蒸汽蒸馏的方法，提取出当归、白术、薄荷三味中药的挥发油；②、提取挥发油后的药渣与白芍、茯苓、红花、皂角刺、竹叶柴胡、甘草一起以水为溶剂，用超声波提取得水提取物；③、将提取的挥发油用 β－环糊精包结，即得挥发油 β－环糊精包结物；④、将步骤②所得水提取物和步骤③所得挥发油 β－环糊精包结物加入淀粉，制粒压制成片，包薄膜衣，即得。

2. 根据权利要求 1 所述的一种红花逍遥片的制备方法，其特征在于：所述原料药的重量为：当归 260g、白芍 260g、白术 260g、茯苓 260g、红花 50g、皂角刺 80g、竹叶柴胡 260g、薄荷 40g、甘草 195g。

3. 根据权利要求 1 所述的一种红花逍遥片的制备方法，其特征在于：所述步骤①中的水蒸汽蒸馏方法是指先将当归、白术、薄荷三味药材粉碎，在水中浸泡 1.5-3 小时，然后用挥发油蒸馏器进行水内蒸馏，直至油水分离器挥发油不再增加。

4. 根据权利要求 1 或 3 所述的一种红花逍遥片的制备方法，其特征在于：所述步骤①中当归、白术、薄荷三味药材的出油率为 0.7-0.9ml/100g。

5. 根据权利要求 1 所述的一种红花逍遥片的制备方法，其特征在于：所述步骤②中的超声波提取是指将提取挥发油后的药渣与白芍、茯苓、红花、皂角刺、竹叶柴胡、甘草一起以 8-12 倍量水为溶剂，用超声波提取器提取 2-3 次，每次 30-50min，之后过滤，水提取液经减压浓缩，干燥后得水提取物。

6. 根据权利要求 5 所述的一种红花逍遥片的制备方法，其特征在于：所述步骤②中加入的水的重量包括步骤①中蒸馏过药材后的蒸煮水。

7. 根据权利要求 1 所述的一种红花逍遥片的制备方法，其特征在于：所述步骤③中的将提取的挥发油用 β－环糊精包结是指在挥发油体积：β－环糊精质量为 0.5-1.5:4-8 的比例条件下，采用饱和水溶液法，在 $30^{0}C$-$50^{0}C$ 下搅拌包结 20-40min，而后低温沉淀，抽滤烘干即得，所述挥发油体积单位为：毫升；所述 β－环糊精质量单位为：克。

8. 根据权利要求 7 所述的一种红花逍遥片的制备方法，其特征在于：所述挥发油体积：β－环糊精质量为 1:5，包结反应温度为 40 ^{0}C，包结时间为 30min。

9. 根据权利要求 1 所述的一种红花逍遥片的制备方法，其特征在于：所述挥发油 β－环糊精包结物与水提取物也可用于制备红花逍遥颗粒剂、红花逍遥胶囊剂等制剂。

一种红花逍遥片的制备方法

技术领域

[0001]　本发明涉及一种中药制剂的制备方法，具体涉及一种红花逍遥片的制备方法。

背景技术

[0002]　红花逍遥片是江西普正制药有限公司 2005 年以来开发的第九类新药，该药处方由当归、白芍、白术、茯苓、红花、皂角刺、竹叶柴胡、薄荷、甘草九味药组成。功能主治为舒肝、理气、活血，用于肝气不舒，胸胁胀痛，头晕目眩，食欲减退，月经不调，乳房胀痛或伴见颜面黄褐斑。

[0003]　红花逍遥片的传统制备方法是依据“红花逍遥胶囊”处方和生产工艺，经过简单改变剂型而来，方法为：在上述九味药中，取当归、白术、薄荷水蒸气蒸馏提取挥发油，挥发油备用；药渣与其余六味药材加水煎煮二次，每次 2 小时，分次滤过，合并滤液并浓缩至相对密度为 1.40（$50^{0}C$）的稠膏，干燥，粉碎；加入淀粉、硬脂酸镁适量，混匀，用 95% 乙醇制成颗粒，喷入上述挥发油，密闭 12 小时，压制成片，包薄膜衣，即得。该制备方法采用热回流法提取药材中的有效成分，很容易因煎煮而破坏遇热不稳定的有效成份，提取率不高，煎煮所用的时间较长，耗费能源；该制备方法中挥发油的加入是把挥发油直接喷在颗粒上，而后压片，这样做挥发油的自然逸散速度快，具有药理作用的挥发油在制剂中保留指数不高。

发明内容

[0004]　本发明的目的是针对上面所述缺陷，提供一种药材有效成份提取率高，药物中挥发油保留率高的红花逍遥片的制备方法。

[0005]　本发明的目的是通过以下技术方案予以实现的：

一种红花逍遥片的制备方法，包括下列重量份的原料药：当归 220g-300g、白芍 220g-300g、白术 220g-300g、茯苓 220g-300g、红花 30g-70g、皂角刺 60g-100g、竹叶柴胡 220g-300g、薄荷 20g-60g、甘草 160g-240g，依次包括如下步骤：①、采用水蒸汽蒸馏的方法，提取出当归、白术、薄荷三味中药的挥发油；②、提取挥发油后的药渣与白芍、茯苓、红花、皂角刺、竹叶柴胡、甘草一起以水为溶剂，用超声波提取得水提取物；③、将提取的挥发油用 β－环糊精包结，即得挥发油 β－环糊精包结物；④、将步骤②所得水提取物和步骤③所得挥发油 β－环糊精包结物加入淀粉，制粒压制成片，包薄膜衣，即得。

[0006]　所述原料药的重量优选为：当归 260g、白芍 260g、白术 260g、茯苓 260g、红花 50g、皂角刺 80g、竹叶柴胡 260g、薄荷 40g、甘草 195g。

[0007]　所述步骤①中的水蒸汽蒸馏方法是指先将当归、白术、薄荷三味药材粉碎，在水中浸泡 1.5-3 小时，然后用挥发油蒸馏器进行水内蒸馏，直至油水分离器挥发油不再增加。

[0008]　所述步骤①中当归、白术、薄荷三味药材的出油率为 0.7-0.9ml/100g。

[0009]　所述步骤②中的超声波提取是指将提取挥发油后的药渣与白芍、茯苓、红花、皂角刺、竹叶柴胡、甘草一起以 8-12 倍量水为溶剂，用超声波提取器提取 2-3 次，每次 30-50min，之后过滤，水提取液经减压浓缩，干燥后得水提取物。

[0010]　所述步骤②中加入的水的重量包括步骤①中蒸馏过药材后的蒸煮水。

[0011]　所述步骤③中的将提取的挥发油用 β-环糊精包结是指在挥发油体积：β-环糊精质量为 0.5-1.5:4-8 的比例条件下，采用饱和水溶液法，在 30^0C-50^0C 下搅拌包结 20-40min，而后低温沉淀，抽滤烘干即得。

[0012]　所述挥发油体积：β-环糊精质量优选为 1:5，包结反应温度优选为 40 ^{0C}，包结时间优选为 30min。

[0013]　所述挥发油体积单位为：毫升；所述 β-环糊精质量单位为：克。

[0014]　所述挥发油 β-环糊精包结物与水提取物也可用于制备红花逍遥颗粒剂、红花逍遥胶囊剂等制剂。

[0015]　本发明采用 $L_9(3^4)$ 正交设计表安排实验，以挥发油利用率为指标，对 3 个因素进行考察，优选出 β-环糊精包结挥发油的最佳工艺。

[0016]　① 考察因素水平：影响包合物制备的主要因素为：A 为挥发油：β-环糊精 (ml/g)，B 为温度 (^{0}C)，C 为包结时间 (min)，因素水平见表 1。

[0017]

表 1 因素水平表

水平	因素		
	A(ml:g)	B (℃)	C(min)
1	1:5	40	30
2	1:6	50	75
3	1:7	60	120

② 评价指标：包结率(即挥发油利用率)是衡量包合效果的重要指标，包合率越高，包合效果越好，故可作为工艺筛选的主要评价指标。但包结物收得率在大生产中也很有意义，在 β-环糊精和挥发油投入量一定的情况下，包结物收得率越高，包合效果越好，故包结物收得率可选为次要评价指标。

[0018]　③ 制备方法：取 β-环糊精精密称定，置烧杯中，加入水使其成饱和溶液，置于水浴上保温搅拌，一定时间内缓慢滴加挥发油。包结完后，置冰箱中冷藏 24h，倾去上层清液，滤出沉淀，用少量水洗涤，抽滤干，再用少量乙醇洗涤，抽滤干，40^0C 真空干燥，即得包合物。

[0019]　④ β-环糊精挥发油包合物中挥发油的测定：方法参见 2010 年版《中华人民共和国药典》(一部)附录Ⅹ D 挥发油测定方法甲法。

[0020]　⑤ 实验安排及结果分析：本实验选用正交设计，根据因素水平表选用 $L_9(3^4)$ 正交安排实验，以挥发油包结率、包结物收得率为指标综合评分为考察指标，进行数据处理，见表 2。

[0021]　挥发油包结率计算方法如下式：

$$\text{包结率\%} = \frac{\text{包结物中挥发油含量 (ml)}}{\text{挥发油加入量 (ml)}} \times 100\%$$

包结物收得率计算方法如下式：

$$包结物收得率\%== \frac{包结物重量（g）}{\beta\text{-CD 重量（g）+ 挥发油加入量（ml）}} x100\%$$

表 2　结果与分析

实验号	因素				水平		
	A(ml:g)	B(℃)	C(min)	D(误差)	包结率（%）	收率（%）	综合评分
1	1:5	40	30	1	78.58	95.543	83.37
2	1:5	50	75	2	71.42	91.02	77.31
3	1:5	60	120	3	72.87	90.76	78.23
4	1:6	40	75	3	65.01	94.71	73.93
5	1:6	50	120	1	62.01	90.23	70.48
6	1:6	60	30	2	51.02	85.56	61.36
7	1:7	40	120	2	73.09	92.99	79.06
8	1:7	50	30	3	57.70	93.68	68.48
9	1:7	60	75	1	41.66	83.68	57.42
I_j	238.89	236.32	213.22	211.24			
II_j	205.75	216.26	208.02	217.72			
III_j	204.95	197.01	227.75	220.63			
I_j	79.63	78.77	71.07	70.41		G=649.59	
II_j	68.59	72.09	69.54	72.57			
III_j	68.32	65.67	75.92	73.54		CT=46 885.24	
R_j	11.31	13.10	6.38	3.13			
I_j^2	57068.43	55847.14	45462.77	44622.34			
II_j^2	42333.06	46768.39	43522.30	47402.00			
III_j^2	42004.50	38812.94	51870.06	48677.60			
$R_j^2=I_j^2+II_j^2+III_j^2$							
	141406.00	141428.47	140855.14	140701.93			
$S_j=R_j^2/3-CT$							
	250.08	257.59	66.48	15.41			

由表 2 极差分析结果表明：各因素对试验结果的影响程度分别为 B>A>C，各因素的最佳水平为 $A_1B_1C_3$。对其进一步进行方差分析，其结果见表 3。

表 3 实验方差分析表

方差来源	离差平方和	自由度	F	P
A	250.08	2	16.23	<0.1
B	257.59	2	16.74	<0.1
C	66.48	2	4.33	>0.1

注：$F_{1-0.05}(2,2)=19.0$； $F_{1-0.10}(2,2)=9.0$

[0022] 方差分析结果表明：各因素对试验结果的影响程度依次为 B>A>C，其中 A、B 两因素对试验结果在统计学上有影响（P<0.1）；C 因素对试验结果无统计学意义（P>0.1）。其中 A_1 优于 A_2 和 A_3；B_1 优于 B_2 和 B_3；C 因素对试验结果无显著影响，从节省时间和成本考虑，选择 C_1。

[0023] ⑥ 验证试验：综合极差分析与方差分析结果，最佳包合工艺确定为 $A_1B_1C_1$。

[0024] 按最佳包结工艺，制备 3 批 β-环糊精挥发油包合物，测定其包结率和包结物收得率，并且计算综合评分，结果见表 4。

表 4 验证试验结果

试验号	包结率%	包结物收得率%	评 分
1	82.86	90.93	86.90
2	80.00	88.96	84.48
3	81.57	91.47	86.52

[0025] 本发明采用正交设计对红花逍遥片制备工艺中挥发油进行了 β-环糊精包结工艺研究，结果可知，挥发油与 β-环糊精的比例为 1 ∶ 5(ml/g)、包结反应温度为 40℃、包结时间为 30min 时挥发油的包合率最佳。

[0026] 本发明的有益效果是：本发明采用超声波提取方法，能够使药材细胞内的有效成份迅速溶出，且不破坏有效成份的化学结构，时间短，提取率高。在挥发油加入方法上采用 β-环糊精包结技术，使药物中的挥发部分被包结在 β-环糊精的空腔中，从而切断了不稳定成份与周围媒价接触，使药物分子得到保护，有效地防止了挥发，更避免了光、氧以及水解条件的影响，增加了稳定性，有效地增加中药挥发性成分在制剂中的保留指数。

具体实施方式

[0027] 实施例一：

取当归 260g、白术 260g 和薄荷 40g 粉碎过 40 目筛孔，加 6.72Kg 水浸泡 2 小时，用水中蒸馏法以水蒸气蒸馏约 4 小时提取挥发油，直至油水分离器中挥发油不再增加，分离出含水挥发油，用无水硫酸钠干燥，得挥发油 3.64ml，备用。取 β-环糊精 18.2g，制成饱和水溶液，温度保持在 40℃，搅拌下滴加上述挥发油，30min 后停止搅拌，把反应物放凉并移入冷

藏箱，在 3°C 放置 24 小时使包结物沉淀。后排去上部清液(留着下次包结用)，吸滤干得沉淀物，40°C 低温真空干燥，得到 19.5g 挥发油 β－环糊精包结物。

[0028] 取白芍 360g、茯苓 360g、红花 50g、皂角刺 80g、竹叶柴胡 260g、甘草 195g 粉碎过 40 目筛孔与提取挥发油后的药渣一起用 10.2Kg 水(包括蒸馏挥发油的残留水)浸泡 60min。然后在超声波频率 40kHz 下，提取 40min，过滤。滤渣再加入 8.3Kg 水在超声波频率 40kHz 下，重复提取 40min，过滤并合并两次滤液，水提取液经减压浓缩后得水提物浸膏。以上浸膏在 0.85Mpa，60°C 真空烘干到含水量 5% 以下。得提取物干膏 156.7g，粉碎到 80 目，备用。

[0029] 取提取物干膏 156.7g、挥发油 β－环状糊精包结物 19.5g、加适量辅料药用淀粉使其总量为 368g，用递增法混合均匀，用 95% 乙醇制粒，烘干，整粒，加入硬脂酸镁 4g 拌匀，压片，制成 1000 片，包薄膜衣，即得。

[0030] 实施例二：

取当归 260g、白术 260g 和薄荷 40g 粉碎过 40 目筛孔，加 7.74Kg 水浸泡 2 小时，用水中蒸馏法以水蒸气蒸馏约 4 小时提取挥发油，直至油水分离器中挥发油不再增加，分离出含水挥发油油，用无水硫酸钠干燥，得挥发油 4.07ml，备用。取 β－环状糊精 20.4g，制成饱和水溶液，温度保持在 40℃，搅拌下滴加上述挥发油，30 min 后停止搅拌，把反应物放凉并移入冷藏箱，在 2—4℃放置 24 小时使包结物沉淀。后排去上部清液(留着下次包结用)，吸滤干得沉淀物，40℃低温真空干燥，得到 23.8g 挥发油 β－环状糊精包结物。

[0031] 取白芍 260g、茯苓 260g 、红花 50g 、皂角刺 80g、竹叶柴胡 260g 、甘草 195g 粉碎过 40 目筛孔与提取挥发油后的药渣一起用 10.3Kg 水(包括蒸馏挥发油的残留水)浸泡 60 min 。然后在超声波频率 40kHz 下，提取 40min，过滤。滤渣再加入 8.4Kg 水在超声波频率 40kHz 下，重复提取 20min，过滤并合并两次滤液，水提取液经减压浓缩后得水提物浸膏。以上浸膏在 0.85MPa，60℃真空烘干到含水量 5% 以下。得提取物干膏 156.2g，粉碎到 80 目，备用。

[0032] 取提取物干膏 156.2g、挥发油 β－环状糊精包结物 23.8g、加适量辅料药用淀粉使其总量为 389g，用递增法混合均匀，用 95% 乙醇制粒，烘干，整粒，加入硬脂酸镁 4g 拌匀，压片，制成 1000 片，包薄膜衣，即得。

实施例三：

取当归 520g、白术 520g 和薄荷 80g 粉碎过 40 目筛孔，加 13.44Kg 水浸泡 2 小时，用水中蒸馏法以水蒸气蒸馏约 4 小时提取挥发油，直至油水分离器中挥发油不再增加，分离出含水挥发油油，用无水硫酸钠干燥，得挥发油 7.28ml，备用。取 β－环状糊精 36.4g，制成饱和水溶液，温度保持在 40℃，搅拌下滴加上述挥发油，30 min 后停止搅拌，把反应物放凉并移入冷藏箱，在 2—4℃放置 24 小时使包结物沉淀。后排去上部清液(留着下次包结用)，吸滤干得沉淀物，40℃低温真空干燥，得到 38.9g 挥发油 β－环状糊精包结物。

[0033] 取白芍 520g、茯苓 520g 、红花 100g 、皂角刺 160g、竹叶柴胡 520g 、甘草 390g 粉碎过 40 目筛孔与提取挥发油后的药渣一起用 20.4Kg 水(包括蒸馏挥发油的残留水)浸泡 60 min 。然后在超声波频率 40kHz 下，提取 40min，过滤。滤渣再加入 16.6Kg 水在超声波频率 40kHz 下，重复提取 20min，过滤并合并两次滤液，水提取液经减压浓缩后得水提物浸膏。以上浸膏在 0.85MPa，60℃真空烘干到含水量 5% 以下。得提取物干膏 311.4g，粉碎到 80 目，备用。

[0034] 取提取物干膏 311.4g、挥发油 β-环状糊精包结物 38.9g、加适量辅料药用淀粉使其总量为 736g，用递增法混合均匀，用 95% 乙醇制粒，烘干，整粒，加入硬脂酸镁 8g 拌匀，压片，制成 2000 片，包薄膜衣，即得。

[0035] 实施例四：

取当归 780g、白术 780g 和薄荷 120g 粉碎过 40 目筛孔，加 21.2Kg 水浸泡 2 小时，用水中蒸馏法以水蒸气蒸馏约 4 小时提取挥发油，直至油水分离器中挥发油不再增加，分离出含水挥发油油，用无水硫酸钠干燥，得挥发油 11.82ml，备用。取 β-环状糊精 59.1g，制成饱和水溶液，温度保持在 40℃，搅拌下滴加上述挥发油，30 min 后停止搅拌，把反应物放凉并移入冷藏箱，在 2—4℃放置 24 小时使包结物沉淀。后排去上部清液(留着下次包结用)，吸滤干得沉淀物，40℃低温真空干燥，得到 69.8g 挥发油 β-环状糊精包结物。

[0036] 取白芍 780g、茯苓 780g 、红花 150g 、皂角刺 240g、竹叶柴胡 780g 、甘草 585g 粉碎过 40 目筛孔与提取挥发油后的药渣一起用 30.83Kg 水(包括蒸馏挥发油的残留水)浸泡 60 min 。然后在超声波频率 40kHz 下，提取 40min，过滤。滤渣再加入 25.4Kg 水在超声波频率 40kHz 下，重复提取 20min，过滤并合并两次滤液，水提取液经减压浓缩后得水提物浸膏。以上浸膏在 0.85MPa，60℃真空烘干到含水量 5% 以下。得提取物干膏 466.9g，粉碎到 80 目，备用。

[0037] 取提取物干膏 466.9g、挥发油 β-环状糊精包结物 69.8g、加适量辅料药用淀粉使其总量为 867g，用递增法混合均匀，用 95% 乙醇制粒，烘干，整粒，加入硬脂酸镁 12g 拌匀，压片，制成 3000 片，包薄膜衣，即得。

取实施例一所得片剂与原工艺制备的红花逍遥片每份 200g 做恒温加速试验(温度 40±2℃)，以实验前与实验后品样品中挥发油含量为指标，考察恒温加速试验中，样品中挥发油的保留率，结果列于表 5。挥发油保留率计算方法：

$$挥发油保留率 = \frac{实验后品样品中挥发油的含量(ml)}{实验品前样品中挥发油的含量(ml)} \times 100\%$$

表 5 样品中挥发油保留率

项 目	0 天后	60 天后	120 天后	180 天后
原工艺样品<br>挥发油保留率(%)	100	72.86	40.93	30.20
新工艺样品<br>挥发油保留率(%)	100	91.27	86.47	71.26

6 个月后，新工艺制备的产品挥发油保留率为在 71.2% 以上，原工艺制剂其保留率仅在 30% 左右。
