# Supplementary material for: Efficacy of Xiaoyao-san preparations in treating Hashimoto’s thyroiditis: a meta-analysis and systematic review
Source: Front Pharmacol. 2025 Jun 13;16:1528506. doi: 10.3389/fphar.2025.1528506 (PMC12202410; doi:10.3389/fphar.2025.1528506)
Supplement: Supplementary file 2 [file Supplementaryfile2.zip › Supplementary Files 2/Quality Control Method for Honghua Xiaoyao Tablets - Invention Patent Application Specification CN201810915952..pdf]

(19)中华人民共和国国家知识产权局

(12)发明专利申请

(10)申请公布号 CN 108982740 A
(43)申请公布日 2018.12.11

(21)申请号 201810915952.4

(22)申请日 2018.08.13

(71)申请人 江西普正制药有限公司
地址 343799 江西省吉安市井冈山经济技术开发区吉安大道5号

(72)发明人 李小锋　杨明　康兴东　舒积成
高映　杜剑松　张金良

(74)专利代理机构 北京精金石知识产权代理有限公司 11470
代理人 张黎

(51)Int.Cl.
G01N 30/90(2006.01)

权利要求书3页　说明书6页　附图2页

(54)发明名称

一种红花逍遥片的质量控制方法

(57)摘要

本发明涉及一种红花逍遥片的质量控制方法,属于中药质量控制技术领域。本发明采用薄层色谱方法对方中的红花、白术及竹叶柴胡中的kaerophyllin进行定性鉴别,实现了对药品质量的有效控制。试验证明,该方法准确度、灵敏度高,检测范围广,可作为红花逍遥片质量检测的常规方法。

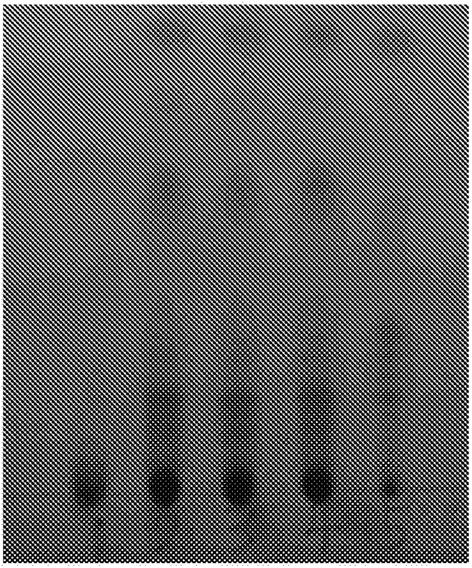

CN 108982740 A

1.一种红花逍遥片的质量控制方法,红花逍遥片包括如下重量份的原料药:当归130-390份,白芍130-390份,白术130-390份,茯苓130-390份,红花20-80份,皂角刺40-120份,竹叶柴胡130-390份,薄荷20-60份,甘草100-300份。其特征在于:所述质量检测方法包括如下薄层鉴别方法中的一种或几种:

A.红花的薄层色谱鉴别

取红花逍遥片10-20片,研碎,置于锥形瓶中,加入30-50ml甲醇,超声提取20-40min,离心,取上清液,蒸干,用10-30ml双蒸水溶解,用乙酸乙酯萃取两次,每次10-30ml,合并乙酸乙酯层,用1%氢氧化钠溶液萃取两次,每次10-30ml,合并乙酸乙酯液,蒸干,用0.5-2ml乙酸乙酯溶解,作为供试品溶液。

另取红花0.5-2g,加入10-30ml甲醇,超声20-40min,过滤,滤液蒸干,用10-30ml双蒸水溶解,用乙酸乙酯萃取两次,每次10-30ml,合并乙酸乙酯层,用1%氢氧化钠溶液萃取两次,每次10-30ml,合并乙酸乙酯液,蒸干,用0.5-2ml乙酸乙酯溶解,作为对照品溶液。

按照所述红花逍遥片的原料药处方配制不含红花的阴性样品,另取缺红花的阴性样品10-20片,按照所述供试品溶液的制备同法制成阴性对照品溶液。

照薄层色谱法试验,吸取供试品、阴性对照品溶液和对照药材溶液各3-7μl,分别点于同一以羟甲基纤维素钠为黏合剂的硅胶G薄层板上,以体积比为3-7:0.2-0.6:0.1-0.2的环己烷-乙酸乙酯-冰醋酸为展开剂,展开,取出,晾干,均匀喷洒10%硫酸乙醇溶液,加热至斑点显色清晰。

B.白术的薄层色谱鉴别

取红花逍遥片10-30片,研碎,置于锥形瓶中,加入40-80ml水,超声提取20-40min,离心,取上清液,用石油醚萃取两次,每次20-40ml,合并石油醚层,蒸干,用0.5-2ml甲醇溶解,作为供试品溶液。

另取白术药材0.5-2g,加入10-30ml水,回流提取20-40min,离心,取上清液,用石油醚萃取两次,每次20-40ml,合并石油醚层,蒸干,用0.5-2ml甲醇溶解,作为对照药材溶液。

按照所述红花逍遥片的原料药处方配制不含白术的阴性样品,另取缺白术的阴性样品10-30片,按照所述供试品溶液的制备同法制成阴性对照品溶液。

照薄层色谱法试验,吸取供试品、阴性对照品溶液和对照药材溶液各3-7μl,分别点于同一硅胶G薄层板上,以体积比为3-7:0.2-0.6:0.1-0.2的环己烷-乙酸乙酯-冰醋酸为展开剂,展开,取出,晾干,均匀喷洒10%硫酸乙醇显色剂,加热至斑点显色清晰。

C.kaerophyllin的薄层色谱鉴别

取红花逍遥片10-20片,研碎,置于锥形瓶中,加入30-50ml甲醇,超声提取20-40min,离心,取上清液,蒸干,残渣用体积比0.5-2:0.5-2:0.5-2的石油醚-甲醇-水萃取10-30ml,取甲醇-水层,蒸干,再用体积比0.5-2:0.5-2的正己烷-水萃取10-30ml,取正己烷部位,蒸干,残渣用0.5-2ml甲醇溶解,作为供试品溶液。

取竹叶柴胡药材0.5-2g,加入10-30ml甲醇,超声提取20-40min,过滤,蒸干,残渣用体积比0.5-2:0.5-2:0.5-2的石油醚-甲醇-水萃取10-30ml,取甲醇-水层,蒸干,再用体积比0.5-2:0.5-2的正己烷-水萃取10-30ml,取正己烷部位,蒸干,残渣用0.5-2ml甲醇溶解,作为对照药材溶液。

另取kaerophyllin,加甲醇制成0.4mg/ml的对照品溶液。

照薄层色谱法试验，吸取供试品、对照品溶液和对照药材溶液各3-7μl，分别点于同一硅胶GF254薄层板上，以体积比3-6:1-3:0.05-0.2的石油醚-丙酮-氨水为展开剂，展开，取出，晾干，在254nm紫外光下检视。

2.根据权利要求1所述的一种竹叶柴胡的质量控制方法，其特征在于：所述质量检测方法包括如下薄层鉴别方法中的一种或几种：

A.红花的薄层色谱鉴别

取红花逍遥片15片，研碎，置于锥形瓶中，加入40ml甲醇，超声提取30min，2500转/min离心15min，取上清液，蒸干，用20ml双蒸水溶解，用乙酸乙酯萃取两次，每次20ml，合并乙酸乙酯层，用1％氢氧化钠溶液萃取两次，每次20ml，合并乙酸乙酯液，蒸干，用1ml乙酸乙酯溶解，作为供试品溶液。

另取红花1g，加入20ml甲醇，超声30min，过滤，滤液蒸干，用20ml双蒸水溶解，用乙酸乙酯萃取两次，每次20ml，合并乙酸乙酯层，用1％氢氧化钠溶液萃取两次，每次20ml，合并乙酸乙酯液，蒸干，用1ml乙酸乙酯溶解，作为对照品溶液。

按照所述红花逍遥片的原料药处方配制不含红花的阴性样品，另取缺红花的阴性样品15片，按照所述供试品溶液的制备同法制成阴性对照品溶液。

照薄层色谱法试验，吸取供试品、阴性对照品溶液和对照药材溶液各5μl，分别点于同一以羟甲基纤维素钠为黏合剂的硅胶G薄层板上，以体积比为5:0.4:0.15的环己烷-乙酸乙酯-冰醋酸为展开剂，展开，取出，晾干，均匀喷洒10％硫酸乙醇溶液，加热至斑点显色清晰。

B.白术的薄层色谱鉴别

取红花逍遥片20片，研碎，置于锥形瓶中，加入60ml水，超声提取30min，2500转/min离心15min，取上清液，用石油醚萃取两次，每次30ml，合并石油醚层，蒸干，用1ml甲醇溶解，作为供试品溶液。

另取白术药材1g，加入20ml水，回流提取30min，2500转/min离心15min，取上清液，用石油醚萃取两次，每次30ml，合并石油醚层，蒸干，用1ml甲醇溶解，作为对照药材溶液。

按照所述红花逍遥片的原料药处方配制不含白术的阴性样品，另取缺白术的阴性样品20片，按照所述供试品溶液的制备同法制成阴性对照品溶液。

照薄层色谱法试验，吸取供试品、阴性对照品溶液和对照药材溶液各5μl，分别点于同一硅胶G薄层板上，以体积比为5:0.4:0.15的环己烷-乙酸乙酯-冰醋酸为展开剂，展开，取出，晾干，均匀喷洒10％硫酸乙醇显色剂，加热至斑点显色清晰。

C.kaerophyllin的薄层色谱鉴别

取红花逍遥片15片，研碎，置于锥形瓶中，加入40ml甲醇，超声提取30min，2500转/min离心15min，取上清液，蒸干，残渣用体积比1:1:1的石油醚-甲醇-水萃取20ml，取甲醇-水层，蒸干，再用正己烷:水＝1:1萃取，各20ml，取正己烷部位，蒸干，残渣用1ml甲醇溶解，作为供试品溶液。

取竹叶柴胡药材1g，加入20ml甲醇，超声提取30min，过滤，蒸干，残渣用体积比1:1:1的石油醚-甲醇-水萃取20ml，取甲醇-水层，蒸干，再用体积比1:1的正己烷-水萃取20ml，取正己烷部位，蒸干，残渣用1ml甲醇溶解，作为对照药材溶液。

另取kaerophyllin适量，加甲醇制成0.4mg/ml的对照品溶液。

照薄层色谱法试验，吸取供试品、对照品溶液和对照药材溶液各5μl，分别点于同一硅

胶GF254薄层板上，以体积比4:2:0.1的石油醚-丙酮-氨水为展开剂，展开，取出，晾干，在254nm紫外光下检视。

一种红花逍遥片的质量控制方法

技术领域：

[0001] 本发明涉及一种红花逍遥片的质量控制方法，属于中药质量控制技术领域。

背景技术：

[0002] 红花逍遥片是由当归、白术、薄荷、白芍、红花、茯苓、皂角刺、竹叶柴胡、甘草九味药组成的复方制剂。主要有舒肝、理气、活血的功效，临床上用于治疗肝气不舒、月经不调、黄褐斑、围绝经期综合征、多囊卵巢综合征等，具有良好的疗效。

[0003] 目前红花逍遥片的质量控制技术较少，包括当归药材、竹叶柴胡药材、芍药苷、甘草次酸、薄荷脑等薄层色谱鉴别方法以及芍药苷含量测定方法。现有质量控制技术中，芍药苷薄层色谱鉴别方法和含量测定方法是为了对白芍药材进行质量控制；甘草次酸薄层色谱鉴别方法是对甘草药材进行质量控制；薄荷脑的薄层鉴别方法是对薄荷药材进行质量控制。因此，红花逍遥片中的红花药材、白术药材、茯苓药材、皂角刺药材均缺乏质量控制技术。

[0004] 现有红花逍遥片质量控制技术中，是通过竹叶柴胡药材的薄层色谱鉴别方法来对竹叶柴胡药材进行质量控制，但缺少对竹叶柴胡中特征性木脂素类成分的鉴别方法，对红花逍遥片的质量保障还存在不足。

发明内容：

[0005] 为此，本发明所要解决的技术问题在于提供一种红花逍遥片的质量控制方法，克服现有质量控制技术不全面、缺乏特征性的的缺陷，提升红花逍遥片的质量稳定性、可控性。

[0006] 为解决上述技术问题，本发明提供了一种红花逍遥片的质量控制方法，所述红花逍遥片包括如下重量份的原料药：当归130-390份，白芍130-390份，白术130-390份，茯苓130-390份，红花20-80份，皂角刺40-120份，竹叶柴胡130-390份，薄荷20-60份，甘草100-300份。

[0007] 所述质量检测方法包括如下薄层鉴别方法中的一种或几种：

[0008] A.红花的薄层色谱鉴别

[0009] 取红花逍遥片10-20片，研碎，置于锥形瓶中，加入30-50ml甲醇，超声提取20-40min，离心，取上清液，蒸干，用10-30ml双蒸水溶解，用乙酸乙酯萃取两次，每次10-30ml，合并乙酸乙酯层，用1%氢氧化钠溶液萃取两次，每次10-30ml，合并乙酸乙酯液，蒸干，用0.5-2ml乙酸乙酯溶解，作为供试品溶液。

[0010] 另取红花0.5-2g，加入10-30ml甲醇，超声20-40min，过滤，滤液蒸干，用10-30ml双蒸水溶解，用乙酸乙酯萃取两次，每次10-30ml，合并乙酸乙酯层，用1%氢氧化钠溶液萃取两次，每次10-30ml，合并乙酸乙酯液，蒸干，用0.5-2ml乙酸乙酯溶解，作为对照品溶液。

[0011] 按照所述红花逍遥片的原料药处方配制不含红花的阴性样品，另取缺红花的阴性样品10-20片，按照所述供试品溶液的制备同法制成阴性对照品溶液。

[0012] 照薄层色谱法试验，吸取供试品、阴性对照品溶液和对照药材溶液各3-7μl，分别点于同一以羟甲基纤维素钠为黏合剂的硅胶G薄层板上，以体积比为3-7:0.2-0.6:0.1-0.2的环已烷-乙酸乙酯-冰醋酸为展开剂，展开，取出，晾干，均匀喷洒10%硫酸乙醇溶液，加热至斑点显色清晰。

[0013] B.白术的薄层色谱鉴别

[0014] 取红花逍遥片10-30片，研碎，置于锥形瓶中，加入40-80ml水，超声提取20-40min，离心，取上清液，用石油醚萃取两次，每次20-40ml，合并石油醚层，蒸干，用0.5-2ml甲醇溶解，作为供试品溶液。

[0015] 另取白术药材0.5-2g，加入10-30ml水，回流提取20-40min，离心，取上清液，用石油醚萃取两次，每次20-40ml，合并石油醚层，蒸干，用0.5-2ml甲醇溶解，作为对照药材溶液。

[0016] 按照所述红花逍遥片的原料药处方配制不含白术的阴性样品，另取缺白术的阴性样品10-30片，按照所述供试品溶液的制备同法制成阴性对照品溶液。

[0017] 照薄层色谱法试验，吸取供试品、阴性对照品溶液和对照药材溶液各3-7μl，分别点于同一硅胶G薄层板上，以体积比为3-7:0.2-0.6:0.1-0.2的环已烷-乙酸乙酯-冰醋酸为展开剂，展开，取出，晾干，均匀喷洒10%硫酸乙醇显色剂，加热至斑点显色清晰。

[0018] C.kaerophyllin的薄层色谱鉴别

[0019] 取红花逍遥片10-20片，研碎，置于锥形瓶中，加入30-50ml甲醇，超声提取20-40min，离心，取上清液，蒸干，残渣用体积比0.5-2:0.5-2:0.5-2的石油醚-甲醇-水萃取10-30ml，取甲醇-水层，蒸干，再用体积比0.5-2:0.5-2的正已烷-水萃取10-30ml，取正已烷部位，蒸干，残渣用0.5-2ml甲醇溶解，作为供试品溶液。

[0020] 取竹叶柴胡药材0.5-2g，加入10-30ml甲醇，超声提取20-40min，过滤，蒸干，残渣用体积比0.5-2:0.5-2:0.5-2的石油醚-甲醇-水萃取10-30ml，取甲醇-水层，蒸干，再用体积比0.5-2:0.5-2的正已烷-水萃取10-30ml，取正已烷部位，蒸干，残渣用0.5-2ml甲醇溶解，作为对照药材溶液。

[0021] 另取kaerophyllin，加甲醇制成0.4mg/ml的对照品溶液。

[0022] 照薄层色谱法试验，吸取供试品、对照品溶液和对照药材溶液各3-7μl，分别点于同一硅胶GF254薄层板上，以体积比3-6:1-3:0.05-0.2的石油醚-丙酮-氨水为展开剂，展开，取出，晾干，在254nm紫外光下检视。

[0023] 优选的，所述质量检测方法包括如下薄层鉴别方法中的一种或几种：

[0024] A.红花的薄层色谱鉴别

[0025] 取红花逍遥片15片，研碎，置于锥形瓶中，加入40ml甲醇，超声提取30min，2500转/min离心15min，取上清液，蒸干，用20ml双蒸水溶解，用乙酸乙酯萃取两次，每次20ml，合并乙酸乙酯层，用1%氢氧化钠溶液萃取两次，每次20ml，合并乙酸乙酯液，蒸干，用1ml乙酸乙酯溶解，作为供试品溶液。

[0026] 另取红花1g，加入20ml甲醇，超声30min，过滤，滤液蒸干，用20ml双蒸水溶解，用乙酸乙酯萃取两次，每次20ml，合并乙酸乙酯层，用1%氢氧化钠溶液萃取两次，每次20ml，合并乙酸乙酯液，蒸干，用1ml乙酸乙酯溶解，作为对照品溶液。

[0027] 按照所述红花逍遥片的原料药处方配制不含红花的阴性样品，另取缺红花的阴性

样品15片，按照所述供试品溶液的制备同法制成阴性对照品溶液。

[0028] 照薄层色谱法试验，吸取供试品、阴性对照品溶液和对照药材溶液各5μl，分别点于同一以羟甲基纤维素钠为黏合剂的硅胶G薄层板上，以体积比为5:0.4:0.15的环己烷-乙酸乙酯-冰醋酸为展开剂，展开，取出，晾干，均匀喷洒10％硫酸乙醇溶液，加热至斑点显色清晰。

[0029] B.白术的薄层色谱鉴别

[0030] 取红花逍遥片20片，研碎，置于锥形瓶中，加入60ml水，超声提取30min，2500转/min离心15min，取上清液，用石油醚萃取两次，每次30ml，合并石油醚层，蒸干，用1ml甲醇溶解，作为供试品溶液。

[0021] 另取白术药材1g，加入20ml水，回流提取30min，2500转/min离心15min，取上清液，用石油醚萃取两次，每次30ml，合并石油醚层，蒸干，用1ml甲醇溶解，作为对照药材溶液。

[0032] 按照所述红花逍遥片的原料药处方配制不含白术的阴性样品，另取缺白术的阴性样品20片，按照所述供试品溶液的制备同法制成阴性对照品溶液。

[0033] 照薄层色谱法试验，吸取供试品、阴性对照品溶液和对照药材溶液各5μl，分别点于同一硅胶G薄层板上，以体积比为5:0.4:0.15的环己烷-乙酸乙酯-冰醋酸为展开剂，展开，取出，晾干，均匀喷洒10％硫酸乙醇显色剂，加热至斑点显色清晰。

[0034] C.kaerophyllin的薄层色谱鉴别

[0035] 取红花逍遥片15片，研碎，置于锥形瓶中，加入40ml甲醇，超声提取30min，2500转/min离心15min，取上清液，蒸干，残渣用体积比1:1:1的石油醚-甲醇-水萃取20ml，取甲醇-水层，蒸干，再用正己烷:水＝1:1萃取，各20ml，取正己烷部位，蒸干，残渣用1ml甲醇溶解，作为供试品溶液。

[0036] 取竹叶柴胡药材1g，加入20ml甲醇，超声提取30min，过滤，蒸干，残渣用体积比1:1:1的石油醚-甲醇-水萃取20ml，取甲醇-水层，蒸干，再用体积比1:1的正己烷-水萃取20ml，取正己烷部位，蒸干，残渣用1ml甲醇溶解，作为对照药材溶液。

[0037] 另取kaerophyllin适量，加甲醇制成0.4mg/ml的对照品溶液。

[0038] 照薄层色谱法试验，吸取供试品、对照品溶液和对照药材溶液各5μl，分别点于同一硅胶GF254薄层板上，以体积比4:2:0.1的石油醚-丙酮-氨水为展开剂，展开，取出，晾干，在254nm紫外光下检视。

[0039] 本发明所述的质量检测方法采用薄层色谱方法对方中的红花、白术及竹叶柴胡中的kaerophyllin进行定性鉴别，实现了对药品质量的有效控制。试验证明，该方法准确度、灵敏度高，检测范围广，可作为红花逍遥片质量检测的常规方法。

附图说明

[0040] 为了使本发明的内容更容易被清楚的理解，下面根据本发明的具体实施例并结合附图，对本发明作进一步详细的说明，其中

[0041] 图1是本发明实施例1中所述的红花逍遥片的红花薄层色谱图，1为阴性对照、2-4为供试品、5为红花对照药材；

[0042] 图2是本发明实施例4中所述的红花逍遥片的白术薄层色谱图，1为阴性对照、2-4为供试品、5为白术对照药材；

[0022] 图3是本发明实施例7中所述的红花逍遥片的kaerophyllin薄层色谱图，1为kaerophyllin对照品、2为竹叶柴胡对照药材、3-5为供试品；

具体实施方式

[0023] 为了更好的解释本发明的实施，提供下述测定方法的实施实例。这些实施实例仅仅是解释、而不是限制本发明的范围。下面通过典型的实施例对本发明做进一步的说明。

[0024] 实施例1:红花的薄层色谱鉴别

[0009] 取红花逍遥片15片，研碎，置于锥形瓶中，加入40ml甲醇，超声提取30min，2500转/min离心15min，取上清液，蒸干，用20ml双蒸水溶解，用乙酸乙酯萃取两次，每次20ml，合并乙酸乙酯层，用1％氢氧化钠溶液萃取两次，每次20ml，合并乙酸乙酯液，蒸干，用1ml乙酸乙酯溶解，作为供试品溶液。

[0010] 另取红花1g，加入20ml甲醇，超声30min，过滤，滤液蒸干，用20ml双蒸水溶解，用乙酸乙酯萃取两次，每次20ml，合并乙酸乙酯层，用1％氢氧化钠溶液萃取两次，每次20ml，合并乙酸乙酯液，蒸干，用1ml乙酸乙酯溶解，作为对照品溶液。

[0011] 按照所述红花逍遥片的原料药处方配制不含红花的阴性样品，另取缺红花的阴性样品15片，按照所述供试品溶液的制备同法制成阴性对照品溶液。

[0012] 照薄层色谱法试验，吸取供试品、阴性对照品溶液和对照药材溶液各5μl，分别点于同一以羟甲基纤维素钠为黏合剂的硅胶G薄层板上，以体积比为5:0.4:0.15的环已烷-乙酸乙酯-冰醋酸为展开剂，展开，取出，晾干，均匀喷洒10％硫酸乙醇溶液，加热至斑点显色清晰。

[0026] 实施例2:红花的薄层色谱鉴别

[0009] 取红花逍遥片20片，研碎，置于锥形瓶中，加入50ml甲醇，超声提取40min，离心，取上清液，蒸干，用30ml双蒸水溶解，用乙酸乙酯萃取两次，每次30ml，合并乙酸乙酯层，用1％氢氧化钠溶液萃取两次，每次30ml，合并乙酸乙酯液，蒸干，用2ml乙酸乙酯溶解，作为供试品溶液。

[0010] 另取红花2g，加入30ml甲醇，超声40min，过滤，滤液蒸干，用30ml双蒸水溶解，用乙酸乙酯萃取两次，每次30ml，合并乙酸乙酯层，用1％氢氧化钠溶液萃取两次，每次30ml，合并乙酸乙酯液，蒸干，用2ml乙酸乙酯溶解，作为对照品溶液。

[0011] 按照所述红花逍遥片的原料药处方配制不含红花的阴性样品，另取缺红花的阴性样品20片，按照所述供试品溶液的制备同法制成阴性对照品溶液。

[0012] 照薄层色谱法试验，吸取供试品、阴性对照品溶液和对照药材溶液各7μl，分别点于同一以羟甲基纤维素钠为黏合剂的硅胶G薄层板上，以体积比为7:0.6:0.2的环已烷-乙酸乙酯-冰醋酸为展开剂，展开，取出，晾干，均匀喷洒10％硫酸乙醇溶液，加热至斑点显色清晰。

[0028] 实施例3:红花的薄层色谱鉴别

[0009] 取红花逍遥片10片，研碎，置于锥形瓶中，加入30ml甲醇，超声提取20min，离心，取上清液，蒸干，用10ml双蒸水溶解，用乙酸乙酯萃取两次，每次10ml，合并乙酸乙酯层，用1％氢氧化钠溶液萃取两次，每次10ml，合并乙酸乙酯液，蒸干，用0.5ml乙酸乙酯溶解，作为供试品溶液。

[0010] 另取红花0.5g,加入10ml甲醇,超声20min,过滤,滤液蒸干,用10ml双蒸水溶解,用乙酸乙酯萃取两次,每次10ml,合并乙酸乙酯层,用1%氢氧化钠溶液萃取两次,每次10ml,合并乙酸乙酯液,蒸干,用0.5ml乙酸乙酯溶解,作为对照品溶液。

[0011] 按照所述红花逍遥片的原料药处方配制不含红花的阴性样品,另取缺红花的阴性样品10片,按照所述供试品溶液的制备同法制成阴性对照品溶液。

[0012] 照薄层色谱法试验,吸取供试品、阴性对照品溶液和对照药材溶液各3μl,分别点于同一以羟甲基纤维素钠为黏合剂的硅胶G薄层板上,以体积比为3:0.2:0.1的环己烷-乙酸乙酯-冰醋酸为展开剂,展开,取出,晾干,均匀喷洒10%硫酸乙醇溶液,加热至斑点显色清晰。

[0030] 实施例4:白术的薄层色谱鉴别

[0030] 取红花逍遥片20片,研碎,置于锥形瓶中,加入60ml水,超声提取30min,2500转/min离心15min,取上清液,用石油醚萃取两次,每次30ml,合并石油醚层,蒸干,用1ml甲醇溶解,作为供试品溶液。

[0021] 另取白术药材1g,加入20ml水,回流提取30min,2500转/min离心15min,取上清液,用石油醚萃取两次,每次30ml,合并石油醚层,蒸干,用1ml甲醇溶解,作为对照药材溶液。

[0032] 按照所述红花逍遥片的原料药处方配制不含白术的阴性样品,另取缺白术的阴性样品20片,按照所述供试品溶液的制备同法制成阴性对照品溶液。

[0033] 照薄层色谱法试验,吸取供试品、阴性对照品溶液和对照药材溶液各5μl,分别点于同一硅胶G薄层板上,以体积比为5:0.4:0.15的环己烷-乙酸乙酯-冰醋酸为展开剂,展开,取出,晾干,均匀喷洒10%硫酸乙醇显色剂,加热至斑点显色清晰。

[0035] 实施例5:白术的薄层色谱鉴别

[0014] 取红花逍遥片10片,研碎,置于锥形瓶中,加入40ml水,超声提取20min,离心,取上清液,用石油醚萃取两次,每次20ml,合并石油醚层,蒸干,用0.5ml甲醇溶解,作为供试品溶液。

[0015] 另取白术药材0.5g,加入10ml水,回流提取20min,离心,取上清液,用石油醚萃取两次,每次20ml,合并石油醚层,蒸干,用0.5ml甲醇溶解,作为对照药材溶液。

[0016] 按照所述红花逍遥片的原料药处方配制不含白术的阴性样品,另取缺白术的阴性样品10片,按照所述供试品溶液的制备同法制成阴性对照品溶液。

[0017] 照薄层色谱法试验,吸取供试品、阴性对照品溶液和对照药材溶液各3μl,分别点于同一硅胶G薄层板上,以体积比为3:0.2:0.1的环己烷-乙酸乙酯-冰醋酸为展开剂,展开,取出,晾干,均匀喷洒10%硫酸乙醇显色剂,加热至斑点显色清晰。

[0042] 实施例6:白术的薄层色谱鉴别

[0014] 取红花逍遥片30片,研碎,置于锥形瓶中,加入80ml水,超声提取40min,离心,取上清液,用石油醚萃取两次,每次40ml,合并石油醚层,蒸干,用2ml甲醇溶解,作为供试品溶液。

[0015] 另取白术药材2g,加入30ml水,回流提取40min,离心,取上清液,用石油醚萃取两次,每次40ml,合并石油醚层,蒸干,用2ml甲醇溶解,作为对照药材溶液。

[0016] 按照所述红花逍遥片的原料药处方配制不含白术的阴性样品,另取缺白术的阴性样品30片,按照所述供试品溶液的制备同法制成阴性对照品溶液。

[0017] 照薄层色谱法试验,吸取供试品、阴性对照品溶液和对照药材溶液各7μl,分别点于同一硅胶G薄层板上,以体积比为7:0.6:0.2的环己烷-乙酸乙酯-冰醋酸为展开剂,展开,取出,晾干,均匀喷洒10%硫酸乙醇显色剂,加热至斑点显色清晰。

[0042] 实施例7:kaerophyllin的薄层色谱鉴别

[0035] 取红花逍遥片15片,研碎,置于锥形瓶中,加入40ml甲醇,超声提取30min,2500转/min离心15min,取上清液,蒸干,残渣用体积比1:1:1的石油醚-甲醇-水萃取20ml,取甲醇-水层,蒸干,再用正己烷:水=1:1萃取,各20ml,取正己烷部位,蒸干,残渣用1ml甲醇溶解,作为供试品溶液。

[0036] 取竹叶柴胡药材1g,加入20ml甲醇,超声提取30min,过滤,蒸干,残渣用体积比1:1:1的石油醚-甲醇-水萃取20ml,取甲醇-水层,蒸干,再用体积比1:1的正己烷-水萃取20ml,取正己烷部位,蒸干,残渣用1ml甲醇溶解,作为对照药材溶液。

[0037] 另取kaerophyllin适量,加甲醇制成0.4mg/ml的对照品溶液。

[0038] 照薄层色谱法试验,吸取供试品、对照品溶液和对照药材溶液各5μl,分别点于同一硅胶GF254薄层板上,以体积比4:2:0.1的石油醚-丙酮-氨水为展开剂,展开,取出,晾干,在254nm紫外光下检视。

[0018] 实施例8:kaerophyllin的薄层色谱鉴别

[0019] 取红花逍遥片10片,研碎,置于锥形瓶中,加入50ml甲醇,超声提取20min,离心,取上清液,蒸干,残渣用体积比0.5:2:0.5的石油醚-甲醇-水萃取30ml,取甲醇-水层,蒸干,再用体积比2:0.5的正己烷-水萃取30ml,取正己烷部位,蒸干,残渣用0.5ml甲醇溶解,作为供试品溶液。

[0020] 取竹叶柴胡药材0.5g,加入30ml甲醇,超声提取20min,过滤,蒸干,残渣用体积比0.5:2:0.5的石油醚-甲醇-水萃取30ml,取甲醇-水层,蒸干,再用体积比2:0.5的正己烷-水萃取30ml,取正己烷部位,蒸干,残渣用0.5ml甲醇溶解,作为对照药材溶液。

[0021] 另取kaerophyllin,加甲醇制成0.4mg/ml的对照品溶液。

[0022] 照薄层色谱法试验,吸取供试品、对照品溶液和对照药材溶液各3μl,分别点于同一硅胶GF254薄层板上,以体积比3:3:0.2的石油醚-丙酮-氨水为展开剂,展开,取出,晾干,在254nm紫外光下检视。

[0018] 实施例9:kaerophyllin的薄层色谱鉴别

[0019] 取红花逍遥片20片,研碎,置于锥形瓶中,加入30ml甲醇,超声提取40min,离心,取上清液,蒸干,残渣用体积比2:0.5:2的石油醚-甲醇-水萃取10ml,取甲醇-水层,蒸干,再用体积比0.5:2的正己烷-水萃取10ml,取正己烷部位,蒸干,残渣用2ml甲醇溶解,作为供试品溶液。

[0020] 取竹叶柴胡药材2g,加入10ml甲醇,超声提取40min,过滤,蒸干,残渣用体积比2:0.5:2的石油醚-甲醇-水萃取10ml,取甲醇-水层,蒸干,再用体积比0.5:2的正己烷-水萃取10ml,取正己烷部位,蒸干,残渣用2ml甲醇溶解,作为对照药材溶液。

[0021] 另取kaerophyllin,加甲醇制成0.4mg/ml的对照品溶液。

[0022] 照薄层色谱法试验,吸取供试品、对照品溶液和对照药材溶液各7μl,分别点于同一硅胶GF254薄层板上,以体积比6:1:0.05的石油醚-丙酮-氨水为展开剂,展开,取出,晾干,在254nm紫外光下检视。

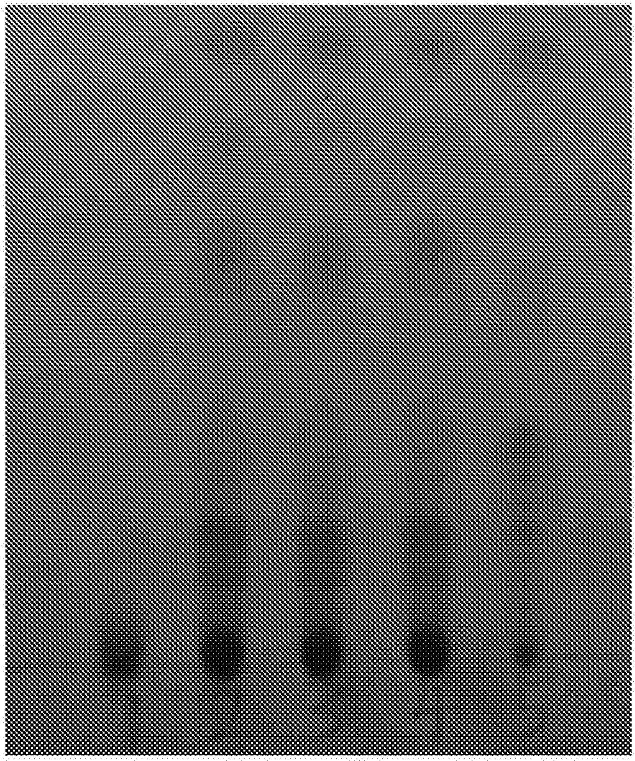

图1

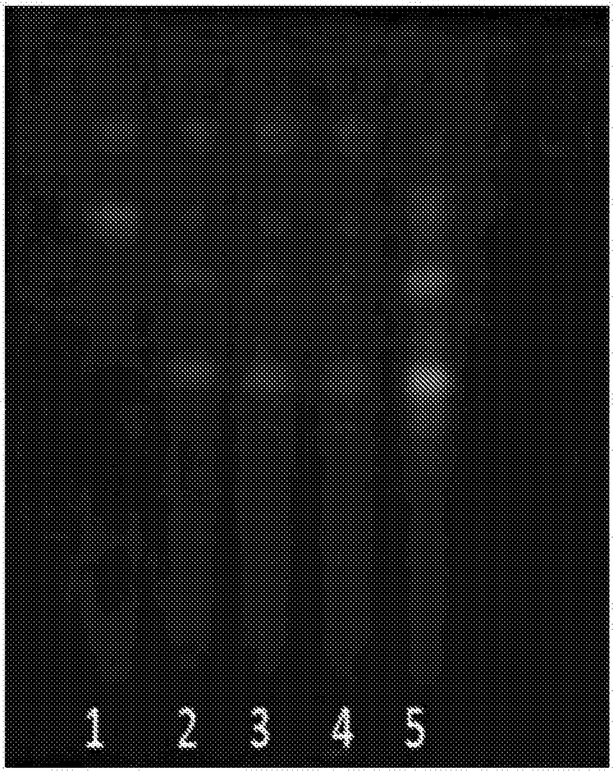

图2

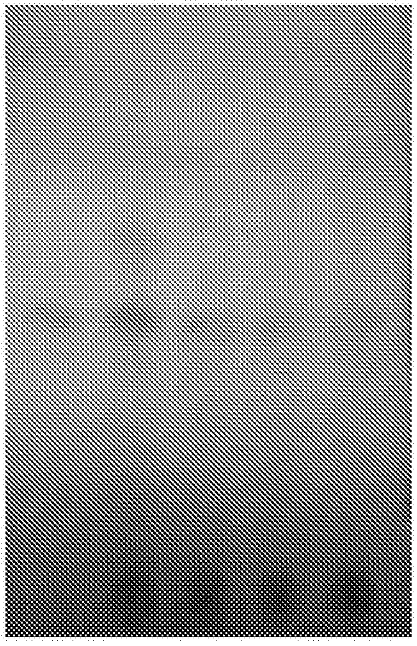

图3
