# Supplementary material for: Efficacy of Xiaoyao-san preparations in treating Hashimoto’s thyroiditis: a meta-analysis and systematic review
Source: Front Pharmacol. 2025 Jun 13;16:1528506. doi: 10.3389/fphar.2025.1528506 (PMC12202410; doi:10.3389/fphar.2025.1528506)
Supplement: Supplementary file 2 [file Supplementaryfile2.zip › Supplementary Files 2/Supplementary Files 2 formula granules section/Baizhu_YBZ-PFKL-2021161.pdf]

国家药品监督管理局
国家药品标准

YBZ-PFKL-2021161

白术配方颗粒

Baizhu Peifangkeli

【来源】 本品为菊科植物白术 *Atractylodes macrocephala* Koidz.的干燥根茎经炮制并按标准汤剂的主要质量指标加工制成的配方颗粒。

【制法】 取白术饮片 1300g，加水煎煮，滤过，滤液浓缩成清膏（干浸膏出膏率为 40%~74%），加入辅料适量，干燥（或干燥，粉碎），再加入辅料适量，混匀，制粒，制成 1000g，即得。

【性状】 本品为浅黄色至棕黄色的颗粒；气微，味淡、微甘。

【鉴别】 取本品 2g，研细，加水 20ml 使溶解，用乙酸乙酯振摇提取 2 次，每次 20ml，合并乙酸乙酯液，蒸干，残渣加甲醇 lml 使溶解，作为供试品溶液。另取白术对照药材 2g，加水 50ml，煮沸 30 分钟，滤过，滤液浓缩至约 20ml，同法制成对照药材溶液。照薄层色谱法（中国药典 2020 年版通则 0502）试验，吸取上述两种溶液各 10μl，分别点于同一高效硅胶 G 薄层板上，以环己烷-乙酸乙酯（1∶1）为展开剂，展开，取出，晾干，喷以 10％硫酸乙醇液，在 105℃加热至斑点显色清晰，置紫外光灯（365nm）下检视。供试品色谱中，在与对照药材色谱相应的位置上，显相同颜色的荧光斑点。

【特征图谱】 照高效液相色谱法（中国药典 2020 年版通则 0512）测定。

色谱条件与系统适用性试验 以十八烷基硅烷键合硅胶为填充剂（柱长为 100mm，内径为 2.1mm，粒径为 1.8μm）；以乙腈为流动相 A，以 0.1％磷酸溶液为流动相 B，按下表中的规定进行梯度洗脱；流速为每分钟 0.25ml；柱温为 25℃；检测波长为 235nm。理论板数按绿原酸峰计算应不低于 5000。

时间（min）	流动相 A（%）	流动相 B（%）
0～1	2	98
1～5	2→8	98→92
5～15	8→25	92→75
15～19	25→43	75→57
19～25	43→70	57→30
25～29	70→100	30→0

参照物溶液的制备 取白术对照药材 0.5g，置锥形瓶中，加水 5ml，超声处理 30 分钟，放冷，摇匀，滤过，取续滤液，作为对照药材参照物溶液。另取绿原酸对照品、白术内酯 III 对照品适量，加甲醇制成每 1ml 含绿原酸、白术内酯 III 各 50μg 的混合溶液，摇匀，作

为对照品参照物溶液。

供试品溶液的制备 取本品适量，研细，取 2g，置 10ml 量瓶中，加水适量，超声处理 20 分钟，取出，放冷，加水至刻度，摇匀，滤过，取续滤液，即得。

测定法 分别精密吸取参照物溶液与供试品溶液各 1μl，注入液相色谱仪，测定，即得。

供试品色谱中应呈现 4 个特征峰，并应与对照药材参照物色谱峰中的 4 个特征峰保留时间相对应，其中 2 个峰应分别与相应对照品参照物峰的保留时间相对应。与绿原酸参照物相应的峰为 S 峰，计算峰 1、3 与 S 峰的相对保留时间，其相对保留时间应在规定值的±10%范围之内；规定值为：0.82（峰 1）、1.04（峰 3）。

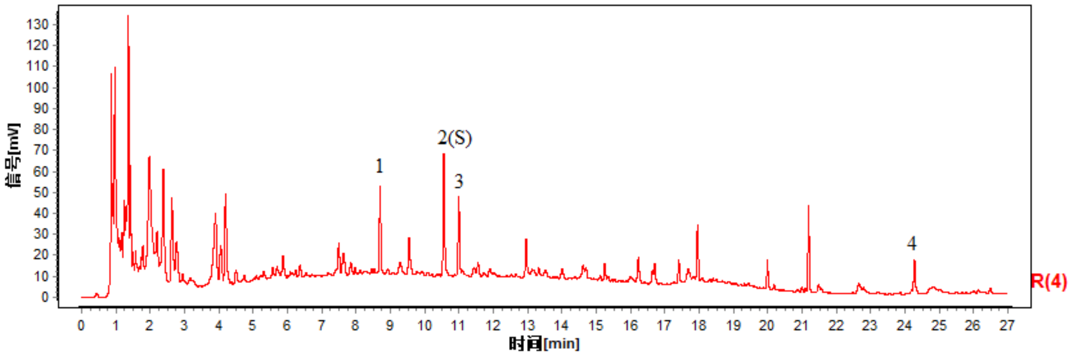

对照特征图谱

峰 1：新绿原酸；峰 2（S）：绿原酸；峰 3：隐绿原酸；峰 4：白术内酯III

色谱柱：HSS T3，100 mm×2.1mm，1.8μm

【检查】 应符合颗粒剂项下有关的各项规定（中国药典 2020 年版通则 0104）。

【浸出物】 照醇溶性浸出物测定法项下的热浸法（中国药典 2020 年版通则 2201）测定，用乙醇作溶剂，不得少于 5.0%。

【含量测定】 **新绿原酸、绿原酸、隐绿原酸** 照高效液相色谱法（中国药典 2020 年版通则 0512）测定。

色谱条件与系统适用性试验 以十八烷基硅胶键合硅胶为填充剂（柱长为 50mm，内径为 2.1mm，粒径为 1.7μm）；以乙腈为流动相 A，以 0.1％磷酸溶液为流动相 B，按下表中的规定进行梯度洗脱；流速为每分钟 0.25ml；柱温为 25℃；检测波长为 325nm。理论板数按绿原酸峰计算应不低于 5000。

时间（min）	流动相 A（%）	流动相 B（%）
0～4	1→6	99→94
4～10	6→9	94→91
10～16	9→18	91→82
16～22	18→20	82→80
22～23	20→90	80→10

对照品溶液的制备 取绿原酸对照品适量，精密称定，加水制成每 1ml 含 10μg 的溶液，摇匀，即得。

供试品溶液的制备 取本品适量，研细，取约 1g，精密称定，置具塞锥形瓶中，精密

加水15ml，密塞，称定重量，超声处理（功率250W，频率40kHz）30分钟，取出，放冷，再称定重量，用水补足减失的重量，摇匀，滤过，取续滤液，即得。

测定法　分别精密吸取对照品溶液与供试品溶液各2μl，注入液相色谱仪，测定。

以绿原酸对照品为参照，以其相应的峰为S峰，计算新绿原酸和隐绿原酸的相对保留时间，其相对保留时间应在规定值的±10%范围之内（若相对保留时间偏离超过±10%，则应以相应的被替代对照品确证为准）。相对保留时间及相对校正因子见下表：

待测成分（峰）	相对保留时间（RT）	相对校正因子（F）
新绿原酸	0.67	1.02
绿原酸（S）	1.00	1.00
隐绿原酸	1.14	1.00

以绿原酸的峰面积为对照，分别乘以校正因子，计算新绿原酸、隐绿原酸和绿原酸的含量。

本品每1克含新绿原酸（$C_{16}H_{18}O_9$）、绿原酸（$C_{16}H_{18}O_9$）和隐绿原酸（$C_{16}H_{18}O_9$）总量，应为0.12mg~0.95mg。

果糖、蔗糖　照高效液相色谱法（中国药典2020年版通则0512）测定。

色谱条件与系统适用性试验　以酰胺基键合硅胶为填充剂（柱长为100mm，内径为2.1mm，粒径为1.7μm）；以乙腈-0.2%三乙胺溶液（86:14）为流动相；流速为每分钟0.3ml；柱温为40℃；蒸发光散射检测器检测。理论板数按果糖峰计算应不低于1500。

对照品溶液的制备　取果糖对照品、蔗糖对照品适量，精密称定，加水制成每1ml含果糖0.4mg、蔗糖0.2mg的混合溶液，即得。

供试品溶液的制备　取本品适量，研细，取约0.1g，精密称定，置具塞锥形瓶中，精密加入70%甲醇溶液15ml，密塞，称定重量，超声处理（功率250W，频率40kHz）30分钟，放冷，再称定重量，用70%甲醇补足减失的重量，摇匀，滤过，取续滤液，即得。

测定法　分别精密吸取对照品溶液0.5μl、1.5μl，供试品溶液1μl，注入液相色谱仪，测定，用外标两点法对数方程计算，即得。

本品每1g含果糖（$C_6H_{12}O_6$）与蔗糖（$C_{12}H_{22}O_{11}$）的总量应为30.0mg~155.0mg。

（色谱柱：BEH Amide，2.1×100mm，1.7μm）

【规格】　每1g配方颗粒相当于饮片1.3g

【贮藏】　密封。
