# Supplementary material for: Efficacy of Xiaoyao-san preparations in treating Hashimoto’s thyroiditis: a meta-analysis and systematic review
Source: Front Pharmacol. 2025 Jun 13;16:1528506. doi: 10.3389/fphar.2025.1528506 (PMC12202410; doi:10.3389/fphar.2025.1528506)
Supplement: Supplementary file 2 [file Supplementaryfile2.zip › Supplementary Files 2/Supplementary Files 2 formula granules section/Banxia_JS-YBZ-2021082.pdf]

江苏省药品监督管理局
中药配方颗粒标准

JS-YBZ-2021082

清半夏配方颗粒

Qingbanxia　Peifangkeli

【来源】本品为天南星科植物半夏 *Pinellia ternata* (Thunb.) Breit.的炮制加工品按标准汤剂的主要质量指标加工制成的配方颗粒。

【制法】取清半夏饮片 3200g，加水煎煮，滤过，滤液浓缩成清膏（出膏率为 15~26%），加辅料适量，干燥（或干燥，粉碎），再加入辅料适量，混匀，制粒，制成 1000g，即得。

【性状】本品为白色至黄白色的颗粒；气微，味淡。

【鉴别】取本品 2g，研细，加甲醇 20ml，加热回流 30 分钟，滤过，滤液挥至 0.5ml，作为供试品溶液。另取半夏对照药材 1g，同法制成对照药材溶液。再取精氨酸、丙氨酸、缬氨酸、亮氨酸对照品适量，加 70%甲醇制成每 1ml 各含 1mg 的混合溶液，作为对照品溶液。照薄层色谱法（中国药典 通则 0502）试验，吸取供试品溶液、对照药材溶液 5μl、对照品溶液 1μl，分别点于同一硅胶 G 薄层板上，以正丁醇-冰醋酸-水（8∶3∶1）为展开剂，展开，取出，晾干，喷以茚三酮试液，在 105℃加热至斑点显色清晰。置日光下检视。供试品色谱中，在与对照药材色谱和对照品色谱相应的位置上，显相同颜色的斑点。

【特征图谱】照高效液相色谱法（中国药典 通则 0512）测定。

色谱条件与系统适用性试验　以十八烷基硅烷键合硅胶为填充剂；以乙腈为流动相 A，以 0.1%磷酸溶液为流动相 B，按下表梯度洗脱；流速为 0.3ml/min；柱温为 25℃；检测波长为 270nm。理论板数按 *L*-色氨酸峰计算应不低于 5000。

时间（分钟）	流动相 A（%）	流动相 B（%）
0	0	100
5	0	100
7	5	95
11	11	89
18	28	72
25	40	60

30	60	40
32	0	100

参照物溶液的制备 取半夏对照药材 2g，加入水 25ml，加热回流 1 小时，放冷，摇匀，滤过，取续滤液，作为对照药材参照物溶液。另取尿苷、鸟苷、*L*-色氨酸对照品适量，精密称定，加水制成每 lml 各含 50μg 的混合溶液，作为对照品参照物溶液。

供试品溶液的制备 取本品适量，研细，取 2g，加 30%甲醇 20ml，超声处理（功率 250W，频率 40kHz）30 分钟，放冷，滤过，取续滤液，即得。

测定法 分别精密吸取参照物溶液与供试品溶液各 1μl，注入液相色谱仪，测定，即得。

供试品色谱图中应呈现与对照药材参照物色谱图中 7 个保留时间相对应的特征峰，峰 1、峰 2、峰 5 应分别与对照品参照物峰的保留时间相对应。峰 3、峰 4、峰 6、峰 7 与 S 峰（峰 5）的相对保留时间依次约为：0.64、0.80、1.30、1.34。

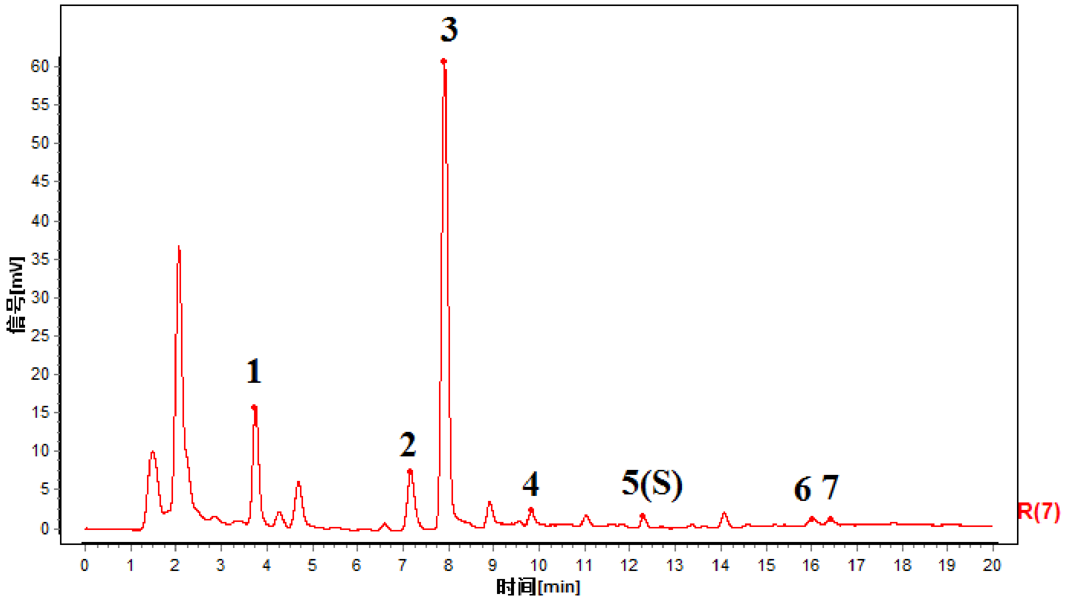

对照特征图谱

峰 1：尿苷 峰 2：鸟苷 峰 5（S）：色氨酸

色谱柱： CORTECS T3（100mm×2.1mm，1.6μm）

【检查】溶化性 照颗粒剂溶化性检查方法（中国药典 通则 0104）检查，加热水 200ml，搅拌 5 分钟（必要时加热煮沸 5 分钟），立即观察，应全部溶化或轻微浑浊，不得有焦屑或异物。

水麦冬酸 照高效液相色谱法（中国药典 通则 0512）测定。

色谱条件与系统适用性试验 以十八烷基硅烷键合硅胶为填充剂（柱长为

100mm，内径为 2.1mm，粒径为 1.6μm）；以乙腈为流动相 A，以 0.1%磷酸溶液为流动相 B，按下表梯度洗脱；采用二极管阵列检测器；流速为 0.3ml/min；柱温为 25℃；检测波长为 210nm。理论板数按水麦冬酸峰计算应不低于 3000。

时间（分钟）	流动相 A（%）	流动相 B（%）
0	1	99
9	1	99
10	10	90
11	1	99

对照品溶液的制备 （临用新制）取水麦冬酸对照品适量，精密称定，加乙腈-0.1%磷酸溶液（1:99）制成每 1ml 含 0.25μg 的溶液，作为对照品溶液。

供试品溶液的制备 同【特征图谱】项。

测定法 分别精密吸取对照品溶液与供试品溶液各 1μl，注入高效液相色谱仪，测定，记录色谱图，即得。

结果判断 供试品溶液色谱中，在与水麦冬酸对照品溶液色谱峰保留时间相应的位置上不得出现相同的色谱峰。若出现保留时间相同的色谱峰，则采用二极管阵列检测器比较相应色谱峰在 190～400nm 波长范围内紫外-可见吸收光谱，吸收光谱应不相同。

备注：必要时可采用高效液相色谱-质谱联用方法确证。建议采用甲醇-0.02%氨溶液（5:95）流动相系统。

其他 应符合颗粒剂项下有关的各项规定（中国药典 通则 0104）。

【规格】 每 1g 配方颗粒相当于饮片 3.2g

【贮藏】 密封。
