# Supplementary material for: Efficacy of Xiaoyao-san preparations in treating Hashimoto’s thyroiditis: a meta-analysis and systematic review
Source: Front Pharmacol. 2025 Jun 13;16:1528506. doi: 10.3389/fphar.2025.1528506 (PMC12202410; doi:10.3389/fphar.2025.1528506)
Supplement: Supplementary file 2 [file Supplementaryfile2.zip › Supplementary Files 2/Supplementary Files 2 formula granules section/Bohe_YBZ-PFKL-2021010 .pdf]

国家药品监督管理局
国家药品标准

YBZ-PFKL-2021010

薄荷配方颗粒

Bohe Peifangkeli

【来源】 本品为唇形科植物薄荷 *Mentha haplocalyx* Briq.的干燥地上部分经炮制并按标准汤剂的主要质量指标加工制成的配方颗粒。

【制法】 取薄荷饮片 3700g，加水煎煮，同时提取挥发油适量（以β-环糊精包合，备用），滤过，滤液浓缩成清膏（干浸膏出膏率范围：16%~25%），加入挥发油包合物，加入辅料适量，干燥（或干燥、粉碎），加辅料适量，混匀，制粒，制成 1000g，即得。

【性状】 本品为浅黄棕色至棕绿色颗粒；有特殊清凉香气，味辛凉。

【鉴别】 取本品 2.5g，研细，加水 15ml，超声处理 30 分钟，用石油醚（60～90℃）振摇提取 2 次，每次 15ml，合并石油醚液，低温浓缩挥至 1ml，作为供试品溶液。另取薄荷脑对照品，加石油醚（60～90℃）制成每 1ml 含 2mg 的溶液，作为对照品溶液。照薄层色谱法（中国药典 2020 年版 通则 0502）试验，吸取上述两种溶液各 5μl，分别点于同一硅胶 G 薄层板上，以甲苯-乙酸乙酯（19∶1）为展开剂，展开，取出，晾干，喷以 5%香草醛硫酸溶液，在 100℃加热至斑点显色清晰。供试品色谱中，在与对照品色谱相应的位置上，显相同颜色的斑点。

【特征图谱】 照高效液相色谱法（中国药典 2020 年版 通则 0512）测定。

色谱条件与系统适用性试验 同〔含量测定〕项。

参照物溶液的制备 取薄荷对照药材 0.5g，加水 15ml，加热回流 30 分钟，放冷，离心，取上清液，置 10ml 容量瓶中，加水至刻度，滤过，取续滤液作为对照药材参照物溶液。另取蒙花苷对照品适量，精密称定，加少量二甲基亚砜使溶解，再加甲醇制成每 1ml 含 0.1mg 的溶液，作为蒙花苷对照品参照物溶液。再取〔含量测定〕项对照品溶液，作为迷迭香酸对照品参照物溶液。

供试品溶液的制备 同〔含量测定〕项。

测定法 分别精密吸取参照物溶液与供试品溶液各 2μl，注入液相色谱仪，测定，即得。

供试品色谱中应呈现 7 个特征峰，并应与对照药材参照物色谱中的 7 个特征峰保留时间相对应，其中 2 个峰应分别与相应对照品参照峰的保留时间相对应，与迷迭香酸相对应的峰为 S 峰，计算其余特征峰与 S 峰的相对保留时间，其相对保留时间应在规定值的±10%范围之内。规定值为：0.30（峰 1）、0.34（峰 2）、0.64（峰 3）、0.91（峰 4）、1.16（峰 6）。计算峰 7 与 S 峰的相对峰面积，其比值不得大于 0.5。

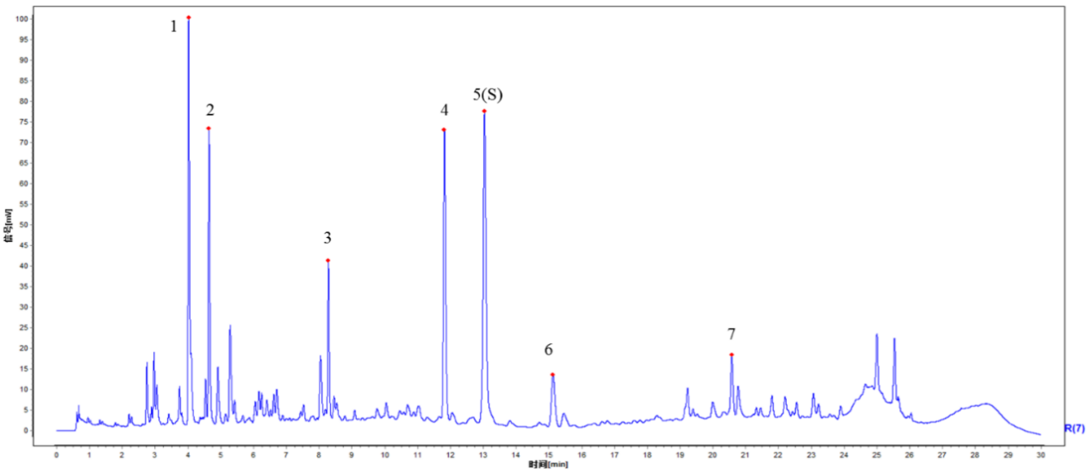

对照特征图谱

峰 5（S）：迷迭香酸；峰 7：蒙花苷

色谱柱： CORTECS T3， 2.1mm×100mm，1.6μm

【检查】 应符合颗粒剂项下有关的各项规定（中国药典 2020 年版 通则 0104）

【浸出物】 照醇溶性浸出物测定法（中国药典 2020 年版 通则 2201）项下的热浸法测定，用乙醇作溶剂，不得少于 12.0%。

【含量测定】 **挥发油** 取本品每 100g 加水 2000ml，照挥发油测定法（中国药典 2020 版 通则 2204 甲法）保持微沸 3 小时测定。

本品含挥发油应为 0.25%-0.50%（ml/g）。

迷迭香酸 照高效液相色谱法（中国药典 2020 年版 通则 0512）测定。

色谱条件与系统适用性试验 以十八烷基硅烷键合硅胶为填充剂（柱长为 100mm，内径为 2.1mm，粒径为 1.6μm）；以甲醇为流动相 A，以 0.10%磷酸溶液为流动相 B，按下表中的规定进行梯度洗脱；流速为每分钟 0.35ml；柱温为 40℃；检测波长为 334nm。理论板数按迷迭香酸峰计算应不低于 3000。

时间（分钟）	流动相 A（%）	流动相 B（%）
0～5	10→25	90→75
5～9	25→33	75→67
9～14	33	67
14～22.5	33→50	67→50
22.5～26	50→95	50→5
26～27	95	5
27～30	95→10	5→90

对照品溶液的制备 取迷迭香酸对照品适量，精密称定，加甲醇制成每 1ml 含 30μg 的溶液，即得。

供试品溶液的制备 取本品适量，研细，取约 0.2g，精密称定，置具塞锥形瓶中，精密

加水20ml，使溶解，摇匀，滤过，取续滤液，即得。

测定法　分别精密吸取对照品溶液与供试品溶液各2μl，注入液相色谱仪，测定，即得。

本品每1g含迷迭香酸（$C_{18}H_{16}O_8$）应为1.0mg～4.0mg。

【规格】　每1g配方颗粒相当于饮片3.7g

【贮藏】　密封，置阴凉干燥处。
