# Supplementary material for: Efficacy of Xiaoyao-san preparations in treating Hashimoto’s thyroiditis: a meta-analysis and systematic review
Source: Front Pharmacol. 2025 Jun 13;16:1528506. doi: 10.3389/fphar.2025.1528506 (PMC12202410; doi:10.3389/fphar.2025.1528506)
Supplement: Supplementary file 2 [file Supplementaryfile2.zip › Supplementary Files 2/Supplementary Files 2 formula granules section/Chaihu_YBZ-PFKL-2021011 .pdf]

国家药品监督管理局
国家药品标准

YBZ-PFKL-2021011

北柴胡配方颗粒

Beichaihu Peifangkeli

【来源】 本品为伞形科植物柴胡 *Bupleurum chinense* DC.的干燥根经炮制并按标准汤剂的主要质量指标加工制成的配方颗粒。

【制法】 取北柴胡饮片 4000g，加水煎煮，滤过，滤液浓缩成清膏（干浸膏出膏率为12%~19%），加辅料适量，干燥（或干燥，粉碎），再加辅料适量，混匀，制粒，制成 1000g，即得。

【性状】 本品为黄色至黄棕色的颗粒；气微，味微苦。

【鉴别】 取本品 0.5g，研细，加甲醇 20ml，超声处理 10 分钟，滤过，滤液浓缩至2ml，作为供试品溶液。另取柴胡（北柴胡）对照药材 1g，加水 50ml，煎煮 30 分钟，滤过，滤液蒸干，残渣加甲醇 20ml，同法制备对照药材溶液。再取柴胡皂苷 a 对照品，加甲醇制成每 1ml 含 0.5mg 的溶液，作为对照品溶液。照薄层色谱法（中国药典 2020 年版通则 0502）试验，吸取上述供试品溶液及对照品溶液各 3μl、对照药材溶液 6μl，分别点于同一硅胶 G 薄层板上，以乙酸乙酯-乙醇-水（8∶2∶1）为展开剂，预饱和 30 分钟，展开，取出，晾干，喷以 2%对二甲氨基苯甲醛的 40%硫酸溶液，在 60℃加热至斑点显色清晰，分别在日光和紫外光（365nm）下检视。供试品色谱中，在与对照药材和对照品色谱相应位置上，分别显相同颜色的斑点或荧光斑点。

【特征图谱】 照高效液相色谱法（中国药典 2020 年版通则 0512）测定。

色谱条件与系统适用性试验 以十八烷基硅烷键合硅胶为填充剂，（柱长为 100mm，内径为 2.1mm，粒径为 1.7μm）；以乙腈为流动相 A，以水为流动相 B，按下表中的规定进行梯度洗脱；检测波长为 211nm、250nm；柱温为 35℃；流速为每分钟 0.4ml。理论板数按柴胡皂苷 a 峰计算应均不低于 10000。

时间（分钟）	流动相 A（%）	流动相 B（%）
0～8	25→28	75→72
8～15	28→29	72→71
15～20	29→36	71→64
20～28	36	64
28～31	36→40	64→60
31～37	40	60

国家药品监督管理局　　发布　　　　国家药典委员会　　审定

参照物溶液的制备　取柴胡（北柴胡）对照药材0.5g，置锥形瓶中，加水25ml，加热回流30分钟，滤过，取续滤液20ml，蒸干，加5%浓氨试液的50%乙醇溶液25ml，超声（功率250W，频率40kHz）30分钟，取出，放冷，摇匀，滤过，取续滤液，作为对照药材参照物溶液。另取〔含量测定〕项对照品溶液，作为对照品参照物溶液。

供试品溶液的制备　同〔含量测定〕项。

测定法　精密吸取参照物溶液及供试品溶液各3μl，注入液相色谱仪，测定，即得。

在211nm下，供试品色谱中应呈现4个特征峰，并应与对照药材参照物色谱中的4个特征峰保留时间相对应，与柴胡皂苷a参照物峰相应的峰为S峰，计算各特征峰与S峰的相对保留时间，峰1和峰2的相对保留时间应在规定值的±10%范围之内，峰4的相对保留时间应在规定值的±8%范围之内，规定值为：0.61（峰1）、0.69（峰2）、1.04（峰4）；且峰1与峰3面积的比值应小于0.35；在250nm下，供试品色谱中应呈现4个特征峰，并应与对照药材参照物色谱中的4个特征峰保留时间相对应，峰8面积与峰8面积及峰6峰面积之和的比值应不大于0.85。

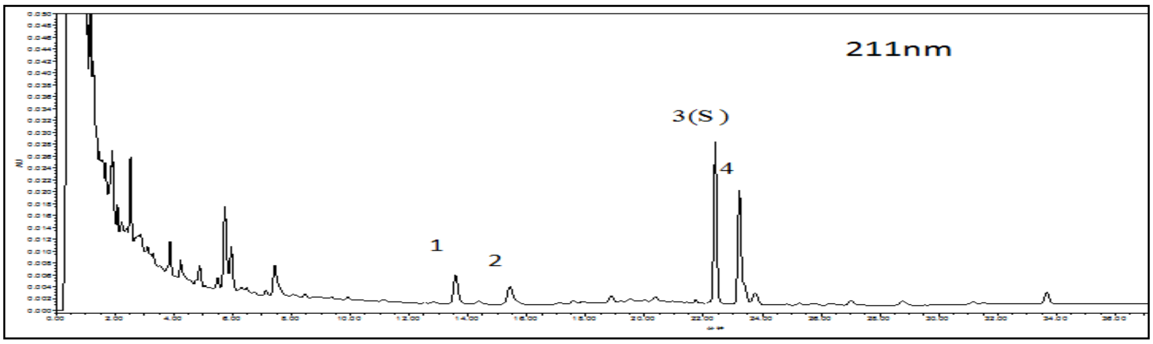

对照特征图谱（211nm）

峰1：柴胡皂苷c；峰2：柴胡皂苷f；峰3：柴胡皂苷a；峰4：柴胡皂苷b_2

色谱柱：　BEH C18，2.1mm×100mm，1.7μm

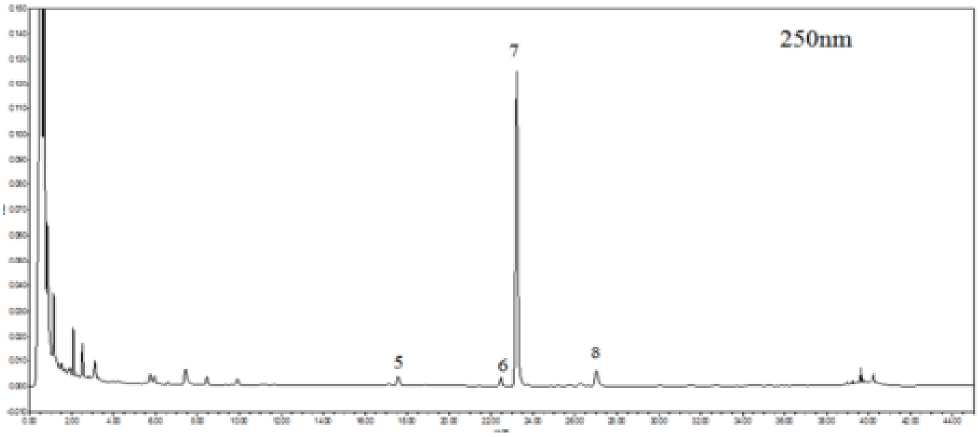

对照特征图谱（250nm）

峰7：柴胡皂苷b_2；峰8：柴胡皂苷b_1

色谱柱： BEH C18，2.1mm×100mm，1.7μm

【检查】 应符合颗粒剂项下有关的各项规定（中国药典 2020 年版通则 0104）。

【浸出物】 照醇溶性浸出物测定法（中国药典 2020 年版通则 2201）项下的热浸法测定，用乙醇作溶剂，不得少于 18.0%。

【含量测定】 照高效液相色谱法（中国药典 2020 年版通则 0512）测定。

色谱条件与系统适用性试验 以十八烷基硅烷键合硅胶为填充剂，（柱长为 100mm，内径为 2.1mm，粒径为 1.7μm）；以乙腈为流动相 A，以水为流动相 B，按下表中的规定进行梯度洗脱；检测波长为 211nm；柱温为 35℃；流速为每分钟 0.4ml。理论板数按柴胡皂苷 a 峰计算应均不低于 10000。

时间（分钟）	流动相 A（%）	流动相 B（%）
0～8	25→28	75→72
8～15	28→29	72→71
15～20	29→36	71→64
20～28	36	64
28～31	36→40	64→60
31～37	40	60

对照品溶液的制备 取柴胡皂苷 a 对照品适量，精密称定，加甲醇制成每 1ml 含 75μg 的溶液，即得。

供试品溶液的制备 取本品适量，研细，取约 1.0g，精密称定，置具塞锥形瓶中，精密加入含 5%浓氨试液的 50%乙醇溶液 25ml，密塞，称定重量，超声处理（功率 250W，频率 40kHz）30 分钟，放冷，再称定重量，用 5%浓氨试液的 50%乙醇溶液补足减失的重量，摇匀，滤过，取续滤液，即得。

测定法 分别精密吸取对照品溶液与供试品溶液各 3μl，注入液相色谱仪，测定，即得。

本品每 1g 含柴胡皂苷 a（$C_{42}H_{68}O_{13}$）应为 1.60mg～5.00mg。

【规格】 每 1g 配方颗粒相当于饮片 4g

【贮藏】 密封。
