# Supplementary material for: Efficacy of Xiaoyao-san preparations in treating Hashimoto’s thyroiditis: a meta-analysis and systematic review
Source: Front Pharmacol. 2025 Jun 13;16:1528506. doi: 10.3389/fphar.2025.1528506 (PMC12202410; doi:10.3389/fphar.2025.1528506)
Supplement: Supplementary file 2 [file Supplementaryfile2.zip › Supplementary Files 2/Supplementary Files 2 formula granules section/Chenpi_YBZ-PFKL-2021025.pdf]

国家药品监督管理局
国家药品标准

YBZ-PFKL-2021025

陈皮配方颗粒

Chenpi Peifangkeli

【来源】 本品为芸香科植物橘 *Citrus reticulate* Blanco 及其栽培变种的干燥成熟果皮经炮制并按标准汤剂的主要质量指标加工制成的配方颗粒。

【制法】 取陈皮饮片 2000g，加水煎煮，滤过，滤液浓缩成清膏（干浸膏出膏率为 25%~40%），加入辅料适量，干燥（或干燥，粉碎），再加入辅料适量，混匀，制粒，制成 1000g，即得。

【性状】 本品为棕黄色至棕色的颗粒；气香，味辛、苦。

【鉴别】 取本品1g，研细，加甲醇10ml，加热回流20分钟，滤过，取滤液5ml，浓缩至约1ml，作为供试品溶液。另取陈皮对照药材1g，同法制成对照药材溶液。再取橙皮苷对照品，加甲醇制成饱和溶液，作为对照品溶液。照薄层色谱法（中国药典2020年版通则0502）试验，吸取供试品溶液2~5μl、对照品溶液5μl、对照药材溶液3μl，分别点于同一用0.5%氢氧化钠溶液制备的硅胶G薄层板上，以乙酸乙酯-甲醇-水（100∶17∶13）为展开剂，展至约3cm，取出，晾干，再以甲苯-乙酸乙酯-甲酸-水（20∶10∶1∶1）的上层溶液为展开剂，展至约8cm，取出，晾干，喷以三氯化铝试液，置紫外光灯（365nm）下检视。供试品色谱中，在与对照药材和对照品色谱相应的位置上，显相同颜色的荧光斑点。

【特征图谱】 照高效液相色谱法（中国药典 2020 年版通则 0512）测定。

色谱条件与系统适用性试验 以十八烷基硅烷键合硅胶为填充剂（柱长为 100mm，内径为 2.1mm，粒径为 2.2μm）；以乙腈为流动相 A，以 0.5%冰醋酸溶液为流动相 B，按下表中的规定进行梯度洗脱；流速为每分钟 0.40ml；柱温为 25℃；检测波长为 283nm。理论板数按橙皮苷峰计算应不低于 15000。

时间（分钟）	流动相 A（%）	流动相 B（%）
0~15	13→20	87→80
15~25	20→34	80→66
25~44	34→42	66→58

参照物溶液的制备 取陈皮对照药材 1g，置具塞锥形瓶中，加甲醇 100ml，超声处理（功率 300W，频率 45kHz）60 分钟，取出，放冷，摇匀，滤过，取续滤液，作为对照药材参照物溶液；另取柚皮芸香苷对照品、橙皮苷对照品、川陈皮素对照品、橘皮素对照品适量，精密称定，加甲醇制成每 1ml 各含 100μg 的混合溶液，作为对照品参照物溶液。

供试品溶液的制备 同〔含量测定〕项。

测定法 分别精密吸取参照物溶液与供试品溶液各 1μl，注入液相色谱仪，测定，即得。

供试品色谱中应呈现 5 个特征峰，并应与对照药材参照物色谱中的 5 个特征峰保留时间相对应，其中 4 个峰应分别与相应对照品参照物峰的保留时间相对应。与橙皮苷参照物峰相对应的峰为 S 峰，计算峰 3 与 S 峰的相对保留时间，其相对保留时间应在规定值的±10%范围之内，规定值为：1.60（峰 3）；计算峰 1、峰 4、峰 5 与 S 峰的相对峰面积，其相对峰面积应在规定的范围内，规定范围为：0.03~0.92（峰 1）、不低于 0.03（峰 4）、不低于 0.02（峰 5）。

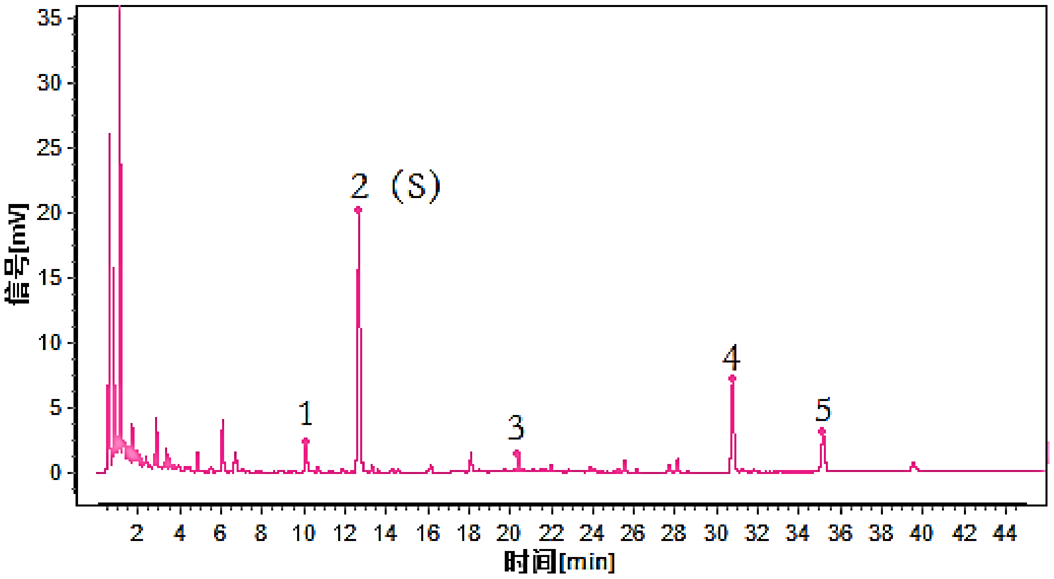

对照特征图谱

峰 1：柚皮芸香苷;峰 2（S）：橙皮苷;峰 4：川陈皮素;峰 5：橘皮素

色谱柱:Acclaim RSLC 120 C18，2.1mm×100mm，2.2μm

【检查】 **黄曲霉毒素** 照黄曲霉毒素测定法（中国药典 2020 年版 通则 2351） 测定。

本品每 1000g 含黄曲霉毒素 B1 不得过 5μg，含黄曲霉毒素 G2、黄曲霉毒素 G1、黄曲霉毒素 B2 和黄曲霉毒素 B1 的总量不得过 10μg。

其他 应符合颗粒剂项下有关的各项规定（中国药典 2020 年版通则 0104）。

【浸出物】 取本品研细，取约 2g，精密称定，精密加入乙醇 100ml，照醇溶性浸出物测定法（中国药典 2020 年版通则 2201）项下的热浸法测定，不得少于 23.0%。

【含量测定】 照高效液相色谱法（中国药典 2020 年版通则 0512）测定。

色谱条件与系统适用性试验 以十八烷基硅烷键合硅胶为填充剂；以甲醇-醋酸-水（35∶4∶61）为流动相；检测波长为 283nm。理论板数按橙皮苷峰计算应不低于 2000。

对照品溶液的制备 取橙皮苷对照品适量，精密称定，加甲醇制成每 1ml 含 0.1mg 的溶液，即得。

供试品溶液的制备 取本品适量，研细，取约 0.2g，精密称定，置具塞锥形瓶中，精密加入甲醇 50ml，称定重量，超声处理（功率 300W，频率 40kHz）30 分钟，放冷，再称定重量，用甲醇补足减失的重量，摇匀，滤过，取续滤液，即得。

测定法 分别精密吸取对照品溶液与供试品溶液各 10μl，注入液相色谱仪，测定，即得。

本品每 1g 含橙皮苷（$C_{28}H_{34}O_{15}$）应为 6.5mg~14.5mg。

【规格】 每 1g 配方颗粒相当于饮片 2g

【贮藏】 密封。
