# Supplementary material for: Efficacy of Xiaoyao-san preparations in treating Hashimoto’s thyroiditis: a meta-analysis and systematic review
Source: Front Pharmacol. 2025 Jun 13;16:1528506. doi: 10.3389/fphar.2025.1528506 (PMC12202410; doi:10.3389/fphar.2025.1528506)
Supplement: Supplementary file 2 [file Supplementaryfile2.zip › Supplementary Files 2/Supplementary Files 2 formula granules section/Danggui_YBZ-PFKL-2021037.pdf]

国家药品监督管理局
国家药品标准

YBZ-PFKL-2021037

当归配方颗粒
Danggui Peifangkeli

【来源】 本品为伞形科植物当归 *Angelica sinensis* (Oliv.) Diels 的干燥根经炮制并按标准汤剂的主要质量指标加工制成的配方颗粒。

【制法】 取当归饮片 1500g，加水煎煮，滤过，滤液浓缩成清膏（干浸膏出膏率为 35%~56%），加辅料适量，干燥（或干燥，粉碎），再加辅料适量，混匀，制粒，制成 1000g，即得。

【性状】 本品为浅黄色至棕黄色的颗粒；气微，味微甘、微苦。

【鉴别】 （1）取本品 1g，研细，加水 20ml 使溶解，用乙醚振摇提取 2 次，每次 20ml，合并乙醚液，挥干，残渣加甲醇 1ml 使溶解，作为供试品溶液。另取当归对照药材 0.5g，加乙醚 20ml，超声处理 10 分钟，滤过，滤液挥干，残渣加甲醇 1ml 使溶解，作为对照药材溶液。照薄层色谱法（中国药典 2020 年版通则 0502）试验，吸取上述两种溶液各 10µl，分别点于同一硅胶 G 薄层板上，以正己烷-乙酸乙酯（1∶1）为展开剂，展开，取出，晾干，置紫外光灯（365nm）下检视。供试品色谱中，在与对照药材色谱相应的位置上，显相同颜色的荧光主斑点。

（2）取本品 1g，研细，加 1%碳酸氢钠溶液 50ml，超声处理 30 分钟，滤过，滤液用稀盐酸调节 pH 值至 2～3，用乙醚振摇提取 3 次（20ml，15ml，15ml），合并乙醚液，挥干，残渣加甲醇 1ml 使溶解，作为供试品溶液。另取当归对照药材 1g，同法制成对照药材溶液。再取阿魏酸对照品，加甲醇制成每 1ml 含 1mg 的溶液，作为对照品溶液。照薄层色谱法（中国药典 2020 年版通则 0502）试验，吸取上述三种溶液各 10µl，分别点于同一硅胶 G 薄层板上，以环己烷-二氯甲烷-乙酸乙酯-甲酸（4∶1∶1∶0.1）为展开剂，展开，取出，晾干，置紫外光灯（365nm）下检视。供试品色谱中，在与对照药材色谱和对照品色谱相应的位置上，显相同颜色的荧光斑点。

【特征图谱】 照高效液相色谱法（中国药典 2020 年版通则 0512）测定。

色谱条件与系统适用性试验 以十八烷基硅烷键合硅胶为填充剂（柱长为 100mm，内径为 2.1mm，粒径为 1.6µm）；以乙腈为流动相 A，以 0.1%甲酸溶液为流动相 B，按下表中的规定进行梯度洗脱；流速为每分钟 0.3ml；柱温为 35℃；检测波长为 270nm。理论板数按阿魏酸峰计算应不低于 5000。

时间（分钟）	流动相 A（%）	流动相 B（%）
0～3	0	100
3～5	0→4	100→96
5～16	4→30	96→70
16～17	30→100	70→0
17～20	100	0

国家药品监督管理局 发布 国家药典委员会 审定

20～20.1	100→0	0→100

参照物溶液的制备　取当归对照药材 0.2g，置具塞锥形瓶中，加水 20ml，超声处理（功率 250W，频率 40kHz）20 分钟，放冷，摇匀，滤过，取续滤液，作为对照药材参照物溶液。另取腺苷对照品、色氨酸对照品、阿魏酸对照品适量，精密称定，置棕色量瓶中，加 70%甲醇制成每 1ml 含腺苷 20μg、色氨酸 20μg、阿魏酸 12μg 的溶液，作为对照品参照物溶液。

供试品溶液的制备　取本品适量，研细，取 0.2g，同“对照药材参照物溶液”制备方法制备供试品溶液。

测定法　分别精密吸取参照物溶液 1μl、供试品溶液 2μl，注入液相色谱仪，测定，即得。

供试品色谱中应呈现 7 个特征峰，并应与对照药材参照物色谱中的 7 个特征峰保留时间相对应；其中峰 2、峰 4 和峰 5 应分别与腺苷对照品、色氨酸对照品和阿魏酸对照品参照物峰的保留时间相对应。与腺苷参照物相应的峰为 S1 峰，计算峰 1、峰 3 与 S1 峰的相对保留时间，其相对保留时间应在规定值的±10%之内。规定值为：0.56（峰 1）、1.20（峰 3）。与阿魏酸参照物相应的峰为 S2 峰，计算峰 6、峰 7 与 S2 峰的相对保留时间，其相对保留时间应在规定值的±10%之内。规定值为：1.16（峰 6）、1.21（峰 7）。

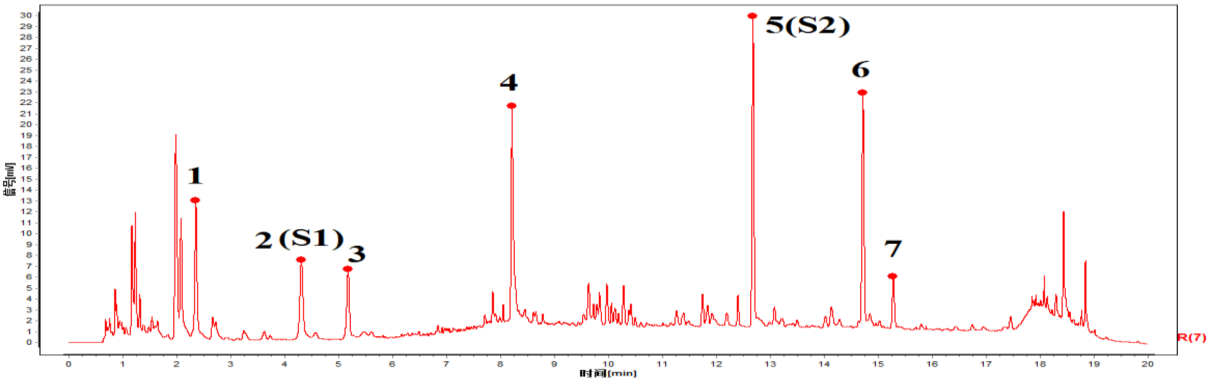

对照特征图谱

峰 1：尿苷；峰 2（S1）：腺苷；峰 3：鸟苷；峰 4：色氨酸

峰 5（S2）：阿魏酸；峰 6：洋川芎内酯 I；峰 7：洋川芎内酯 H

色谱柱：CORTECS T3 C18，2.1mm×100mm，1.6μm

【检查】　**重金属及有害元素**　照铅、镉、砷、汞、铜测定法（中国药典 2020 年版通则 2321 原子吸收分光光度法或电感耦合等离子体质谱法）测定，铅不得过 5mg/kg；镉不得过 1 mg/kg；砷不得过 2mg/kg；汞不得过 0. 2mg/kg；铜不得过 20mg/kg。

其他　应符合颗粒剂项下有关的各项规定（中国药典 2020 年版通则 0104）。

【浸出物】　照醇溶性浸出物测定法（中国药典 2020 年版通则 2201）项下的热浸法测定，用乙醇作溶剂，不得少于 10.0%。

【含量测定】　照高效液相色谱法（中国药典 2020 年版通则 0512）测定。

色谱条件与系统适用性试验　以十八烷基硅烷键合硅胶为填充剂（柱长为 100mm，内径为 2.1mm，粒径为 2.2μm）；以乙腈-0.085%磷酸溶液（17∶83）为流动相；流速为每分钟 0.4ml；检测波长为 316nm。理论板数按阿魏酸峰计算应不低于 5000。

对照品溶液的制备　取阿魏酸对照品适量，精密称定，置棕色量瓶中，加 70%甲醇制成每 1ml 含 6μg 的溶液，即得。

供试品溶液的制备　取本品适量，研细，取约 0.2g，精密称定，置具塞锥形瓶中，精密加入 70%甲醇 25ml，密塞，称定重量，超声处理（功率 250W，频率 40kHz）30 分钟，放冷，再称定重量，用 70%甲醇补足减失的重量，摇匀，滤过，取续滤液，即得。

测定法　分别精密吸取对照品溶液与供试品溶液各 2μl，注入液相色谱仪，测定，即得。

本品每 1g 含阿魏酸（$C_{10}H_{10}O_4$）应为 0.70mg~1.80mg。

【规格】　每 1g 配方颗粒相当于饮片 1.5g

【贮藏】　密封。
