# Supplementary material for: Efficacy of Xiaoyao-san preparations in treating Hashimoto’s thyroiditis: a meta-analysis and systematic review
Source: Front Pharmacol. 2025 Jun 13;16:1528506. doi: 10.3389/fphar.2025.1528506 (PMC12202410; doi:10.3389/fphar.2025.1528506)
Supplement: Supplementary file 2 [file Supplementaryfile2.zip › Supplementary Files 2/Supplementary Files 2 formula granules section/Foshou_YBZ-PFKL-2021044.pdf]

国家药品监督管理局
国家药品标准

YBZ-PFKL-2021044

佛手配方颗粒

Foshou Peifangkeli

【来源】 本品为芸香科植物佛手 *Citrus medica* L. var. *sarcodactylis* Swingle 的干燥果实经炮制并按标准汤剂的主要质量指标加工制成的配方颗粒。

【制法】 取佛手饮片 1600g，加水煎煮，滤过，滤液浓缩成清膏（干浸膏出膏率为 32%~45%），加入辅料适量，干燥（或干燥，粉碎），再加入辅料适量，混匀，制粒，制成 1000g，即得。

【性状】 本品为浅棕黄色至黄棕色的颗粒；气微香，味微甜后苦。

【鉴别】 取本品 0.3g，研细，加无水乙醇 10ml，超声处理 20 分钟，滤过，滤液蒸干，残渣加无水乙醇 0.5ml 使溶解，作为供试品溶液。另取佛手对照药材 1g，加水 50ml，煮沸 30 分钟，滤过，滤液蒸干，残渣加无水乙醇 10ml，同法制成对照药材溶液。照薄层色谱法（中国药典 2020 年版通则 0502）试验，吸取上述两种溶液各 6μl，分别点于同一硅胶 G 薄层板上，以环己烷-乙酸乙酯（3∶1）为展开剂，展开，取出，晾干，置紫外光灯（365nm）下检视。供试品色谱中，在与对照药材色谱相应的位置上，显相同颜色的荧光斑点。

【特征图谱】 照高效液相色谱法（中国药典 2020 年版通则 0512）测定。

色谱条件与系统适用性试验 以十八烷基硅烷键合硅胶为填充剂（柱长为 250mm，内径为 4.6mm，粒径为 5μm）；以乙腈为流动相 A，0.1%磷酸溶液为流动相 B，按下表中的规定进行梯度洗脱；流速为每分钟 1.0ml；柱温为 30℃；检测波长为 284nm。理论板数按橙皮苷峰计算应不低于 5000。

时间（分钟）	流动相 A（%）	流动相 B（%）
0~30	15→30	85→70
30~50	30→60	70→40
50~55	60→85	40→15
55~60	85	15

参照物溶液的制备 取佛手对照药材 0.5g，置具塞锥形瓶中，加 70%甲醇 50ml，超声处理（功率 250W，频率 40kHz）1 小时，放冷，摇匀，滤过，取续滤液，作为对照药材参照物溶液。另取橙皮苷对照品、5,7−二甲氧基香豆素对照品适量，精密称定，加甲醇制成每 1 ml 各含 10μg 的混合溶液，作为对照品参照物溶液。

供试品溶液的制备 同〔含量测定〕橙皮苷项。

测定法 分别精密吸取参照物溶液与供试品溶液各 10μl，注入液相色谱仪，测定，即得。

供试品色谱中应呈现 7 个特征峰，并应与对照药材参照物色谱中的 7 个特征峰保留时间相对应，其中 2 个峰应分别与相应的对照品参照物峰保留时间相对应。与橙皮苷参照物峰相对应的峰为 S 峰，计算峰 1、峰 3、峰 4、峰 5、峰 6 与 S 峰的相对保留时间，其相对保留时间应在规定值的±10%范围之内，规定值为：0.97（峰 1）、1.26（峰 3）、1.29（峰 4）、1.35（峰 5）、1.49（峰 6）。

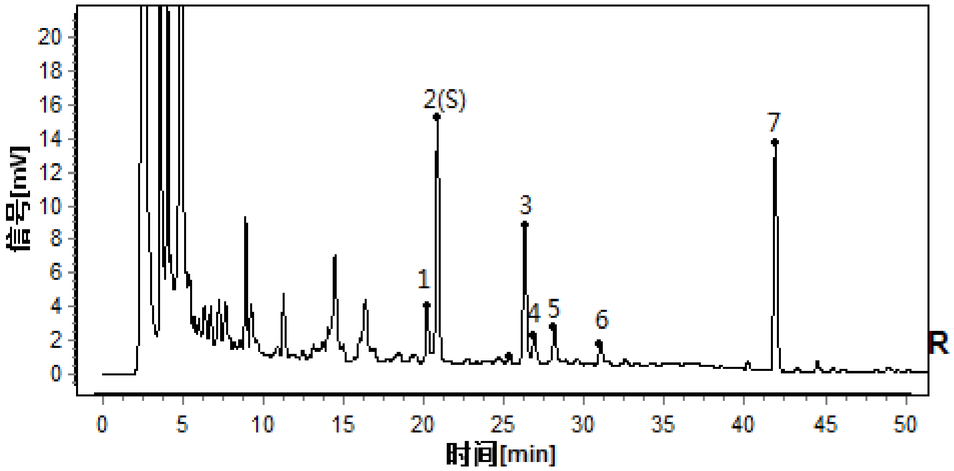

对照特征图谱

峰 1：香叶木苷；峰 2（S）：橙皮苷；峰 6：水合氧化前胡素；峰 7：5,7-二甲氧基香豆素

色谱柱：Xselect HSS T3 C18，4.6mm×250mm，5μm

【检查】 应符合颗粒剂项下有关的各项规定（中国药典 2020 年版通则 0104）。

【浸出物】 取本品研细，取约 2g，精密称定，精密加入乙醇 100ml，照醇溶性浸出物测定法（中国药典 2020 年版通则 2201）项下的热浸法测定，不得少于 22.0%。

【含量测定】 **总黄酮** 对照品溶液的制备 取槲皮素对照品适量，精密称定，加 80%乙醇制成每 1ml 含 0.2mg 的溶液，即得。

标准曲线的制备 精密量取对照品溶液 0.5ml、1.0ml、1.5ml、2.0ml、2.5ml、4.0ml，分别置 50ml 量瓶中，加 80%乙醇至刻度，摇匀。以 80%乙醇为空白，照紫外-可见分光光度法（中国药典 2020 年版通则 0401），在 256nm 波长处测定吸光度，以吸光度为纵坐标，浓度为横坐标，绘制标准曲线。

测定法 取本品适量，研细，取约 0.18g，精密称定，置具塞锥形瓶中，精密加入 80%乙醇 100ml，称定重量，超声处理（功率 250W，频率 40kHz）30 分钟，放冷，再称定重量，用 80%乙醇补足减失的重量，摇匀，滤过，精密量取续滤液 10ml，置分液漏斗中，用石油醚（60~90℃）洗涤 3 次，每次 5ml，弃去石油醚液，精密量取上清液 5ml，置 10ml 量瓶中，加 80%乙醇至刻度，摇匀。照标准曲线制备项下方法，测定其吸光度，根据标准曲线计算供试品中总黄酮含量。

本品每 1g 含总黄酮以槲皮素（$C_{15}H_{10}O_7$）计，应为 4.0mg~10.0mg。

橙皮苷 照高效液相色谱法（中国药典 2020 年版通则 0512）测定。

色谱条件与系统适用性试验 以十八烷基硅烷键合硅胶为填充剂（柱长为 100mm，内径为 2.1mm，粒径为 1.6μm~1.9μm）；以甲醇-水-冰醋酸（33∶63∶2）为流动相；流速为每分钟 0.22ml；柱温为 30℃；检测波长为 284 nm。理论板数按橙皮苷峰计算应不低于 5000。

对照品溶液的制备 取橙皮苷对照品适量，精密称定，加甲醇制成每 1 ml 含 10μg 的溶液，即得。

供试品溶液的制备 取本品适量，研细，取约 0.5g，精密称定，置具塞锥形瓶中，精密加入甲醇

15ml，称定重量，超声处理（功率 250W，频率 40kHz）30 分钟，放冷，再称定重量，用甲醇补足减失的重量，摇匀，滤过，取续滤液，即得。

测定法　分别精密吸取对照品溶液与供试品溶液各 1μl，注入液相色谱仪，测定，即得。

本品每 1g 含橙皮苷（$C_{28}H_{34}O_{15}$）应为 0.15mg~0.50mg。

【规格】　每 1g 配方颗粒相当于饮片 1.6g

【贮藏】　密封。
