# Supplementary material for: Efficacy of Xiaoyao-san preparations in treating Hashimoto’s thyroiditis: a meta-analysis and systematic review
Source: Front Pharmacol. 2025 Jun 13;16:1528506. doi: 10.3389/fphar.2025.1528506 (PMC12202410; doi:10.3389/fphar.2025.1528506)
Supplement: Supplementary file 2 [file Supplementaryfile2.zip › Supplementary Files 2/Supplementary Files 2 formula granules section/Fuling_BJ-PFKL-2022044.pdf]

# 北京市药品监督管理局
# 北京市中药配方颗粒标准

BJ-PFKL-2022044

## 茯苓配方颗粒

## Fuling Peifangkeli

**【来源】** 本品多孔菌科真菌茯苓 *Poria cocos* （Schw.） Wolf 的干燥菌核经炮制并按标准汤剂的主要质量指标加工制成的配方颗粒。

**【制法】** 取茯苓饮片 12500g，加水煎煮，滤过，滤液浓缩成清膏（干浸膏出膏率为 1.5%~3.5%），加入辅料适量，干燥，再加入辅料适量，混匀，制粒，制成 1000g，即得。

**【性状】** 本品为类白色至灰白色的颗粒；气微，味淡。

**【鉴别】** 取本品 3g，研细，加乙醚 50ml，超声处理 30 分钟，滤过，滤液蒸干，残渣加甲醇 1ml 使溶解，作为供试品溶液。另取茯苓对照药材 1g，同法制成对照药材溶液。照薄层色谱法（中国药典 2020 年版通则 0502）试验，吸取供试品溶液 8µl、对照药材溶液 12µl，分别点于同一硅胶 G 薄层板上，以甲苯-乙酸乙酯-甲酸（20:5:0.5）为展开剂，展开，取出，晾干，喷以 5%香草醛硫酸溶液，在 105℃加热至斑点显色清晰。供试品色谱中，在与对照药材色谱相应的位置上，显相同颜色的斑点。

**【特征图谱】** 照高效液相色谱法（中国药典 2020 年版通则 0512）测定。

**色谱条件与系统适用性试验** 同〔含量测定〕项。

**参照物溶液的制备** 取茯苓对照药材 3g，置具塞锥形瓶中，加 50%甲醇 25ml，置恒温振荡器中振摇 2 小时，超声处理（功率 250W，频率 40kHz）60 分钟，放冷，摇匀，离心，滤过，取续滤液，作为对照药材参照物溶液。另取茯苓酸 B 对照品、茯苓酸 A 对照品、猪苓酸 C 对照品适量，精密称定，加甲醇制成每 1ml 各含 20µg 的混合溶液，作为对照品参照物溶液。

**供试品溶液的制备** 同〔含量测定〕项。

**测定法** 精密吸取对照药材参照物溶液 10µl、对照品参照物溶液与供试品溶液各 2µl，注入超高效液相色谱仪，测定，即得。

供试品色谱中应呈现 4 个特征峰，并应与对照药材参照物色谱中的 4 个特征峰保留时间相对应。其中峰 2、峰 3、峰 4 应分别与茯苓酸 B、茯苓酸 A、猪苓酸 C 对照品参照物峰保留时间相对应；与茯苓酸 B 参照物峰相应的峰为 S 峰，计算峰 1 与 S 峰的相对保留时间，其相对保留时间应在规定值的±10%范围之内。规定值为：0.76（峰 1）。

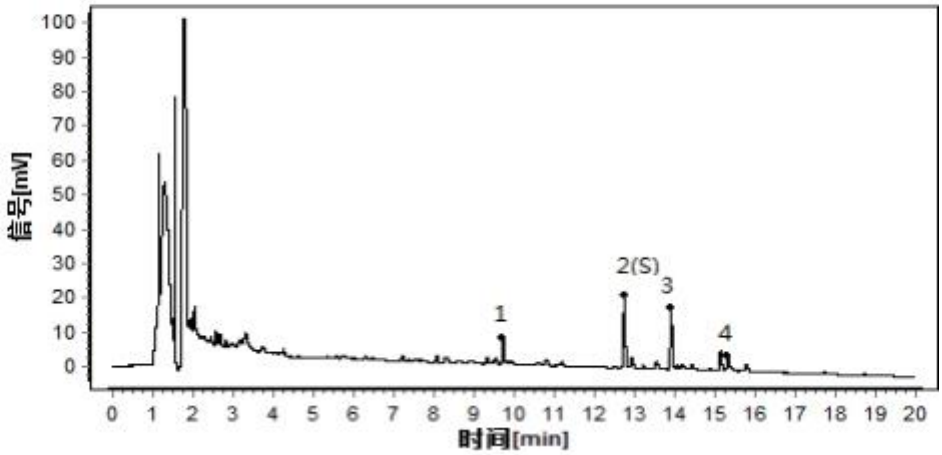


**对照特征图谱**

峰 2(S)：茯苓酸B　峰 3：茯苓酸A　峰 4：猪苓酸C

色谱柱:Luna Omega PS C18，2.1mm×150mm，1.6μm

**【检查】** 应符合颗粒剂项下有关的各项规定（中国药典 2020 年版通则 0104）。

**【浸出物】** 照醇溶性浸出物测定法（中国药典 2020 年版通则 2201）项下的热浸法测定，用乙醇作溶剂，不得少于 15.0%。

**【含量测定】** 照高效液相色谱法（中国药典 2020 年版通则 0512）测定。

**色谱条件与系统适用性试验** 以十八烷基硅烷键合硅胶为填充剂（柱长为 150mm，内径为 2.1mm，粒径为 1.6μm）；以乙腈为流动相 A，以 0.1%甲酸溶液为流动相 B，按下表中的规定进行梯度洗脱；流速为每分钟 0.2ml；柱温为 30℃；检测波长为 252nm。理论板数按茯苓酸 B 峰计算应不低于 5000。

| 时间（分钟） | 流动相 A（%） | 流动相 B（%） |
|---|---|---|
| 0~21 | 40→99 | 60→1 |
| 21~22 | 99→40 | 1→60 |

**对照品溶液的制备** 取茯苓酸B对照品、茯苓酸A对照品适量，精密称定，加甲醇制成每 1ml各含 20μg的混合溶液，即得。

**供试品溶液的制备** 取本品适量，研细，取约 0.24g，精密称定，置具塞锥形瓶中，精密加入 50%甲醇 10ml，密塞，称定重量，超声处理（功率 250W，频率 40kHz）30 分钟，放冷，再称定重量，用 50%甲醇补足减失的重量，摇匀，离心，取上清液，滤过，取续滤液，即得。

**测定法** 分别精密吸取对照品溶液与供试品溶液各 2μl，注入超高效液相色谱仪，测定，即得。

本品每 1g 含茯苓酸 B（$C_{30}H_{44}O_5$）应为 0.10mg~0.40mg；茯苓酸 A（$C_{31}H_{46}O_5$）应为 0.08mg~0.30mg。

**【规格】** 每 1g 配方颗粒相当于饮片 12.5g

**【贮藏】** 密封。
