# Supplementary material for: Efficacy of Xiaoyao-san preparations in treating Hashimoto’s thyroiditis: a meta-analysis and systematic review
Source: Front Pharmacol. 2025 Jun 13;16:1528506. doi: 10.3389/fphar.2025.1528506 (PMC12202410; doi:10.3389/fphar.2025.1528506)
Supplement: Supplementary file 2 [file Supplementaryfile2.zip › Supplementary Files 2/Supplementary Files 2 formula granules section/Fuling_JS-YBZ-2022216 .pdf]

江苏省药品监督管理局

中药配方颗粒标准

JS-YBZ-2022216

茯苓配方颗粒

Fuling Peifangkeli

【来源】 本品为多孔菌科真菌茯苓 *Poria cocos*（Schw.） Wolf 的干燥菌核经炮制并按标准汤剂的主要质量指标加工制成的配方颗粒。

【制法】 取茯苓饮片 12500g，加水煎煮，滤过，滤液浓缩成清膏（出膏率为 1.5~3.5%），加入辅料适量，干燥，再加入辅料适量，混匀，制粒，制成 1000g，即得。

【性状】 本品为浅灰色至灰黄色的颗粒；气微，味淡。

【鉴别】 取本品适量，研细，取 1g，加甲醇 20ml，超声处理 30 分钟，滤过，滤液蒸干，残渣加水 20ml，超声使溶解，加乙酸乙酯振摇提取 2 次，每次 20ml，合并乙酸乙酯液，蒸干，残渣加甲醇 1ml 使溶解，作为供试品溶液。另取茯苓对照药材 3g，加水 50ml，煮沸 30 分钟，滤过，滤液蒸干，残渣自“加甲醇 20ml”起，同法制成对照药材溶液。照薄层色谱法（中国药典 通则 0502）试验，吸取供试品溶液 3μl、对照药材溶液 20μl，分别点于同一聚酰胺薄膜上，以醋酸为展开剂，展开，取出，晾干，喷以 5%三氯化铝乙醇溶液，热风吹干，置紫外光灯（365nm）下检视。供试品色谱中，在与对照药材色谱相应的位置上，显相同颜色的荧光斑点。

【特征图谱】 照高效液相色谱法（中国药典 通则 0512）测定。

色谱条件与系统适用性试验 以十八烷基硅烷键合硅胶为填充剂；以乙腈为流动相 A，以 0.1%甲酸为流动相 B，按下表梯度洗脱；流速为 0.2ml/min；柱温为 30℃；检测波长为 252nm。理论板数按茯苓酸 B 计应不低于 5000。

时间（分钟）	流动相 A（%）	流动相 B（%）
0	40	60
21	99	1
22	40	60

参照物溶液的制备 取茯苓对照药材 3g，加 50%甲醇 25ml，超声处理 60 分钟，放冷，离心，滤过，取续滤液，作为对照药材参照物溶液。另取茯苓酸 B、茯苓酸 A、猪苓酸 C 对照品适量，精密称定，加甲醇制成每 1ml 各含 50μg 的混

合溶液，作为对照品参照物溶液。

供试品溶液的制备　取本品适量，研细，取约 0.24g，精密称定，置具塞锥形瓶中，精密加入 50%甲醇 10ml，称定重量，超声处理（功率 250W，频率 40kHz）30 分钟，放冷，再称定重量，用 50%甲醇补足减失的重量，摇匀，离心，上清液过 0.22μm 微孔滤膜，取续滤液，即得。

测定法　精密吸取对照品参照物溶液和供试品溶液各 2μl、对照药材参照物溶液 10μl，注入液相色谱仪，测定，即得。

供试品色谱图中应呈现与对照药材参照物色谱图中 4 个保留时间相对应的特征峰，峰 2~4 应分别与相应对照品参照物峰的保留时间相对应。峰 1 与 S 峰（峰 2）的相对保留时间约为：0.76。

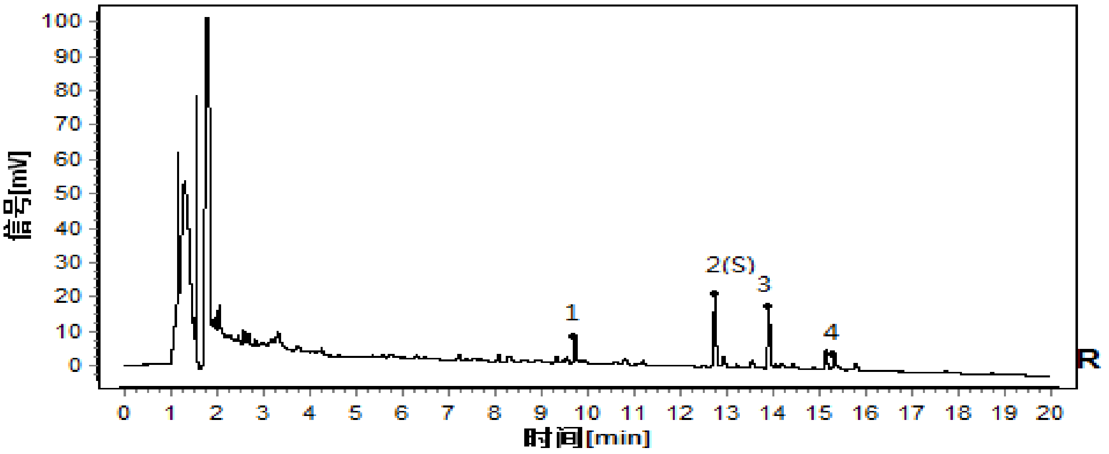

对照特征图谱

峰 2（S）：茯苓酸 B　峰 3：茯苓酸 A　峰 4：猪苓酸 C

色谱柱：　Luna Omega PS C18（150mm×2.1mm，1.6μm）

【检查】　应符合颗粒剂项下有关的各项规定（中国药典　通则 0104）。

【浸出物】　取本品研细，取约 2g，精密称定，精密加入乙醇 100ml，照醇溶性浸出物测定法（中国药典　通则 2201）项下的热浸法测定，不得少于 15.0%。

【含量测定】　照高效液相色谱法（中国药典　通则 0512）测定。

色谱条件与系统适用性试验　同【特征图谱】项。

对照品溶液的制备　取茯苓酸 B、茯苓酸 A 对照品适量，精密称定，加甲醇制成每 1ml 各含 20μg 的混合溶液，即得。

供试品溶液的制备　同【特征图谱】项。

测定法　精密吸取对照品溶液和供试品溶液各 2μl，注入液相色谱仪，测定，即得。

本品每 1g 含茯苓酸 B（$C_{30}H_{44}O_5$）含量应为 0.10~0.70mg；茯苓酸 A（$C_{31}H_{46}O_5$）

含量应为 0.09~0.60mg。

【规格】 每 1g 配方颗粒相当于饮片 12.5g

【贮藏】 密封。
