# Supplementary material for: Efficacy of Xiaoyao-san preparations in treating Hashimoto’s thyroiditis: a meta-analysis and systematic review
Source: Front Pharmacol. 2025 Jun 13;16:1528506. doi: 10.3389/fphar.2025.1528506 (PMC12202410; doi:10.3389/fphar.2025.1528506)
Supplement: Supplementary file 2 [file Supplementaryfile2.zip › Supplementary Files 2/Supplementary Files 2 formula granules section/Gancao_YBZ-PFKL-2021049.pdf]

国家药品监督管理局
国家药品标准

YBZ-PFKL-2021049

甘草（甘草）配方颗粒

Gancao（Gancao） Peifangkeli

【来源】 本品为豆科植物甘草 *Glycyrrhiza uralensis* Fisch. 的干燥根和根茎经炮制并按标准汤剂的主要质量指标加工制成的配方颗粒。

【制法】 取甘草饮片 3000g，加水煎煮，滤过，滤液浓缩成清膏（干浸膏出膏率为 20%~33%），干燥（或干燥，粉碎），加辅料适量，混匀，制粒，制成 1000g，即得。

【性状】 本品为黄色至棕黄色的颗粒；气微，味甜而特异。

【鉴别】 取本品 0.2g，研细，加水 20ml 使溶解，用水饱和的正丁醇振摇提取 2 次，每次 20ml，合并正丁醇液，蒸干，残渣加甲醇 5ml 使溶解，作为供试品溶液。另取甘草（甘草）对照药材 0.5g，加水 50ml，煮沸 30 分钟，滤过，滤液浓缩至 20ml，同法制成对照药材溶液。再取甘草酸铵对照品，加甲醇制成每 1ml 含 2mg 的溶液，作为对照品溶液。照薄层色谱法（中国药典 2020 年版通则 0502）试验，吸取上述三种溶液各 2μl，分别点于同一用 1%氢氧化钠溶液制备的硅胶 G 薄层板上，以乙酸乙酯-甲酸-冰醋酸-水（15∶1∶1∶2）为展开剂，展开，取出，晾干，喷以 10%硫酸乙醇溶液，在 105℃加热至斑点显色清晰，置紫外光灯（365nm）下检视。供试品色谱中，在与对照药材色谱和对照品色谱相应的位置上，显相同颜色的荧光斑点。

【特征图谱】 照高效液相色谱法（中国药典 2020 年版通则 0512）测定。

色谱条件与系统适用性试验 以十八烷基硅烷键合硅胶为填充剂（柱长为 100mm，内径为 2.1mm，粒径为 2.2μm）；以乙腈为流动相 A，以 0.1%磷酸溶液为流动相 B，按下表中的规定进行梯度洗脱；流速为每分钟 0.3ml；柱温为 30℃；检测波长为 237nm。理论板数按甘草酸峰计算应不低于 5000。

时间（分钟）	流动相 A（%）	流动相 B（%）
0～1	5→27	95→73
1～2	27	73
2～10	27→46	73→54
10～16	46→64	54→36
16～24	64→95	36→5
24～25	95	5

参照物溶液的制备 取甘草（甘草）对照药材0.2g，置具塞锥形瓶中，加入70%乙醇100ml，密塞，超声处理（功率250W，频率40kHz）30分钟，放冷，摇匀，滤过，取续滤液，作为对照药材参照物溶液。另取甘草苷对照品、甘草素对照品、甘草酸铵对照品适量，精密称定，加甲醇制成每1ml含甘草苷0.1mg、甘草素0.1mg、甘草酸铵0.2mg的溶液，作为对照品参照物溶液（甘草酸重量=甘草酸铵重量

/1.0207）。

供试品溶液的制备　同〔含量测定〕项下供试品溶液制备。

测定法　分别精密吸取参照物溶液与供试品溶液各 1μl，注入液相色谱仪，测定，即得。

供试品色谱中应呈现 12 个特征峰，并应与对照药材参照物色谱中的 12 个特征峰保留时间相对应，其中峰 3、峰 6、峰 10 应分别与甘草苷对照品、甘草素对照品、甘草酸对照品参照物峰保留时间相对应。与甘草苷参照物相应的峰为 S1 峰，计算峰 1~峰 2 与 S1 峰的相对保留时间，其相对保留时间应在规定值的±10%范围之内。规定值为 0.83（峰 1）、0.98（峰 2）。与甘草素参照物相应的为 S2 峰，计算峰 4、峰 5、峰 7 与 S2 峰的相对保留时间，其相对保留时间应在规定值的±10%范围之内。规定值为：0.82（峰 4）、0.87（峰 5）、1.09（峰 7）。与甘草酸参照物相应的峰作为 S3 峰，计算峰 8~峰 12 与 S3 峰的相对保留时间，其相对保留时间应在规定值的±10%范围之内，规定值为 0.80（峰 8）、0.92（峰 9）、1.09（峰 11）、1.26（峰 12）。

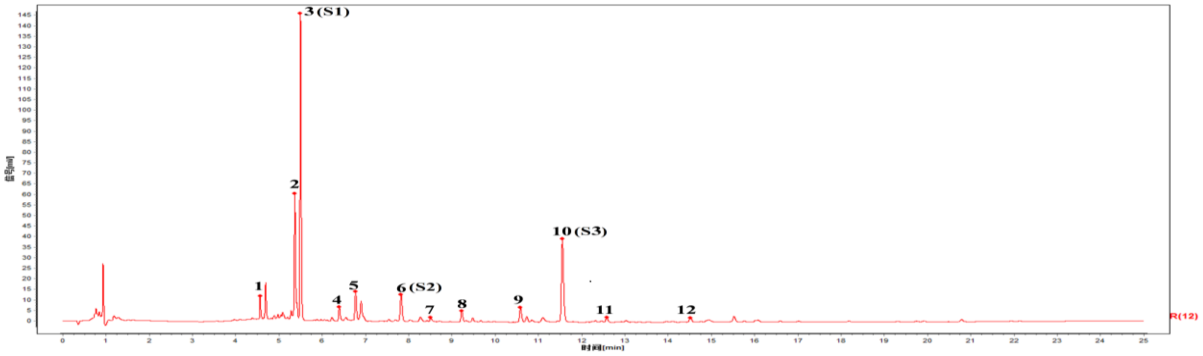

对照特征图谱

峰 2：芹糖甘草苷；峰 3（S1）：甘草苷；峰 5：异甘草苷

峰 6（S2）：甘草素；峰 10（S3）：甘草酸

色谱柱：Acclaim RSLC 120 C18，2.1mm×100mm，2.2μm

【检查】　**重金属及有害元素**　照铅、镉、砷、汞、铜测定法（中国药典 2020 年版通则 2321 电感耦合等离子体质谱法）测定，铅不得过 5mg/kg；镉不得过 1mg/kg；砷不得过 2mg/kg；汞不得过 0.2mg/kg；铜不得过 20mg/kg。

其他有机氯类农药残留量　照农药残留量测定法（中国药典 2020 年版通则 2341 有机氯类农药残留量测定法—第一法）测定。

含五氯硝基苯不得过 0.1mg/kg。

其他　应符合颗粒剂项下有关的各项规定（中国药典 2020 年版通则 0104）。

【浸出物】　照醇溶性浸出物测定法（中国药典 2020 年版通则 2201）项下的热浸法测定，用乙醇作溶剂，不得少于 32.0%。

【含量测定】　照高效液相色谱法（中国药典 2020 年版通则 0512）测定。

色谱条件与系统适用性试验　以十八烷基硅烷键合硅胶为填充剂(柱长为 100mm，内径为 2.1mm，粒径为 2.2μm)；以乙腈为流动相 A，以 0.05%磷酸溶液为流动相 B，按下表中规定进行梯度洗脱；流速为每分钟 0.4ml；检测波长为 237nm。理论板数按甘草苷峰计算应不低于 5000。

时间（分钟）	流动相 A（%）	流动相 B（%）
0~2	19	81
2~12.5	19→50	81→50
12.5~13	50→100	50→0
13~15	100→19	0→81

对照品溶液的制备 取甘草苷对照品、甘草酸铵对照品适量，精密称定，加 70%乙醇制成每 1ml 含甘草苷 60μg、甘草酸铵 0.1mg 的溶液，即得（甘草酸重量=甘草酸铵重量/1.0207）。

供试品溶液的制备 取本品适量，研细，取约 0.1g，精密称定，置具塞锥形瓶中，精密加入 70%乙醇 50ml，密塞，称定重量，超声处理（功率 250W，频率 40kHz）30 分钟，放冷，再称定重量，用 70%乙醇补足减失的重量，摇匀、滤过，取续滤液，即得。

测定法 分别精密吸取对照品溶液与供试品溶液各 1μl，注入液相色谱仪，测定，即得。

本品每 1g 含甘草苷（$C_{21}H_{22}O_9$）应为 15.0mg~35.0mg、含甘草酸（$C_{42}H_{62}O_{16}$）应为 29.0mg~80.0mg。

【注意】 不宜与海藻、京大戟、红大戟、甘遂、芫花同用。

【规格】 每 1g 配方颗粒相当于饮片 3g

【贮藏】 密封。
