# Supplementary material for: Efficacy of Xiaoyao-san preparations in treating Hashimoto’s thyroiditis: a meta-analysis and systematic review
Source: Front Pharmacol. 2025 Jun 13;16:1528506. doi: 10.3389/fphar.2025.1528506 (PMC12202410; doi:10.3389/fphar.2025.1528506)
Supplement: Supplementary file 2 [file Supplementaryfile2.zip › Supplementary Files 2/Supplementary Files 2 formula granules section/Haizao_Page 22_JS-YBZ-2022317.pdf]

江苏省药品监督管理局

中药配方颗粒标准

JS-YBZ-2022309

阿胶配方颗粒

Ejiao Peifangkeli

【来源】 本品为马科动物驴*Equus asinus* L.的干燥皮或鲜皮经煎煮、浓缩制成的固体胶经炮制并按标准汤剂的主要质量指标加工制成的配方颗粒。

【制法】 取阿胶900g，加水煎煮溶化，滤过（出膏率为72~100%），加入辅料适量，干燥（或干燥，粉碎），再加入辅料适量，混匀，制粒，制成1000g，即得。

【性状】 本品为浅棕色至棕褐色的颗粒；气微腥，味微甘。

【鉴别】 （1）取本品适量，研细，取0.2g，置氨基酸水解管中，加6mol/L盐酸溶液8ml，密塞，在105℃加热6小时，放冷，加水6ml，摇匀，滤过，滤液蒸干，残渣加甲醇10ml使溶解，作为供试品溶液。另取阿胶对照药材0.2g，同法制成对照药材溶液。再取甘氨酸对照品，加甲醇制成每1ml含1mg的溶液，作为对照品溶液。照薄层色谱法（中国药典 通则0502）试验，吸取供试品溶液与对照药材溶液各1μl、对照品溶液5μl，分别点于同一硅胶G薄层板上，以苯酚-0.5%硼砂溶液（4∶1）为展开剂，展开，取出，晾干，喷以0.5%茚三酮乙醇溶液，在105℃加热至斑点显色清晰。供试品色谱中，在与对照药材色谱和对照品色谱相应的位置上，显相同颜色的斑点。

（2）取本品适量，研细，取0.1g，加1%碳酸氢铵溶液50ml，超声处理30分钟，用微孔滤膜滤过，取续滤液100μl，置微量进样瓶中，加胰蛋白酶溶液10μl（取序列分析用胰蛋白酶，加1%碳酸氢铵溶液制成每1ml中含1mg的溶液，临用时配制），摇匀，37℃恒温酶解12小时，作为供试品溶液。另取阿胶对照药材0.1g，同法制成对照药材溶液。照【含量测定】特征多肽项下色谱、质谱条件试验，选择质荷比（m/z）539.8（双电荷）→612.4和m/z 539.8（双电荷）→923.8作为检测

离子对。取阿胶对照药材溶液，进样5μl，按上述检测离子对测定的MRM色谱峰的信噪比均应大于3∶1。

吸取供试品溶液5μl，注入高效液相色谱-质谱联用仪，测定。以质荷比（m/z）539.8（双电荷）→612.4和m/z 539.8（双电荷）→923.8离子对提取的供试品离子流色谱中，应同时呈现与对照药材色谱保留时间一致的色谱峰。

【检查】　重金属及有害元素　照铅、镉、砷、汞、铜测定法（中国药典 通则2321）测定，铅不得过5mg/kg；镉不得过1mg/kg；砷不得过2mg/kg；汞不得过0.2mg/kg；铜不得过20mg/kg。

其他　应符合颗粒剂（中国药典 通则0104）项下有关的各项规定。

【浸出物】　取本品适量，研细，取约2g，精密称定，精密加入乙醇100ml，照醇溶性浸出物测定法（中国药典 通则2201）项下的热浸法测定，不得少于5.0%。

【含量测定】　氨基酸　照高效液相色谱法（中国药典 通则0512）测定。

色谱条件与系统适用性试验　以十八烷基硅烷键合硅胶为填充剂；以乙腈-0.1mol/L醋酸钠溶液（用醋酸调节pH值至6.5）（7∶93）的混合溶液为流动相A，以乙腈-水（4∶1）的混合溶液为流动相B，按下表梯度洗脱；柱温为43℃；检测波长为254nm。理论板数按L-羟脯氨酸峰计应不低于4000。

时间（分钟）	流动相 A（%）	流动相 B（%）
0	100	0
11	93	7
13.9	88	12
14	85	15
29	66	34
30	0	100

对照品溶液的制备　取L-羟脯氨酸、甘氨酸、丙氨酸、脯氨酸对照品适量，精密称定，加0.1mol/L盐酸溶液制成每1ml分别含L-羟脯氨酸80μg、甘氨酸0.16mg、丙氨酸70μg、脯氨酸0.12mg的混合溶液，即得。

供试品溶液的制备　取本品适量，研细，取约0.3g，精密称定，置25ml量瓶中，加0.1mol/L盐酸溶液20ml，超声处理（功率500W，频率40kHz）30分钟，放冷，用0.1mol/L盐酸溶液稀释至刻度，摇匀。精密量取2ml，置氨基酸水解管中，加盐酸2ml，150℃水解1小时，放冷，移至蒸发皿中，用水10ml分次洗涤，洗液并入蒸发皿中，蒸干，残渣加0.1mol/L盐酸溶液使溶解，并分次转移至25ml量瓶中，用0.1mol/L盐酸溶液稀释至刻度，摇匀，即得。

测定法　精密量取上述对照品溶液和供试品溶液各5ml，分别置25ml量瓶中，

各加0.1mol/L异硫氰酸苯酯（PITC）的乙腈溶液2.5ml，1mol/L三乙胺的乙腈溶液2.5ml，摇匀，室温放置1小时后，用50%乙腈稀释至刻度，摇匀。取10ml，加正己烷10ml，振摇，放置10分钟，取下层溶液，滤过，取续滤液，分别精密吸取衍生化后的对照品溶液与供试品溶液各2μl，注入液相色谱仪，测定，即得。

本品每1g含L-羟脯氨酸（$C_5H_9NO_3$）应为48~110mg；含甘氨酸（$C_2H_5NO_2$）应为96~220mg；含丙氨酸（$C_3H_7NO_2$）应为39~90mg；含脯氨酸（$C_5H_9NO_2$）应为55~130mg。

特征多肽 照高效液相色谱-质谱法（中国药典 通则0512和通则0431）测定。

色谱、质谱条件与系统适用性试验 以十八烷基硅烷键合硅胶为填充剂（柱长内径为2.1mm），以乙腈为流动相A，以0.1%甲酸溶液为流动相B，按下表梯度洗脱，流速为0.3ml/min。理论板数按驴源多肽A1峰计应不低于4000。

时间（分钟）	流动相 A（%）	流动相 B（%）
0	50	95
25	20	80
40	50	50

采用三重四极杆质谱检测器，电喷雾离子化（ESI）正离子模式下多反应监测（MRM），监测离子对见下表：

测定成分	定量离子对m/z	定性离子对m/z
驴源多肽A1	469.25（双电荷）→712.30	469.25（双电荷）→783.40
驴源多肽A2	618.35（双电荷）→779.40	618.35（双电荷）→850.40

对照品溶液的制备 取驴源多肽A1对照品、驴源多肽A2对照品适量，精密称定，加1%碳酸氢铵溶液制成每1ml各含2.5μg的混合溶液，即得。

供试品溶液的制备 取本品适量，研细，取约0.lg，精密称定，置50ml量瓶中，加1%碳酸氢铵溶液40ml，超声处理（功率250W，频率40kHz）30分钟，用1%碳酸氢铵溶液稀释至刻度，摇匀。精密量取1ml至5ml量瓶中，加胰蛋白酶溶液（取序列分析级胰蛋白酶，加1%碳酸氢铵溶液制成每1ml中含1mg的溶液，临用前新制）1ml，用1%碳酸氢铵溶液稀释至刻度，摇匀，37℃恒温酶解12小时，滤过，取续滤液，即得。

测定法 精密量取对照品溶液1ml、2ml、5ml、10ml、20ml和25ml，分别置50ml量瓶中，用1%碳酸氢铵溶液稀释至刻度，制成标准曲线溶液。分别精密吸取不同浓度的标准曲线溶液与供试品溶液各5μl，注入高效液相色谱-质谱联用仪，以对照品峰面积为纵坐标，对照品浓度为横坐标制备标准曲线。从标准曲线读出

供试品溶液中相当于驴源多肽A1和驴源多肽A2的量，计算即得。

本品每 1g 含特征多肽以驴源多肽 A1（$C_{41}H_{68}N_{12}O_{13}$）和驴源多肽 A2（$C_{51}H_{82}N_{18}O_{18}$）的总量计应为 0.8~3.0mg。

【规格】 每1g配方颗粒相当于饮片0.9g

【贮藏】 密封。

江苏省药品监督管理局

中药配方颗粒标准

JS-YBZ-2022310

白药子配方颗粒

Baiyaozi Peifangkeli

【来源】 本品为防己科植物头花千金藤 *Stephania cepharantha* Hayata 的干燥块根经炮制并按标准汤剂的主要质量指标加工制成的配方颗粒。

【制法】 取白药子饮片 7100g，加水煎煮，滤过，滤液浓缩成清膏（出膏率为 8~14%），干燥（或干燥、粉碎），加辅料适量，混匀，制粒，制成 1000g，即得。

【性状】 本品为浅黄色至棕黄色颗粒；气微，味苦。

【鉴别】 取本品 0.5g，研细，加甲醇 10ml，超声处理 20 分钟，滤过，滤液蒸干，残渣加甲醇 1ml 使溶解，作为供试品溶液。另取白药子对照药材 0.5g，同法制成对照药材溶液。取千金藤素对照品，加甲醇制成每 1ml 含 1mg 的溶液，作为对照品溶液。照薄层色谱法（中国药典 通则 0502）试验，吸取上述对照品溶液、对照药材溶液和供试品溶液各 10μl，分别点于同一硅胶 G 薄层板上，以三氯甲烷-甲醇（8∶1)为展开剂，展开，取出，晾干，碘熏至斑点显色清晰。供试品色谱中，在与对照品色谱和对照药材色谱相应的位置上，显相同颜色的斑点。

【特征图谱】 照高效液相色谱法（中国药典 通则 0512）测定。

色谱条件与系统适用性试验 以十八烷基硅烷键合硅胶为填充剂；以甲醇为流动相 A，以 0.1%三乙胺溶液为流动相 B，按下表梯度洗脱；柱温为 30℃；检测波长为 282nm。理论板数按千金藤素峰计应不低于 5000。

时间（分钟）	流动相 A（%）	流动相 B（%）
0	40	60
10	47	53
18	58	42
24	58	42
30	75	25
40	75	25
60	90	10

参照物溶液的制备　取白药子对照药材 1.0g，加水 30ml，煮沸后加热回流 30 分钟，放冷，摇匀，滤过，取续滤液，作为对照药材参照物溶液。另取千金藤素对照品适量，精密称定，加甲醇制成每 1ml 含 25μg 的溶液，作为对照品参照物溶液。

供试品溶液的制备　取本品适量，研细，取约 0.25g，精密称定，置具塞锥形瓶中，精密加 70%甲醇 25ml，密塞，称定重量，超声处理（功率 250W，频率 40kHz）40 分钟，放冷，再称定重量，用 70%甲醇补足减失的重量，摇匀，滤过，取续滤液，即得。

测定法　分别精密吸取参照物溶液与供试品溶液各 10μl，注入液相色谱仪，测定，即得。

供试品色谱中应呈现对照药材参照物色谱图中的 6 个保留时间相对应的特征峰，峰 6 应与对照品参照物色谱峰的保留时间相对应。峰 1～5 与 S 峰（峰 6）的相对保留时间依次约为：0.26、0.45、0.51、0.80、0.93。

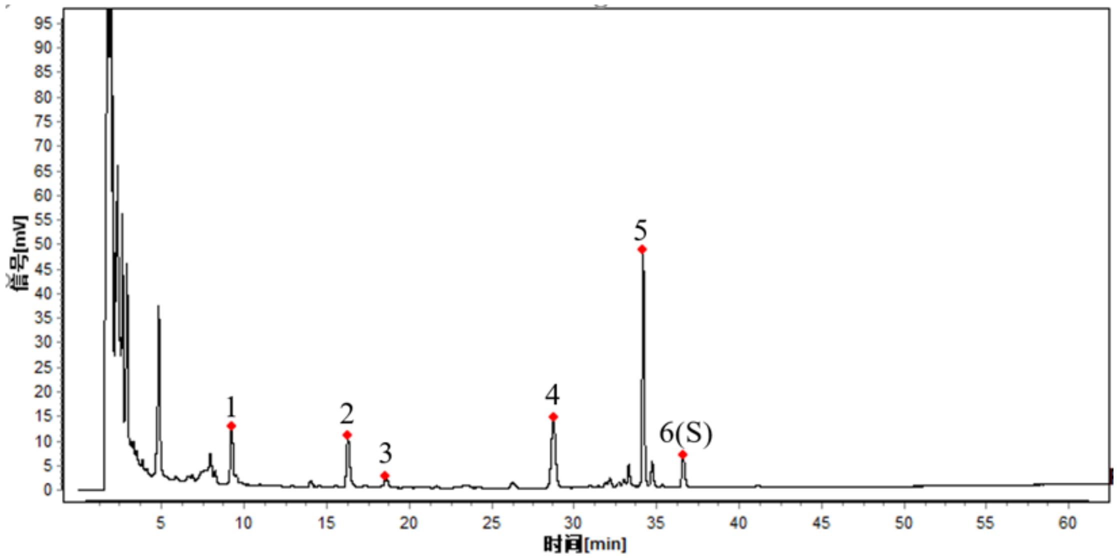

对照特征图谱

峰 6（S）：千金藤素

色谱柱：XBridge Shield RP18（250mm×4.6mm，5μm）

【检查】　应符合颗粒剂（中国药典　通则 0104）项下有关的各项规定。

【浸出物】　照醇溶性浸出物测定法（中国药典　通则 2201）项下的热浸法测定，不得少于 12.0%。

【含量测定】　照高效液相色谱法（中国药典　通则 0512）测定。

色谱条件与系统适用性试验　同【特征图谱】项。

对照品溶液的制备　同【特征图谱】项下的对照品参照物溶液。

供试品溶液的制备　同【特征图谱】项。

测定法　精密吸取对照品溶液与供试品溶液各 10μl，注入液相色谱仪，测定，即得。

本品每 1g 含千金藤素（$C_{37}H_{38}N_2O_6$）应为 0.5~1.1mg。

【规格】　每 1g 配方颗粒相当于饮片 7.1g

【贮藏】　密封，置阴凉干燥处。

江苏省药品监督管理局

中药配方颗粒标准

JS-YBZ-2022311

炒僵蚕配方颗粒

Chaojiangcan Peifangkeli

【来源】本品为蚕蛾科昆虫家蚕*Bombyx mori* Linnaeus 4~5龄的幼虫感染（或人工接种）白僵菌*Beauveria bassiana*(Bals.)Vuillant而致死的干燥体经炮制并按标准汤剂的主要质量指标加工制成的配方颗粒。

【制法】取炒僵蚕饮片3000g，加水煎煮，滤过，滤液浓缩成清膏（出膏率为17~27%），加入辅料适量，干燥（或干燥，粉碎），再加入辅料适量，混匀，制粒，制成1000g，即得。

【性状】本品为浅黄棕色至黄褐色的颗粒；气腥，味淡、微咸。

【鉴别】（1）取本品适量，研细，取0.2g，加50%乙醇5ml，超声处理10分钟，滤过，滤液作为供试品溶液。另取僵蚕对照药材1g，加水50ml，煮沸30分钟，滤过，滤液蒸干，残渣加50%乙醇5ml，同法制成对照药材溶液。照薄层色谱法（中国药典 通则0502）试验，吸取供试品溶液3μl、对照药材溶液4μl，分别点于同一硅胶G薄层板上，以正丁醇-冰醋酸-水（4∶1∶5）的上层溶液为展开剂，展开，取出，晾干，喷以0.5%茚三酮乙醇溶液，在105℃加热至斑点显色清晰。供试品色谱中，在与对照药材色谱相应的位置上，显相同颜色的斑点。

（2）取本品适量，研细，取0.5g，加1%碳酸氢铵溶液50ml，超声处理30分钟，用微孔滤膜滤过，取续滤液500μl，置进样瓶中，加胰蛋白酶溶液300μl（取序列分析用胰蛋白酶，加1%碳酸氢铵溶液制成每1ml中含1mg的溶液，临用时配制），摇匀，37℃恒温酶解12小时，作为供试品溶液。另取僵蚕多肽Ⅰ对照品、僵蚕多肽Ⅱ对照品，加1%碳酸氢铵溶液制成每1ml各含2μg的混合溶液，作为对照品溶液。照高效液相色谱法-质谱法（中国药典 通则0512和通则0431）试验，以十八烷基硅烷键合硅胶为填充剂（柱长为150mm，内径为2.1mm，粒径为1.7~1.9μm），以乙腈为流动相A，以0.1%甲酸溶液为流动相B，按下表梯度洗脱；流速为0.3ml/min。采用质谱检测器，电喷雾正离子模式（ESI+），进行多反应监测（MRM），选择质荷比（m/z）823（双电荷）→1070和m/z 823（双电荷）→1345

作为僵蚕多肽Ⅰ的检测离子对；质荷比（m/z）637（三电荷）→825和m/z 637（三电荷）→926作为僵蚕多肽Ⅱ的检测离子对。取上述混合对照品溶液，进样5μl，按上述检测离子对测定的MRM色谱峰的信噪比均应大于3:1。

时间（分钟）	流动相 A（%）	流动相 B（%）
0	3	97
3	3	97
8	5	95
10	5	95
18	7	93
19	90	10
21	90	10

吸取供试品溶液5μl，注入高效液相色谱-质谱联用仪，测定。以质荷比（m/z）823（双电荷）→1070、m/z 823（双电荷）→1345和质荷比（m/z）637（三电荷）→825、m/z 637（三电荷）→926离子对提取的供试品离子流色谱中，应同时呈现与相应对照品色谱保留时间一致的色谱峰。

【特征图谱】 照高效液相色谱法（中国药典 通则0512）测定。

色谱条件与系统适用性试验 以十八烷基硅烷键合硅胶为填充剂；以甲醇为流动相A，以水为流动相B，按下表梯度洗脱；流速为0.35ml/min；柱温为40℃；检测波长为260nm。理论板数按腺苷峰计应不低于5000。

时间（分钟）	流动相 A（%）	流动相 B（%）
0	0	100
5	0	100
12	5	95
20	25	75

参照物溶液的制备 取僵蚕对照药材1g，加水50ml，超声处理（功率250W，频率40kHz）1小时，置100℃水浴加热5分钟，放冷，摇匀，滤过，取续滤液，作为对照药材参照物溶液。另取腺嘌呤对照品、鸟苷对照品、腺苷对照品，加10%甲醇制成每1ml含腺嘌呤10μg、鸟苷25μg、腺苷20μg的混合溶液，作为对照品参照物溶液。

供试品溶液的制备 取本品适量，研细，取约0.5g，精密称定，置具塞锥形瓶中，精密加入10%乙醇25ml，称定重量，加热回流30分钟，放冷，再称定重量，用10%乙醇补足减失的重量，摇匀，滤过，取续滤液，即得。

测定法 分别精密吸取参照物溶液与供试品溶液各1μl，注入液相色谱仪，测定，即得。

供试品色谱图中应呈现与对照药材参照物色谱图中5个保留时间相对应的特

征峰，峰1～2、峰4应分别与相应对照品参照物峰的保留时间相对应。峰3、峰5与S峰（峰4）的相对保留时间依次约为：0.92、1.20。

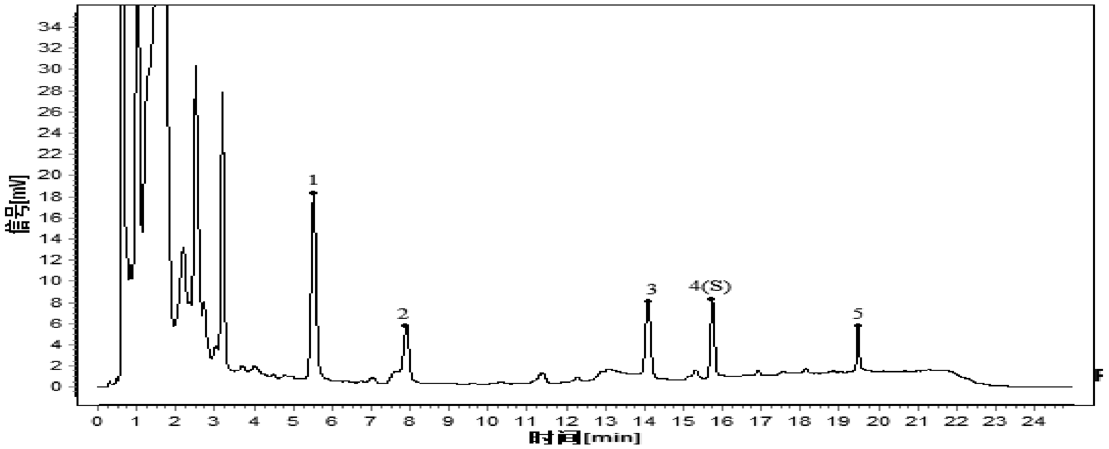

对照特征图谱

峰1：腺嘌呤　峰2：鸟苷　峰4（S）：腺苷

色谱柱：Triart C18（100mm×2.1mm，1.9μm）

【检查】　黄曲霉毒素　照黄曲霉毒素测定法（中国药典　通则2351）测定。

本品每1kg含黄曲霉毒素B1不得过5μg；含黄曲霉毒素G2、黄曲霉毒素G1、黄曲霉毒素B2和黄曲霉毒素B1的总量不得过10μg。

其他　应符合颗粒剂（中国药典　通则0104）项下有关的各项规定。

【浸出物】　取本品适量，研细，取约2g，精密称定，精密加入乙醇100ml，照醇溶性浸出物测定法（中国药典　通则2201）项下的热浸法测定，不得少于8.0%。

【含量测定】　照高效液相色谱法（中国药典　通则0512）测定。

色谱条件与系统适用性试验　同【特征图谱】项。

对照品溶液的制备　取腺嘌呤、腺苷对照品适量，精密称定，加10%乙醇溶液制成每1ml各含10μg的混合溶液，即得。

供试品溶液的制备　同【特征图谱】项。

测定法　分别精密吸取对照品溶液与供试品溶液各1μl，注入液相色谱仪，测定，即得。

本品每1g含腺嘌呤（$C_5H_5N_5$）和腺苷（$C_{10}H_{13}N_5O_4$）的总量应为0.5~2.6mg。

【规格】　每1g配方颗粒相当于饮片3g

【贮藏】　密封。

江苏省药品监督管理局

中药配方颗粒标准

JS-YBZ-2022312

赤茯苓配方颗粒

Chifuling Peifangkeli

【来源】 本品为多孔菌科真菌茯苓 *Poria cocos* (Schw.) Wolf 干燥菌核近外皮部的淡红色部分经炮制并按标准汤剂的主要质量指标加工制成的配方颗粒。

【制法】 取赤茯苓饮片 5500g，加水煎煮，滤过，滤液浓缩成清膏（出膏率为 12~18%），干燥（或干燥、粉碎），加辅料适量，混匀，制粒，制成 1000g，即得。

【性状】 本品为浅棕色至棕褐色颗粒，气微，味酸。

【鉴别】 取本品 2g，研细，加三氯甲烷 25ml，超声处理 30 分钟，滤过，滤液蒸干，残渣加甲醇 1ml 使溶解，作为供试品溶液。另取赤茯苓对照药材 1g，同法制成对照药材溶液。再取茯苓酸对照品，加甲醇制成每 1ml 含 2mg 的溶液，作为对照品溶液。照薄层色谱法（中国药典 通则 0502）试验，吸取上述三种溶液各 5μl，分别点于同一硅胶 G 薄层板上，以甲苯-乙酸乙酯-甲酸（20：5：0.5）为展开剂，展开，取出，晾干，喷以 10%硫酸乙醇溶液，在 105℃加热至斑点显色清晰，置紫外光灯（365nm）下检视。供试品色谱中，在与对照品及对照药材色谱相应的位置上，显相同颜色的荧光斑点。

【特征图谱】 照高效液相色谱法（中国药典 通则 0512）测定。

色谱条件与系统适用性试验 以十八烷基硅烷键合硅胶为填充剂（柱长为 100mm，内径为 2.1mm，粒径为 1.7μm）；以甲醇为流动相 A，以乙腈为流动相 B，以 0.1%磷酸为流动相 C，按下表梯度洗脱；流速为 0.2ml/min；柱温为 40℃；检测波长为 210nm、242nm。理论板数按茯苓酸峰计应不低于 5000。

时间（分钟）	流动相 A（%）	流动相 B（%）	流动相 C（%）
0	3	55	42
12	3	85	12
16	3	97	0
18	0	100	0

参照物溶液的制备 取赤茯苓对照药材 0.5g，加甲醇 25ml，超声处理 30 分钟，放冷，滤过，取续滤液，作为对照药材参照物溶液。另取茯苓酸对照品适量，

精密称定，加甲醇制成每 1ml 含 5μg 的溶液，作为对照品参照物溶液。

供试品溶液的制备 取本品适量，研细，取约 0.5g，精密称定，置具塞锥形瓶中，精密加甲醇 25ml，密塞，称定重量，超声处理（功率 390W，频率 40kHz）30 分钟，放冷，再称定重量，用甲醇补足减失的重量，摇匀，滤过，取续滤液，即得。

测定法 分别精密吸取参照物溶液与供试品溶液各 5μl，注入液相色谱仪，测定，即得。

供试品色谱图（242nm）中应呈现与对照药材参照物色谱峰图中的 5 个保留时间相对应的特征峰，峰 3（242nm）应与对照品参照物峰（210nm）的保留时间相近。峰 1～2、峰 4～5 与 S 峰（峰 3）的相对保留时间依次约为：0.45、0.62、1.27、1.39。

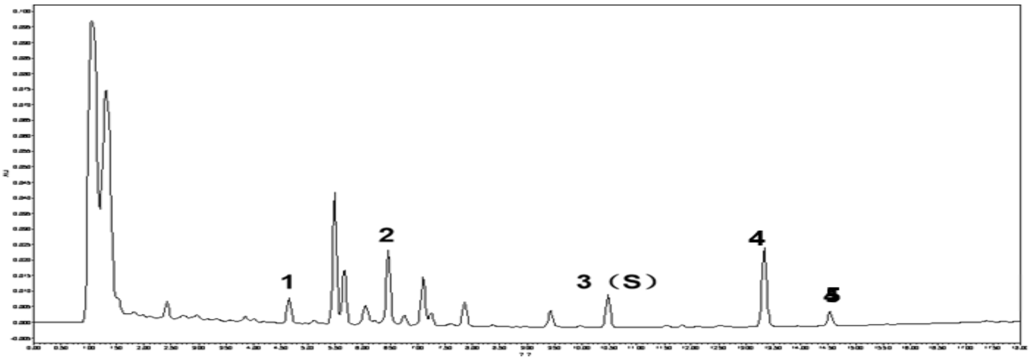

对照特征图谱

色谱柱：BEH C18（100mm×2.1mm，1.7 μm）

【检查】 应符合颗粒剂（中国药典 通则 0104）项下有关的各项规定。

【浸出物】 照醇溶性浸出物测定法（中国药典 通则 2201）项下的热浸法测定 不得少于 24.0%。

【含量测定】 照高效液相色谱法（中国药典 通则 0512）测定。

色谱条件与系统适用性试验 检测波长为 210nm，其余同【特征图谱】项。

对照品溶液的制备 同【特征图谱】项下的对照品参照物溶液。

供试品溶液的制备 同【特征图谱】项。

测定法 分别精密吸取对照品溶液与供试品溶液各 5μl，注入液相色谱仪，测定，即得。

本品每 1g 含茯苓酸（$C_{33}H_{52}O_5$）应为 0.02~0.11mg。

【规格】 每 1g 配方颗粒相当于饮片 5.5g

【贮藏】 密封。

江苏省药品监督管理局

中药配方颗粒标准

JS-YBZ-2022313

代代花配方颗粒

Daidaihua Peifangkeli

【来源】 本品为芸香科植物代代花（*Citrus aurantium* L.*var.amara* Engl）的干燥花蕾经炮制并按标准汤剂的主要质量指标加工制成的配方颗粒。

【制法】 取代代花饮片 2800g，加水煎煮，滤过，滤液浓缩成清膏（出膏率为 29~35%），干燥（或干燥、粉碎），加入辅料适量，混匀，制粒，制成 1000g，即得。

【性状】 本品为淡黄色至黄棕色颗粒；气香，味微苦。

【鉴别】 取本品粉末 0.3g，加甲醇 20ml，超声处理 30 分钟，滤过，滤液蒸干，残渣加甲醇 2ml 使溶解，作为供试品溶液。另取柚皮苷对照品，加甲醇制成每 1ml 含 1mg 的溶液，作为对照品溶液。照薄层色谱法（中国药典 通则 0502）试验，吸取上述两种溶液各 2μl，分别点于同一硅胶 G 薄层板上，以乙酸乙酯-甲醇-水（100：17：13）为展开剂，展至约 5cm，取出，晾干，再以甲苯-乙酸乙酯-甲酸-水（20：10：1：1）的上层溶液为展开剂，展至约 8cm，取出，晾干，喷以三氯化铝试液，置紫外光灯（365nm）下检视。供试品色谱中，在与对照品色谱相应的位置上，显相同颜色的荧光斑点。

【特征图谱】 照高效液相色谱法（中国药典 通则 0512）测定。

色谱条件与系统适用性试验 以十八烷基硅烷键合硅胶为填充剂；以乙腈为流动相 A，以 0.1%甲酸为流动相 B，按下表梯度洗脱；柱温为 35℃；流速为 0.35ml/min；检测波长为 283nm。理论板数按柚皮苷峰计应不低于 10000。

时间（分钟）	流动相 A（%）	流动相 B（%）
0	12	88
12	16	84
18	19	81
21	26	74

参照物溶液的制备 取柚皮苷对照品适量，精密称定，加甲醇制成每 1ml 含柚皮苷 0.2mg 的溶液，作为对照品参照物溶液。

供试品溶液的制备　取本品适量，研细，取约 0.1g，精密称定，置具塞锥形瓶中，精密加入 70%甲醇 25ml，密塞，称定重量，超声处理（功率 250W，频率 40kHz）10 分钟，放冷，再称定重量，用 70%甲醇补足减失的重量，摇匀，滤过，取续滤液，即得。

测定法　精密吸取参照物溶液与供试品溶液各 1μl，注入液相色谱仪，测定，即得。

供试品色谱图中应呈现 5 个特征峰，峰 3 应与对照品参照物峰的保留时间相对应。峰 1～2、峰 4～5 与 S 峰（峰 3）的相对保留时间依次约为：0.66、0.87、1.15、1.29。

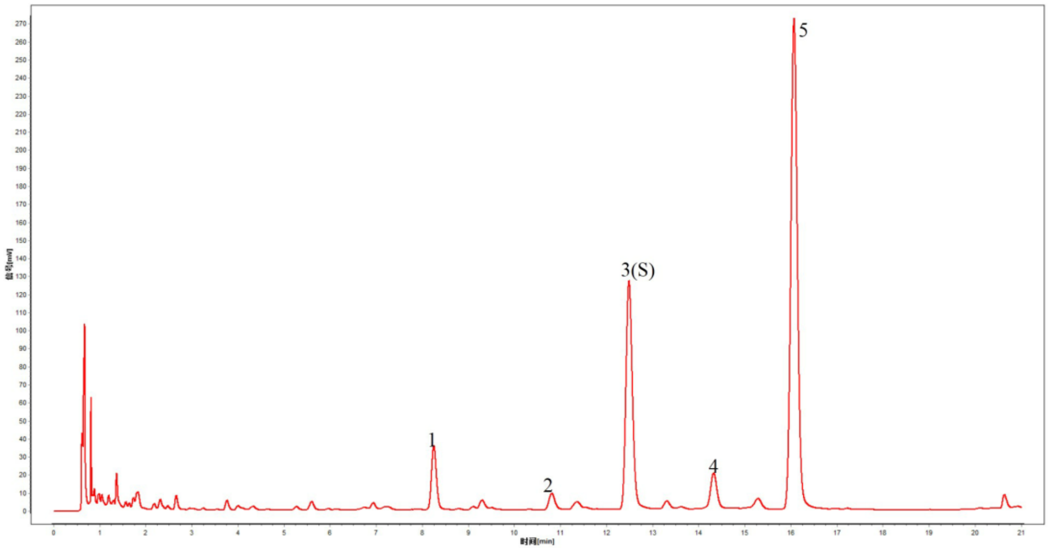

对照特征图谱

峰 2：芸香柚皮苷　峰 3（S）：柚皮苷　峰 4：橙皮苷　峰 5：新橙皮苷

色谱柱：BEH C18（100mm×2.1mm，1.7μm）

【检查】　应符合颗粒剂（中国药典 通则 0104）项下有关的各项规定。

【浸出物】　照醇溶性浸出物测定法（中国药典 通则 2201）项下的热浸法测定，不得少于 20.0%。

【含量测定】　照高效液相色谱法（中国药典 通则 0512）测定。

色谱条件与系统适用性试验　同【特征图谱】项。

对照品溶液的制备　同【特征图谱】项下的对照品参照物溶液。

供试品溶液的制备　同【特征图谱】项。

测定法　分别精密吸取对照品溶液与供试品溶液各 1μl，注入液相色谱仪，测定，即得。

本品每 1g 含柚皮苷（$C_{27}H_{32}O_{14}$）应为 46~66mg。

【规格】 每 1g 配方颗粒相当于饮片 2.8g

【贮藏】 密封。

江苏省药品监督管理局

中药配方颗粒标准

JS-YBZ-2022314

胆南星配方颗粒

Dannanxing Peifangkeli

【来源】 本品为制天南星的细粉与牛、羊或猪胆汁经加工而成，或为生天南星细粉与牛、羊或猪胆汁经发酵加工而成经炮制并按标准汤剂的主要质量指标加工制成的配方颗粒。

【制法】 取胆南星饮片4300g，加水煎煮，滤过，滤液浓缩成清膏（出膏率为12~23%），干燥（或干燥，粉碎），加入辅料适量，混匀，制粒，制成1000g，即得。

【性状】 本品为黄色至棕黄色颗粒；气微腥，味苦。

【鉴别】 （1）取本品0.2g，研细，加水5ml，振摇，滤过，取滤液2ml置试管中，加新制的糠醛溶液（1→100）0.5ml，沿管壁加硫酸2ml，两液接界处即显棕红色环。

（2）取本品2.0g，研细，加甲醇30ml，超声处理30分钟，滤过，滤液蒸干，残渣加甲醇1ml使溶解，作为供试品溶液。另取胆酸对照品，加甲醇制成每1ml含1mg的溶液，作为对照品溶液。照薄层色谱法（中国药典 通则0502）试验，吸取上述供试品溶液10μl、对照品溶液2μl，分别点于同一硅胶G薄层板上，以正己烷-乙酸乙酯-醋酸-甲醇（20:25:2:3）上层溶液为展开剂，展开，取出，晾干，喷以10%磷钼酸乙醇溶液，在105℃加热至斑点显色清晰。供试品色谱中，在与对照品色谱相应的位置上，显相同颜色的斑点。

【特征图谱】 照高效液相色谱法（中国药典 通则0512）测定。

色谱条件与系统适用性试验 以十八烷基硅烷键合硅胶为填充剂；以乙腈为流动相A，以0.1%磷酸溶液为流动相B，按下表梯度洗脱；流速为0.9ml/min；检测波长为0~23分钟，260nm；23~45分钟，284nm。理论板数按尿嘧啶峰计应均不低于5000。

时间（分钟）	流动相 A（%）	流动相 B（%）
0	0	100
8	0	100
13	3	97
25	10	90
40	25	75
45	40	60

参照物溶液的制备 取尿嘧啶对照品适量，精密称定，加 10%甲醇制成每 1ml 含 10μg 的溶液，作为对照品参照物溶液。

供试品溶液的制备 取本品适量，研细，取约 0.1g，置具塞锥形瓶中，精密加入 10%甲醇 20ml，密塞，称定重量，超声处理（功率 250W，频率 40kHz）30 分钟，取出，放冷，再称定重量，用 10%甲醇补足减失的重量，摇匀，滤过，取续滤液，即得。

测定法 分别精密吸取参照物溶液 10μl、供试品溶液 20μl，注入液相色谱仪，测定，即得。

供试品色谱图中应呈现 8 个特征峰，峰 2 应与对照品参照物峰的保留时间相对应。峰 1、峰 3～8 与 S 峰（峰 2）的相对保留时间约为：0.92、1.34、1.99、2.87、3.22、3.35、4.54。

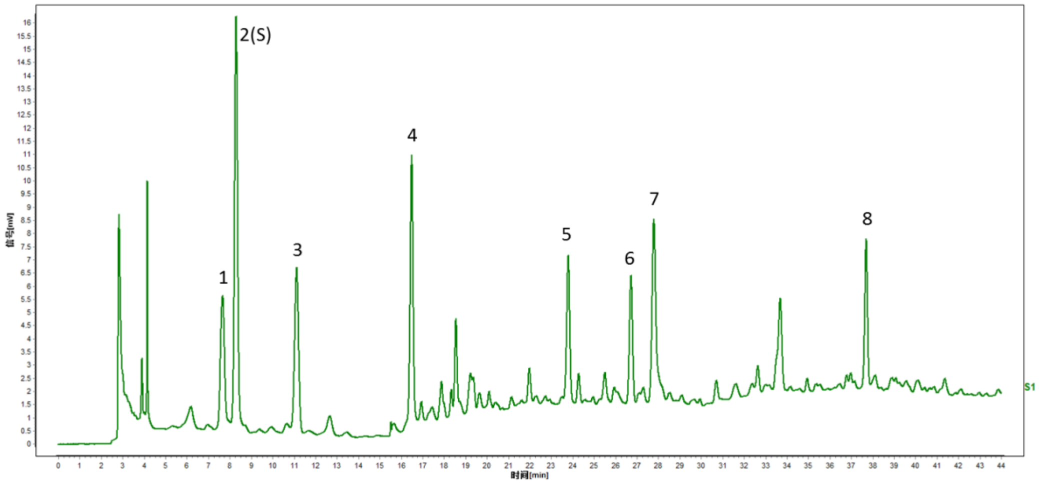

对照特征图谱

峰 2（S）：尿嘧啶

色谱柱：Shim-pack GIST C18-AQ（250mm×4.6mm，5μm）

【检查】 应符合颗粒剂（中国药典 通则 0104）项下有关的各项规定。

【浸出物】 照醇溶性浸出物测定法（中国药典 通则 2201）项下的热浸法测定，用乙醇作溶剂，应不得少于 5.0%。

【含量测定】 照高效液相色谱法（中国药典 通则 0512）测定。

色谱条件与系统适用性试验 同【特征图谱】项。

对照品溶液的制备 同【特征图谱】项下的对照品参照物溶液。

供试品溶液的制备 同【特征图谱】项。

测定法 分别精密吸取对照品溶液 10μl、供试品溶液 20μl，注入液相色谱仪，测定，即得。

本品每 1g 含尿嘧啶（$C_4H_4N_2O_2$）含量应为 0.2~1.5mg。

【规格】 每 1g 配方颗粒相当于饮片 4.3g

【贮藏】 密封。

江苏省药品监督管理局

中药配方颗粒标准

JS-YBZ-2022315

伏龙肝配方颗粒

Fulonggan Peifangkeli

【来源】 本品为久经柴草熏烧的灶心土经炮制并按标准汤剂的主要质量指标加工制成的配方颗粒。

【制法】 取伏龙肝饮片5500g，加水煎煮，滤过，滤液浓缩成清膏（出膏率为10~18%），干燥（或干燥，粉碎），加入辅料适量，混匀，制粒，制成1000g，即得。

【性状】 本品为淡红褐色至红褐色颗粒，微具烟熏气，味淡。

【鉴别】 取本品1g，研细，加稀盐酸10ml，离心，取上清液1ml，加亚铁氰化钾试液，即生成蓝色沉淀。

【检查】 应符合颗粒剂（中国药典 通则0104）项下有关的各项规定。

【含量测定】 **二氧化硅** 取本品适量，研细，取约1g，精密称定，置于蒸发皿中，精密加入盐酸（1：1）25ml加热分解后，水浴蒸干。转移至120℃干燥1小时，放冷，精密加入盐酸（1：1）10ml，水浴加热20分钟，取出，放凉，加水稀释至100ml，用无灰滤纸滤过，用3%盐酸和温水洗涤至洗液呈中性为止。滤渣连同滤纸移至同一恒重的坩埚内，干燥，炽灼至恒重。根据残渣重量，计算本品中二氧化硅的含量（%）。

氧化铁、氧化铝 取上法所得滤液，加入浓硝酸6滴，加热煮沸，放凉后加入10g氯化铵，使完全溶解，加适量氨水于水浴中加热至沉淀完全。用无灰滤纸滤过，用水洗涤至洗液呈中性为止。滤渣连同滤纸移至同一恒重的坩埚内，干燥，炽灼至恒重。根据残渣重量，计算本品中氧化铁和氧化铝的含量（%）。

本品含二氧化硅（SiO_2）应为21～36%，含氧化铁（Fe_2O_3）和氧化铝（$A1_20_3$）的总量应为1.9～5.0%。

【规格】 每1g配方颗粒相当于饮片5.5g

【贮藏】 密封。

江苏省药品监督管理局

中药配方颗粒标准

JS-YBZ-2022316

海螵蛸（金乌贼）配方颗粒

Haipiaoxiao（Jinwuzei）Peifangkeli

【来源】 本品为乌贼科金乌贼 *Sepia esculenta* Hoyle 的干燥内壳经炮制并按标准汤剂的主要质量指标加工制成的配方颗粒。

【制法】 取海螵蛸（金乌贼）饮片 10000g，加水煎煮，滤过（出膏率为 0.5~5.5%），加入辅料适量，混匀，浓缩成清膏，干燥（或干燥，粉碎），再加入辅料适量，混匀，制粒，制成 1000g，即得。

【性状】 本品为类白色至浅灰黄色的颗粒；气腥，味咸。

【鉴别】 （1）取本品 0.2g，研细，加稀盐酸，产生气泡。

（2）取本品 2g，研细，加稀盐酸 5ml，待溶解后，滤过，滤液显钙盐（中国药典 通则 0301）的鉴别反应。

（3）取本品 0.25g，研细，加稀盐酸 15ml，超声处理 30 分钟，用氢氧化钠试液调节 pH 值至 12，静置 10 分钟，离心，取沉淀置水解管中，加 6mol/L 盐酸 10ml，150℃水解 1 小时，放冷，离心，取上清液，蒸干，残渣加甲醇 2ml 使溶解，作为供试品溶液。另取海螵蛸（金乌贼）对照药材 0.5g，加水 50ml，加热溶解 30 分钟，趁热用纱布滤过，滤液蒸干，残渣加稀盐酸 15ml，同法制成对照药材溶液。照薄层色谱法(中国药典 通则 0502)试验，吸取供试品溶液 2μl、对照药材溶液 1μl，分别点于同一硅胶 G 薄层板上，以正丁醇-冰醋酸-水-丙酮-无水乙醇-0.5%茚三酮丙酮溶液(40:14:12:5:4:4)为展开剂，展开，取出，晾干，在 105℃加热至斑点显色清晰。供试品色谱中，在与对照药材色谱相应的位置上，显相同颜色的斑点。

【检查】 **重金属及有害元素** 照铅、镉、砷、汞、铜测定法（中国药典 通则 2321 原子吸收分光光度法或电感耦合等离子体质谱法）测定，铅不得过 5mg/kg；镉不得过 5mg/kg；砷不得过 100mg/kg；汞不得过 0.2mg/kg；铜不得过 50mg/kg。

其他 除溶化性外，应符合颗粒剂（中国药典 通则 0104）项下有关的各项规

定。

【含量测定】 **碳酸钙**　取本品适量，研细，取约 0.1g，精密称定，置锥形瓶中，加稀盐酸 10ml，加热使溶解，加水 20ml 与甲基红指示剂 1 滴，滴加 10%氢氧化钾试液至溶液显浅黄色，继续多加 5ml，再加钙黄绿素指示剂少量，用乙二胺四醋酸二钠滴定液（0.05mol/L）滴定至溶液黄绿色荧光消失，并显橙色。每 1ml 乙二胺四醋酸二钠滴定液（0.05mol/L）相当于 5.004mg 的碳酸钙（$CaCO_3$）。

本品每 1g 含碳酸钙（$CaCO_3$）应为 30~320mg。

总氮量　取本品适量，研细，取约 0.15g，精密称定，照氮测定法（中国药典通则 0704）测定。

本品每 1g 含总氮（N）应为 3.0~15.0mg。

【规格】 每 1g 配方颗粒相当于饮片 10g

【贮藏】 密封。

江苏省药品监督管理局

中药配方颗粒标准

JS-YBZ-2022317

海藻（羊栖菜）配方颗粒

Haizao（Yangqicai） Peifangkeli

【来源】 本品为马尾藻科植物羊栖菜 *Sargassum fusiforme*（Harv.）Setch. 的干燥藻体经炮制并按标准汤剂的主要质量指标加工制成的配方颗粒。

【制法】 取海藻（羊栖菜）饮片 5000g，加水煎煮，滤过，滤液浓缩成清膏（出膏率为 12~20%），干燥（或干燥，粉碎），加入辅料适量，混匀，制粒，制成 1000g，即得。

【性状】 本品为棕褐色至灰棕色颗粒；气微，味微咸。

【鉴别】 取本品粉末 3g，加乙酸乙酯 30ml，超声处理 30min，滤过，滤液蒸干，残渣加乙酸乙酯 1ml 使溶解，作为供试品溶液。另取海藻（羊栖菜）随行对照药材 2g，加水 50ml，煎煮 30min，滤过，滤液蒸干，残渣自“加乙酸乙酯 30ml”起，同法制成对照药材溶液。照薄层色谱法（中国药典 通则 0502）试验，吸取供试品溶液 20µl、对照药材溶液 15µl，分别点于同一硅胶 G 薄层板上，以甲苯-乙酸乙酯-甲酸（10：1：0.2）为展开剂，展开，取出，晾干，喷以 10%硫酸乙醇溶液，在 105℃加热至斑点显色清晰。供试品色谱中，在与对照药材色谱相应的位置上，显相同颜色的斑点。

【特征图谱】 照高效液相色谱法（中国药典 通则 0512）测定。

色谱条件与系统适用性试验 以十八烷基硅烷键合硅胶为填充剂；以乙腈为流动相 A，以 0.08%磷酸为流动相 B，按下表梯度洗脱；流速为 0.8ml/min；柱温为 35℃；检测波长为 265nm。理论塔板数按尿苷峰计应不低于 5000。

时间（分钟）	流动相 A(%)	流动相 B（%）
0	0	100
10	0	100
28	5	95
40	10	90

参照物溶液的制备 取尿苷对照品适量，精密称定，加 10%甲醇制成每 1ml 含 15µg 的溶液，作为对照品参照物溶液。

供试品溶液的制备 取本品适量，研细，取约 0.5g，精密称定，置具塞锥形

瓶中，精密加入10%甲醇10ml，称定重量，超声处理（功率250W，频率40kHz）30分钟，放冷，再称定重量，用10%甲醇补足减失的重量，离心，过滤，取续滤液，即得。

测定法　分别精密吸取参照物溶液与供试品溶液各10μl，注入液相色谱仪，测定，即得。

供试品色谱图中应呈现7个特征峰，峰6应与对照品参照物峰的保留时间相对应。峰1~5、峰7与S峰（峰6）的相对保留时间依次约为：0.29、0.55、0.65、0.74、0.93、1.37。

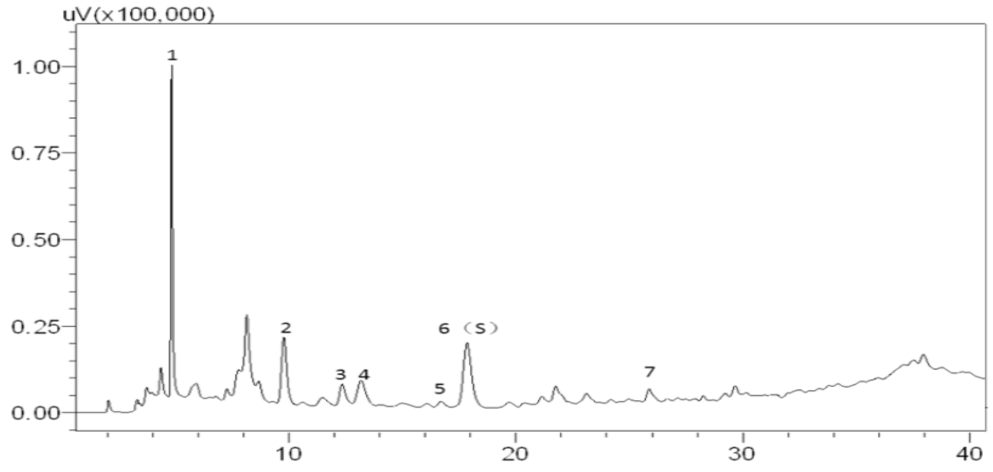

对照特征图谱

峰6（S）：尿苷

色谱柱：Shim-pack GIST C18-AQ（250mm×4.6mm，5μm）

【检查】 应符合颗粒剂（中国药典 通则0104）项下有关的各项规定。

【浸出物】 照醇溶性浸出物测定法（中国药典 通则2201）项下的热浸法测定，用乙醇作溶剂，应不得少于9.0%。

【含量测定】 照高效液相色谱法（中国药典 通则0512）测定。

色谱条件与系统适用性试验 同【特征图谱】项。

对照品溶液的制备 同【特征图谱】项下的对照品参照物溶液。

供试品溶液的制备 同【特征图谱】项。

测定法　分别精密吸取对照品溶液与供试品溶液各10μl，注入液相色谱仪，测定，即得。

本品每1g含尿苷（$C_9H_{12}N_2O_6$）应为0.15~0.35mg。

【规格】　每1g配方颗粒相当于饮片5g

【贮藏】　密封。

江苏省药品监督管理局

中药配方颗粒标准

JS-YBZ-2022318

黑芝麻配方颗粒

Heizhima Peifangkeli

【来源】本品为脂麻科植物脂麻 *Sesamum indicum* L .的干燥成熟种子经炮制并按标准汤剂的主要质量指标加工制成的配方颗粒。

【制法】取黑芝麻饮片 12500g，加水煎煮，滤过，滤液浓缩成清膏（出膏率为 5~8%），干燥（或干燥，粉碎），加入辅料适量，混匀，制粒，制成 1000g，即得。

【性状】本品为浅灰色至灰褐色颗粒；气微，味甘、有油香气。

【鉴别】取本品 1g，研细，加无水乙醇 10ml，超声处理 20 分钟，静置，取上清液作为供试品溶液。另取黑芝麻对照药材 0.5g，同法制成对照药材溶液。再取芝麻素对照品，加无水乙醇制成每 1ml 含 1mg 的溶液，作为对照品溶液。照薄层色谱法（中国药典 通则 0502）试验，吸取上述供试品溶液和对照药材溶液各 5μl、对照品溶液 2μl，分别点于同一硅胶 G 薄层板上，以环己烷-乙醚-乙酸乙酯（20：5.5：2.5）为展开剂，展开，取出，晾干，喷以 10%硫酸乙醇溶液，加热至斑点显色清晰。供试品色谱中，在与对照药材色谱和对照品色谱相应的位置上，显相同颜色的斑点。

【特征图谱】照高效液相色谱法（中国药典 通则 0512）测定。

色谱条件与系统适应性试验 以十八烷基硅烷键合硅胶为填充剂；以乙腈为流动相 A，以 0.1%磷酸溶液为流动相 B，按下表梯度洗脱；流速为 0.3ml/min；柱温为 35℃；检测波长为 210nm。理论板数按芝麻素峰计算应不低于 5000。

时间（min）	流动相 A（%）	流动相 B（%）
0	10	90
6	40	60
17	70	30
20	95	5
23	95	5

参照物溶液的制备 取黑芝麻对照药材 1g，置具塞锥形瓶中，加水 30ml，

加热回流 30 分钟，取出，放冷，摇匀，滤过，滤液蒸干，残渣加 70%甲醇溶解并转移至 10ml 量瓶中，加 70%甲醇至刻度，摇匀，滤过，取续滤液，作为对照药材参照物溶液。另取芝麻素对照品适量，精密称定，加甲醇制成每 1ml 含芝麻素 8μg 的溶液，作为对照品参照物溶液。

供试品溶液的制备 取本品适量，研细，取约 0.3g，精密称定，置具塞锥形瓶中，精密加入 70%甲醇 25ml，称定重量，超声处理（功率 250W，频率 40kHz）15 分钟，取出，放冷，再称定重量，用 70%甲醇补足减失的重量，摇匀，滤过，取续滤液，即得。

测定法 分别精密吸取参照物溶液与供试品溶液各 1μl，注入液相色谱仪，测定，即得。

供试品色谱图中应呈现与对照药材参照物色谱中 4 个保留时间相对应的特征峰，峰 3 应与对照品参照物峰的保留时间相对应。峰 1～2、峰 4 与 S 峰（峰 3）的相对保留时间依次约为：0.21、0.42、1.09。

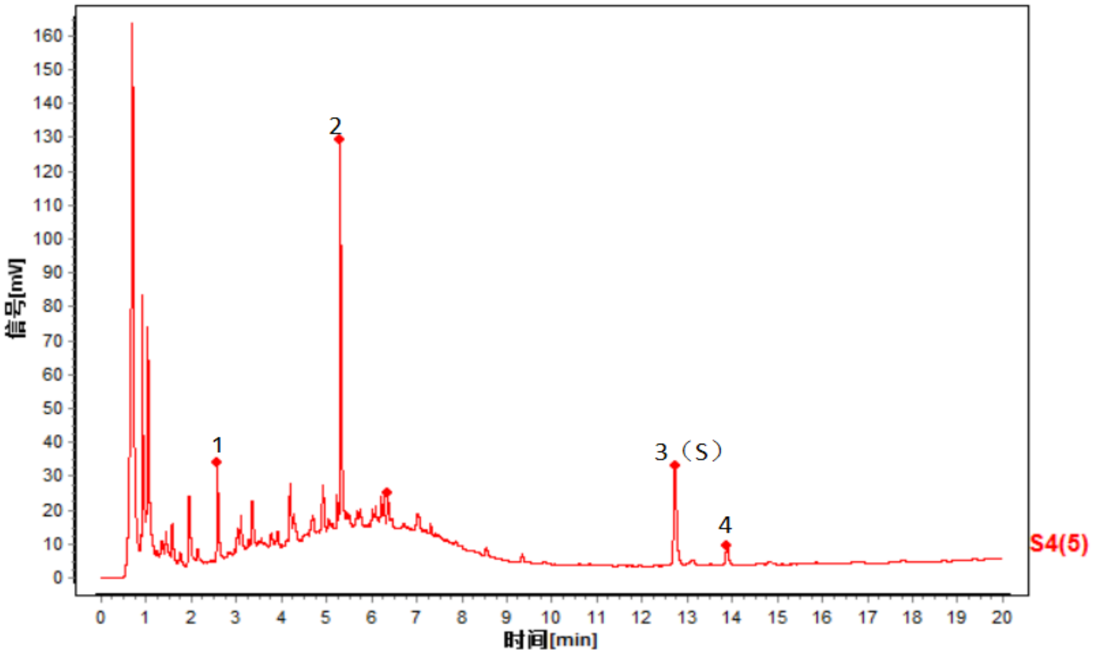

对照特征图谱

峰 3（S）：芝麻素

色谱柱：HSS T3（100mm×2.1mm，1.8μm）

【检查】 应符合颗粒剂（中国药典 通则 0104）项下有关的各项规定。

【浸出物】 照醇溶性浸出物测定法（中国药典 通则 2201）项下的热浸法测定，用乙醇作溶剂，不得少于 7.5%。

【含量测定】 照高效液相色谱法（中国药典 通则 0512）测定。

色谱条件与系统适应性实验 检测波长为201nm，其余同【特征图谱】项。

对照品溶液的制备 同【特征图谱】项下的对照品参照物溶液。

供试品溶液的制备 同【特征图谱】项。

测定法 分别精密吸取对照品溶液与供试品溶液各 1μl，注入液相色谱仪，测定，即得。

本品每1g含芝麻素（$C_{20}H_{18}O_6$）应为0.50~4.50mg。

【规格】 每1g配方颗粒相当于饮片12.5g

【贮藏】 密封。

江苏省药品监督管理局

中药配方颗粒标准

JS-YBZ-2022319

黄芩炭配方颗粒

Huangqintan Peifangkeli

【来源】 本品为唇形科植物黄芩 *Scutellaria baicalensis* Georgi 的干燥根经炮制并按标准汤剂的主要质量指标加工制成的配方颗粒。

【制法】 取黄芩炭饮片 4200g，加水煎煮，滤过，滤液浓缩成清膏（出膏率为 15～24%），加入辅料适量，干燥，再加入辅料适量，混匀，制粒，制成 1000g，即得。

【性状】 本品为黄色至黄棕色的颗粒；气微，味苦。

【鉴别】 取本品 1g，加乙酸乙酯-甲醇（3:1）的混合溶液 30ml，加热回流 30 分钟，放冷，滤过，滤液蒸干，残渣加甲醇 5ml 使溶解，取上清液作为供试品溶液。另取黄芩素对照品、汉黄芩素对照品，加甲醇分别制成每 1ml 各含 0.5mg 的溶液，作为对照品溶液。照薄层色谱法(中国药典 通则 0502)试验，吸取上述供试品溶液 2μl 及上述两种对照品溶液各 1μl，分别点于同一聚酰胺薄膜上，以甲苯-乙酸乙酯-甲醇-甲酸（10：3：1：2）为展开剂，预平衡 30 分钟，展开，取出，晾干，置紫外光灯（365nm）下检视。供试品色谱中，在与对照品色谱相应的位置上，显相同颜色的荧光斑点。

【特征图谱】 照高效液相色谱法（中国药典 通则 0512）测定。

色谱条件与系统适用性试验 以十八烷基硅烷键合硅胶为填充剂；以 0.2%磷酸水为流动相 A，以 0.2%磷酸甲醇为流动相 B，按下表梯度洗脱；流速为 0.8ml/min；柱温为 30℃；检测波长为 280nm。理论板数按黄芩苷峰计应不低于 2500。

时间（分钟）	流动相 A(%)	流动相 B(%)
0	53	47
15	53	47
20	40	60
35	40	60

参照物溶液的制备 取黄芩素、黄芩苷对照品适量，加甲醇分别制成每

1ml 各含 0.1mg 的对照品溶液，作为对照品参照物溶液。**【特征图谱】**

供试品溶液的制备　取本品适量，研细，取约 75mg，精密称定，置具塞锥形瓶中，精密加入 70%乙醇 50ml，称定重量，超声处理（功率 250W，频率 40kHz）30 分钟，放冷，再称定重量，用 70%乙醇补足减失的重量，摇匀，离心，取上清液，即得。

测定法　精密吸取参照物溶液与供试品溶液各 10μl，注入液相色谱仪，测定，即得。

供试品色谱图中应呈现与对照药材参照物色谱图中 10 个保留时间相对应的特征峰，峰 1、峰 8 应分别与相应对照品参照物峰的保留时间相对应。峰 2～5 与 S1(峰 1）的相对保留时间依次约为：1.39、1.58、1.69、1.77；峰 6～7、峰 9～10 与 S2 峰（峰 8）的相对保留时间依次约为：0.93、0.95、1.26、1.37。

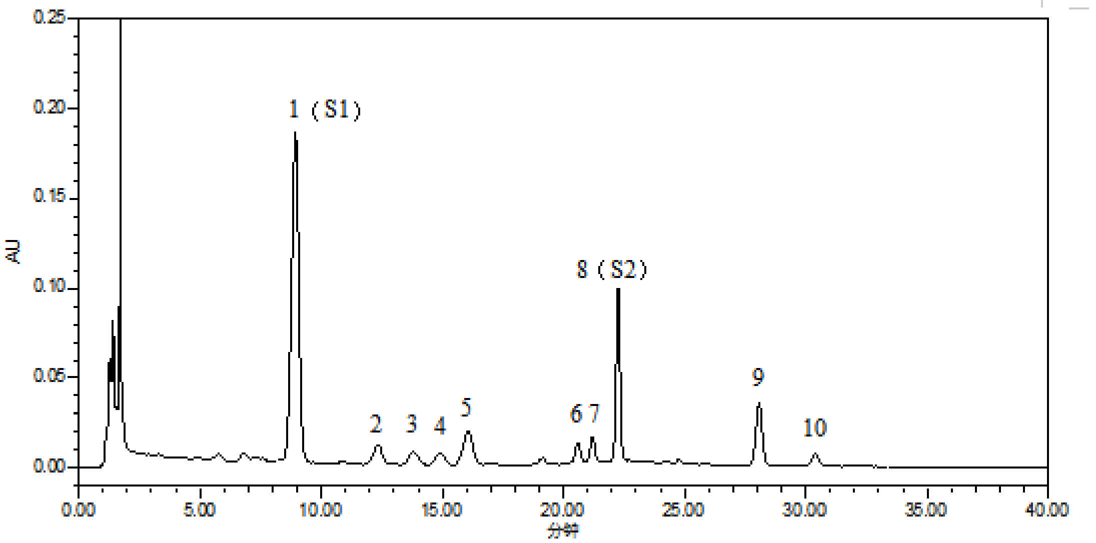

对照特征图谱

峰 1（S1）：黄芩苷　峰 8（S2）：黄芩素

色谱柱：ES Epic C18 （4.6mm×100mm，3μm）

【检查】 应符合颗粒剂（中国药典 通则 0104）项下有关的各项规定。

【浸出物】 照醇溶性浸出物测定法（中国药典 通则 2201）项下的热浸法测定，用乙醇作溶剂，不得少于 14.0%。

【含量测定】 照高效液相色谱法（中国药典 通则 0512）测定。

色谱条件与系统适用性试验　以十八烷基硅烷键合硅胶为填充剂（柱长为 100mm，内径为 4.6mm，粒径为 3μm），以 0.2%磷酸水为流动相 A，以 0.2%磷酸甲醇为流动相 B，按下表梯度洗脱；流速为 0.8ml/min；柱温为 30℃；检测波长为 280nm；理论板数按大黄素峰计应不低于 2500。

时间（分钟）	流动相 A(%)	流动相 B(%)
0	53	47
13	53	47
15	20	80
20	20	80

对照品溶液的制备　取黄芩苷对照品适量，精密称定，加甲醇制成每 1ml 含 60μg 的溶液，即得。

供试品溶液的制备 同【特征图谱】项。

测定法　精密吸取对照品溶液与供试品溶液各 10μl，注入液相色谱仪，测定，即得。

本品每 1g 含黄芩苷（$C_{21}H_{18}O_{11}$）应为 31~95mg。

【规格】　每 1g 配方颗粒相当于饮片 4.2g

【贮藏】　密封。

江苏省药品监督管理局

中药配方颗粒标准

JS-YBZ-2022320

僵蚕配方颗粒

Jiangcan Peifangkeli

【来源】 本品为蚕蛾科昆虫家蚕 *Bombyx mori* Linnaeus 4~5 龄的幼虫感染（或人工接种）白僵菌 *Beauveria bassiana*(Bals.)Vuillant 而致死的干燥体经炮制并按标准汤剂的主要质量指标加工制成的配方颗粒。

【制法】 取僵蚕饮片 3000g，加水煎煮，滤过，滤液浓缩成清膏（出膏率为 17~27%），加入辅料适量，干燥（或干燥，粉碎），再加入辅料适量，混匀，制粒，制成 1000g，即得。

【性状】 本品为浅灰黄色至黄棕色的颗粒；气腥，味淡、微咸。

【鉴别】 （1）取本品适量，研细，取0.2g，加50%乙醇5ml，超声处理10分钟，滤过，滤液作为供试品溶液。另取僵蚕对照药材1g，加水50ml，煎煮30分钟，滤过，滤液蒸干，残渣加50%乙醇5ml，同法制成对照药材溶液。照薄层色谱法（中国药典 通则0502）试验，吸取供试品溶液8μl、对照药材溶液3μl，分别点于同一硅胶G薄层板上，以正丁醇-冰醋酸-水（4∶1∶5）的上层溶液为展开剂，展开，取出，晾干，喷以0.5%茚三酮乙醇溶液，在105℃加热至斑点显色清晰。供试品色谱中，在与对照药材色谱相应的位置上，显相同颜色的斑点。

（2）取本品适量，研细，取0.5g，加1%碳酸氢铵溶液50ml，超声处理30分钟，用微孔滤膜滤过，取续滤液500μl，置进样瓶中，加胰蛋白酶溶液300μl（取序列分析用胰蛋白酶，加1%碳酸氢铵溶液制成每1ml中含1mg的溶液，临用时配制），摇匀，37℃恒温酶解12小时，作为供试品溶液。另取僵蚕多肽Ⅰ对照品、僵蚕多肽Ⅱ对照品，加1%碳酸氢铵溶液制成每1ml各含2μg的混合溶液，作为对照品溶液。照高效液相色谱法-质谱法（中国药典 通则0512、通则0431）试验，以十八烷基硅烷键合硅胶为填充剂（柱长为150mm，内径为2.1mm，粒径为1.7~1.9μm）；以乙腈为流动相A，以0.1%甲酸溶液为流动相B，按下表梯度洗脱，流速为0.3ml/min。采用质谱检测器，电喷雾正离子模式（ESI+），进行多反应监

测（MRM），选择质荷比（m/z）823（双电荷）→1070和m/z 823（双电荷）→1345作为僵蚕多肽Ⅰ的检测离子对；质荷比（m/z）637（三电荷）→825和m/z 637（三电荷）→926作为僵蚕多肽Ⅱ的检测离子对。取上述混合对照品溶液，进样5μl，按上述检测离子对测定的MRM色谱峰的信噪比均应大于3:1。

时间（分钟）	流动相 A（%）	流动相 B（%）
0	3	97
3	3	97
8	5	95
10	5	95
18	7	93
19	90	10
21	90	10

吸取供试品溶液5μl，注入高效液相色谱-质谱联用仪，测定。以质荷比（m/z）823（双电荷）→1070、m/z 823（双电荷）→1345和质荷比（m/z）637（三电荷）→825、m/z 637（三电荷）→926离子对提取的供试品离子流色谱中，应同时呈现与相应对照品色谱保留时间一致的色谱峰。

【特征图谱】 照高效液相色谱法（中国药典 通则 0512）测定。

色谱条件与系统适用性试验 以十八烷基硅烷键合硅胶为填充剂；以甲醇为流动相 A，以水为流动相 B，按下表梯度洗脱；流速为 0.35ml/min；柱温为 40 ℃；检测波长为 260nm。理论板数按腺苷峰计算应不低于 5000。

时间（分钟）	流动相 A(%)	流动相 B(%)
0	0	100
5	0	100
12	5	95
20	25	75

参照物溶液的制备 取僵蚕对照药材 1g，加水 50ml，超声处理（功率 250W，频率 40kHz）1 小时，置 100℃水浴加热 5 分钟，放冷，摇匀，滤过，取续滤液，作为对照药材参照物溶液。另取腺嘌呤、鸟苷、腺苷对照品适量，加 10%甲醇制成每 1ml 含腺嘌呤 10μg、鸟苷 25μg、腺苷 20μg 的混合溶液，作为对照品参照物溶液。

供试品溶液的制备 取本品适量，研细，取约 0.5g，精密称定，置具塞锥形瓶中，精密加入 10%乙醇 25ml，称定重量，加热回流 30 分钟，放冷，再称定重量，用 10%乙醇补足减失的重量，摇匀，滤过，取续滤液，即得。

测定法 分别精密吸取参照物溶液与供试品溶液各 1μl，注入液相色谱仪，测定，即得。

供试品色谱图中应呈现与对照药材参照物色谱图中 5 个保留时间相对应的特征峰，峰 1、峰 2、峰 4 应分别与相应对照品参照物峰的保留时间相对应。峰 3、5 与 S 峰（峰 4）的相对保留时间依次约为：0.92、1.20。

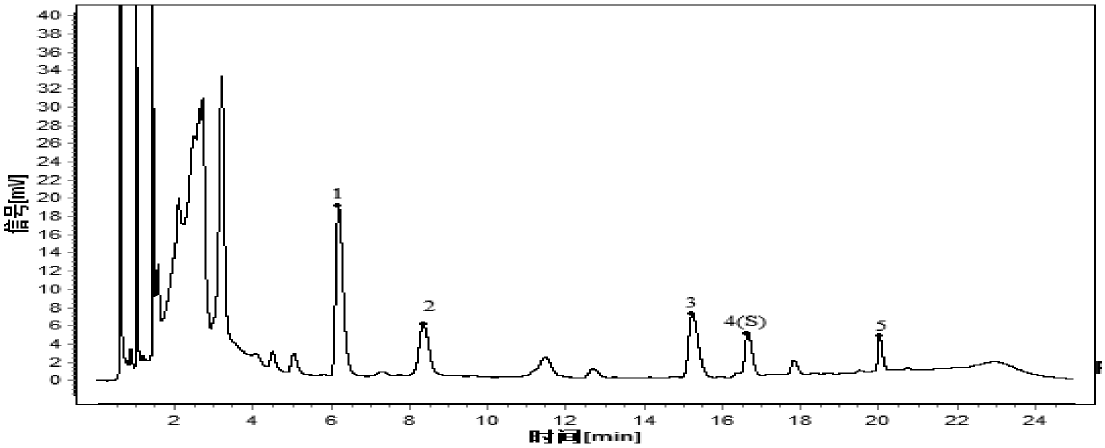

对照特征图谱

峰 1：腺嘌呤　峰 2：鸟苷　峰 4（S）：腺苷

色谱柱：Triart C18（100mm×2.1mm，1.9μm）

【检查】　黄曲霉毒素 照黄曲霉毒素测定法（中国药 通则2351）测定。

本品每 1kg 含黄曲霉毒素 B1 不得过 5μg；含黄曲霉毒素 G2、黄曲霉毒素 G1、黄曲霉毒素 B2 和黄曲霉毒素 B1 的总量不得过 10μg。

其他 应符合颗粒剂（中国药典 通则0104）项下有关的各项规定。

【浸出物】 取本品适量，研细，取约 2g，精密称定，精密加入乙醇 100ml，照醇溶性浸出物测定法（中国药典 通则 2201）项下的热浸法测定，不得少于 13.0%。

【含量测定】 照高效液相色谱法（中国药典 通则 0512）测定。

色谱条件与系统适用性试验　同【特征图谱】项。

对照品溶液的制备　取腺嘌呤、腺苷对照品适量，精密称定，加 10%乙醇溶液制成每 1ml 各含 10μg 的混合溶液，即得。

供试品溶液的制备　同【特征图谱】项。

测定法　精密吸取对照品溶液与供试品溶液各 1μl，注入液相色谱仪，测定，即得。

本品每 1g 含腺嘌呤（$C_5H_5N_5$）和腺苷（$C_{10}H_{13}N_5O_4$）的总量应为 0.4~2.4mg。

【规格】　每 1g 配方颗粒相当于饮片 3g

【贮藏】　密封。

江苏省药品监督管理局

中药配方颗粒标准

JS-YBZ-2022321

焦谷芽配方颗粒

Jiaoguya Peifangkeli

【来源】 本品为禾本科植物粟 *Setaria italica*(L.)Beauv.的成熟果实经发芽干燥的炮制加工品经炮制并按标准汤剂的主要质量指标加工制成的配方颗粒。

【制法】 取焦谷芽饮片 5900g，加水煎煮，滤过，滤液浓缩成清膏（出膏率为 10～17%），干燥（或干燥，粉碎），加入辅料适量，混匀，制粒，制成 1000g，即得。

【性状】 本品为土黄色至黄褐色颗粒；具焦香气，味微甘。

【鉴别】 取本品 1g，研细，加无水乙醇 10ml，超声处理 40 分钟，滤过，滤液加 50%氢氧化钾溶液 1.5ml，加热回流 15 分钟，置冰浴中冷却 5 分钟，用石油醚（30～60℃）振摇提取 3 次，每次 10ml，合并石油醚液，挥干，残渣加乙酸乙酯 1ml 使溶解，作为供试品溶液。另取谷芽对照药材 1g，同法制成对照药材溶液。照薄层色谱法（中国药典 通则 0502）试验，吸取上述两种溶液各 30μl，分别点于同一硅胶 G 薄层板上，以石油醚（30～60℃）–乙酸乙酯（4:1）为展开剂，展开，取出，晾干，喷以香草醛硫酸试液，在 105℃加热至斑点显色清晰，日光下检视，供试品色谱中，在与对照药材色谱相应的位置上，显相同颜色的斑点。

【特征图谱】 照高效液相色谱法（中国药典 通则 0512）测定。

色谱条件与系统适用性试验 以十八烷基硅烷键合硅胶为填充剂；以乙腈为流动相 A，0.5%乙酸为流动相 B，按下表梯度洗脱；流速为 0.8ml/min；柱温为 30℃；检测波长为 310nm。理论板数按 4-香豆酸峰计算应不低于 5000。

时间（分钟）	流动相 A(%)	流动相 B(%)
0	5	95
15	10	90
17	14	86
50	20	80
60	22	78

参照物溶液的制备 取 5-羟甲基糠醛、4-香豆酸对照品适量，精密称定，加甲醇制成每 1ml 含 5-羟甲基糠醛 10μg、4-香豆酸 20μg 的混合溶液，作为对照

品参照物溶液。

供试品溶液的制备 取本品适量，研细，约 1.0g，精密称定，加 50%甲醇 50ml，称定重量，超声（频率 250W，功率 40kHz）处理 30 分钟，取出，放冷，滤过，滤液浓缩至 5ml，转移至 10ml 容量瓶中，加 50%甲醇至刻度，摇匀，滤过，即得。

测定法 精密吸取参照物溶液与供试品溶液各 10μl，注入液相色谱仪，测定，即得。

供试品色谱图中应呈现与对照药材参照物色谱图中 5 个保留时间相对应的特征峰，峰 1、峰 4 应分别与相应对照品参照物峰的保留时间相对应。峰 2~3、5 与 S 峰（峰 4）的相对保留时间依次约为：0.32、0.91、0.95、1.12。

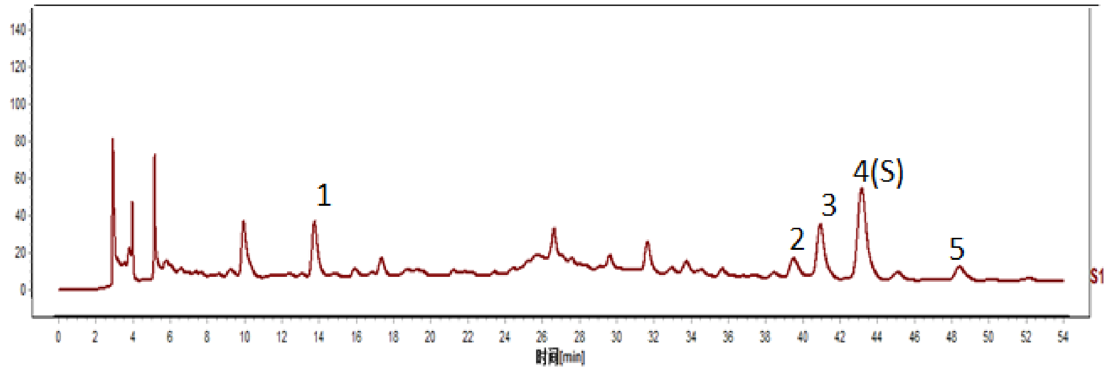

对照特征图谱

峰 1：5-羟甲基糠醛 峰 4（S）：4-香豆酸

色谱柱：Wondasil C18（250mm×4.6mm，5μm）

【检查】 应符合颗粒剂（中国药典 通则 0104）项下有关的各项规定。

【浸出物】 照醇溶性浸出物测定法（中国药典 通则 2201）项下的热浸法测定，不得少于 5.0%。

【含量测定】 照高效液相色谱法（中国药典 通则 0512）测定。

色谱条件与系统适用性试验 同【特征图谱】项。

对照品溶液的制备 同【特征图谱】项下的对照品参照物溶液。

供试品溶液的制备 同【特征图谱】项。

测定法 精密吸取参照物溶液与供试品溶液各 10μl，注入液相色谱仪，测定，即得。

本品每 1g 含 4-香豆酸（$C_9H_8O_3$）应为 0.05~0.20mg。

【规格】 每 1g 配方颗粒相当于饮片 5.9g

【贮藏】 密封。

江苏省药品监督管理局

中药配方颗粒标准

JS-YBZ-2022322

荆芥穗炭配方颗粒

Jingjiesuitan Peifangkeli

【来源】 本品为唇形科植物荆芥 *Schizonepeta tenuifolia* Briq.的干燥花穗经炮制并按标准汤剂的主要质量指标加工制成的配方颗粒。

【制法】 取荆芥穗炭饮片 6700g，加水煎煮，滤过，滤液浓缩成清膏（出膏率为 8～15%），干燥（或干燥，粉碎），加入辅料适量，混匀，制粒，制成 1000g，即得。

【性状】 本品为棕褐色至褐色颗粒；气芳香，味微涩而辛。

【鉴别】 取本品 1.0g，研细，加 75%乙醇 25ml，超声处理 30 分钟，滤过，用石油醚（60～90℃）萃取两次，每次 30ml，合并萃取液，蒸干，残渣加石油醚（60～90℃）1ml 使溶解，作为供试品溶液。另取荆芥穗对照药材 1.0g，加水 40ml，煮沸 30 分钟，滤过，滤液蒸干，残渣加 75%乙醇 25mL，同法制成对照药材溶液。按照薄层色谱法（中国药典 通则 0502）试验，吸取上述两种溶液各 10μl，分别点于同一硅胶 G 薄层板上，以甲苯-乙酸乙酯-甲酸（10:1:0.1）为展开剂，展开，取出，晾干，喷以 10%硫酸乙醇溶液，于 105℃加热至斑点显色清晰，置紫外光灯（365nm）下检视。供试品色谱中，在与对照药材色谱相应的位置上，显相同颜色的荧光斑点。

【特征图谱】 照高效液相色谱法（中国药典 通则 0512）测定。

色谱条件与系统适应性 以十八烷基硅烷键合硅胶为填充剂；以乙腈为流动相 A，以 0.1%磷酸水为流动相 B，按下表梯度洗脱；流速为 0.3ml/min；柱温为 40℃；检测波长为 348nm。理论板数按木犀草素峰计应不低于 10000。

时间（分钟）	流动相 A（%）	流动相 B（%）
0	7	93
2	18	82
15	25	75
19	32	68
21	7	93
30	7	93

参照物溶液的制备　取木犀草素对照品适量，精密称定，加 70%乙醇制成每 1ml 含 2μg 的溶液，摇匀，作为对照品参照物溶液。

供试品溶液的制备　取本品适量，研细，取约 0.5g，精密称定，置具塞锥形瓶中，精密加入 70%乙醇 25ml，称定重量，超声处理（功率 500W，频率 40kHz）30 分钟，取出，放冷，再称定重量，用 70%乙醇补足减失的重量，摇匀，滤过，取续滤液，即得。

测定法　精密吸取参照物溶液与供试品溶液各 2μl，注入液相色谱仪，测定，即得。

供试品色谱图中应呈现与对照药材参照物色谱图中 8 个保留时间相对应的特征峰，峰 1、4、6 应分别与相应对照品参照物峰的保留时间相对应。峰 1～5、7~8 与 S 峰（峰 6）的相对保留时间依次约为：0.46、0.48、0.55、0.66、0.96、1.08、1.27。

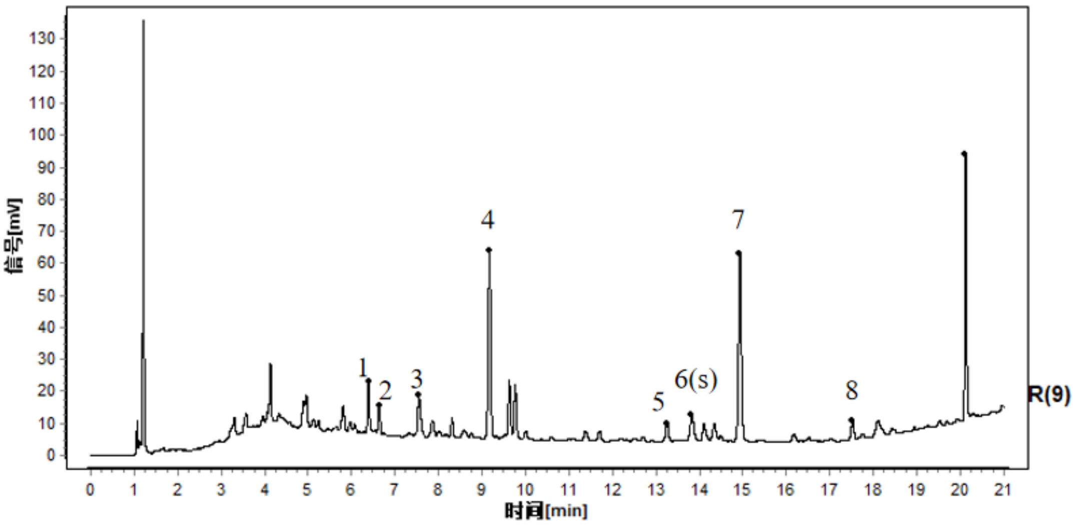

对照特征图谱

峰 1：木犀草苷；峰 4：迷迭香酸；峰 6（S）：木犀草素

色谱柱：　CORTECS UPLC T3 （150mm×2.1mm，1.6μm）

【检查】　应符合颗粒剂项下有关的各项规定（中国药典　通则 0104）。

【浸出物】　照醇溶性浸出物测定法（中国药典　通则 2201）项下的热浸法测定，用乙醇作溶剂，不得少于 11.0%。

【含量测定】　照高效液相色谱法（中国药典　通则 0512）测定。

色谱条件与系统适应性　同【特征图谱】项。

对照品溶液的制备　同【特征图谱】项下的对照品参照物溶液。

供试品溶液的制备 同【特征图谱】项。

测定法 精密吸取对照品溶液与供试品溶液各 2μl，注入液相色谱仪，测定，即得。

本品每 1g 含木犀草素（$C_{15}H_{10}O_6$）应为 0.06~0.11mg。

【规格】 每 1g 配方颗粒相当于饮片 6.7g

【贮藏】 密封。

江苏省药品监督管理局

中药配方颗粒标准

JS-YBZ-2022323

莲房配方颗粒

Lianfang Peifangkeli

【来源】 本品为睡莲科植物莲 *Nelumbo nucifera* Gaertn.的干燥花托经炮制并按标准汤剂的主要质量指标加工制成的配方颗粒。

【制法】 取莲房饮片 5000g，加水煎煮，滤过，滤液浓缩成清膏（出膏率为 10～16%），加入辅料适量，干燥（或干燥，粉碎），再加入辅料适量，混匀，制粒，制成 1000g，即得。

【性状】 本品为红棕色至深棕色颗粒；气微，味微苦、涩。

【鉴别】 取本品粉末 1g，加 95%乙醇 10ml，置 60℃水浴中静置 1 小时，冷却至室温，滤过，滤液蒸干，残渣加乙醇 1ml 使溶解，作为供试品溶液。另取金丝桃苷对照品，加乙醇制成每 1ml 含 0.5mg 的溶液，作为对照品溶液。照薄层色谱法（中国药典 通则 0502）试验，吸取上述两种溶液各 1~2μl，分别点于同一高效硅胶 GF_{254} 薄层板上，以乙酸乙酯-甲酸-水（8:1:1）为展开剂，展开，取出，晾干，喷以 10%硫酸乙醇溶液，在 105℃加热至斑点显色清晰。置紫外光灯（365nm）下检视。供试品色谱中，在与对照品色谱相应位置上，显相同颜色的荧光斑点。

【特征图谱】 照高效液相色谱法（中国药典 通则 0512）测定。

色谱条件与系统适用性试验 以十八烷基硅烷键合硅胶为填充剂；以乙腈为流动相 A，以 0.1%甲酸溶液为流动相 B，按下表梯度洗脱；柱温为 30℃；检测波长为 354nm。理论板数按金丝桃苷峰计应不低于 5000。这

时间（分钟）	流动相 A（%）	流动相 B（%）
0	11	89
10	14	86
20	16	84
28	16	84
35	21	79
45	22	78
47	75	25
50	90	10
52	11	89

参照物溶液的制备 取莲房对照药材 1g，按供试品溶液制备方法制成对照药材参照物溶液；另取金丝桃苷、异槲皮苷对照品适量，加 70%甲醇制成每 1ml 含金丝桃苷 20μg、异槲皮苷 10μg 的混合溶液，作为对照品参照物溶液。

供试品溶液的制备 取本品粉末约 1g，精密称定，置具塞锥形瓶中，精密加 70%甲醇 25ml，密塞，称定重量，超声处理（功率 500W，频率 40kHz）20 分钟，放冷，再称定重量，用 70%甲醇补足减失的重量，摇匀，滤过，取续滤液，即得。

测定法 精密吸取参照物溶液及供试品溶液各 10μl，注入液相色谱仪，测定，即得。

供试品色谱图中应呈现与对照药材参照物色谱图中 6 个保留时间相对应的特征峰，峰 4~5 应分别与相应对照品参照物峰的保留时间相对应。峰 1～3、峰 6 与 S 峰（峰 4）的相对保留时间依次约为：0.70、0.73、0.85、1.23。

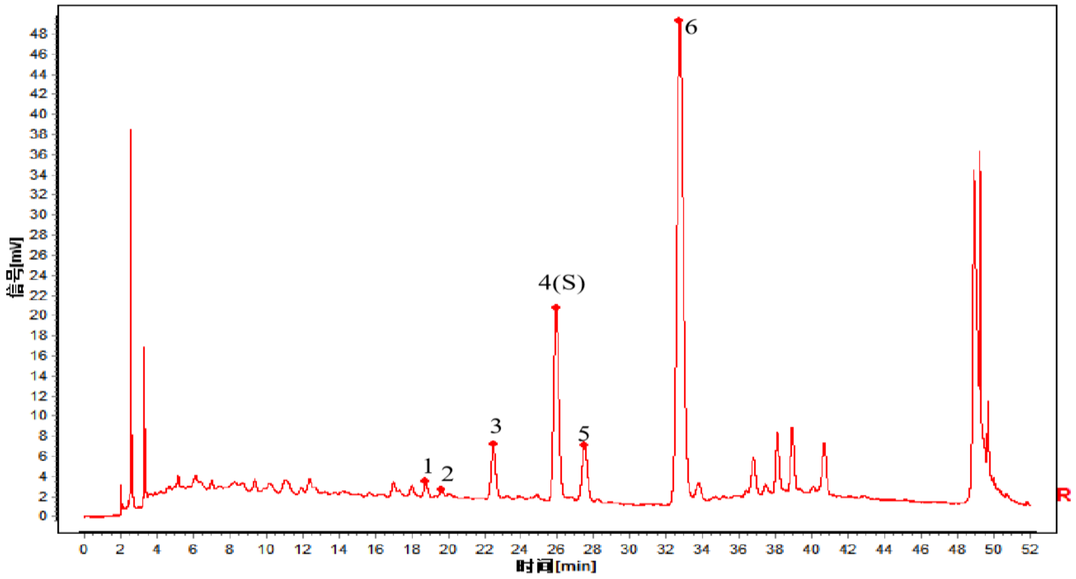

对照特征图谱

峰 4（S）：金丝桃苷　峰 5：异槲皮苷

色谱柱：Waters Symmetry® C18（250mm×4.6mm，5μm）

【**检查**】 应符合颗粒剂（中国药典 通则 0104）项下有关的各项规定。

【**浸出物**】 照醇溶性浸出物测定法项下的热浸法（中国药典 通则 2201）测定，用乙醇作溶剂，不得少于 7.0%。

【**含量测定**】 照高效液相色谱法（中国药典 通则 0512）测定。

色谱条件与系统适用性试验 同【特征图谱】项。

对照品溶液的制备 同【特征图谱】项下的对照品参照物溶液。

供试品溶液的制备　同【特征图谱】项。

测定法　精密吸取对照品溶液与供试品溶液各 10μl，注入液相色谱仪，测定，即得。

本品每 1g 含金丝桃苷（$C_{21}H_{20}O_{12}$）应为 0.2~2.4mg。

【规格】　每 1g 配方颗粒相当于饮片 5g

【贮藏】　密封。

江苏省药品监督管理局

中药配方颗粒标准

JS-YBZ-2022324

蓼大青叶配方颗粒

Liaodaqingye Peifangkeli

【来源】本品为蓼科植物蓼蓝 *Polygonum tinctorium* Ait.的干燥叶经炮制并按标准汤剂的主要质量指标加工制成的配方颗粒。

【制法】取蓼大青叶饮片 6700g，加水煎煮，滤过，滤液浓缩成清膏（出膏率为 12～15%），干燥（或干燥，粉碎），加入辅料适量，混匀，制粒，制成 1000g，即得。

【性状】本品为黄色至棕黄色颗粒；气微、味微涩而稍苦。

【鉴别】取本品 5g，研细，置锥形瓶中，加 2%水合氯醛的三氯甲烷溶液约 25ml，超声处理（功率 250W，频率 33kHz）1.5 小时，取出，放冷，摇匀，滤过，取溶液 10ml，浓缩至约 1ml，作为供试品溶液。另取靛蓝对照品，加三氯甲烷制成每 1ml 含 1mg 的溶液，作为对照品溶液。照薄层色谱法（中国药典 通则 0502）试验，吸取上述两种溶液各 5~10μl，分别点于同一硅胶 G 薄层板上，以苯-三氯甲烷-丙酮（5:4:1）为展开剂，展开，取出，晾干。供试品色谱中，在与对照品色谱相应的位置上，显相同颜色的蓝色斑点。

【特征图谱】照高效液相色谱法（中国药典 通则 0512）测定。

色谱条件与系统适用性实验 以十八烷基硅烷键合硅胶为填充剂；以甲醇为流动相 A，以水为流动相 B，按下表梯度洗脱；柱温为 30℃；检测波长为 260nm。理论板数按鸟苷峰计应不低于 3000。

时间（分钟）	流动相 A（%）	流动相 B（%）
0	3	97
10	3	97
15	25	75
20	55	45
25	64	36
35	90	10

参照物溶液的制备 取鸟苷、尿苷、腺苷对照品适量，精密称定，加 10%甲醇分别制成每 1ml 含 20μg 的溶液，作为对照品参照物溶液。

供试品溶液的制备 取本品适量，研细，取约 0.1g，精密加入水 15ml，密塞，称定重量，超声处理（功率 250W，频率 40kHz）20 分钟，放冷，再称定重量，用水补足减失的重量，摇匀，滤过，取续滤液，即得。

测定法 精密吸取对照品溶液与供试品溶液各 10μl，注入液相色谱仪，测定，即得。

供试品色谱图中应呈现与对照药材参照物色谱图中9个保留时间相对应的特征峰，峰3、峰5、峰7应分别与相应对照品参照物峰的保留时间相对应。峰1~2、峰4、峰6、峰8~9与S峰（峰5）的相对保留时间依次约为：0.34、0.39、0.58、1.09、2.30、2.35。

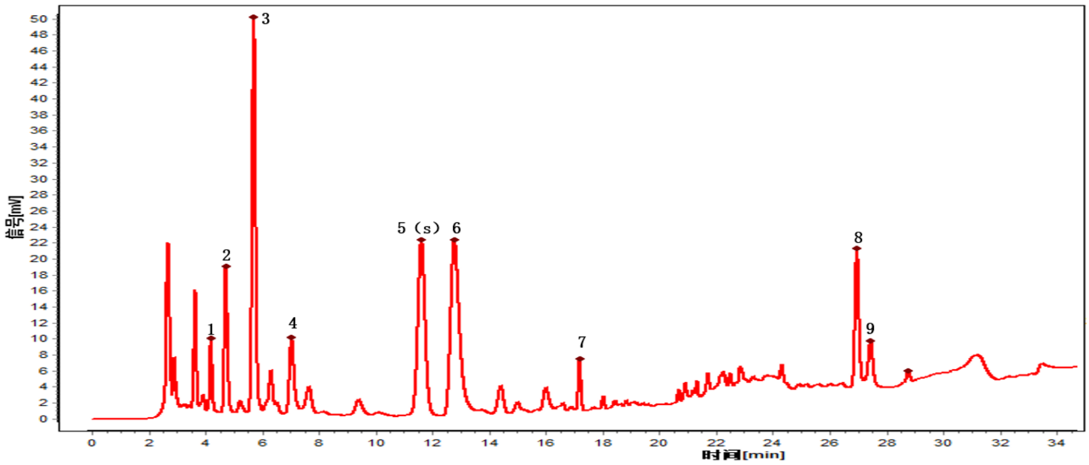

对照特征图谱

峰 3：尿苷　峰 5（S）：鸟苷　峰 7：腺苷

色谱柱：ZORBAX Bonus-RP（250mm×4.6mm，5μm）

【检查】 应符合颗粒剂（中国药典 通则 0104）项下有关的各项规定。

【浸出物】 照醇溶性浸出物测定法（中国药典 通则 2201）项下的热浸法测定，用乙醇作溶剂，应不得少于 24.0%。

【含量测定】 照高效液相色谱法（中国药典 通则 0512）测定。

色谱条件与系统适用性实验 同【特征图谱】项。

对照品溶液的制备 同【特征图谱】项下的对照品参照物溶液。

供试品溶液的制备 同【特征图谱】项。

测定法 精密吸取对照品溶液与供试品溶液各 10μl，注入液相色谱仪，测定，即得。

本品每 1g 含鸟苷（$C_{10}H_{13}N_5O_5$）应为 1.0~3.0mg。

【规格】 每 1g 配方颗粒相当于饮片 6.7g

【贮藏】 密封。

江苏省药品监督管理局

中药配方颗粒标准

JS-YBZ-2022325

木鳖子仁配方颗粒

Mubieziren Peifangkeli

【来源】 本品为葫芦科植物木鳖 *Momordica cochinchinensis* (Lour.) Spreng. 的干燥成熟种子经炮制并按标准汤剂的主要质量指标加工制成的配方颗粒。

【制法】 取木鳖子仁 10000g，加水煎煮，滤过，滤液浓缩成清膏（出膏率为 2.0-5.0%），加入辅料适量，干燥（或干燥，粉碎），再加入辅料适量，混匀，制粒，制成 1000g，即得。

【性状】 本品为近白色至灰白色颗粒，有特殊的油腻气，味苦。

【鉴别】 取本品 3g，研细，加乙酸乙酯 20ml，超声处理 30 分钟，滤过，滤液蒸干，残渣加乙酸乙酯 1ml 使溶解，作为供试品溶液。取木鳖子对照药材 0.5g，同法制成对照药材溶液。照薄层色谱法（中国药典 通则 0502）试验，吸取供试品溶液 10-15µl、对照药材溶液 1µl，分别点于同一硅胶 G 薄层板上，以环己烷-乙酸乙酯-丙酮-甲酸(6：0.5：1：0.1)为展开剂，展开，取出，晾干，置紫外光灯（365nm）下检视。供试品色谱中，在与对照药材色谱相应位置上，显相同颜色的荧光斑点。

【特征图谱】 照高效液相色谱法（中国药典 通则 0512）测定。

色谱条件与系统适用性试验 以十八烷基硅烷键合硅胶为填充剂；以 0.1%甲酸为流动相 A，以乙腈为流动相 B，按下表梯度洗脱；流速为 0.4ml/min；柱温为 35℃；检测波长为 254nm。理论板数按对羟基苯甲酸峰计应不低于 3000。

时间（分钟）	流动相 A（%）	流动相 B（%）
0	95	5
5	95	5
15	90	10
16	90	10
17	95	5
20	95	5

参照物溶液的制备 取木鳖子对照药材 2.0g，置锥形瓶中，加水 25ml，超声处理 30 分钟，放冷，摇匀，滤过，取续滤液，作为对照药材参照物溶液。另取

对羟基苯甲酸对照品适量，加甲醇制成每 1ml 含 2μg 的溶液，作为对照品参照物溶液。

供试品溶液的制备 取本品适量，研细，取约 0.2g，精密称定，置具塞锥形瓶中，精密加水 25ml，密塞，称定重量，超声处理（功率 390W，频率 40kHz）30 分钟，放冷，再称定重量，用水补足减失的重量，摇匀，滤过，取续滤液，即得。

测定法 精密吸取参照物溶液与供试品溶液各 2μl，注入液相色谱仪，测定，即得。

供试品色谱图中应呈现与对照药材参照物色谱图中 3 个保留时间相对应的特征峰，峰 1 应与对照品参照物峰的保留时间相对应。峰 2~3 与 S 峰（峰 1）的相对保留时间依次约为：1.41、2.71。

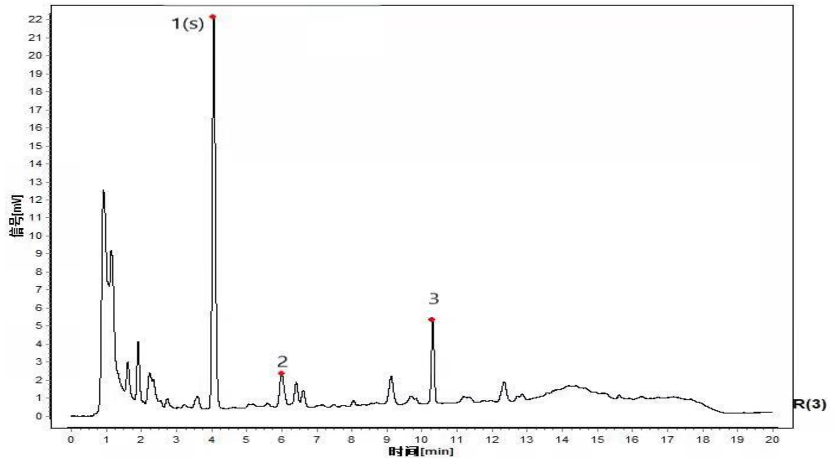

对照特征图谱

峰 1（S）：对羟基苯甲酸

参考色谱柱： BEH C18（100mm×2.1mm，1.7μm）

【检查】 应符合颗粒剂（中国药典 通则 0104）项下有关的各项规定。

【浸出物】 照醇溶性浸出物测定法（中国药典 通则 2201）项下的热浸法测定，用乙醇作溶剂，不得少于 5.0%。

【含量测定】 对羟基苯甲酸 照高效液相色谱法（中国药典 通则 0512）测定。

色谱条件与系统适用性试验 同【特征图谱】项。

对照品溶液的制备 同【特征图谱】项下的对照品参照物溶液。

供试品溶液的制备 同【特征图谱】项。

测定法　精密吸取对照品溶液与供试品溶液各 2μl，注入液相色谱仪，测定，即得。

本品每 1g 含对羟基苯甲酸（$C_7H_6O_3$）应为 0.20mg~0.70mg。

丝石竹皂苷元 3-O-β-D 葡萄糖醛酸甲酯　照高效液相色谱法（中国药典 通则 0512）测定。

色谱条件与系统适用性试验　以十八烷基硅烷键合硅胶为填充剂；以乙腈-0.4%磷酸溶液（70∶30）为流动相；检测波长为 203nm。理论板数按丝石竹皂苷元 3-O-β-D 葡萄糖醛酸甲酯峰计应不低于 6000。

对照品溶液的制备　取丝石竹皂苷元 3-O-β-D 葡萄糖醛酸甲酯对照品适量，精密称定，加甲醇制成每 1ml 含 0.1mg 的溶液，即得。

供试品溶液的制备　取本品适量，研细，取约 0.2g，精密称定，置锥形瓶中，加 60%甲醇 50ml，加热回流 1 小时，提取液蒸干。残渣加水 10ml 使溶解并转移至具塞试管中，加硫酸 0.6ml，摇匀，塞紧。置沸水浴中加热 2 小时，取出，放冷，离心，弃去滤液，残渣加甲醇 8ml 使溶解，转移至 10ml 量瓶中，加硫酸 1 滴使溶液 pH 值至 2，摇匀，50℃水浴中放置 1 小时，取出，放冷，加甲醇补至刻度，摇匀，滤过，取续滤液，即得。

测定法　精密吸取对照品溶液与供试品溶液各 10μl，注入液相色谱仪，测定，即得。

本品每 1g 含丝石竹皂苷元 3-O-β-D 葡萄糖醛酸甲酯（$C_{37}H_{56}O_{10}$）应为 2.6~6.6mg。

【规格】　每 1g 配方颗粒相当于饮片 10g

【贮藏】　密封。

江苏省药品监督管理局

中药配方颗粒标准

JS-YBZ-2022326

南刘寄奴配方颗粒

Nanliujinu Peifangkeli

【来源】 本品为菊科植物奇蒿 *Artemisia anomala* S.Moore 的干燥地上部分经炮制并按标准汤剂的主要质量指标加工制成的配方颗粒。

【制法】 取南刘寄奴饮片 5800g，加水煎煮，滤过，滤液浓缩成清膏（出膏率为 9~17%），干燥（或干燥，粉碎），加入辅料适量，混匀，制粒，制成 1000g，即得。

【性状】 本品为棕黄色至棕色颗粒；微香，味淡。

【鉴别】 取本品 0.5g，研细，加 30%甲醇 10ml，超声处理 30 分钟，滤过，作为供试品溶液。另取 3,5-O-二咖啡酰基奎宁酸、4,5-O-二咖啡酰奎宁酸对照品适量，精密称定，加 30%甲醇分别制成每 1ml 含 0.5mg 的溶液，作为对照品溶液。照薄层色谱法（中国药典 通则 0502）试验，吸取上述三种溶液各 1~3 μl，分别点于同一硅胶 G 薄层板上，以石油醚（60~90℃）-乙酸丁酯-甲酸-水（2:7:5:3）的上层溶液为展开剂，展开，取出，晾干，置紫外灯（365nm）下检视。供试品色谱中，在与对照品色谱相应的位置上，显相同颜色的荧光斑点。

【特征图谱】 照高效液相色谱法（中国药典 通则 0512）测定。

色谱条件与系统适用性试验 以十八烷基硅烷键合硅胶为填充剂；以乙腈为流动相 A，以 0.1%磷酸溶液为流动相 B，按下表梯度洗脱；流速为 0.3ml/min；柱温 30℃；检测波长为 280nm。理论板数按绿原酸峰计应不低于 5000。

时间（分钟）	流动相 A（%）	流动相 B（%）
0	8	92
20	12	88
55	23	77
70	35	65

参照物溶液的制备 取新绿原酸、隐绿原酸、绿原酸、异绿原酸 B、3,5-O-二咖啡酰基奎宁酸、4,5-O-二咖啡酰奎宁酸对照品适量，精密称定，加 50%甲醇制成每 1ml 分别含 20μg 的溶液，作为对照品参照物溶液。

供试品溶液的制备　取本品适量，研细，取约 0.4g，精密称定，置具塞锥形瓶中，精密加入 50%甲醇 25ml，密塞，称定重量，超声处理（功率 700W，频率 40kHz）20 分钟，放冷，再称定重量，用 50%甲醇补足减失的重量，摇匀，滤过，取续滤液，即得。

测定法　精密吸取参照物溶液与供试品溶液各 3μl，注入液相色谱仪，测定，即得。

供试品色谱图中应呈现与对照品参照物色谱图中 8 个保留时间相对应的特征峰，峰 1～3、峰 6～8 应分别与相应对照品参照物峰的保留时间相对应。峰 4～5 与 S 峰（峰 3）的相对保留时间依次约为：1.09、1.22。

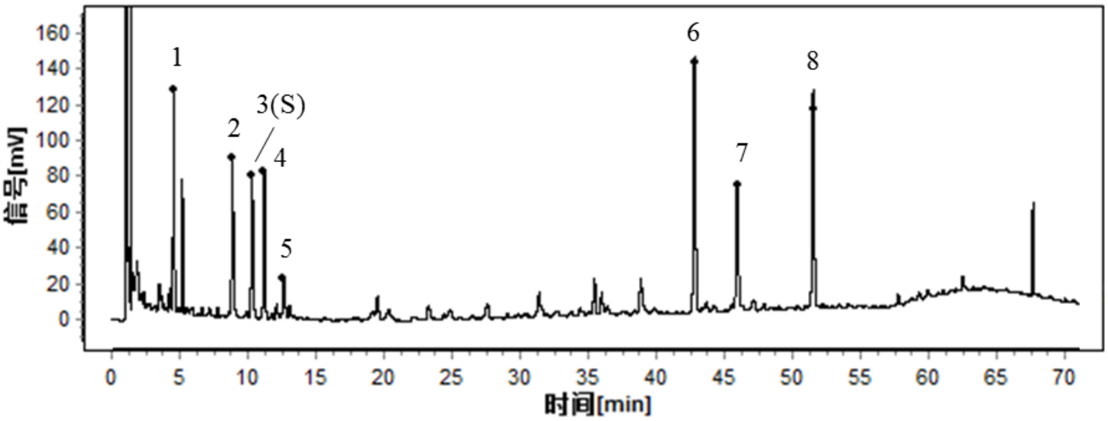

对照特征图谱

峰 1：新绿原酸　峰 2：隐绿原酸　峰 3（S）：绿原酸　峰 6：异绿原酸 B

峰 7：3,5-O-二咖啡酰基奎宁酸　峰 8：4,5-O-二咖啡酰奎宁酸

色谱柱：BEH Shield RP18（150mm×2.1mm，1.7μm）

【检查】　应符合颗粒剂（中国药典　通则 0104）项下有关的各项规定。

【浸出物】　照醇溶性浸出物测定法（中国药典　通则 2201）项下的热浸法测定，用乙醇作溶剂，不得少于 19.0%。

【含量测定】　照高效液相色谱法（中国药典　通则 0512）测定。

色谱条件与系统适用性试验　以十八烷基硅烷键合硅胶为填充剂（柱长 150mm，直径 2.1mm，粒径 1.7μm）；以乙腈为流动相 A，以 0.1%磷酸溶液为流动相 B，按下表梯度洗脱；流速为 0.3ml/min；柱温为 30℃；检测波长为 326nm。理论板数按 4,5-O-二咖啡酰奎宁酸峰计应不低于 5000。

时间（分钟）	流动相 A（%）	流动相 B（%）
0	18	82
20	20	80
30	80	20
35	18	82

对照品溶液的制备　取 4,5-O-二咖啡酰奎宁酸对照品适量，精密称定，加 70%甲醇制成每 1ml 含 40 μg 的溶液，即得（临用现配）。

供试品溶液的制备　取本品适量，研细，取约 0.2g，精密称定，置具塞锥形瓶中，精密加入 70%甲醇 25ml，密塞，称定重量，超声处理（功率 700W，频率 40kHz）30 分钟，放冷，再称定重量，用 70%甲醇补足减失的重量，摇匀，滤过，取续滤液，即得。

测定法　精密吸取对照品溶液与供试品溶液各 3μl，注入液相色谱仪，测定，即得。

本品每 1g 含 4,5-O-二咖啡酰奎宁酸（$C_{25}H_{24}O_{12}$）应为 3.0~6.3mg。

【规格】　每 1g 配方颗粒相当于饮片 5.9g

【贮藏】　密封。

江苏省药品监督管理局

中药配方颗粒标准

JS-YBZ-2022327

细辛（北细辛）配方颗粒

Xixin（Beixixin） Peifangkeli

【来源】 本品为马兜铃科植物北细辛 *Asarum heterotropoides* Fr.Schmidt var. *mandshuricum*（Maxim.）Kitag.的干燥根和根茎经炮制并按标准汤剂的主要质量指标加工制成的配方颗粒。

【制法】 取细辛（北细辛）饮片 3300g，加水煎煮，滤过，滤液浓缩成清膏（出膏率为 15～20%），加入辅料适量，干燥（或干燥，粉碎），再加入辅料适量，混匀，制粒，制成 1000g，即得。

【性状】 本品为黄色至黄棕色颗粒；气微，味辛辣，苦，麻舌。

【鉴别】 取本品适量，研细，取 0.5g，加甲醇 20ml，超声处理 45 分钟，滤过，滤液蒸干，残渣加甲醇 2ml 使溶解，作为供试品溶液。另取细辛（北细辛）对照药材 1.0g，加水 50ml，煮沸 30 分钟，滤过，滤液蒸干，残渣加甲醇 20ml，同法制成对照药材溶液。再取细辛脂素对照品，加甲醇制成每 1ml 含 1mg 的溶液，作为对照品溶液。照薄层色谱法（中国药典 通则 0502）试验，吸取供试品溶液、细辛对照药材溶液各 10μl、细辛脂素对照品溶液 5μl，分别点于同一硅胶 G 薄层板上，以石油醚（60～90℃）-乙酸乙酯（3：2）为展开剂，展开，取出，晾干，喷以 1%香草醛硫酸溶液，热风吹至斑点显色清晰。供试品色谱中，在与对照药材色谱和对照品色谱相应的位置上，显相同颜色的斑点。

【特征图谱】 照高效液相色谱法（中国药典 通则 0512）测定。

色谱条件与系统适用性试验 以十八烷基硅烷键合硅胶为填充剂（柱长为 10cm，直径为 2.1mm，粒径为 1.7μm），以乙腈为流动相 A，0.2%磷酸溶液为流动相 B，按下表梯度洗脱；流速为 0.4ml/min；柱温 40℃；检测波长为 287nm。理论板数按细辛脂素峰计应不低于 10000。

时间（分钟）	A（%）	B（%）
0	5	95
6	11	89
10	15	85
16	32	68
27	55	45

参照物溶液的制备 取细辛（北细辛）对照药材1.0g，加40ml水，煎煮30分钟，过滤，减压蒸干，精密加入70%甲醇30ml，超声处理（功率250W，频率40kHz）20分钟，放冷，摇匀，滤过，取续滤液，即得细辛对照药材参照物溶液。另取细辛脂素对照品适量，精密称定，加甲醇制成每1ml含细辛脂素30μg的溶液，即得细辛脂素对照品参照物溶液。

供试品溶液的制备 取本品适量，研细，精密称定约0.5g，置具塞锥形瓶中，精密加入70%甲醇25ml，密塞，称定重量，超声处理（功率250W，频率40kHz）40分钟，取出，放冷，再称定重量，用70%甲醇补足减失的重量，摇匀，滤过，取续滤液，即得。

测定法 精密吸取参照物溶液与供试品溶液各1μl，注入液相色谱仪，测定，即得。

供试品色谱图中应呈现与对照药材参照物色谱图中 6 个保留时间相对应的特征峰，峰6应与对照品参照物峰的保留时间相对应。峰1~5与S峰（峰6）的相对保留时间依次约为：0.07、0.21、0.23、0.28、0.34。

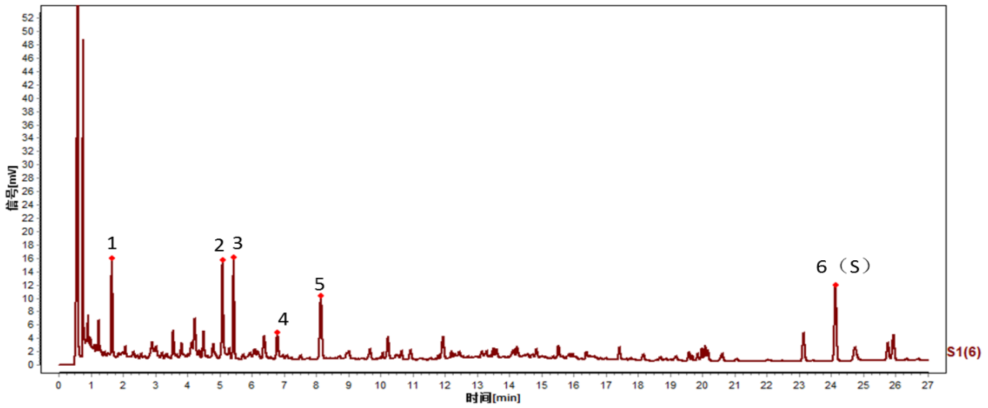

对照特征图谱

峰6（S）：细辛脂素

色谱柱：BEH C18（100mm×2.1mm，1.7μm）

【检查】 应符合颗粒剂（中国药典 通则0104）项下有关的各项规定。

马兜铃酸Ⅰ限量 照高效液相色谱法（中国药典 通则0512）测定。

色谱条件与系统适用性试验 以十八烷基硅烷键合硅胶为填充剂；以乙腈为

流动相 A，以 0.05%磷酸溶液为流动相 B，按下表梯度洗脱；检测波长 260nm。理论板数按马兜铃酸 I 峰计应不低于 5000。

时间（分钟）	流动相 A（%）	流动相 B（%）
0	30	70
10	34	66
18	35	65
20	45	55
30	45	55
31	53	47
35	53	47
40	100	0

对照品溶液的制备　取马兜铃酸 I 对照品适量，精密称定，加甲醇制成每 1ml 含 0.2μg 的溶液，作为对照品参照物溶液。

供试品溶液的制备　取本品适量，研细，精密称定约 0.5g，置具塞锥形瓶中，精密加入 70%甲醇 25ml，密塞，称定重量，超声处理（功率 250W，频率 40kHz）40 分钟，取出，放冷，再称定重量，用 70%甲醇补足减失的重量，摇匀，滤过，取续滤液，即得。

测定法　精密吸取对照品溶液与供试品溶液 10μl，注入液相色谱仪，测定，即得。

本品每 1g 含马兜铃酸 I（$C_{17}H_{11}NO_7$）不得过 0.01mg。

【浸出物】　照醇溶性浸出物测定法（中国药典 通则 2201）项下的热浸法测定，用乙醇作溶剂，不得少于 8.0%。

【含量测定】　照高效液相色谱法（中国药典 通则 0512）测定。

色谱条件与系统适用性试验　以十八烷基硅烷键合硅胶为填充剂（柱长为 10cm，柱内径为 2.1mm，粒径为 1.7μm）色谱柱；其余同【特征图谱】项。

对照品溶液的制备　取细辛脂素对照品适量，精密称定，加甲醇制成每 1ml 含细辛脂素 30μg 的溶液，即得。

供试品溶液的制备　同【特征图谱】项。

测定法　精密吸取对照品溶液与供试品溶液各 1μl，注入液相色谱仪，测定，即得。

本品每 1g 含细辛脂素（$C_{20}H_{18}O_6$）应为 0.3～1.7mg。

【规格】　每 1g 配方颗粒相当于饮片 3.3g

【贮藏】　密封。

江苏省药品监督管理局

中药配方颗粒标准

JS-YBZ-2022328

玉米须配方颗粒

Yumixu Peifangkeli

【来源】 本品为禾本科植物玉蜀黍 *Zea mays* L.的干燥花柱和柱头经炮制并按标准汤剂的主要质量指标加工制成的配方颗粒。

【制法】 取玉米须饮片 9100g，加水煎煮，滤过，滤液浓缩成清膏（出膏率为 6～11%），干燥（或干燥，粉碎），加入辅料适量，混匀，制粒，制成 1000g，即得。

【性状】 本品为棕黄色至棕褐色颗粒；气微，味淡。

【鉴别】 取本品 1g，研细，加甲醇 20ml，超声处理 30 分钟，滤过，滤液蒸干，残渣加甲醇 1ml 使溶解，作为供试品溶液。另取玉米须对照药材 2g，加水 100ml，煮沸 30 分钟，滤过，滤液蒸干，残渣加甲醇 2ml 使溶解，制成对照药材溶液。照薄层色谱法(中国药典 通则 0502)试验，吸取供试品溶液 15μl、对照药材溶液 10μl，分别点于同一硅胶 G 薄层板上，以正己烷-乙酸乙酯（7:3）为展开剂，展开，取出、晾干，喷以 10%的硫酸乙醇溶液，在 105℃下加热至斑点清晰，置紫外光灯(365nm)下检视。供试品色谱中，在与对照药材色谱相应的位置上，显相同颜色的荧光斑点。

【特征图谱】 照高效液相色谱法（中国药典 通则 0512）测定。

色谱条件与系统适应性试验 以十八烷基硅烷键合硅胶为填充剂；以甲醇为流动相 A，以 0.1%磷酸溶液为流动相 B，按下表梯度洗脱；柱温为 30℃；检测波长为 260nm。理论板数按原儿茶酸峰计应不低于 5000。

时间（分钟）	流动相 A（%）	流动相 B（%）
0	10	90
16	17	83
30	30	70
60	40	60

参照物溶液的制备 取玉米须对照药材约 3.0g，精密称定，置具塞锥形瓶中，加 15%甲醇 25ml，称定重量，超声处理（功率 500W，频率 40kHz）30 分钟，放冷，再称定重量，用 15%甲醇补足减失的重量，摇匀，滤过，取续滤液，作为对照药材参照物溶液。取原儿茶酸对照品适量，精密称定，加 50%甲醇制成每 1ml

含 8μg 的溶液，作为对照品参照物溶液。

供试品溶液的制备 取本品适量，研细，取约 2g，精密称定，置具塞锥形瓶中，精密加入 50%甲醇 25ml，密塞，称定重量，超声处理（功率 700W，频率 40kHz）60 分钟，放冷，再称定重量，用 50%甲醇补足减失的重量，摇匀，滤过，取续滤液，即得。

测定法 精密吸取参照溶液与供试品溶液各 10μl，注入液相色谱仪，测定，即得。

供试品色谱中应呈现与对照药材参照物色谱图中 4 个保留时间相对应的特征峰，峰 1 应与对照品参照物保留时间相对应。峰 2～4 与 S 峰（峰 1）的相对保留时间依次约为：1.61、2.01、3.07。

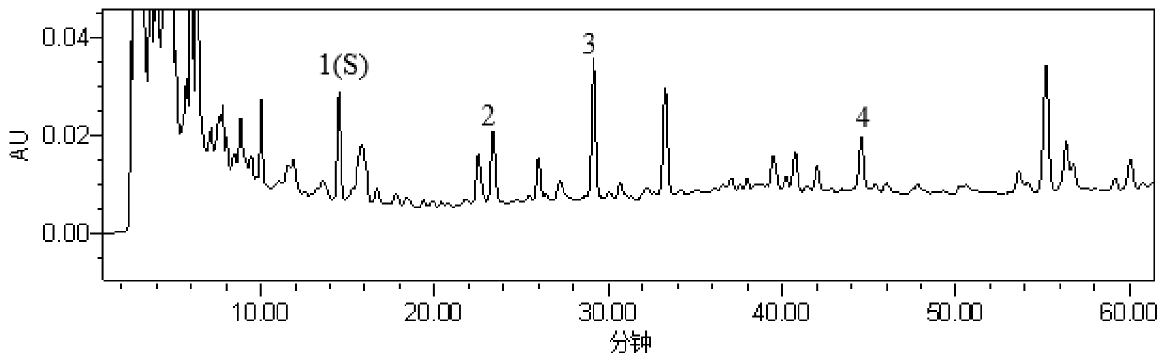

对照特征图谱

峰 1（S）：原儿茶酸

色谱柱：Ultimate LP-C18（250mm×4.6mm，5μm）

【检查】 应符合颗粒剂（中国药典 通则 0104）项下有关的各项规定。

【浸出物】 照醇溶性浸出物测定法（中国药典 通则 2201）项下的热浸法测定，用乙醇作溶剂，应不得少于 7.0%。

【含量测定】 照高效液相色谱法（中国药典 通则 0512）测定。

色谱条件与系统适应性试验 同【特征图谱】项。

对照品溶液的制备 同【特征图谱】项下的对照品参照物溶液。

供试品溶液的制备 同【特征图谱】项。

测定法 分别精密吸取对照品溶液及供试品溶液各 10μl，注入液相色谱仪，测定，即得。

本品每 1g 含原儿茶酸（$C_7H_6O_4$）应为 0.06~0.14mg。

【规格】 每 1g 配方颗粒相当于饮片 9.1g

【贮藏】 密封。

江苏省药品监督管理局

中药配方颗粒标准

JS-YBZ-2022329

苎麻根配方颗粒

Zhumagen Peifangkeli

【来源】 本品为荨麻科植物苎麻 *Boehmeria nivea* (L.) Gaud.的干燥根及根茎经炮制并按标准汤剂的主要质量指标加工制成的配方颗粒。

【制法】 取苎麻根饮片 5900g，加水煎煮，滤过，滤液浓缩成清膏（出膏率为 9～17%），干燥（或干燥，粉碎），加入辅料适量，混匀，制粒，制成 1000g，即得。

【性状】 本品为浅棕色至红棕色颗粒；无臭，味微苦。

【鉴别】 取本品粉末 7g，加无水乙醇 30ml，超声处理 20 分钟，滤过，滤液蒸干，残渣加 80%乙醇 2ml 使溶解，作为供试品溶液。取 β-谷甾醇对照品，加无水乙醇，制成每 1ml 含 1mg 的溶液，作为对照品溶液。照薄层色谱法（中国药典 通则 0502）试验，吸取上述供试品溶液 10~15μl、对照品溶液 1~2μl，分别点于同一硅胶 G 薄层板上，以环己烷-乙醚-乙酸乙酯（20：5.5：2.5）为展开剂，展开，取出，晾干，喷以 10%硫酸乙醇溶液，在 105℃加热数分钟，置紫外光灯（365nm）下检视。供试品色谱中，在与对照品色谱相应的位置上，显相同颜色的荧光斑点。

【特征图谱】 照高效液相色谱法（中国药典 通则 0512）测定。

色谱条件与系统适用性试验 以十八烷基硅烷键合硅胶为填充剂（柱长为 150mm，内径为 2.1mm，粒径为 1.6μm）；以乙腈为流动相 A，以 0.2%甲酸溶液为流动相 B，按下表梯度洗脱；流速为 0.3ml/min；柱温为 30℃；检测波长为 325nm。理论塔板数按隐绿原酸计应不低于 10000。

时间（分钟）	流动相 A（%）	流动相 B（%）
0	3	97
10	8	92
20	10	90
25	20	80
29	20	80
30	3	97

参照物溶液的制备　取苎麻根对照药材约 2.5g，加水 30ml，煮沸 30min，滤过，取续滤液，作为对照药材参照物溶液；另取绿原酸、隐绿原酸对照品适量，精密称定，加 50%甲醇分别制成每 1ml 含绿原酸 12μg、隐绿原酸 14μg 的溶液，作为对照品参照物溶液。

供试品溶液的制备　取本品适量，研细，取约 0.4g，精密称定，置锥形瓶中，加入水 20ml，密塞，超声处理（功率 300W，频率 40kHz）30 分钟，放冷，摇匀，滤过，取续滤液，即得。

测定法　精密吸取对照药材参照物溶液、对照品参照物溶液及供试品溶液各 3μl，注入液相色谱仪，测定，即得。

供试品色谱图中应呈现与对照药材参照物色谱图中 7 个保留时间相对应的特征峰，峰 2、4 应与对照品参照物峰的保留时间相对应。峰 1、3、5~7 与 S 峰（峰 4）的相对保留时间依次约为：0.55、0.91、1.33、1.60、1.67。

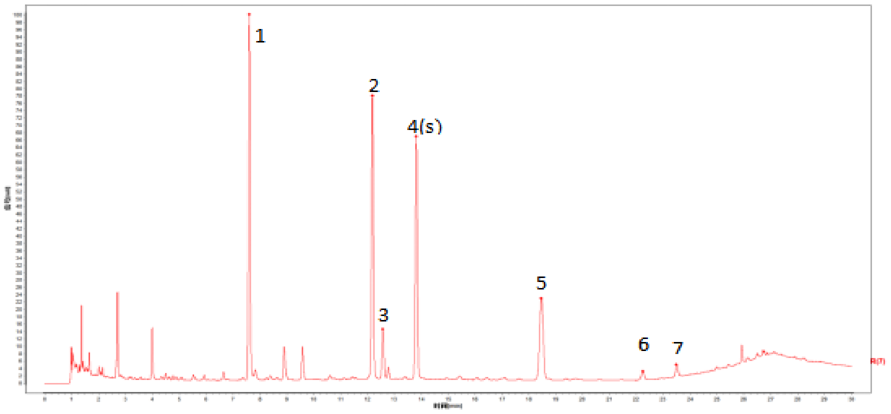

对照特征图谱
峰 2：绿原酸；峰 4（S）：隐绿原酸
色谱柱：CORTECS UPLC T3（150mm×2.1mm，1.6μm）

【检查】　应符合颗粒剂项下有关的各项规定（中国药典 通则 0104）。

【浸出物】　照醇溶性浸出物测定法（中国药典 通则 2201）项下的热浸法测定，用乙醇作溶剂，应不得少于 7.0%。

【含量测定】　照高效液相色谱法（中国药典 通则 0512）测定。

色谱条件与系统适用性试验　同【特征图谱】项。

对照品溶液的制备　同【特征图谱】项下的对照品参照物溶液。

供试品溶液的制备　同【特征图谱】项。

测定法　精密吸取对照品溶液及供试品溶液各 3μl，注入液相色谱仪，测定，

即得。

本品每1g含绿原酸（$C_{16}H_{18}O_9$）与隐绿原酸（$C_{16}H_{18}O_9$）的总量应为1.0~5.0mg。

【规格】 每1g配方颗粒相当于饮片5.9g

【贮藏】 密封。
