# Supplementary material for: Efficacy of Xiaoyao-san preparations in treating Hashimoto’s thyroiditis: a meta-analysis and systematic review
Source: Front Pharmacol. 2025 Jun 13;16:1528506. doi: 10.3389/fphar.2025.1528506 (PMC12202410; doi:10.3389/fphar.2025.1528506)
Supplement: Supplementary file 2 [file Supplementaryfile2.zip › Supplementary Files 2/Supplementary Files 2 formula granules section/Kunbu_Page 27_JS-YBZ-2024401.pdf]

附件

江苏省药品监督管理局

中药配方颗粒标准

JS-YBZ-2024393

萆薢（长托菝葜）配方颗粒

Bixie（Changtuobaqia） Peifangkeli

【来源】 本品为百合科植物长托菝葜 *Smilax ferox* Wall.ex Kunth 的干燥根茎经炮制并按标准汤剂的主要质量指标加工制成的配方颗粒。

【制法】 取萆薢（长托菝葜）饮片 6000g，加水煎煮，滤过，滤液浓缩成清膏（出膏率为 8-15%），加辅料适量，干燥（或干燥，粉碎），再加辅料适量，混匀，制粒，制成 1000g，即得。

【性状】 本品为棕黄色至棕褐色的颗粒；气微，味涩。

【鉴别】 取本品 1g，研细，加甲醇 10ml，超声处理 20 分钟，滤过，作为供试品溶液。另取白藜芦醇对照品，加甲醇制成每 1ml 含 0.2mg 的溶液，作为对照品溶液。照薄层色谱法（中国药典 通则 0502）试验，吸取供试品溶液与对照品溶液各 4μl，分别点于同一硅胶 GF_{254} 薄层板上，以甲苯-乙酸乙酯-甲酸（4：3：0.2）为展开剂，展开，取出，晾干，置紫外光灯（254 nm）下检视。供试品色谱中，在与对照品色谱相对应的位置上，显示相同颜色的斑点。

【特征图谱】 照高效液相色谱法（中国药典 通则 0512）测定。

色谱条件与系统适用性试验 以十八烷基硅烷键合硅胶为填充剂；以乙腈为流动相 A，以 0.1%磷酸溶液为流动相 B，按下表梯度洗脱；检测波长为 290nm；柱温为 30℃。理论板数按落新妇苷峰计应不低于 3000。

时间（分钟）	流动相 A（%）	流动相 B（%）
0	4	96
15	8	92
35	13	87
47	16	84
74	16	84
105	25	75

参照物溶液的制备 取萆薢（长托菝葜）对照药材 2g，加水 50ml，煎煮 40 分钟，放冷，滤过，滤液蒸干，残渣加 60%甲醇 10ml，超声处理 20 分钟，放冷，摇匀，滤过，取续滤液，作为对照药材参照物溶液。另取落新妇苷对照品适量，

精密称定，加 60%甲醇制成每 1ml 含 40μg 的溶液，作为对照品参照物溶液。

供试品溶液的制备　取本品适量，研细，取约 0.5g，精密称定，置具塞锥形瓶中，精密加入 60%甲醇 25ml，密塞，称定重量，超声处理（功率 600W，频率 40kHz）20 分钟，放冷，再称定重量，用 60%甲醇补足减失的重量，摇匀，滤过，取续滤液，即得。

测定法　分别精密吸取参照物溶液与供试品溶液各 10μl，注入液相色谱仪，测定，即得。

供试品色谱图中应呈现与对照药材参照物色谱图中 9 个保留时间相对应的特征峰。峰 1～4、峰 6~9 与 S 峰（峰 5）相对保留时间依次约为：0.31、0.48、0.51、0.96、1.08、1.14、1.21、1.37。

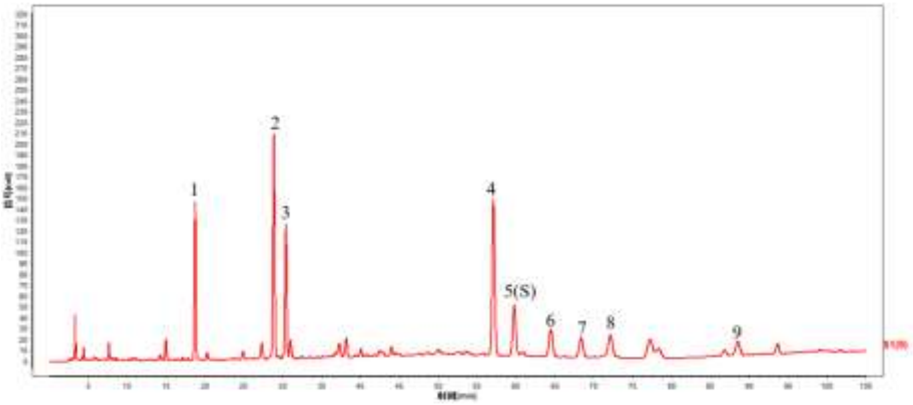

对照特征图谱

峰 1：新绿原酸 峰 2：绿原酸 峰 3：隐绿原酸 峰 4：新落新妇苷 峰 5（S）：落新妇苷 峰 7：新异落新妇苷 峰 8：异落新妇苷 峰 9：白藜芦醇

色谱柱：5 TC-C18（250mm×4.6mm，5μm）

【检查】 应符合颗粒剂（中国药典 通则 0104）项下有关的各项规定。

【浸出物】 照醇溶性浸出物测定法（中国药典 通则 2201）项下的热浸法测定，用乙醇作溶剂，不得少于 18.0%。

【含量测定】　照高效液相色谱法（中国药典 通则 0512）测定。

色谱条件与系统适用性试验　以十八烷基硅烷键合硅胶为填充剂；以乙腈为流动相 A，以 0.1%磷酸溶液为流动相 B，按下表梯度洗脱；检测波长为 290nm；柱温为 30℃。理论板数按落新妇苷峰计应不低于 3000。

时间（分钟）	流动相 A（%）	流动相 B（%）
0	20	80
10	16	84
40	16	84
42	20	80

对照品溶液的制备　同【特征图谱】项下的对照品参照物溶液。

供试品溶液的制备　同【特征图谱】项。

测定法 分别精密吸取对照品溶液与供试品溶液各 10μl，注入液相色谱仪，测定，即得。

本品每 1g 含落新妇苷（$C_{21}H_{22}O_{11}$）应为 0.85～4.40mg。

【规格】 每 1g 配方颗粒相当于饮片 6g

【贮藏】 密封。

江苏省药品监督管理局

中药配方颗粒标准

JS-YBZ-2024394

炒建曲配方颗粒

Chaojiangqu　Peifangkeli

【来源】 本品为辣子草、苍耳草等二十三味的加工品经炮制并按标准汤剂的主要质量指标加工制成的配方颗粒。

【制法】 取炒建曲饮片 2200g，加水煎煮，滤过，滤液浓缩成清膏（出膏率为 23~36%），加入辅料适量，干燥（或干燥，粉碎），再加入辅料适量，混匀，制粒，制成 1000g，即得。

【性状】 本品为灰黄色至灰褐色的颗粒；气微，味微苦。

【鉴别】 （1）取本品 3g，研细，加甲醇 100ml，加热回流 1 小时，滤过，滤液蒸干，残渣加水 20ml，加热使溶解，滤过，滤液加乙酸乙酯振摇提取两次，每次 20ml，合并乙酸乙酯液，蒸干，残渣加甲醇 1ml 使溶解，作供试品溶液。取橙皮苷对照品，加甲醇制成饱和溶液，作为对照品溶液。另取青蒿对照药材 1g，加水 50ml，煮沸 30 分钟，滤过，滤液蒸干，残渣加水 20ml 使溶解，用乙酸乙酯振摇提取 2 次，每次 20ml，合并乙酸乙酯液，蒸干，残渣加乙醇 1ml 使溶解，作为对照药材溶液。照薄层色谱法（中国药典 通则 0502）试验，吸取供试品溶液与对照品溶液各 5μl，对照药材溶液 1μl，分别点于同一用 0.5%氢氧化钠溶液制备的硅胶 G 薄层板上，以乙酸乙酯-甲醇-水（100∶17∶13）为展开剂，展开，展距约 4cm，取出，晾干；再以甲苯-乙酸乙酯-甲酸-水（20∶10∶1∶1）的上层溶液为展开剂，展开，展距约 8cm，取出，晾干，置紫外光灯（365nm）下检视。供试品色谱中，在与对照药材色谱相应的位置上，显相同颜色的荧光斑点。喷以 1%三氯化铝乙醇液，置紫外光（365nm）下检视。供试品色谱中，在与对照品色谱相应的位置上，显相同颜色的荧光斑点。

（2）取本品适量，研细，取 0.5g，加甲醇 30ml，超声处理 30 分钟，滤过，滤液蒸干，残渣加水 10ml 使溶解，用乙醚振摇提取两次，每次 20ml，弃去乙醚液，再用乙酸乙酯振摇提取两次，每次 20ml，合并乙酸乙酯液，蒸干，残渣加甲

醇 1ml 使溶解，作为供试品溶液。另取柚皮苷对照品，加甲醇制成每 1ml 含 0.5mg 的溶液，作为对照品溶液。照薄层色谱法（中国药典 通则 0502）试验，吸取供试品溶液 15μl，对照品溶液 1μl，分别点于同一聚酰胺薄膜上，以三氯甲烷-甲醇-丙酮-甲酸（20:3.5:1.5:0.1）为展开剂，展开，取出，晾干，喷以 5%三氯化铝乙醇溶液，在 105℃加热约 2 分钟，置紫外光灯（365nm）下检视。供试品色谱中，在与对照品色谱相应的位置上，显相同颜色的荧光斑点。

【特征图谱】 照高效液相色谱法（中国药典 通则 0512）测定。

色谱条件与系统适用性试验 以十八烷基硅烷键合硅胶为填充剂；以甲醇:乙腈（1:1）为流动相 A，以 0.1%甲酸溶液为流动相 B，按下表梯度洗脱；流速为 0.3ml/min；检测波长为 300nm；柱温为 30℃。理论板数按水合氧化前胡素峰计应不低于 5000。

时间（分钟）	流动相 A（%）	流动相 B（%）
0	16	84
12	18	82
13	28	72
18	30	70
40	30	70
41	42	58
60	56	44
65	90	10
70	90	10

参照物溶液的制备 取建曲对照药材 1g，加 70%乙醇 20ml，超声处理（功率 300W，频率 45kHz）30 分钟，放冷，摇匀，滤过，滤液蒸干，残渣加水 20ml 使溶解，，用二氯甲烷振摇提取 2 次，每次 20ml，合并二氯甲烷液，减压回收溶剂至干，残渣加甲醇使溶解，并分次转移至 5ml 量瓶中，加甲醇稀释至刻度，摇匀，滤过，取续滤液，作为对照药材参照物溶液。另取东莨菪内酯、甘草素、水合氧化前胡素、柚皮素、和厚朴酚、厚朴酚对照品适量，精密称定，加甲醇制成每 1ml 各含 0.1mg 的混合溶液，作为对照品参照物溶液。

供试品溶液的制备 取本品适量，研细，取 1g，加 70%乙醇 20ml，超声处理（功率 300W，频率 45kHz）30 分钟，放冷，摇匀，滤过，滤液蒸干，残渣加水 20ml 使溶解，用二氯甲烷振摇提取 2 次，每次 20ml，合并二氯甲烷液，蒸干，残渣加甲醇使溶解，并分次转移至 5ml 量瓶中，加甲醇稀释至刻度，摇匀，滤过，取续滤液，即得。

测定法 分别精密吸取参照物溶液和供试品溶液各 2μl，注入液相色谱仪，测定，即得。

供试品色谱图中应呈现与对照药材参照物色谱图中 8 个保留时间相对应的特征峰，其中峰 1、峰 3~4、峰 6~8 应分别与相应对照品参照物峰的保留时间相对应。峰 5 与 S 峰（峰 4）的相对保留时间约为：1.07。

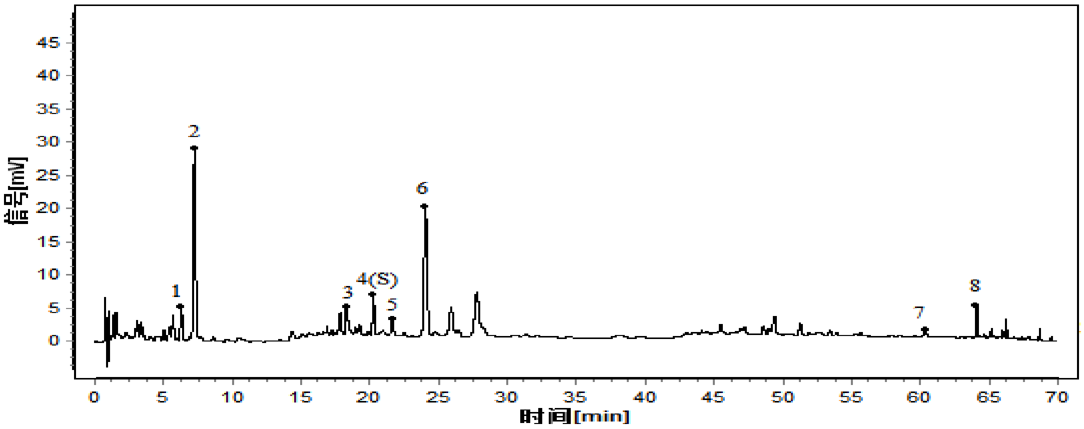

对照特征图谱

峰 1：东莨菪内酯　峰 3：甘草素　峰 4（S）：水合氧化前胡素

色谱柱：BEH C18（100mm×2.1mm, 1.7μm）

【检查】黄曲霉毒素 照黄曲霉毒素测定法（中国药典 通则 2351）测定。

本品每 1kg 含黄曲霉毒素 B1 不得过 5μg，黄曲霉毒素 G2、黄曲霉毒素 G1、黄曲霉毒素 B2 和黄曲霉毒素 B1 总量不得过 10μg。

溶化性 照颗粒剂溶化性检查方法（中国药典 通则 0104）检查，加热水 200ml，加热煮沸 5 分钟，立刻观察，应全部溶解或轻微浑浊，不得有焦屑或异物。

其他 应符合颗粒剂（中国药典 通则 0104）项下有关的各项规定。

【浸出物】 取本品研细，取约 2g，精密称定，精密加入乙醇 100ml，照醇溶性浸出物测定法（中国药典 通则 2201）项下的热浸法测定，不得少于 6.0%。

【含量测定】 照高效液相色谱法（中国药典 通则 0512）测定。

色谱条件与系统适用性试验 以十八烷基硅烷键合硅胶为填充剂（柱长为 100mm，内径为 2.1mm，粒径为 1.8μm~1.9μm）；以乙腈-水（17∶83）为流动相；流速为 0.40ml/min；检测波长为 283nm；柱温为 30℃。理论板数按橙皮苷峰计应不低于 8000。

对照品溶液的制备 取橙皮苷对照品适量，精密称定，加甲醇制成每 1ml 含

85μg 的溶液，即得。

供试品溶液的制备　取本品适量，研细，取约 0.5g，精密称定，置具塞锥形瓶中，加 70%甲醇 20ml，称定重量，超声处理（功率 300W，频率 45kHz）30 分钟，取出，放冷，再称定重量，用 70%甲醇补足减失重量，摇匀，滤过，取续滤液，即得。

测定法　精密吸取对照品溶液与供试品溶液各 1μl，注入液相色谱仪，测定，即得。

本品每 1g 含橙皮苷（$C_{28}H_{34}O_{15}$）应为 0.60~3.50mg。

【规格】 每 1g 配方颗粒相当于饮片 2.2g

【贮藏】 密封。

江苏省药品监督管理局

中药配方颗粒标准

JS-YBZ-2024395

醋甘遂配方颗粒

Cugansui　Peifangkeli

【来源】 本品为大戟科植物甘遂 *Euphorbia kansui* T.N. Liou ex T.P.Wang 的干燥块根经炮制并按标准汤剂的主要质量标准加工制成的配方颗粒。

【制法】 取醋甘遂饮片 1000g，加水煎煮，滤过，滤液浓缩成清膏（出膏率为 9~18%），加辅料适量，干燥（或干燥，粉碎），再加辅料适量，混匀，制粒，制成 1000g，即得。

【性状】 本品为黄白色至棕褐色颗粒；气微，味微甘而微辣。

【鉴别】 取本品 1g，研细，加乙醇 30ml，超声处理 1 小时，滤过，滤液挥干，残渣加甲醇 3ml 使溶解，作为供试品溶液。另取甘遂对照药材 2g，加水 50ml，煎煮 30 分钟，离心（3000 转/分钟）3 分钟，取上清液蒸至近干，加乙醇 30ml，搅匀，超声处理 30 分钟，滤过，滤液低温蒸至近干，残渣加甲醇 3ml 使溶解，作为甘遂对照药材溶液。照薄层色谱法（中国药典 通则 0502）试验，吸取甘遂对照药材溶液 20μl、供试品溶液 25μl，分别点于同一硅胶 GF_{254} 薄层板上，以甲苯-丙酮（10:1.2）为展开剂，在氨蒸气饱和条件下展开，取出，晾干，喷以 10%硫酸乙醇溶液，在 105℃加热至斑点显色清晰，置紫外光灯（365nm）下检视。供试品色谱中，在与对照药材色谱相对应的位置上，显示相同颜色的荧光主斑点。

【特征图谱】 照高效液相色谱法（中国药典 通则 0512）测定。

色谱条件与系统适用性试验 以十八烷基硅烷键合硅胶为填充剂；以甲醇为流动相 A，以水为流动相 B，按下表梯度洗脱；检测波长为 260nm；柱温为 20℃。理论板数按腺苷峰计应不低于 8000。

时间（分钟）	流动相 A（%）	流动相 B（%）
0	3	97
12	5	95
15	10	90
20	13	87
30	20	80
35	20	80

参照物溶液的制备 取甘遂对照药材 2g，加 20%甲醇溶液 20ml，密塞，超

声处理 30 分钟，放冷，滤过，取续滤液，作为对照药材参照物溶液。另取腺苷对照品适量，精密称定，加 20%甲醇制成每 1ml 含 10μg 的溶液，作为对照品参照物溶液。

供试品溶液的制备 取本品 0.5g，研细，加 20%甲醇溶液 10ml，密塞，超声处理 30 分钟，放冷，滤过，取续滤液，即得。

测定法 精密吸取参照物溶液与供试品溶液各 10μl，注入液相色谱仪，测定，即得。

供试品色谱中应呈现与对照药材参照物色谱中 6 个保留时间相对应的特征峰，峰 6 应与对照品参照物峰的保留时间相对应。峰 1~5 与 S 峰（峰 6）的相对保留时间依次约为：0.21、0.37、0.62、0.70、0.86。

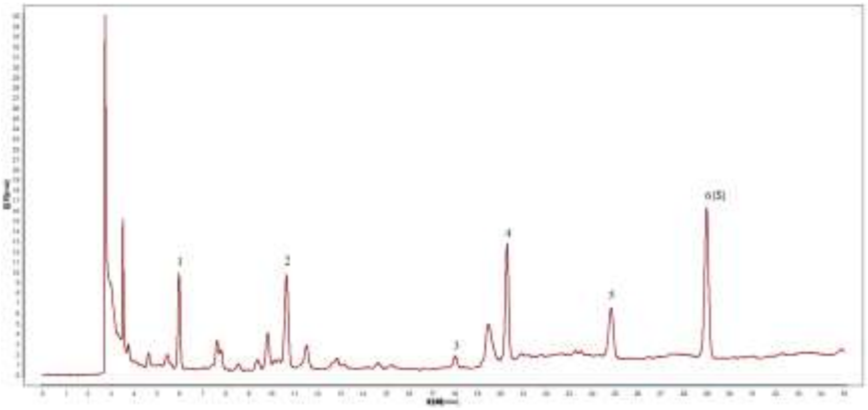

对照特征图谱

峰 2：尿苷　峰 4：鸟苷　峰 5：色氨酸　峰 6（S）：腺苷

色谱柱：5 TC-C18（250 mm×4.6mm，5μm）

【检查】 应符合颗粒剂（中国药典 通则 0104）项下有关的各项规定。

【浸出物】 照醇溶性浸出物测定法（中国药典 通则 2201）项下的热浸法测定，用乙醇作溶剂，不得少于 6.0%。

【含量测定】 照高效液相色谱法（中国药典 通则 0512）测定。

色谱条件与系统适用性试验 以十八烷基硅烷键合硅胶为填充剂；以甲醇-水（10∶90）为流动相；柱温为 20℃；检测波长为 260nm；理论板数按腺苷峰计应不低于 3000。

对照品溶液的制备 取腺苷对照品适量，精密称定，加 20%甲醇制成每 1ml 含 10μg 的溶液，即得。

供试品溶液的制备 取本品适量，研细，取约 0.5g，精密称定，置具塞锥形瓶中，精密加入 20%甲醇 10ml，密塞，称定重量，超声处理（功率 600W，频率

40kHz）30 分钟，放冷，再称定重量，用 20%甲醇补足减失的重量，摇匀，滤过，取续滤液，即得。

测定法　分别精密吸取对照品溶液与供试品溶液各 10μl，注入液相色谱仪，测定，即得。

本品每 1g 含腺苷（$C_{10}H_{13}N_5O_4$）应为 0.06~0.16mg。

【注意】 孕妇禁用；不宜与甘草同用。

【规格】 每 1g 配方颗粒相当于饮片 1g

【贮藏】 密封。

江苏省药品监督管理局

中药配方颗粒标准

JS-YBZ-2024396

醋芫花配方颗粒

Cuyuanhua Peifangkeli

【来源】 本品为瑞香科植物芫花 *Daphne genkwa* Sieb. et Zucc. 的干燥花蕾经炮制加工并按标准汤剂的主要质量指标加工制成的配方颗粒。

【制法】 取醋芫花饮片 3000g，加水煎煮，滤过，滤液浓缩成清膏（出膏率为 18~29%），干燥（或干燥，粉碎），加入辅料适量，混匀，制粒，制成 1000g，即得。

【性状】 本品为棕黄色至黄棕色的颗粒；气微，味淡。

【鉴别】 取本品 1g，研细，加甲醇 25ml，超声处理 10 分钟，滤过，滤液蒸干，残渣加乙醇 1ml 使溶解，作为供试品溶液。另取芫花对照药材 1g，加水 50ml，煎煮 30 分钟，滤过，滤液蒸干，残渣加甲醇 25ml，同法制成对照药材溶液。再取芫花素对照品，加甲醇制成每 1ml 含 2mg 的溶液，作为对照品溶液。照薄层色谱法（中国药典 通则 0502）试验，吸取上述三种溶液各 4μl，分别点于同一硅胶 G 薄层板上，以甲苯-乙酸乙酯-甲酸（8∶4∶0.2）为展开剂，展开，取出，晾干，喷以 10%硫酸乙醇溶液，在 105℃加热至斑点显色清晰，在紫外光灯（365nm）下检视。供试品色谱中，在与对照药材色谱和对照品色谱相应的位置上，显相同颜色的荧光斑点。

【特征图谱】 照高效液相色谱法（中国药典 通则 0512）测定。

色谱条件与系统适用性试验 以十八烷基硅烷键合硅胶为填充剂；以甲醇为流动相 A，以 0.1%甲酸溶液为流动相 B，按下表梯度洗脱；检测波长为 265nm；柱温为 30℃。理论板数按芫花素峰计应不低于 5000。

时间（分钟）	流动相 A（%）	流动相 B（%）
0	1	99
5	1	99
30	37	63
45	37	63
75	60	40
90	99	1
100	99	1

参照物溶液的制备　取芫花对照药材 1g，加水 50ml，加热回流 30 分钟，滤过，滤液蒸干，残渣加 70%甲醇 25ml，超声处理 45 分钟，放冷，滤过，取续滤液，作为对照药材参照物溶液。另取芫花素对照品适量，精密称定，加甲醇制成每 1ml 含 20μg 的溶液，作为对照品参照物溶液。

供试品溶液的制备　取本品适量，研细，取约 0.2g，置具塞锥形瓶中，加入 70%甲醇 25ml，超声处理（功率 250W，频率 40kHz）30 分钟，放冷，摇匀，滤过，取续滤液，即得。

测定法　精密吸取参照物溶液与供试品溶液各 10μl，注入液相色谱仪中，测定，即得。

供试品色谱图中应呈现与对照药材参照物色谱图中 10 个保留时间相对应的特征峰，峰 10 应与对照品参照物峰的保留时间相对应。峰 1～9 与 S 峰（峰 10）的相对保留时间依次约为：0.17、0.32、0.34、0.51、0.67、0.73、0.84、0.90、0.97。

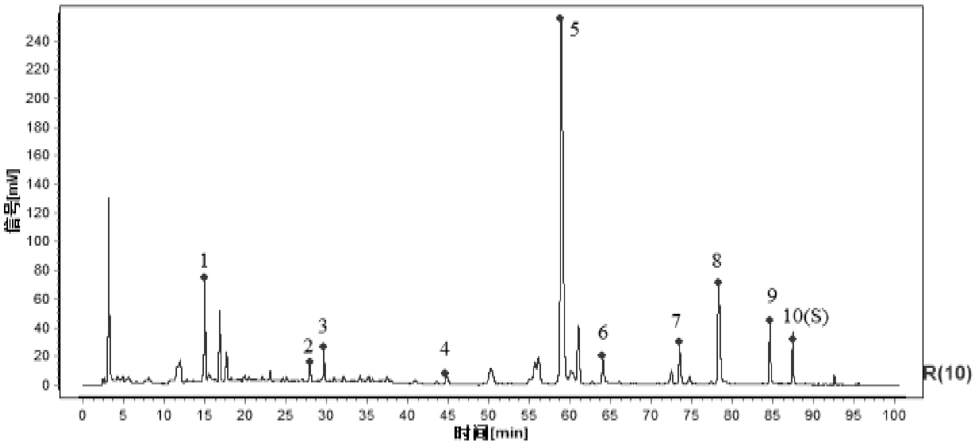

对照特征图谱

峰 10（S）：芫花素

色谱柱：pursuit XRs 5 C18（250mm×4.6mm，5μm）

【检查】　应符合颗粒剂（中国药典 通则 0104）项下有关的各项规定。

【浸出物】　取本品适量，研细，取约 2g，精密称定，置具塞锥形瓶中，精密加入乙醇 100ml，照醇溶性浸出物测定法（中国药典 通则 2201）项下的热浸法测定，不得少于 14.0%。

【含量测定】　照高效液相色谱法（中国药典 通则 0512）测定。

色谱条件与系统适用性试验　以十八烷基硅烷键合硅胶为填充剂；以甲醇-水-醋酸（65:35:0.8）为流动相；检测波长为 338nm。理论板数按芫花素峰计应不低于 3000。

对照品溶液的制备　同【特征图谱】项下的对照品参照物溶液。

供试品溶液的制备　取本品适量，研细，取约 0.5g，精密称定，置具塞锥形瓶中，精密加入甲醇 25ml，密塞，称定重量，超声处理（功率 300W，频率 40kHz）30 分钟，放冷，再称定重量，用甲醇补足减失重量，摇匀，滤过，取续滤液，即得。

测定法　精密吸取对照品溶液与供试品溶液各 10μl，注入液相色谱仪中，测定，即得。

本品每 1g 含芫花素（$C_{16}H_{12}O_5$）应为 0.45～1.2mg。

【规格】 每 1g 配方颗粒相当于饮片 3g

【贮藏】 密封。

江苏省药品监督管理局

中药配方颗粒标准

JS-YBZ-2024397

大豆黄卷配方颗粒

Dadouhuangjuan　Peifangkeli

【来源】 本品为豆科植物大豆 *Glycine max*（L.）Merr. 的成熟种子经发芽干燥的炮制加工品经炮制并按标准汤剂的主要质量指标加工制成的配方颗粒。

【制法】 取大豆黄卷饮片 4000g，加水煎煮，滤过，滤液浓缩成清膏（出膏率为 14~25%），加入辅料适量，干燥（或干燥，粉碎），再加入辅料适量，混匀，制粒，制成 1000g，即得。

【性状】 本品为黄白色至棕黄色的颗粒；气微，味微甜。

【鉴别】 （1）取本品适量，研细，取 0.3g，加甲醇 10mL，超声处理 30 分钟，滤过，取滤液作为供试品溶液。取大豆黄卷对照药材 1g，加水 50ml，煮沸，保持微沸 30 分钟，放冷，离心（转速为 2000r/min）10 分钟，滤过，滤液蒸干，残渣加甲醇 2ml，作为对照药材溶液。再取亮氨酸对照品，加甲醇制成每 1ml 含 0.5mg 的溶液，作为对照品溶液。照薄层色谱法（中国药典 通则 0502）试验，吸取供试品溶液 10μl、对照药材溶液和对照品溶液各 5μl，分别点于同一硅胶 G 薄层板上，以正丁醇-冰醋酸-水（19：5：5）为展开剂，展开，取出，晾干，喷以茚三酮试液，在 105℃加热至斑点显色清晰。供试品色谱中，在与对照药材色谱和对照品色谱相应的位置上，显相同颜色的斑点。

（2）取染料木苷对照品，加甲醇制成每 1ml 含 0.5mg 的溶液，作为对照品溶液。照薄层色谱法(中国药典 通则 0502)试验，吸取上述对照品溶液与【鉴别】（1）项下对照药材溶液各 5μl、【鉴别】（1）项下供试品溶液 10μl，分别点于同一硅胶 G 薄层板上，以乙酸乙酯-甲醇-水(10：1.7：1.3)为展开剂，置展开缸中预饱和 30 分钟，展开，取出，晾干，喷以 2%三氯化铝乙醇溶液，在 105℃加热数分钟，置紫外光灯(365nm)下检视。供试品色谱中，在与对照药材色谱和对照品色谱相应的位置上，显相同颜色的荧光斑点。

【特征图谱】 照高效液相色谱法（中国药典 通则 0512）测定。

色谱条件与系统适用性试验 以十八烷基硅烷键合硅胶为填充剂；以甲醇为流动相 A，以 1%醋酸溶液为流动相 B，按下表梯度洗脱；流速为 1.0 ml/min；柱温 35℃；检测波长为 260nm。理论板数按染料木苷峰计应不低于 5000。

时间（分钟）	流动相 A（%）	流动相 B（%）
0	5	95
8	10	90
15	28	72
35	45	55
55	60	40

参照物溶液的制备 取大豆黄卷对照药材 1g，加水 50ml，煎煮 30 分钟，放冷，离心（转速为 2000r/min）10 分钟，取上清液，蒸干，残渣加 70%甲醇 10ml 使溶解，滤过，取续滤液，作为对照药材参照物溶液。另取大豆苷、染料木苷、大豆苷元、染料木素对照品适量，精密称定，加 70%甲醇制成每 1ml 各含 20μg 的混合溶液，作为对照品参照物溶液。

供试品溶液的制备 取本品适量，研细，取 0.1g，精密称定，置具塞锥形瓶中，精密加入 70%甲醇 25ml，密塞，超声处理（功率 600W，频率 40kHz）30 分钟，放冷，摇匀，滤过，取续滤液，即得。

测定法 精密吸取参照物溶液与供试品溶液各 10μl，注入液相色谱仪，测定，即得。

供试品色谱图中应呈现与对照药材参照物色谱图中 7 个保留时间相对应的特征峰，其中峰 3~4、峰 6~7 应分别与相应对照品参照物峰的保留时间相对应。峰 1~2、峰 5 与 S 峰（峰 4）的相对保留时间依次约为：0.26、0.51、1.26。

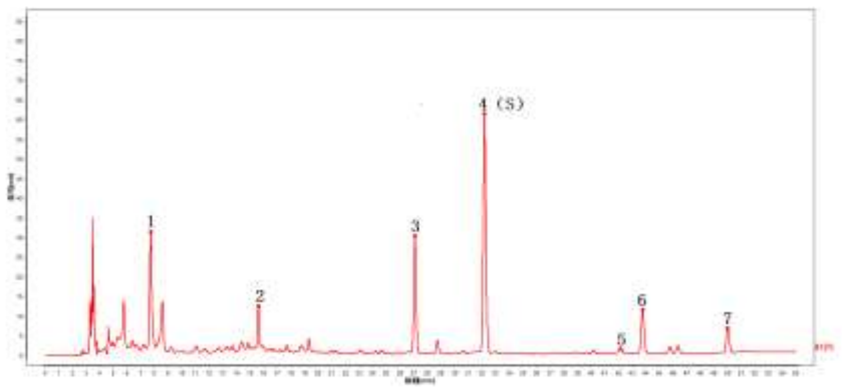

对照特征图谱

峰 3：大豆苷　峰 4（S）：染料木苷　峰 6：大豆苷元　峰 7：染料木素

色谱柱：　5 TC-C18(2)（250mm×4.6mm，5μm）

【检查】 应符合颗粒剂（中国药典 通则 0104）项下有关的各项规定。

【浸出物】 照醇溶性浸出物测定法（中国药典 通则 2201）项下的热浸法测定，用乙醇作溶剂，不得少于 20.0%。

【含量测定】 照高效液相色谱法（中国药典 通则 0512）测定。

色谱条件与系统适用性试验 以十八烷基硅烷键合硅胶为填充剂；以甲醇为流动相 A，以 1%醋酸溶液为流动相 B，按下表梯度洗脱；检测波长为 260nm。理论板数按大豆苷、染料木苷峰计应均不低于 5000。

时间（min）	流动相 A（%）	流动相 B（%）
0	28	72
25	28	72
33	45	55

对照品溶液的制备 取大豆苷、染料木苷对照品各适量，精密称定，加 70%甲醇制成每 1ml 各含 20μg 的混合溶液，即得。

供试品溶液的制备 取本品适量，研细，取约 0.1g，精密称定，置具塞锥形瓶中，精密加入 70%甲醇 25ml，称定重量，加热回流 2 小时，放冷，再称定重量，用 70%甲醇补足减失的重量，摇匀，滤过，取续滤液，即得。

测定法 精密吸取对照品溶液与供试品溶液各 10μl，注入液相色谱仪，测定，即得。

本品每 1g 含大豆苷（$C_{21}H_{20}O_9$）和染料木苷（$C_{21}H_{20}O_{10}$）的总量应为 3.7～6.8mg。

【规格】 每 1g 配方颗粒相当于饮片 4g

【贮藏】 密封。

江苏省药品监督管理局

中药配方颗粒标准

JS-YBZ-2024398

鸡蛋花配方颗粒

Jidanhua　Peifangkeli

【来源】 本品为夹竹桃科植物鸡蛋花 *Plumeria rubra* L. 'Acutifolia'的干燥花经炮制并按标准汤剂的主要质量指标加工制成的配方颗粒。

【制法】 取鸡蛋花饮片 3300g，加水煎煮，滤过，滤液浓缩成清膏（出膏率为 16~30%），加辅料适量，干燥（或干燥，粉碎），再加入辅料适量，混匀，制粒，制成 1000g，即得。

【性状】 本品为棕色至棕褐色的颗粒；气微，味微苦。

【鉴别】 取本品 0.4g，研细，加甲醇 20ml，超声处理 20 分钟，滤过，滤液蒸干，残渣加甲醇 1ml 使溶解，作为供试品溶液。另取鸡蛋花对照药材 0.5g，加水 25ml，煎煮 30 分钟，滤过，滤液蒸干，残渣加甲醇 20ml，同法制成对照药材溶液。照薄层色谱法（中国药典 通则 0502）试验，吸取上述两种溶液各 5μl，分别点于同一硅胶 G 薄层板上，以石油醚（60℃~90℃）-乙酸乙酯（4∶1）为展开剂，展开，取出，晾干，喷以 10%硫酸乙醇溶液，在 105℃加热至斑点显清晰，置紫外光灯（365nm）下检视。供试品色谱中，在与对照药材色谱相应的位置上，显相同颜色的荧光斑点。

【特征图谱】 照高效液相色谱法（中国药典 通则 0512）测定。

色谱条件与系统适用性试验 以十八烷基键硅烷合硅胶为填充剂；以乙腈为流动相 A，以 0.1%磷酸溶液为流动相 B，按下表梯度洗脱；流速为 0.3ml/min；检测波长为 260nm；柱温为 40℃。理论板数按芦丁峰计应不低于 5000。

时间（分钟）	流动相 A（%）	流动相 B（%）
0	5	95
6	8	92
10	9	91
15	14	86
26	18	82

参照物溶液的制备 取鸡蛋花对照药材 1g，加入 50%甲醇 25ml，加热回流

30 分钟，放冷，滤过，取续滤液，作为对照药材参照物溶液。另取绿原酸、芦丁对照品适量，精密称定，加 50%甲醇制成每 1ml 含绿原酸 50μg、芦丁 40μg 的溶液，作为混合对照品参照物溶液。再取原儿茶酸对照品适量，精密称定，加 50%甲醇制成每 1ml 含 30μg 的溶液，作为对照品参照物溶液。

供试品溶液的制备　取本品适量，研细，取约 0.5g，精密称定，置具塞锥形瓶中，精密加入 50%甲醇 25ml，密塞，称定重量，超声处理（功率 250W，频率 40kHZ）30 分钟，放冷，再称定重量，用 50%甲醇补足减失的重量，摇匀，滤过，取续滤液，即得。

测定法　分别精密吸取参照物溶液与供试品溶液各 1μl，注入液相色谱仪，测定，即得。

供试品色谱中应呈现与对照药材参照物色谱图中 12 个保留时间相对应的特征峰，峰 1、峰 3、峰 7 应分别与相应对照品参照物峰的保留时间相对应。峰 2、峰 4 与 S1 峰（峰 3）的相对保留时间依次约为：0.67、1.45；峰 5～6、峰 8~12 与 S2 峰（峰 7）的相对保留时间依次约为：0.89、0.97、1.02、1.04、1.17、1.19、1.34。

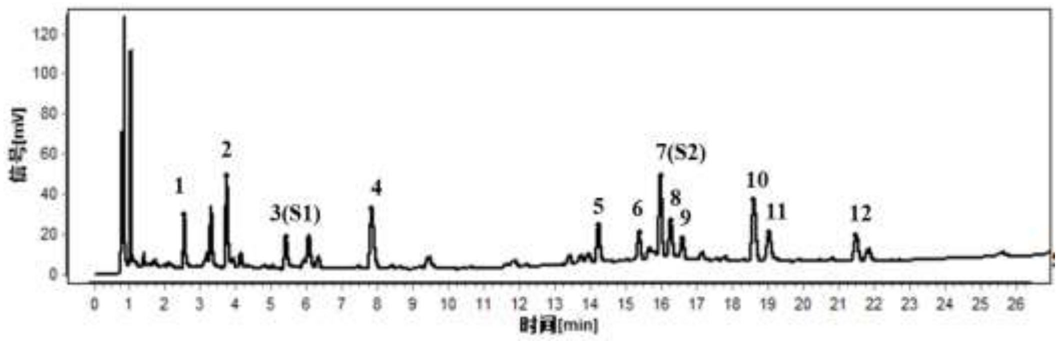

对照特征图谱

峰 1：原儿茶酸　峰 3（S1）：绿原酸　峰 7（S2）：芦丁

色谱柱：BEH C18（100mm×2.1mm，1.7μm）

【检查】 应符合颗粒剂（中国药典 通则 0104）项下有关的各项规定。

【浸出物】 照醇溶性浸出物测定法（中国药典 通则 2201）项下的热浸法测定，用乙醇作溶剂，不得少于 16.0%。

【含量测定】 照高效液相色谱法（中国药典 通则 0512）测定。

色谱条件与系统适用性试验　同【特征图谱】项。

对照品溶液的制备　同【特征图谱】项下的混合对照品参照物溶液。

供试品溶液的制备　同【特征图谱】项。

测定法　分别精密吸取对照品溶液与供试品溶液各 1μl，注入液相色谱仪，测定，即得。

本品每 1g 含绿原酸（$C_{16}H_{18}O_{9}$）应为 0.6~2.7mg；含芦丁（$C_{27}H_{30}O_{16}$）应为 0.3~2.1mg。

【规格】 每 1g 配方颗粒相当于饮片 3.3g

【贮藏】 密封。

江苏省药品监督管理局

中药配方颗粒标准

JS-YBZ-2024399

建曲配方颗粒

Jianqu　Peifangkeli

【来源】 本品为藿香、青蒿等药材细粉与面粉、生麸皮混合发酵而成的加工品经炮制并按标准汤剂的主要质量指标加工制成的配方颗粒。

【制法】 取建曲饮片 2500g，加水煎煮，滤过，滤液浓缩成清膏（出膏率为 21~39%），加入辅料适量，干燥（或干燥，粉碎），再加入辅料适量，混匀，制粒，制成 1000g，即得。

【性状】 本品为灰黄色至灰褐色的颗粒；气微，味微苦。

【鉴别】 （1）取本品 3g，研细，加甲醇 100ml，加热回流 1 小时，滤过，滤液蒸干，残渣加水 20ml，加热使溶解，滤过，滤液加乙酸乙酯振摇提取两次，每次 20ml，合并乙酸乙酯液，蒸干，残渣加甲醇 1ml 使溶解，作供试品溶液。取橙皮苷对照品，加甲醇制成饱和溶液，作为对照品溶液。另取青蒿对照药材 1g，加水 50ml，煮沸 30 分钟，滤过，滤液蒸干，残渣加水 20ml 使溶解，用乙酸乙酯振摇提取 2 次，每次 20ml，合并乙酸乙酯液，蒸干，残渣加乙醇 1ml 使溶解，作为对照药材溶液。照薄层色谱法（中国药典 通则 0502）试验，吸取供试品溶液与对照品溶液各 5μl，对照药材溶液 1μl，分别点于同一用 0.5%氢氧化钠溶液制备的硅胶 G 薄层板上，以乙酸乙酯-甲醇-水（100∶17∶13）为展开剂，展开，展距约 4cm，取出，晾干；再以甲苯-乙酸乙酯-甲酸-水（20∶10∶1∶1）的上层溶液为展开剂，展开，展距约 8cm，取出，晾干，置紫外光灯（365nm）下检视。供试品色谱中，在与对照药材色谱相应的位置上，显相同颜色的荧光斑点。喷以 1%三氯化铝乙醇液，置紫外光（365nm）下检视。供试品色谱中，在与对照品色谱相应的位置上，显相同颜色的荧光斑点。

（2）取本品适量，研细，取 0.5g，加甲醇 30ml，超声处理 30 分钟，滤过，滤液蒸干，残渣加水 10ml 使溶解，用乙醚振摇提取两次，每次 20ml，弃去乙醚液，再用乙酸乙酯振摇提取两次，每次 20ml，合并乙酸乙酯液，残渣加甲醇 1ml

使溶解，作为供试品溶液。另取柚皮苷对照品，加甲醇制成每 1ml 含 0.5mg 的溶液，作为对照品溶液。照薄层色谱法（中国药典 通则 0502）试验，吸取供试品溶液 10μl，对照品溶液 1μl，分别点于同一聚酰胺薄膜上，以三氯甲烷-甲醇-丙酮-甲酸（20：3.5：1.5：0.1）为展开剂，展开，取出，晾干，喷以 5%三氯化铝乙醇溶液，在 105℃加热约 2 分钟，置紫外灯（365nm）下检视。供试品色谱中，在与对照品色谱相应的位置上，显相同颜色的荧光斑点。

【特征图谱】 照高效液相色谱法（中国药典 通则 0512）测定。

色谱条件与系统适用性试验 以十八烷基硅烷键合硅胶为填充剂；以甲醇：乙腈（1:1）为流动相 A，以 0.1%甲酸溶液为流动相 B，按下表梯度洗脱；流速为 0.3ml/min；检测波长为 300nm；柱温为 30℃。理论板数按水合氧化前胡素峰计应不低于 5000。

时间(min)	流动相A(%)	流动相B(%)
0	16	84
12	18	82
13	28	72
18	30	70
40	30	70
41	42	58
60	56	44
65	90	10
70	90	10

参照物溶液的制备 取建曲对照药材 1g，加 70%乙醇 20ml，超声处理 30 分钟，过滤，蒸干，加水 20ml 使溶解，用二氯甲烷提取 2 次，每次 20ml，合并二氯甲烷液，减压回收溶剂至干，残渣加甲醇使溶解，转移至 5ml 量瓶中，加甲醇至刻度，摇匀，滤过，取续滤液，作为对照药材参照物溶液。另取东莨菪内酯、甘草素、水合氧化前胡素、柚皮素、和厚朴酚、厚朴酚对照品适量，精密称定，加甲醇制成每 1ml 各含 0.10mg 的混合溶液，作为对照品参照物溶液。

供试品溶液的制备 取本品适量，研细，取 1g，加 70%乙醇 20ml，超声处理 30 分钟，滤过，蒸干，残渣加水 20ml 使溶解，用二氯甲烷振摇提取 2 次，每次 20ml，合并二氯甲烷液，蒸干，残渣加甲醇 5ml 使溶解，滤过，取续滤液，即得。

测定法 分别精密吸取参照物溶液和供试品溶液各 2μl，注入液相色谱仪，测定，即得。

供试品色谱图中应呈现与对照药材参照物色谱图中 8 个保留时间相对应的

特征峰，其中峰 1、峰 3~4、峰 6~8 应分别与相应对照品参照物峰的保留时间相对应。峰 5 与 S 峰（峰 4）的相对保留时间约为：1.07。

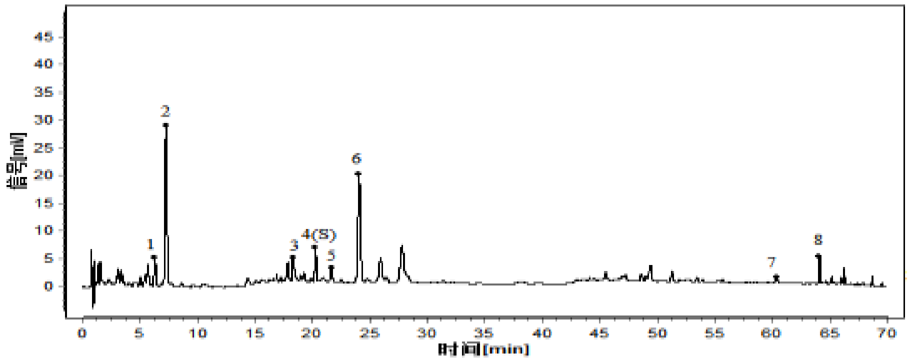

对照特征图谱

峰 1：东莨菪内酯　峰 3：甘草素　峰 4(S)：水合氧化前胡素

峰 6 柚皮素　峰 7：和厚朴酚　峰 8：厚朴酚

色谱柱：BEH C18（100mm×2.1mm, 1.7μm）

【检查】 黄曲霉毒素 照黄曲霉毒素测定法（中国药典 通则 2351）测定。

本品每 1kg 含黄曲霉毒素 B1 不得过 5μg，黄曲霉毒素 G2、黄曲霉毒素 G1、黄曲霉毒素 B2 和黄曲霉毒素 B1 总量不得过 10μg。

溶化性 照颗粒剂溶化性检查方法（中国药典 通则 0104）检查，加热水 200ml，加热煮沸 5 分钟，立刻观察，应全部溶解或轻微浑浊，不得有焦屑或异物。

其他 应符合颗粒剂（中国药典 通则 0104）项下有关的各项规定。

【浸出物】 取本品研细，取约 2g，精密称定，精密加入乙醇 100ml，照醇溶性浸出物测定法（中国药典 通则 2201）项下的热浸法测定，不得少于 6.0%。

【含量测定】 照高效液相色谱法（中国药典 通则 0512）测定。

色谱条件与系统适用性试验 以十八烷基硅烷键合硅胶为填充剂（柱长为 100mm，内径为 2.1mm，粒径为 1.8μm~1.9μm）；以乙腈-水（17∶83）为流动相；流速为 0.40ml/min；检测波长为 283nm；柱温为 30℃。理论板数按橙皮苷峰计应不低于 8000。

对照品溶液的制备 取橙皮苷对照品适量，精密称定，加甲醇制成每 1ml 含 85μg 的溶液，即得。

供试品溶液的制备 取本品适量，研细，取约 0.5g，精密称定，置具塞锥形瓶中，加 70%甲醇 20ml，称定重量，超声处理（功率 300W，频率 45kHz）30 分钟，取出，放冷，再称定重量，用 70%甲醇补足减失重量，摇匀，滤过，取续滤液，即得。

测定法 精密吸取对照品溶液与供试品溶液各 1μl，注入液相色谱仪，测定，即得。

本品每 1g 含橙皮苷（$C_{28}H_{34}O_{15}$）应为 0.70~5.50mg。

【规格】 每 1g 配方颗粒相当于饮片 2.5g

【贮藏】 密封。

江苏省药品监督管理局

中药配方颗粒标准

JS-YBZ-2024400

绞股蓝配方颗粒

Jiaogulan Peifangkeli

【来源】 本品为葫芦科植物绞股蓝 *Gynostemma pentaphyllum* (*Thunb.*) *Makino.* 的干燥全草经炮制并按标准汤剂的主要质量指标加工制成的配方颗粒。

【制法】 取绞股蓝饮片 5000g，加水煎煮，滤过，滤液浓缩成清膏（出膏率为 11~20%），加入辅料适量，干燥（或干燥，粉碎），再加入辅料适量，混匀，制粒，制成 1000g，即得。

【性状】 本品为浅黄棕色至棕褐色的颗粒；气微，味苦。

【鉴别】 取本品适量，研细，取 0.2g，加正丁醇 10ml，超声处理 30 分钟，滤过，滤液蒸干，残渣加 1ml 甲醇使溶解，作为供试品溶液。另取绞股蓝对照药材 2g，加水 25ml，煎煮 1 小时，滤过，滤液蒸干，残渣加正丁醇 10ml，超声处理 30 分钟，滤过，滤液蒸干，残渣加 1ml 甲醇使溶解，作为对照药材溶液。再取芦丁对照品适量，加甲醇制成每 1ml 含 0.1mg 的溶液，作为对照品溶液。照薄层色谱法（中国药典 通则 0502）试验，吸取上述三种溶液各 5μl，分别点于同一硅胶 G 薄层板上，以乙酸乙酯-甲酸-水（8∶1∶1）为展开剂，展开，取出，晾干，喷 1%三氯化铝溶液，在 105℃加热至斑点显色清晰，置紫外光灯（365 nm）下检视。供试品色谱中，在与对照药材色谱和对照品色谱相应的位置上，显相同颜色的荧光斑点。

【特征图谱】 照高效液相色谱法（中国药典 通则 0512）测定。

色谱条件与系统适用性试验 以十八烷基硅烷键合硅胶为填充剂；以乙腈为流动相 A，以 0.1%甲酸溶液为流动相 B，按下表梯度洗脱；检测波长为 254nm；柱温为 30℃。理论板数按芦丁峰计应不低于 3000。

时间（分钟）	流动相 A（%）	流动相 B（%）
0	10	90
15	20	80
50	70	30

参照物溶液的制备 取绞股蓝对照药材 1g，加水 25ml，加热回流 45 分钟，

滤过，滤液蒸干，残渣加 70%甲醇 20ml，超声处理 45 分钟，取出，放冷，滤过，取续滤液，作为对照药材参照物溶液。另取芦丁对照品适量，精密称定，加甲醇制成每 1ml 含 50μg 的溶液，作为对照品参照物溶液。

供试品溶液的制备 取本品适量，研细，取约 0.2g，精密称定，置具塞锥形瓶中，精密加入 70%甲醇 20ml，称定重量，超声处理（功率 250W，频率 40kHz）45 分钟，取出，放冷，再称定重量，用 70%甲醇补足减失的重量，摇匀，滤过，取续滤液，即得。

测定法 精密吸取参照物溶液与供试品溶液各 10μl，注入液相色谱仪，测定，即得。

供试品色谱中应呈现应与对照药材参照物色谱中 6 个保留时间相对应的特征峰，其中峰 3 应与对照品参照物峰保留时间相对应。峰 1~2、峰 4~6 与 S 峰（峰 3）的相对保留时间依次约为：0.42、0.63、1.36、1.49、2.15。

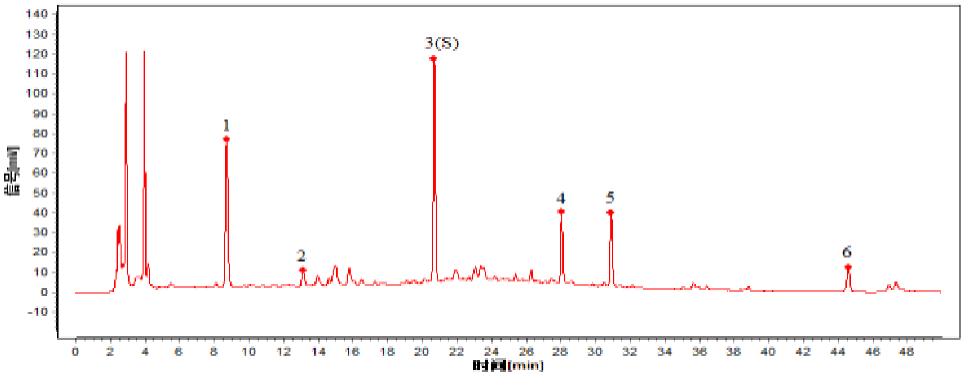

对照特征图谱

峰 3（S 峰）：芦丁　峰 4：商陆苷　峰 5：槲皮素　峰 6：商路素

色谱柱：Platisil 5μm ODS（250mm×4.6mm，5μm）

【检查】 **重金属及有害元素** 照铅、镉、砷、汞、铜测定法（中国药典 通则 2321）测定，铅不得过 5mg/kg；镉不得过 1mg/kg；砷不得过 2mg/kg；汞不得过 0.2mg/kg；铜不得过 20mg/kg。

其他 应符合颗粒剂（中国药典 通则 0104）项下有关的各项规定。

【浸出物】 取本品适量，研细，取 2g，精密称定，置具塞锥形瓶中，精密加入乙醇 100ml，照醇溶性浸出物测定法（中国药典 通则 2201）项下的热浸法测定，不得少于 24.0%。

【含量测定】 照高效液相色谱法（中国药典 通则 0512）测定。

色谱条件与系统适用性试验　以十八烷基硅烷键合硅胶为填充剂；以乙腈-0.1%甲酸溶液（15∶85）为流动相；检测波长为 254nm；柱温为 30℃。理论板数按芦丁峰计应不低于 3000。

对照品溶液的制备　同【特征图谱】项下的对照品参照物溶液。

供试品溶液的制备　同【特征图谱】项

测定法　精密吸取对照品溶液与供试品溶液各 10μl，注入液相色谱仪，测定，即得。

本品每 1g 含芦丁（$C_{27}H_{30}0_{16}$）应为 2.0~9.0mg。

【规格】　每 1g 配方颗粒相当于饮片 5 g

【贮藏】　密封。

江苏省药品监督管理局

中药配方颗粒标准

JS-YBZ-2024401

昆布（海带）配方颗粒

Kunbu（Haidai） Peifangkeli

【来源】本品为海带科植物海带 *Laminaria japonica* Aresch.的干燥叶状体的炮制加工品并按标准汤剂的主要质量指标加工制成的颗粒。

【制法】 取昆布饮片 3800g，加水煎煮，滤过，滤液浓缩成清膏（出膏率为 20～26%），干燥（或干燥、粉碎），加入辅料适量，混匀，制粒，制成 1000g，即得。

【性状】 本品为棕黄色至棕褐色颗粒；气腥，味咸。

【鉴别】 取本品 2g，研细，加水 25ml 使溶解，加入盐酸 5ml，加热回流 1 小时，加三氯甲烷振摇提取 2 次，每次 25ml，合并三氯甲烷液，蒸干，残渣加三氯甲烷 1ml 使溶解，作为供试品溶液。另取昆布对照药材粉末 5g，加水 100ml，回流提取 30 分钟，滤过，滤液浓缩至 25ml，后续操作同供试品自“加水 25ml 使溶解”起，同法制成对照药材溶液。照薄层色谱法（中国药典 通则 0502）试验，分别吸取上述两种溶液 5μl，点于同一硅胶 G 薄层板上，以环己烷-乙酸乙酯-冰乙酸（8:5:0.2）为展开剂，展开，取出，晾干，置紫外光灯（365nm）下检视。供试品色谱中，在相应的位置上，显三个相同颜色的荧光主斑点。

【特征图谱】 照高效液相色谱法（中国药典 通则 0512）测定。

色谱条件与系统适用性试验 以十八烷基硅烷键合硅胶为填充剂；以乙腈为流动相 A，0.05%磷酸溶液为流动相 B，按下表梯度洗脱；检测波长为 210nm。理论塔板数按亚油酸峰计算应不低于 5000。

时间（分钟）	流动相 A（%）	流动相 B（%）
0	70	30
5	70	30
13	80	20
33	100	0
40	100	0

参照物溶液的制备 取昆布对照药材 2.0g，加水 100ml，回流提取 30 分钟，放冷，滤过，滤液减压浓缩至干，残渣加入水 10ml，超声处理 30 分钟，取出，

放冷，用乙酸乙酯萃取 2 次，每次 20ml，合并萃取液，蒸干，用 2ml 甲醇溶解，即得，作为对照药材参照物溶液。另取亚油酸对照品适量，精密称定，加甲醇制成每 1ml 含 0.1mg 的溶液，作为对照品参照物溶液。

供试品溶液的制备 取本品适量，研细，取约 1g，加水 20ml，超声处理 40 分钟，放冷，用乙酸乙酯萃取 2 次，每次 20ml，合并萃取液，蒸干，用 2ml 甲醇溶解，即得。

测定法 分别精密吸取参照物溶液与供试品溶液各 15μl，注入液相色谱仪，测定，即得。

供试品色谱图中应呈现与对照药材参照物色谱图中 9 个保留时间相对应的特征峰，峰 7 应与对照品参照物峰保留时间相对应。峰 1~6、峰 8～9 与 S 峰（峰 7）的相对保留时间依次约为：0.51、0.62、0.75、0.79、0.81、0.94、1.14、1.40。

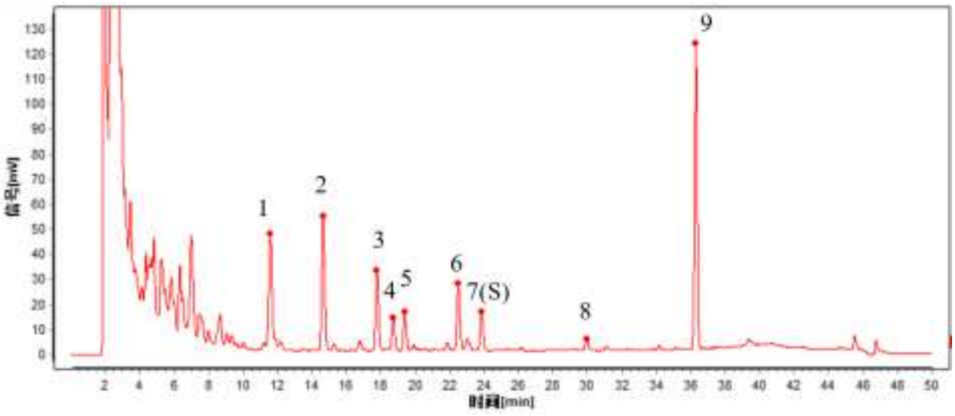

对照特征图谱

峰 7（S）：亚油酸

色谱柱：Extend-C18，4.6mm×250mm，5μm

【检查】 应符合颗粒剂（中国药典 通则 0104）项下有关的各项规定。

重金属及有害元素 照铅、镉、砷、汞、铜测定法（中国药典 通则 2321）原子吸收分光光度法或电感耦合等离子体质谱法测定，铅不得过 5mg/kg；镉不得过 4mg/kg；汞不得过 0.1mg/kg；铜不得过 20mg/kg。

【浸出物】 照醇溶性浸出物测定法（中国药典 通则 2201）项下的热浸法测定，用乙醇作溶剂，不得少于 15.0%。

【含量测定】 照高效液相色谱法（中国药典 通则 0512）测定。

色谱条件与系统适用性试验 以十八烷基硅烷键合硅胶为填充剂（柱长为 100mm，内径为 2.1mm，粒径为 1.8μm）；以乙腈为流动相 A，以 0.1%甲酸和

5mmol/L 醋酸铵溶液为流动相 B，按下表梯度洗脱；流速为 0.4ml/min；检测波长为 246nm；柱温为 30℃。理论板数按半乳糖峰计应不低于 5000。

时间（分钟）	流动相 A（%）	流动相 B（%）
0	15	85
9	18.5	81.5
13	18.5	81.5
25	25	75

对照品溶液的制备 取半乳糖对照品适量，精密称定，加水制成每 1ml 含 6μg 的溶液。精密量取 200μl，精密加入 0.5mol/L 的 PMP（1-苯基-3-甲基-5-吡唑啉酮）甲醇溶液与 0.2mol/L 的氢氧化钠溶液各 160μl，混匀，70℃水浴反应 30 分钟，放冷，再精密加入 0.2mol/L 的盐酸溶液 160μl，混匀，用三氯甲烷洗涤 3 次，每次 1ml，漩涡混匀 10 秒间隔 5 秒，静置，弃去三氯甲烷液，水层离心后，取上清液，即得。

供试品溶液的制备 取本品适量，研细，取约 0.2g，精密称定，置具塞锥形瓶中，精密加入水 25ml，称定重量，超声处理（功率 250W，频率 40kHz）30 分钟，放冷，再称定重量，用水补足减失的重量，摇匀，离心（转速为每分钟 4000 转）10 分钟。精密量取上清液 1ml，置西林瓶中，加 2mol/L 三氟乙酸溶液 0.5ml，密封，110℃水解 4 个小时，放冷，加 2mol/L 氢氧化钠溶液 880μl，转移至 10ml 量瓶中，用少量水分次洗涤容器和残渣，洗液并入同一量瓶中，加水至刻度，摇匀，离心（转速为每分钟 12000 转）5 分钟。精密量取上清液 200μl，按对照品溶液的制备方法，自“精密加入 0.5mol/L 的 PMP 甲醇溶液”起，同法操作，取上清液，即得。

测定法 分别精密吸取对照品溶液与供试品溶液各 5μl，注入液相色谱仪，测定，即得。

本品每 1g 含半乳糖（$C_6H_{12}O_6$）应为 4.0~12.0mg。

【规格】 每 1g 配方颗粒相当于饮片 3.8g

【贮藏】 密封。

江苏省药品监督管理局

中药配方颗粒标准

JS-YBZ-2024402

龙胆草配方颗粒

Longdancao　Peifangkeli

【来源】 本品为龙胆科植物头花龙胆 *Gentiana cephalantha* Franch.的干燥全草经炮制并按标准汤剂的主要质量指标加工制成的配方颗粒。

【制法】 取龙胆草饮片 4500g，加水煎煮，滤过，滤液浓缩成清膏（出膏率为 13~22%），加辅料适量，干燥（或干燥，粉碎），再加辅料适量，混匀，制粒，制成 1000g，即得。

【性状】 本品为棕黄色至黄棕色的颗粒，气微香，味苦。

【鉴别】 取本品0.5g，研细，加入甲醇20ml，超声处理20分钟，滤过，取续滤液作为供试品溶液。另取龙胆草对照药材1.0g，加水50ml，煎煮30分钟，滤过，滤液蒸干，残渣加甲醇5ml溶解，作为对照药材溶液。再取龙胆苦苷对照品，加甲醇制成每1ml含1mg的溶液，作为对照品溶液。照薄层色谱法（中国药典 通则0502）试验，吸取对照品溶液5μl、对照药材溶液和供试品溶液各10μl，分别点于同一硅胶GF_{254}薄层板上，以乙酸乙酯-甲醇-水（20:2:1）为展开剂，展开，取出，晾干，置紫外光灯（254 nm）下检视。供试品色谱中，在与对照药材色谱和对照品色谱相应的位置上，显相同颜色的斑点。

【特征图谱】 照高效液相色谱法（中国药典 通则 0512）测定。

色谱条件与系统适用性试验 以十八烷基硅烷键合硅胶为填充剂；以甲醇为流动相 A，以 0.05%冰乙酸为流动相 B，按下表梯度洗脱；检测波长为 240nm；柱温为 30℃。理论板数按龙胆苦苷峰计应不低于 5000。

时间（分钟）	流动相 A（%）	流动相 B（%）
0	10	90
10	15	85
20	20	80
35	25	75
70	65	35

参照物溶液的制备 取龙胆草对照药材 0.5g，加水 50ml，煎煮 30 分钟，滤过，滤液蒸干，残渣加甲醇 25ml，密塞，超声处理 30 分钟，放冷，滤过，取续

滤液，作为对照药材参照物溶液。另取龙胆苦苷、异荭草苷对照品适量，精密称定，加甲醇制成每 1ml 各含 20μg 的混合溶液，作为对照品参照物溶液。

供试品溶液的制备 取本品适量，研细，取约 0.1g，精密称定，置具塞锥形瓶中，精密加入甲醇 50ml，密塞，称定重量，超声处理（功率 600W，频率 40kHz）30 分钟，放冷，再称定重量，用甲醇补足减失的重量，摇匀，滤过，取续滤液，即得。

测定法 分别精密吸取参照物溶液与供试品溶液各 5μl，注入液相色谱仪，测定，即得。

供试品色谱图中应呈现与对照药材参照物色谱图中 11 个保留时间相对应的特征峰，峰 6、峰 10 应分别与相应对照品参照物峰的保留时间相对应。峰 1~5 与 S1 峰（峰 6）的相对保留时间依次约为：0.25、0.40、0.50、0.76、0.88；峰 7~9、峰 11 与 S2 峰（峰 10）的相对保留时间依次为：0.72、0.91、0.94、1.14。

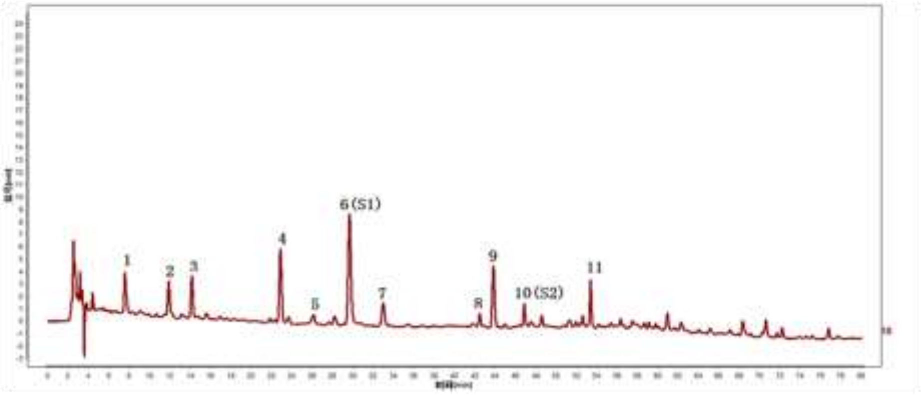

对照特征图谱

峰 4：马钱苷酸　峰 5：獐牙菜苦苷　峰 6（S1）：龙胆苦苷　峰 7：当药苷　峰 10（S2）：异荭草苷

色谱柱：Eclipse XDB C18（250mm×4.6 mm,5 μm）

【**检查**】 应符合颗粒剂项下有关的各项规定（中国药典 通则 0104）。

【**浸出物**】 照醇溶性浸出物测定法（中国药典 通则 2201）项下的热浸法测定，用乙醇作溶剂，不得少于 25.0%。

【**含量测定**】 照高效液相色谱法（中国药典 通则 0512）测定。

色谱条件与系统适用性试验 以十八烷基硅烷键合硅胶为填充剂；以甲醇-水（20：80）为流动相；检测波长为 270nm；柱温为 30℃。理论板数按龙胆苦苷峰计应不低于 3000。

对照品溶液的制备 取龙胆苦苷对照品适量，精密称定，加甲醇制成每 1ml 含 20μg 的溶液，摇匀，即得。

供试品溶液的制备 同【特征图谱】项。

测定法 分别精密吸取对照品溶液与供试品溶液各 5μl，注入液相色谱仪，测定，即得。

本品每1g含龙胆苦苷（$C_{16}H_{20}O_9$）应为6.0-27.0mg。

【规格】 每1g配方颗粒相当于饮片4.5g

【贮藏】 密封。

江苏省药品监督管理局

中药配方颗粒标准

JS-YBZ-2024403

龙葵配方颗粒

Longkui Peifangkeli

【来源】 本品为茄科植物龙葵 *Solanum nigrum L.*的干燥地上部分经炮制加工并按标准汤剂的主要质量指标加工制成的配方颗粒。

【制法】 取龙葵饮片 5500g，加水煎煮，滤过，滤液浓缩成清膏（出膏率为 11~18%），加入辅料适量，干燥（或干燥，粉碎），再加入辅料适量，混匀，制粒，制成 1000g，即得。

【性状】 本品为黄棕色至棕褐色的颗粒；气微，味苦。

【鉴别】 取本品适量，研细，取 0.2g，加乙醇 30ml，加热回流 30 分钟，滤过，滤液蒸干，残渣加乙醇 1ml 使溶解，作为供试品溶液。另取龙葵对照药材 2g，加水 50ml，煮沸 30 分钟，滤过，滤液蒸干，残渣加乙醇 30ml，同法制成对照药材溶液。照薄层色谱法（中国药典 通则 0502）试验，吸取上述两种溶液各 5µl，分别点于同一硅胶 G 薄层板上，以甲苯-乙酸乙酯-甲醇-异丙醇-氨水（6∶3∶1.5∶1.5∶0.5）为展开剂，展开，取出，晾干，喷以 10%硫酸乙醇溶液，置紫外灯（365nm）下检视。供试品色谱中，在与对照药材色谱相应的位置上，显相同颜色的荧光斑点。

【特征图谱】 照高效液相色谱法（中国药典 通则 0512）测定。

色谱条件与系统适用性试验 以十八烷基硅烷键合硅胶为填充剂；以乙腈为流动相 A，以 0.01%磷酸溶液为流动相 B，按下表梯度洗脱；流速为 0.3ml/min；检测波长为 325nm；柱温为 40℃。理论板数按绿原酸峰计应不低于 5000。

时间（分钟）	流动相 A(%)	流动相 B(%)
0	6	94
19	20	80
35	30	70
39	60	40
45	60	40

参照物溶液的制备 取龙葵对照药材 1g，加 80%甲醇 25ml，超声处理 30

分钟，放冷，滤过，取续滤液，作为对照药材参照物溶液。另取绿原酸、咖啡酸乙酯对照品适量，精密称定，加甲醇制成每 1ml 各含 40μg 的溶液，作为对照品参照物溶液。

供试品溶液的制备　取本品适量，研细，取约 0.2g，精密称定，置具塞锥形瓶中，精密加入 80%甲醇 25ml，称定重量，超声处理（功率 300W，频率 40kHz）30 分钟，放冷，再称定重量，用 80%甲醇补足减失的重量，摇匀，滤过，取续滤液，即得。

测定法　分别精密吸取参照物溶液和供试品溶液各 1μl，注入液相色谱仪，测定，即得。

供试品色谱图中应呈现与对照药材参照物色谱图中 6 个保留时间相对应的特征峰，峰 1、峰 5 应分别与相应对照品参照物峰的保留时间相对应。峰 2～3 与 S1 峰（峰 1）的相对保留时间依次约为：1.64、1.94；峰 4、峰 6 与 S2 峰（峰 5）的相对保留时间依次约为：0.97、1.27。

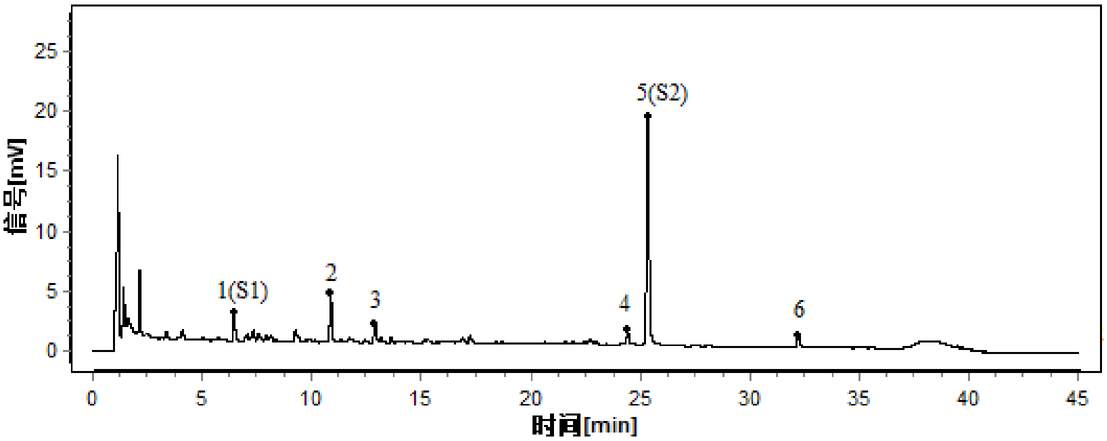

对照特征图谱

峰 1（S1）：绿原酸　峰 5（S2）：咖啡酸乙酯

色谱柱：CORTECS T3（150mm×2.1mm，1.6μm）

【检查】　应符合颗粒剂（中国药典 通则 0104）项下有关的各项规定。

【浸出物】　取本品适量，研细，取约 2g，精密称定，精密加入乙醇 100ml，照醇溶性浸出物测定法（中国药典 通则 2201）项下的热浸法测定，不得少于 25.0%。

【含量测定】　照高效液相色谱法（中国药典 通则 0512）测定。

色谱条件与系统适用性试验　以苯基键合亚乙基桥杂化颗粒或苯基键合硅胶为填充剂；以乙腈-1mmol/L 磷酸氢二钠（38：62）为流动相；流速为每分钟 0.8ml；检测波长为 203nm；柱温为 25℃。理论板数按澳洲茄碱峰计应不低于

3000。

对照品溶液的制备 取澳洲茄碱、澳洲茄边碱对照品适量，精密称定，加甲醇制成每 1ml 各含 100μg 的溶液，即得。

供试品溶液的制备 同【特征图谱】项。

测定法 分别精密吸取对照品溶液与供试品溶液各 10μl，注入液相色谱仪，测定，即得。

本品每 1g 含澳洲茄碱（$C_{45}H_{73}NO_{16}$）和澳洲茄边碱（$C_{45}H_{73}NO_{15}$）的总量应为 10~40mg。

【规格】 每 1g 配方颗粒相当于饮片 5.5g

【贮藏】 密封。

江苏省药品监督管理局

中药配方颗粒标准

JS-YBZ-2024404

青天葵配方颗粒

Qingtiankui　Peifangkeli

【来源】 本品为兰科植物毛唇芋兰 *Nervilia plicata* (Andr.) Schltr.的干燥全草经炮制并按标准汤剂的主要质量指标加工制成的配方颗粒。

【制法】 取青天葵饮片 4000g，加水煎煮，滤过，滤液浓缩成清膏（出膏率为 13~24%），加入辅料适量，干燥（或干燥，粉碎），再加入辅料适量，混匀，制粒，制成 1000g，即得。

【性状】 本品为浅黄棕色至棕褐色的颗粒；气微，味微苦。

【鉴别】 取本品 1g，研细，加水 30ml 使溶解，加盐酸 5ml，加热回流 1 小时，放冷，用乙酸乙酯振摇提取 2 次，每次 20ml，合并乙酸乙酯液，蒸干，残渣加甲醇 1ml 使溶解，作为供试品溶液。另取青天葵对照药材 2g，加水 80ml，煮沸 30 分钟，滤过，滤液浓缩至 30ml，加盐酸 5ml，同法制成对照药材溶液。照薄层色谱法（中国药典 通则 0502）试验，吸取供试品溶液 2μl，对照药材溶液 4μl，分别点于同一用 1%氢氧化钠溶液制备的硅胶 G 薄层板上，以正己烷-甲苯-乙酸乙酯-甲酸（10︰20︰12︰2）为展开剂，展开，取出，晾干，喷以三氯化铝试液，置紫外光灯（365nm）下检视。供试品色谱中，在与对照药材色谱相应的位置上，显相同颜色的荧光主斑点。

【特征图谱】 照高效液相色谱法（中国药典 通则 0512）测定。

色谱条件与系统适用性试验　以十八烷基键合硅胶为填充剂；以乙腈为流动相 A，以 0.2%甲酸溶液为流动相 B，按下表梯度洗脱；流速为 0.3ml/min；检测波长为 256nm；柱温为 40℃。理论板数按沙苑子苷 A 峰计应不低于 5000。

时间（分钟）	流动相 A（%）	流动相 B（%）
0	5	95
10	21	79
15	24	76
20	24	76
22	30	70
32	100	0
35	100	0

参照物溶液的制备　取青天葵对照药材 1g，加水 50ml，加热回流 30 分钟，滤过，滤液蒸干，残渣加 75%甲醇 25ml，超声处理 30 分钟，放冷，滤过，取续滤液，作为对照药材参照物溶液。另取沙苑子苷 A、鼠李柠檬素对照品适量，精密称定，加甲醇制成每 1ml 含沙苑子苷 A 20μg、鼠李柠檬素 40μg 的混合溶液，作为对照品参照物溶液。

供试品溶液的制备　取本品适量，研细，取约 0.2g，精密称定，置具塞锥形瓶中，精密加入 75%甲醇 25ml，称定重量，超声处理（功率 300W，频率 40kHz）30 分钟，放冷，再称定重量，用 75%甲醇补足减失的重量，摇匀，滤过，取续滤液，即得。

测定法　精密吸取参照物溶液和供试品溶液各 1μl，注入液相色谱仪，测定，即得。

供试品色谱中应呈现与对照药材参照物色谱涂中的 4 个保留时间相对应的特征峰，峰 3、峰 4 应分别与相应对照品参照物峰的保留时间相对应。峰 2 与 S 峰（峰 3）的相对保留时间约为：0.69。

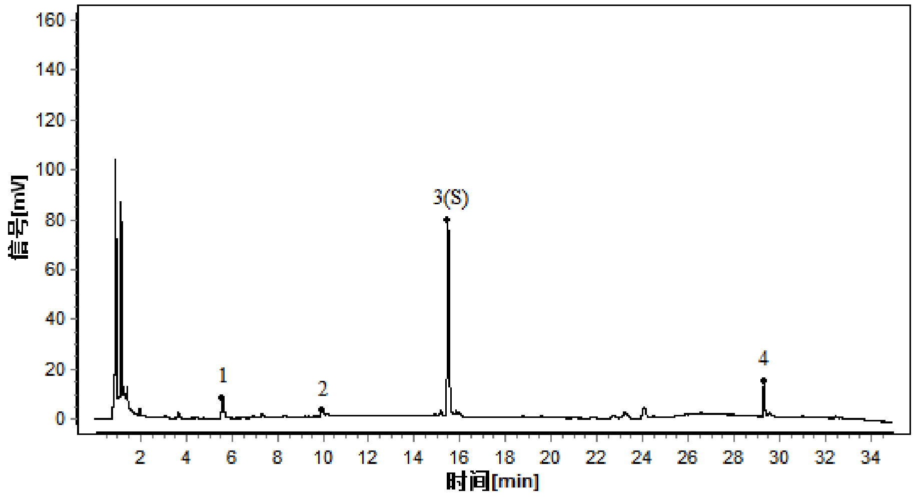

对照特征图谱

峰 3：沙苑子苷 A　峰 4：鼠李柠檬素

色谱柱：HSS T3 （150mm×2.1mm，1.8μm）

【检查】 应符合颗粒剂（中国药典 通则 0104）项下有关的各项规定。

【浸出物】 取本品适量，研细，取约 2g，精密称定，精密加入乙醇 100ml，照醇溶性浸出物测定法项下的热浸法（中国药典 通则 2201）测定，不得少于 8.0%。

【含量测定】 照高效液相色谱法（中国药典 通则 0512）测定。

色谱条件与系统适用性试验　同【特征图谱】项。

对照品溶液的制备　取沙苑子苷 A 对照品适量，精密称定，加 75%甲醇制成每 1ml 含沙苑子苷 A 20μg 的溶液，即得。

供试品溶液的制备　同【特征图谱】项。

测定法　精密吸取对照品溶液与供试品溶液各 1μl，注入液相色谱仪，测定，即得。

本品每 1g 含沙苑子苷 A（$C_{27}H_{30}O_{18}$）应为 3.0~15.0mg。

【规格】　每 1g 配方颗粒相当于饮片 4g

【贮藏】　密封。

江苏省药品监督管理局

中药配方颗粒标准

JS-YBZ-2024405

山枝仁(皱叶海桐）配方颗粒

Shanzhiren（Zhouyehaitong） Peifangkeli

【来源】 本品为海桐花科海桐花属植物皱叶海桐 *Pittosporum crispulum* Gagnep.的干燥种子经炮制并按标准汤剂的主要质量指标加工制成的配方颗粒。

【制法】 取山枝仁（皱叶海桐）饮片 10000g，加水煎煮，滤过，滤液浓缩成清膏（出膏率为 6~10%），加入辅料适量，干燥（或干燥、粉碎），再加入辅料适量，混匀，制粒，制成 1000g，即得。

【性状】 本品为淡黄色至淡红棕色的颗粒；气微，味涩，微苦。

【鉴别】 取本品 0.2g，研细，加甲醇 10ml，超声处理 30 分钟，滤过，取滤液作为供试品溶液。另取山枝仁（皱叶海桐）对照药材 1.0g，加水 50ml，煮沸 30 分钟，离心，取上清液蒸干，残渣加甲醇 5ml 溶解，过滤，作为对照药材溶液。照薄层色谱法(中国药典 通则 0502)试验，吸取对照药材溶液 8ul，供试品溶液 10ul，分别点于同一硅胶 G 薄层板上，以甲苯-乙酸乙酯-甲酸（7:3:1）为展开剂，展开，取出，晾干，喷以 5%三氯化铝乙醇试液，在 105℃加热至斑点显示清晰，置紫外光灯(365nm)下检视。供试品色谱中，在与对照药材色谱相应的位置上，显相同颜色的荧光斑点。

【特征图谱】照高效液相色谱法（中国药典 通则 0512）测定。

色谱条件与系统适用性试验 以十八烷基硅烷键合硅胶为填充剂；以甲醇为流动相 A，以 0.2%磷酸溶液为流动相 B，按下表梯度洗脱；检测波长为 310nm；柱温为 30℃。理论板数按异槲皮苷峰计应不低于 5000。

时间（分钟）	流动相 A（%）	流动相 B（%）
0	12	88
5	12	88
30	33	67
50	55	45
60	55	45

参照物溶液的制备　取山枝仁（皱叶海桐）对照药材0.5g，加70%甲醇25ml，加热回流30分钟，放冷，滤过，取续滤液，作为对照药材参照物溶液。另取异槲皮苷对照品适量，精密称定，加甲醇制成每1ml含50μg的溶液，作为对照品参照物溶液。

供试品溶液的制备　取本品适量，研细，取约0.5g，精密称定，置具塞锥形瓶中，精密加入70%甲醇25ml，密塞，称定重量，加热回流30分钟，放冷，再称定重量，用70%甲醇补足减失的重量，摇匀，滤过，取续滤液，即得

测定法　精密吸取参照物溶液与供试品溶液各10μl，注入液相色谱仪，测定，即得。

供试品色谱中应呈现与对照药材参照物色谱中 9 个保留时间相对应的特征峰，其中峰6应与对照品参照物色谱峰的保留时间相对应。峰1~5、峰7~9与S峰（峰6）的相对保留时间依次约为：0.36、0.46、0.56、0.58、0.73、1.03、1.09、1.10。

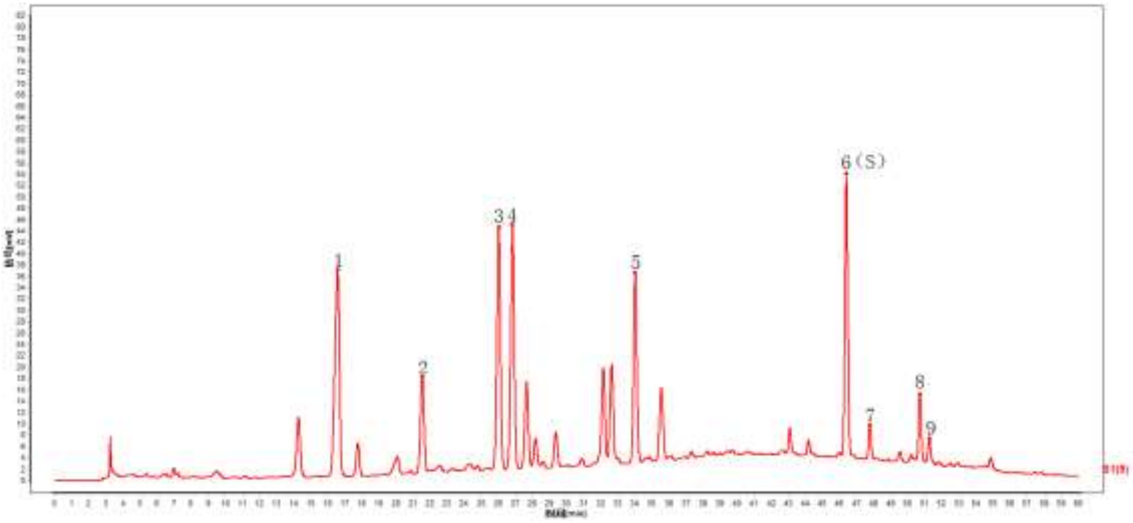

对照特征图谱

峰3：绿原酸　峰4：隐绿原酸　峰6（S）：异槲皮苷

色谱柱：5 TC-C18(2)（250mm×4.6mm，5μm）

【检查】　应符合颗粒剂（中国药典 通则0104）项下有关的各项规定。

【浸出物】　照醇溶性浸出物测定法（中国药典 通则2201）项下的热浸法测定，用乙醇作溶剂，不得少于14.0%。

【含量测定】　照高效液相色谱法（中国药典 通则0512）测定。

色谱条件与系统适用性试验　以十八烷基键合硅胶为填充剂；以甲醇为流动相A，以0.2%磷酸溶液为流动相B，按下表梯度洗脱；检测波长为355nm；柱

温为 30℃。理论板数按异槲皮苷峰计应不低于 5000。

时间（分钟）	流动相 A（%）	流动相 B（%）
0	25	75
10	40	60
30	60	40

对照溶液的制备　同【特征图谱】项下的对照品参照物溶液。

供试品溶液制备　同【特征图谱】项。

测定法　精密吸取对照品溶液与供试品溶液各 10μl，注入液相色谱仪，测定，即得。

本品每 1g 含异槲皮苷（$C_{21}H_{20}O_{12}$）应为 1.5~4.5mg。

【规格】　每 1g 配方颗粒相当于饮片 10g

【贮藏】　密封。

江苏省药品监督管理局

中药配方颗粒标准

JS-YBZ-2024406

水牛角配方颗粒

Shuiniujiao　Peifangkeli

【来源】 本品为牛科动物水牛 *Bubalus bubalis* Linnaeus 的角经炮制并按标准汤剂的主要质量指标加工制成的配方颗粒。

【制法】 取水牛角饮片 20000g，加水煎煮，滤过，滤液浓缩成清膏（出膏率为 0.8~1.5%），加辅料适量，干燥（或干燥，粉碎），再加入辅料适量，混匀，制粒，制成 1000g，即得。

【性状】 本品为黄白色至灰黄色的颗粒；气微腥，味淡。

【鉴别】 （1）取本品 0.2g，研细，加入 70%乙醇 10ml，超声处理 20 分钟，滤过，取滤液作为供试品溶液。另取水牛角对照药材 1g，加入 70%乙醇 10ml，超声处理 20 分钟，滤过，滤液蒸干，残渣加 70%乙醇 1ml 使溶解，作为对照药材溶液。照薄层色谱法（中国药典 通则 0502）试验，吸取上述两种溶液各 10μl，分别点于同一硅胶 G 薄层板上，以正丁醇-冰醋酸-水（3∶1∶1）为展开剂，展开，取出，晾干，喷以 0.2%茚三酮乙醇溶液，在 105℃加热至斑点清晰。供试品色谱中，在与对照药材色谱相应的位置上，显相同颜色的斑点。

（2）水牛角 照高效液相色谱-质谱法（中国药典 通则 0512 和通则 0431）测定。

色谱条件与系统适用性试验 以十八烷基硅烷键合硅胶为填充剂（柱长为 100mm，内径为 2.1mm，粒径为 1.7μm)；以乙腈为流动相 A，以 0.1%甲酸溶液为流动相 B，按下表梯度洗脱；流速为 0.3ml/min；柱温为 35℃；采用质谱检测器：电喷雾离子化（ESI）正离子模式下多反应监测（MRM），选择质荷比（m/z）544.8（双电荷）→663.5 和 544.8（双电荷）→734.6 作为检测离子对，取水牛角对照药材溶液，进样 5μl，按上述检测离子对测定的 MRM 色谱峰的信噪比均应大于 3∶1。

时间（分钟）	流动相 A（%）	流动性 B（%）
0	5	95
8	20	80
12	50	50

参照物溶液的制备 取水牛角对照药材 1g，加水 50ml，加热回流 3 小时，滤过，滤液蒸干，残渣加水 5ml 使溶解，取适量转移至离心管中，离心（12000r/min）10 分钟，取上清液 500μl，转移至离心管中，加胰蛋白酶溶液（取序列分析用胰蛋白酶，加 1%碳酸氢铵溶液制成每 1ml 中含 0.1mg 的溶液，临用时配制）500μl，摇匀，超声处理（功率 250W，频率 40kHz）30 分钟，离心（12000r/min）5 分钟，取上清液，作为对照药材参照物溶液。

供试品溶液的制备 取本品适量，研细，取约 0.5g，精密称定，加 1%碳酸氢铵溶液 10ml，超声处理（功率 250W，频率 40kHz）30 分钟，取适量转移至离心管中，离心（12000r/min）10 分钟，取上清液 500μl，转移至离心管中，加胰蛋白酶溶液（取序列分析用胰蛋白酶，加 1%碳酸氢铵溶液制成每 1ml 中含 0.1mg 的溶液，临用时配制）500μl，摇匀，超声处理（功率 250W，频率 40kHz）30 分钟，离心（12000r/min）5 分钟，取上清液，即得。

测定法 精密吸取参照物溶液与供试品溶液各 5μl，注入高效液相色谱-质谱联用仪，测定。

以质荷比（m/z）544.8（双电荷）→663.5 和（m/z）544.8（双电荷）→734.6 离子对提取的供试品离子流色谱图中，应同时呈现与对照药材色谱保留时间一致的色谱峰。

【特征图谱】 照高效液相色谱法（中国药典 通则 0512）测定。

色谱条件与系统适用性试验 以十八烷基硅烷键合硅胶为填充剂；以 10mmol/L 磷酸氢二钠-硼酸钠缓冲溶液（取无水磷酸氢二钠 1.4g 和十水硼酸钠 3.8g，加水 1000ml 溶解，混匀，用 5%磷酸调节 pH 值至 7.2）为流动相 A，以甲醇-乙腈-水（45∶45∶10）为流动相 B，按下表梯度洗脱；检测波长为 338nm；柱温为 35℃。理论板数按丙氨酸峰计应不低于 5000。

时间（分钟）	流动相 A（%）	流动相 B（%）
0	98	2
12	76	24
26	45	55

参照物溶液的制备 取水牛角对照药材 0.5g，加 0.1mol/L 盐酸溶液 50ml，加

热回流2小时，取出，放冷，静置，取上清液2ml，加盐酸2ml，150℃水解2.5小时，放冷，移至蒸发皿中，用水10ml分次洗涤，洗液并入蒸发皿中，蒸干，残渣加流动相A溶解，转移至5ml量瓶中，加流动相A至刻度，摇匀，滤过，取续滤液，作为对照药材参照物溶液。另取丝氨酸、甘氨酸、精氨酸、丙氨酸、酪氨酸、亮氨酸对照品适量，精密称定，加0.1mol/L盐酸溶液制成每1ml含丝氨酸35μg、甘氨酸35μg、精氨酸30μg、丙氨酸30μg、酪氨酸25μg、亮氨酸25μg的混合溶液，作为对照品参照物溶液。

供试品溶液的制备 取本品适量，研细，取约0.5g，精密称定，置具塞锥形瓶中，精密加入0.1mol/L盐酸溶液50ml，密塞，称定重量，超声处理（功率250W，频率40kHz）30分钟，放冷，再称定重量，用0.1mol/L盐酸溶液补足减失的重量，摇匀，静置。精密量取上清液2ml，加盐酸2ml，150℃水解1.5小时，放冷，移至蒸发皿中，用水10ml分次洗涤，洗液并入蒸发皿中，蒸干，残渣加流动相A溶解，转移至5ml量瓶中，加流动相A稀释至刻度，摇匀，即得。

测定法 精密吸取对照品溶液与供试品溶液各2μl，按照下列衍生化程序进行测定：

1、稀释剂：取100ml流动相A，加0.4ml浓 H3PO4制成稀释剂。

2、0.4mol/L硼酸缓冲液（pH 10.2）：取硼酸2.47g，加水80ml溶解，用氢氧化钠试液调pH值为10.2，加水稀释至100ml作为硼酸缓冲液。

3、OPA衍生剂：取邻苯二甲醛（OPA）80mg，加入7ml 0.4mol/L硼酸缓冲液（pH 10.2），再加巯基丙酸125μl和乙腈1ml混匀，制成OPA衍生剂（本液需冷藏）。

4、柱前衍生化进样程序：

（1）从硼酸缓冲液样品瓶吸取2.5μl；

（2）从样品瓶中吸取2.0μl；

（3）在清洗口，以默认速度将4.5μl混合液混合5次；

（4）洗针；

（5）等待0.2分钟；

（6）从OPA衍生剂样品瓶吸取0.5μl；

（7）在清洗口，以默认速度将5.0μl混合液混合10次；

（8）从稀释剂样品瓶吸取 5.0μl；

（9）在清洗口，以默认速度将 10.0μl 混合液混合 8 次；

（10）注入液相色谱仪。

供试品色谱图中应呈现与对照药材参照物色谱图中 12 个保留时间相对应的特征峰，峰 2~3、峰 5~7、峰 11 应分别与相应对照品参照物峰的保留时间相对应。峰 1、峰 4、峰 8~10、峰 12 与 S 峰（峰 6）的相对保留时间依次约为：0.31、0.81、1.48、1.69、1.72、1.89。

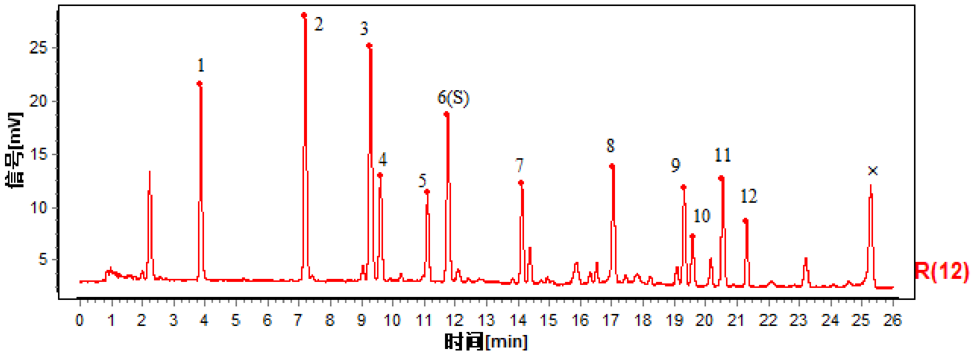

对照特征图谱

峰 1：谷氨酸　峰 2：丝氨酸　峰 3：甘氨酸　峰 4：苏氨酸　峰 5：精氨酸

峰 6（S）：丙氨酸　峰 7：酪氨酸　峰 11：亮氨酸　×：衍生化试剂峰

色谱柱：Poroshell HPH-C18（100mm×4.6mm，2.7μm）

【检查】 应符合颗粒剂（中国药典 通则 0104）项下有关的各项规定。

【浸出物】 照醇溶性浸出物测定法（中国药典 通则 2201）项下的热浸法测定，用乙醇作溶剂，不得少于 6.0%。

【含量测定】 照高效液相色谱法（中国药典 通则 0512）测定。

色谱条件与系统适用性试验 同【特征图谱】项。

对照品溶液的制备 同【特征图谱】项下的对照品参照物溶液。

供试品溶液的制备 同【特征图谱】项。

测定法 同【特征图谱】项。

本品每 1g 含丝氨酸（$C_3H_7NO_3$）应为 2.5～8.5mg；含甘氨酸（$C_2H_5NO_2$）应为 2.5～8.0mg；含精氨酸（$C_6H_{14}N_4O_2$）应为 2.0～6.5mg；含丙氨酸（$C_3H_7NO_2$）应为 1.5～4.5mg；含酪氨酸（$C_9H_{11}NO_3$）应为 1.5～7.0mg；含亮氨酸（$C_6H_{13}NO_2$）应为 1.5～5.0mg。

【规格】 每 1g 配方颗粒相当于饮片 20g

【贮藏】 密封。

江苏省药品监督管理局

中药配方颗粒标准

JS-YBZ-2024407

水蛭（蚂蟥）配方颗粒

Shuizhi(Mahuang)　Peifangkeli

【来源】 本品为水蛭科动物蚂蟥 *Whitmania pigra* Whitman 的干燥全体经炮制并按标准汤剂的主要质量指标加工制成的配方颗粒。

【制法】 取水蛭（蚂蟥）饮片 4000g，加水煎煮，滤过，滤液浓缩成清膏（出膏率为 12~20%），加入辅料适量，干燥（或干燥，粉碎），再加入辅料适量，混匀，制粒，制成 1000g，即得。

【性状】 本品为灰黄色至浅棕褐色的颗粒；气微腥，味淡。

【鉴别】 取本品适量，研细，取 1g，加乙醇 30ml，超声处理 30 分钟，滤过，滤液蒸干，残渣加乙醇 1ml 使溶解，作为供试品溶液。另取水蛭（蚂蟥）对照药材 1g，加水 25ml，煎煮 30 分钟，滤过，蒸干，加乙醇 30ml，同法制成对照药材溶液。再取缬氨酸、丙氨酸对照品，加水制成每 1ml 各含 0.5mg 的混合溶液，作为对照品溶液。照薄层色谱法（中国药典 通则 0502）试验，吸取供试品溶液 3μl，对照药材溶液和对照品溶液各 2μl，分别点于同一硅胶 G 薄层板上，以水饱和正丁醇-冰醋酸（4:1）为展开剂，展开，取出，晾干，喷以茚三酮试液，在 105℃加热至斑点显色清晰。供试品色谱中，在与对照药材和对照品色谱相应的位置上，显相同颜色的斑点。

【特征图谱】 照高效液相色谱法（中国药典 通则 0512）测定。

色谱条件与系统适用性试验 以十八烷基硅烷键合硅胶为填充剂；以乙腈-0.2%磷酸溶液（0.5:99.5）为流动相；流速为 0.8ml/min；检测波长为 270nm；柱温 25℃。理论板数按次黄嘌呤峰计应不低于 5000。

参照物溶液的制备 取水蛭（蚂蟥）对照药材 1.5g，加水 20ml，加热回流 1 小时，放冷，离心处理（转速为每分钟 10000 转）10 分钟，取出，取上清液滤过，作为对照药材参照物溶液。另取次黄嘌呤、尿嘧啶对照品适量，加 10%甲醇制成每 1ml 各含 0.2mg 的混合对照品溶液，作为对照品参照物溶液。

供试品溶液的制备 取本品适量，研细，取约 0.3g，置具塞锥形瓶中，加入水 10ml，超声处理 10 分钟，放冷，离心处理（转速为每分钟 10000 转）10 分钟，取出，取上清液，滤过，取续滤液，即得。

测定法 分别精密吸取参照物溶液与供试品溶液各 10μl，注入液相色谱仪，测定。

供试品色谱图中应呈现与对照药材参照物色谱图中 5 个保留时间相对应的特征峰，峰 2～3 分别应与相应对照品参照物峰的保留时间相对应。峰 1、峰 4～5 与 S 峰（峰 3）的相对保留时间依次约为：0.51、1.41、1.82。

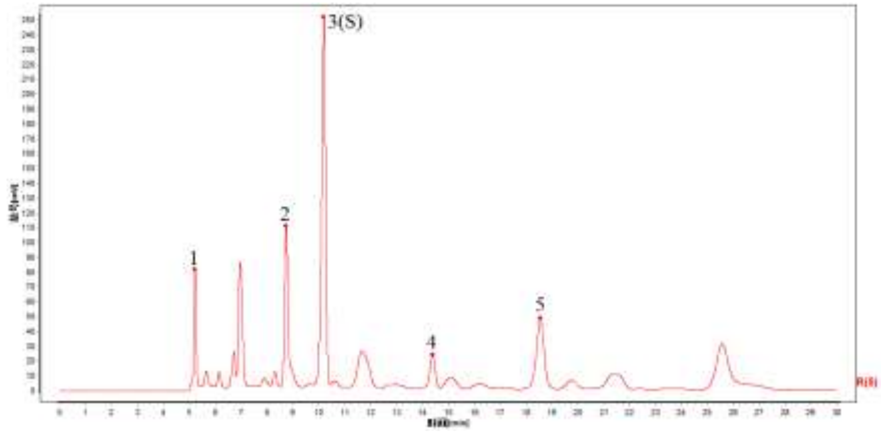

对照特征图谱

峰 2：尿嘧啶 峰 3（S）：次黄嘌呤

色谱柱：Dikma Platisil ODS C18（250mm×4.6mm，5.0 μm）

【检查】 酸碱度 取本品适量，研细，取 0.25g，加入 0.9%氯化钠溶液 10ml，充分搅拌，浸提 30 分钟，并时时振摇，离心，取上清液，照 pH 值测定法（中国药典 通则 0631）测定，应为 5.0～7.5。

重金属及有害元素 照铅、镉、砷、汞、铜测定法（中国药典 通则 2321 原子吸收分光光度法或电感耦合等离子体质谱法）测定，铅不得过 10mg/kg、镉不得过 1mg/kg、砷不得过 20mg/kg、汞不得过 1mg/kg。

黄曲霉毒素 照黄曲霉毒素测定法（中国药典 通则 2351）测定。

本品每 1kg 含黄曲霉毒素 B1 不得过 5μg，黄曲霉毒素 G2、黄曲霉毒素 G1、黄曲霉毒素 B2 和黄曲霉毒素 B1 的总量不得过 10μg。

其他 应符合颗粒剂（中国药典 通则 0104）项下有关的各项规定。

【浸出物】 取本品适量，研细，取约 2g，精密称定，精密加入乙醇 100ml，照醇溶性浸出物测定法（中国药典 通则 2201）项下的热浸法测定，应不少于

10.0%。

【含量测定】 抗凝血酶活性 取本品适量，研细，取约 0.45g，精密称定，精密加入 0.9%氯化钠溶液 5ml，充分搅拌，浸提 30 分钟，并时时振摇，离心，精密量取上清液 100μl，置试管（8mm×38mm）中，加入含 0.5%（牛）纤维蛋白原（以凝固物计）的三羟甲基氨基甲烷盐酸缓冲液（临用配制，取 0.2mol/L 三羟甲基氨基甲烷溶液 25ml 与 0.1mol/L 盐酸溶液约 40ml，加水至 100ml，调节 pH 值至 7.4。）200μl，摇匀，置水浴中（37℃±0.5℃）温浸 5 分钟，滴加每 1ml 中含 10 单位的凝血酶溶液（临用配制，取凝血酶试剂适量，加生理盐水配制成每 1ml 含凝血酶 10 个单位的溶液）（每 4 分钟滴加 1 次，每次 2μl，边滴加边轻轻摇匀）至凝固，记录消耗凝血酶溶液的体积，按下式计算：

$$U = \frac{C_1 V_1}{C_2 V_2}$$

式中 U 为每 1g 含凝血酶活性单位，U/g；

C_1 为凝血酶溶液的浓度，U/ml；

C_2 为供试品溶液的浓度，g/ml；

V_1 为消耗凝血酶溶液的体积，μl；

V_2 为供试品溶液的加入量，μl。

中和一个单位的凝血酶的量，为一个抗凝血酶活性单位。

本品每 1g 含抗凝血酶活性应为 4.0U-13.5U。

【规格】 每 1g 配方颗粒相当于饮片 4 g

【贮藏】 密封。

江苏省药品监督管理局

中药配方颗粒标准

JS-YBZ-2024408

烫水蛭（蚂蟥）配方颗粒

Tangshuizhi(Mahuang)　Peifangkeli

【来源】 本品为水蛭科动物蚂蟥 *Whitmania pigra* Whitman 的干燥全体经炮制并按标准汤剂的主要质量指标加工制成的配方颗粒。

【制法】 取水蛭（蚂蟥）饮片 4000g，加水煎煮，滤过，滤液浓缩成清膏（出膏率为 12~20%），加入辅料适量，干燥（或干燥，粉碎），再加入辅料适量，混匀，制粒，制成 1000g，即得。

【性状】 本品为灰黄色至浅棕褐色的颗粒；气微腥，味淡。

【鉴别】 取本品适量，研细，取 1g，加乙醇 30ml，超声处理 30 分钟，滤过，滤液蒸干，残渣加乙醇 1ml 使溶解，作为供试品溶液。另取水蛭（蚂蟥）对照药材 1g，加水 25ml，煎煮 30 分钟，滤过，滤液蒸干，残渣加乙醇 30ml，同法制成对照药材溶液。再取缬氨酸、丙氨酸对照品，加水制成每 1ml 各含 0.5mg 的混合溶液，作为对照品溶液。照薄层色谱法（中国药典 通则 0502）试验，吸取供试品溶液 2μl、对照药材溶液 8μl、对照品溶液 3μl，分别点于同一硅胶 G 薄层板上，以水饱和正丁醇-冰醋酸（4∶1）为展开剂，展开，取出，晾干，喷以茚三酮试液，在 105℃加热至斑点显色清晰。供试品色谱中，在与对照药材色谱和对照品色谱相应的位置上，显相同颜色的斑点。

【特征图谱】 照高效液相色谱法（中国药典 通则 0512）测定。

色谱条件与系统适用性试验 以十八烷基硅烷键合硅胶为填充剂；以乙腈-0.2%磷酸溶液（0.5∶99.5）为流动相；流速为 0.8ml/min；检测波长为 270nm；柱温为 25℃。理论板数按次黄嘌呤峰计应不低于 5000。

参照物溶液的制备 取水蛭（蚂蟥）对照药材 1.5g，加水 20ml，加热回流 1 小时，放冷，离心处理（转速为每分钟 10000 转）10 分钟，取上清液，滤过，取续滤液，作为对照药材参照物溶液。另取次黄嘌呤、尿嘧啶对照品适量，加 10%甲醇制成每 1ml 各含 0.2mg 的混合溶液，作为对照品参照物溶液。

供试品溶液的制备　取本品适量，研细，取 0.3g，加水 10ml，超声处理 10 分钟，放冷，离心处理（转速为每分钟 10000 转）10 分钟，取出，取上清液，滤过，取续滤液，即得。

测定法　分别精密吸取参照物溶液与供试品溶液各 10μl,注入液相色谱仪，测定。

供试品色谱图中应呈现与对照药材参照物色谱图中 5 个保留时间相对应的特征峰，峰 2～3 分别应与相应对照品参照物峰的保留时间相对应。峰 1、4、5 与 S 峰（峰 3）的相对保留时间依次约为：0.51、1.41、1.82。

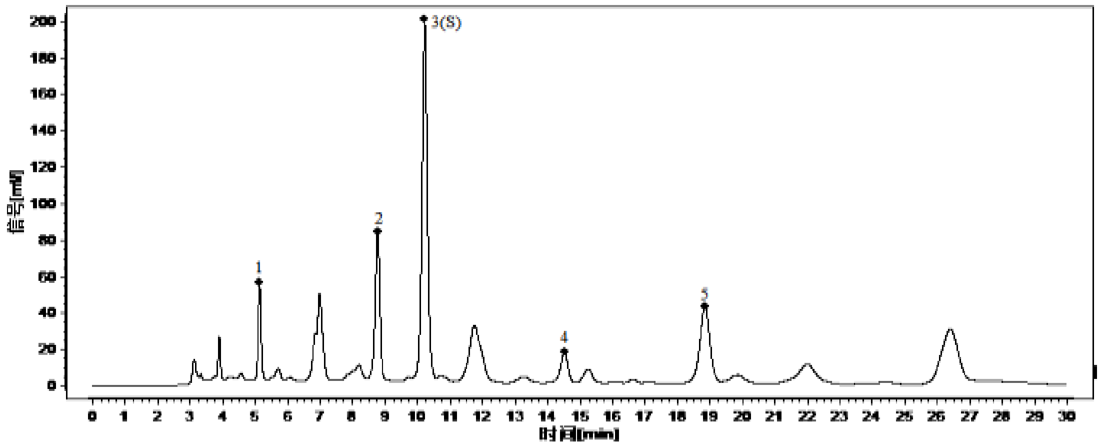

对照特征图谱

峰 2：尿嘧啶　峰 3（S）：次黄嘌呤

色谱柱：Platisil ODS C18（250mm×4.6mm，5.0 μm）

【检查】 **酸碱度**　取本品适量，研细，取 0.25g，加 0.9%氯化钠溶液 10ml，充分搅拌，浸提 30 分钟，并时时振摇，离心，取上清液，照 pH 值测定法（中国药典 通则 0631）测定，应为 5.0~7.5。

重金属及有害元素　照铅、镉、砷、汞、铜测定法（中国药典 通则 2321 原子吸收分光光度法或电感耦合等离子体质谱法）测定，铅不得过 10mg/kg、镉不得过 1mg/kg、砷不得过 20mg/kg、汞不得过 1mg/kg。

黄曲霉毒素　照黄曲霉毒素测定法（中国药典 通则 2351）测定。

本品每 1kg 含黄曲霉毒素 B1 不得过 5μg，黄曲霉毒素 G2、黄曲霉毒素 G1、黄曲霉毒素 B2 和黄曲霉毒素 B1 的总量不得过 10μg。

其他　应符合颗粒剂（中国药典 通则 0104）项下有关的各项规定。

【浸出物】 取本品适量，研细，取约 2g，精密称定，精密加入乙醇 100ml，照醇溶性浸出物测定法（中国药典 通则 2201）项下的热浸法测定，不得少于 9.0%。

【含量测定】 取本品适量，研细，取约 0.45g，精密称定，精密加入 0.9%氯化钠溶液 5ml，充分搅拌，浸提 30 分钟，并时时振摇，离心，精密量取上清液 100μl，置试管（8mm×38mm）中，加入含 0.5%（牛）纤维蛋白原（以凝固物计）的三羟甲基氨基甲烷盐酸缓冲液（临用配制，取 0.2mol/l 三羟甲基氨基甲烷溶液 25ml 与 0.1mol/L 盐酸溶液 40ml，加水至 100ml，调节 pH 值至 7.4）200μl，摇匀，置水浴中（37℃±0.5℃）温浸 5 分钟，滴加每 1ml 中含 10 单位的凝血酶溶液（临用配制，取凝血酶试剂，加生理盐水制成每 1ml 含凝血酶 10 个单位的溶液）（每 4 分钟滴加 1 次，每次 2μl，边滴加边轻轻摇匀）至凝固，记录消耗凝血酶溶液的体积，按下式计算：

$$U = \frac{C_1V_1}{C_2V_2}$$

式中　U 为每 1g 含凝血酶活性单位，U/g；

C_1 为凝血酶溶液的浓度，U/ml；

C_2 为供试品溶液的浓度，g/ml；

V_1 为消耗凝血酶溶液的体积，μl；

V_2 为供试品溶液的加入量，μl。

中和一个单位的凝血酶的量，为一个抗凝血酶活性单位。

本品每 1g 含抗凝血酶活性应为 4.0U~13.5U。

【规格】　每 1g 配方颗粒相当于饮片 4g

【贮藏】　密封。

江苏省药品监督管理局

中药配方颗粒标准

JS-YBZ-2024409

煨葛粉配方颗粒

Weigefen Peifangkeli

【来源】 本品为豆科植物甘葛藤 *Pueraria thomsonii* Benth 的干燥根经炮制并按标准汤剂的主要质量指标加工制成的配方颗粒。

【制法】 取煨粉葛饮片 4000g，加水煎煮，滤过，滤液浓缩成清膏（出膏率为 14~25%），加辅料适量，干燥（或干燥，粉碎），再加辅料适量，混匀，制粒，制成 1000g，即得。

【性状】 本品为浅黄色至棕黄色的颗粒；气微，味微苦。

【鉴别】 取本品 1g，加甲醇 20ml，超声处理 30 分钟，滤过，滤液挥干，残渣加甲醇 1ml 使溶解，作为供试品溶液。另取葛根素对照品，加甲醇制成每 1ml 含 1mg 的溶液，作为对照品溶液。照薄层色谱法（中国药典 通则 0502）试验，吸取上述两种溶液各 3μl，分别点于同一硅胶 G 薄层板上，以三氯甲烷-甲醇-水（28:10:1）为展开剂，展开，取出，晾干，置氨蒸气中熏蒸 5 分钟后，置紫外光灯（365nm）下检视。供试品色谱中，在与对照品色谱相应的位置上，显相同颜色的荧光斑点。

【特征图谱】 照高效液相色谱法（中国药典 通则 0512）测定。

色谱条件与系统适用性试验 以十八烷基硅烷键合硅胶为填充剂；以乙腈为流动相 A，以 0.1%甲酸溶液为流动相 B，按下表梯度洗脱；检测波长为 250nm。理论板数按大豆苷峰计算应不低于 3000。

时间（分钟）	流动相 A（%）	流动相 B（%）
0	10	90
40	35	65
50	35	65

参照物溶液的制备 取粉葛对照药材 0.8g，加 30%乙醇 50ml，加热回流 30 分钟，放冷，滤过，取续滤液，作为对照药材参照物溶液。另取大豆苷对照品适量，精密称定，加 30%乙醇制成每 1ml 含 70μg 的溶液，作为对照品参照物溶液。

供试品溶液的制备　取本品适量，研细，取约 0.5g，精密称定，置具塞锥形瓶中，精密加入 30%乙醇 50ml，密塞，称定重量，回流提取 30 分钟，放冷，再称定重量，用 30%乙醇补足减失的重量，摇匀，滤过，取续滤液，即得。

测定法　精密吸取参照物溶液与供试品溶液各 10μl，注入液相色谱仪，测定，即得。

供试品色谱图中应呈现与对照药材参照物色谱中（除峰 5 外）5 个保留时间相对应的特征峰，峰 3 应与对照品参照物峰的保留时间相对应。峰 1~2、峰 4~6 与 S 峰（峰 3）的相对保留时间依次约为：0.77、0.82、1.35、1.62、1.94。

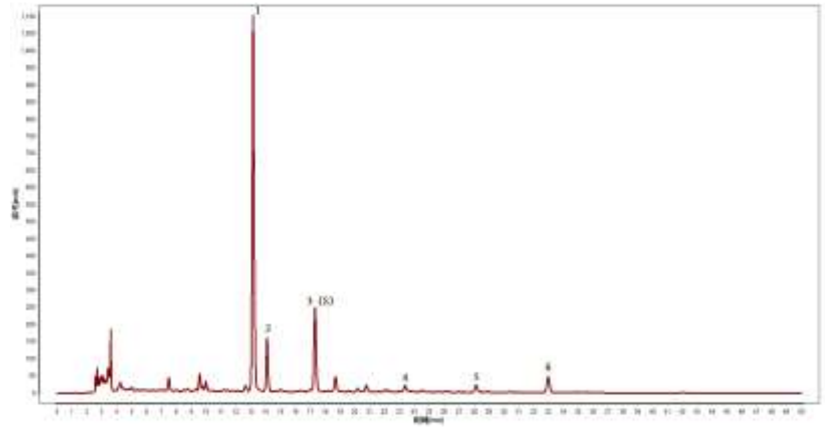

对照特征图谱

峰 1：葛根素　峰 3（S）：大豆苷　峰 6：大豆苷元

色谱柱：　5 TC-C18（250mm×4.6mm，5μm）

【检查】　应符合颗粒剂（中国药典 通则 0104）项下有关的各项规定。

【浸出物】　取本品适量，研细，照醇溶性浸出物测定法（中国药典 通则 2201）项下的热浸法测定，用乙醇作溶剂，不得少于 20.0%。

【含量测定】　照高效液相色谱法（中国药典 通则 0512）测定。

色谱条件与系统适用性试验　以十八烷基硅烷键合硅胶为填充剂；以甲醇-水（25: 75)为流动相；检测波长为 250nm。理论板数按葛根素峰计应不低于 4000。

对照品溶液的制备　取葛根素对照品适量，精密称定，加 30%乙醇制成每 lml 含 80μg 的溶液，即得。

供试品溶液的制备　同【特征图谱】项。

测定法　精密吸取对照品溶液与供试品溶液各 10μl，注入液相色谱仪，测定，即得。

本品每 1g 含葛根素($C_{21}H_{20}O_9$)应为 11~40mg。

【规格】　每 1g 配方颗粒相当于饮片 4g

【贮藏】　密封。

江苏省药品监督管理局

中药配方颗粒标准

JS-YBZ-2024410

盐韭菜子配方颗粒

Yanjiucaizi　Peifangkeli

【来源】 本品为百合科植物韭菜 *Allium tuberosum* Rottl.ex Spreng.的干燥成熟种子经炮制并按标准汤剂的主要质量指标加工制成的配方颗粒。

【制法】 取盐韭菜子饮片 7000g，加水煎煮，滤过，滤液浓缩成清膏（出膏率为 7~13%），加辅料适量，干燥（或干燥、粉碎），再加辅料适量，混匀，制粒，制成 1000g，即得。

【性状】 本品为浅黄色至黄棕色颗粒；气特异，味微辛。

【鉴别】 取本品 0.5g，研细，加水 20ml，微热使溶解，冷却，用乙酸乙酯振摇提取 2 次，每次 20ml，合并乙酸乙酯液，蒸干，残渣加 0.5ml 甲醇使溶解，作为供试品溶液。另取韭菜子对照药材 2g，加水 50ml，加热回流 30 分钟，滤过，滤液浓缩至约 20ml，自“用乙酸乙酯振摇提取”起，同法制成对照药材溶液。照薄层色谱法（中国药典 通则 0502）试验，吸取上述两种溶液各 12μl，分别点于同一硅胶 G 薄层板上，以甲苯-乙酸乙酯-甲酸（7∶2∶1）为展开剂，展开，取出，晾干，置紫外光灯（365nm) 下检视。供试品色谱中，在与对照药材色谱相应的位置上，显相同颜色的荧光主斑点。

【特征图谱】 照高效液相色谱法（中国药典 通则 0512）测定。

色谱条件与系统适用性试验 以十八烷基硅烷键合硅胶为填充剂；以甲醇为流动相 A，以水为流动相 B，按下表梯度洗脱；检测波长为 260nm；柱温为 30℃。理论板数按腺苷峰计应不低于 5000。

时间（分钟）	流动相 A(%)	流动相 B(%)
0	2	98
18	2	98
28	9	91
43	13.5	86.5
50	13.5	86.5

参照物溶液的制备 取韭菜子对照药材 1g，置具塞锥形瓶中，加 20%甲醇 20ml，超声处理（功率 600W，频率 40kHz）30 分钟，放冷，摇匀，滤过，取续

滤液，作为对照药材参照物溶液。另取鸟苷对照品适量，精密称定，加20%甲醇制成每1ml含40μg的溶液，作为对照品参照物溶液。再取尿苷、腺苷对照品适量，精密称定，加20%甲醇制成每1ml各含40μg的混合溶液，作为混合对照品参照物溶液。

供试品溶液的制备　取本品适量，研细，取约0.5g，精密称定，置具塞锥形瓶中，精密加入20%甲醇20ml，密塞，称定重量，超声处理（功率600W，频率40kHz）30分钟，取出，放冷，再称定重量，用20%甲醇补足减失的重量，摇匀，滤过，取续滤液，即得。

测定法　分别精密吸取参照物溶液与供试品溶液各10μl，注入液相色谱仪，测定，即得。

供试品色谱图中应呈现与对照药材参照物色谱图中7个保留时间相对应的特征峰，峰3、峰6～7应分别与相应对照品参照物峰的保留时间相对应。峰1～2、峰4与S1峰（峰3）的相对保留时间依次约为0.70、0.82、1.15；峰5与S2峰（峰6）的相对保留时间约为：0.75。

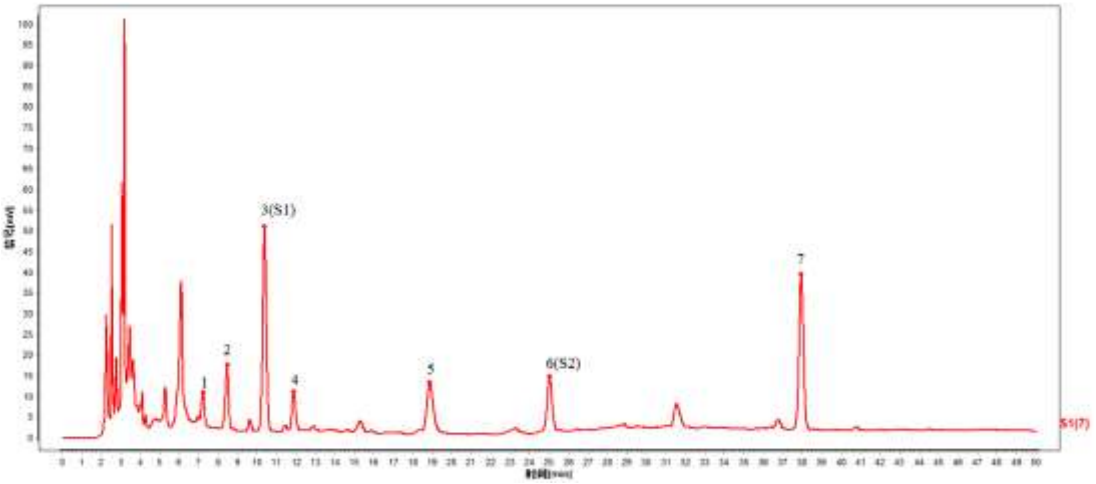

对照特征图谱

峰1：胞苷　峰2：鸟嘌呤　峰3(S1)：尿苷　峰6(S2)：鸟苷　峰7：腺苷

色谱柱：HSS T3（250mm×4.6mm，5μm）

【检查】 应符合颗粒剂（中国药典 通则0104）项下有关的各项规定。

【浸出物】 照醇溶性浸出物测定法（中国药典 通则2201）项下的热浸法测定，用乙醇作溶剂，不得少于18.0%。

【含量测定】 照高效液相色谱法（中国药典 通则0512）测定。

色谱条件与系统适用性试验 同【特征图谱】项。

对照品溶液的制备　同【特征图谱】项下的混合对照品参照物溶液。

供试品溶液的制备　同【特征图谱】项。

测定法　分别精密吸取对照品溶液与供试品溶液各 10μl，注入液相色谱仪，测定，即得。

本品每 1g 含尿苷（$C_9H_{12}N_2O_6$）和腺苷（$C_{10}H_{13}N_5O_4$）的总量应为 0.75~2.30mg。

【规格】 每 1g 配方颗粒相当于饮片 7g

【贮藏】 密封。

江苏省药品监督管理局

中药配方颗粒标准

JS-YBZ-2024411

余甘子配方颗粒

Yuganzi　Peifangkeli

【来源】 本品为大戟科植物余甘子 *Phyllanthus emblica* L. 的干燥成熟果实经炮制并按标准汤剂的主要质量指标加工制成的配方颗粒。

【制法】 取余甘子饮片 2500g，加水煎煮，滤过，滤液浓缩成清膏（出膏率为 21~40%），加辅料适量，干燥（或干燥，粉碎），再加入辅料适量，混匀，制粒，制成 1000g，即得。

【性状】 本品为棕黄色至黄棕色的颗粒；气微，味微苦。

【鉴别】 取本品 0.2g，研细，加乙醇 20ml，超声处理 20 分钟，滤过，滤液蒸干，残渣加乙醇 1ml 使溶解，作为供试品溶液。另取余甘子对照药材 0.5g，加水 25ml，加热回流 60 分钟，放冷，滤过，滤液浓缩至近干，残渣加乙醇 20ml，同法制成对照药材溶液。照薄层色谱法（中国药典 通则 0502）试验，吸取上述两种溶液各 3μl，分别点于同一硅胶 G 薄层板上，以甲苯-甲酸乙酯-甲酸（5∶5∶1）为展开剂，展开，取出，晾干，先喷以三氯化铝试液，热风加热 3 分钟，置紫外光灯（365nm）下检视；再喷以 5%三氯化铁乙醇溶液，置日光下检视。供试品色谱中，在与对照药材色谱相应的位置上，显相同颜色的荧光斑点或斑点。

【特征图谱】 照高效液相色谱法（中国药典 通则 0512）测定。

色谱条件与系统适用性试验 以十八烷基硅烷键合硅胶为填充剂；以乙腈为流动相 A，以 0.05%磷酸溶液为流动相 B，按下表梯度洗脱；流速为 0.20ml/min；检测波长为 235nm；柱温为 30℃。理论板数按没食子酸峰计应不低于 2000。

时间（分钟）	流动相 A（%）	流动相 B（%）
0	0	100
3	0	100
9	3	97
12	10	90
15	10	90
19	15	85
26	20	80
35	20	80

参照物溶液的制备 取余甘子对照药材0.5g，加入水25ml，加热回流2小时，放冷，滤过，取续滤液，作为对照药材参照物溶液。另取没食子酸对照品适量，精密称定，加50%甲醇制成每1ml含0.15mg的溶液，作为对照品参照物溶液I。再取柯里拉京对照品适量，精密称定，加30%甲醇制成每1ml含20μg的溶液，作为对照品参照物溶液II。

供试品溶液的制备 取本品适量，研细，取0.2g，加30%甲醇25ml，密塞，超声处理30分钟，放冷，滤过，取续滤液，即得。

测定法 精密吸取参照物溶液与供试品溶液各1μl，注入液相色谱仪，测定，即得。

供试品色谱图中应呈现与对照药材参照物色谱图中 8 个保留时间相对应的特征峰，峰2、峰7应分别与相应对照品参照物峰的保留时间相对应。峰1与S1峰（峰2）的相对保留时间约为：0.76；峰3~6、峰8与S2峰（峰7）的相对保留时间依次约为：0.48、0.59、0.88、0.95、1.24。

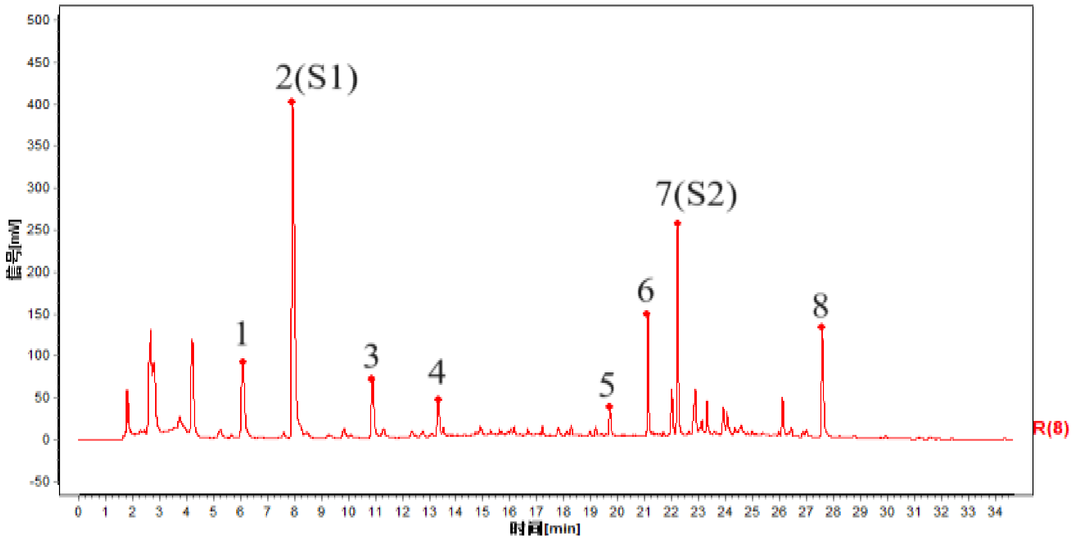

对照特征图谱

峰2(S1)：没食子酸　峰7 (S2)：柯里拉京　峰8：鞣花酸

色谱柱：　CORTECS T3（150mm×2.1mm，1.6μm）

【检查】 应符合颗粒剂（中国药典 通则0104）项下有关的各项规定。

【浸出物】 照醇溶性浸出物测定法（中国药典 通则2201）项下的热浸法测定，用乙醇作溶剂，不得少于40.0%。

【含量测定】 照高效液相色谱法（中国药典 通则0512）测定。

色谱条件与系统适用性试验 以十八烷基硅烷键合硅胶为填充剂；以甲醇-0.2%磷酸溶液（5∶95）为流动相；检测波长为273nm。理论板数按没食子酸峰

计应不低于2000。

对照品溶液的制备 同【特征图谱】项下的对照品参照物溶液I。

供试品溶液的制备 取本品适量，研细，取约0.1g，精密称定，置具塞锥形瓶中，精密加入50%甲醇50ml，密塞，称定重量，超声处理（功率250W，频率40kHz）30分钟，放冷，再称定重量，用50%甲醇补足减失的重量，摇匀，滤过，取续滤液，即得。

测定法 精密吸取对照品溶液与供试品溶液各5μl，注入液相色谱仪，测定，即得。

本品每1g含没食子酸（$C_{21}H_{20}O_{11}$）应为30~100mg。

【规格】 每1g配方颗粒相当于饮片2.5g

【贮藏】 密封。

江苏省药品监督管理局

中药配方颗粒标准

JS-YBZ-2024412

浙桐皮（樗叶花椒）配方颗粒

Zhetongpi（Chuyehuajiao） Peifangkeli

【来源】 本品为芸香科植物樗叶花椒 *Zanthoxylum ailanthoides* Sieb. et Zucc. 的干燥树皮经炮制并按标准汤剂的主要质量指标加工制成的配方颗粒。

【制法】 取浙桐皮饮片 8000g，加水煎煮，滤过，滤液浓缩成清膏（出膏率为 7~12%），加辅料适量，干燥（或干燥，粉碎），再加入辅料适量，混匀，制粒，制成 1000g，即得。

【性状】 本品为灰黄色至棕色的颗粒；气微，味苦。

【鉴别】 取本品 0.2g，研细，加甲醇 20ml，超声处理 30 分钟，滤过，滤液蒸干，残渣加甲醇 1ml 使溶解，作为供试品溶液。另取浙桐皮对照药材 0.5g，加水 50ml，煎煮 30 分钟，滤过，滤液蒸干，残渣加甲醇 20ml，同法制成对照药材溶液。再取木兰花碱对照品，加甲醇制成每 1ml 含 1mg 的溶液，作为对照品溶液。照薄层色谱法（中国药典 通则 0502）试验，吸取上述三种溶液各 2μl，分别点于同一硅胶 G 薄层板上，以乙酸乙酯-丙酮-甲酸-水（7∶2∶2∶1）为展开剂，展开，取出，晾干，置紫外光灯（254nm）下检视。供试品色谱中，在与对照药材色谱和对照品色谱相应的位置上，显相同颜色的荧光斑点。

【特征图谱】 照高效液相色谱法（中国药典 通则 0512）测定。

色谱条件与系统适用性试验 以十八烷基硅烷键合硅胶为填充剂；以乙腈为流动相 A，以 0.1%磷酸溶液为流动相 B，按下表梯度洗脱；流速为 0.35ml/min；检测波长为 230nm；柱温为 35℃。理论板数按橙皮苷峰计应不低于 3000。

时间（分钟）	流动相 A（%）	流动相 B（%）
0	6	94
8	8	92
12	10	90
20	17	83
23	17	83
27	20	80
28	60	40

参照物溶液的制备 取浙桐皮对照药材 0.3g，加 30%甲醇 25ml，加热回流 30 分钟，放冷，滤过，取续滤液，作为对照药材参照物溶液。另取橙皮苷对照品适量，精密称定，加甲醇制成每 1ml 含 0.12mg 的溶液，作为对照品参照物溶液。再取木兰花碱、新绿原酸、绿原酸、隐绿原酸对照品适量，精密称定，加甲醇分别制成每 1ml 各含 50μg 的溶液，作为混合对照品参照物溶液。

供试品溶液的制备 取本品适量，研细，取约 0.2g，精密称定，置具塞锥形瓶中，精密加入甲醇 25ml，密塞，称定重量，超声处理（功率 250W，频率 40kHz）30 分钟，放冷，再称定重量，用甲醇补足减失的重量，摇匀，滤过，取续滤液，即得。

测定法 精密吸取参照物溶液与供试品溶液各 0.5μl，注入液相色谱仪，测定，即得。

供试品色谱中应呈现与对照药材参照物色谱图中的 8 个保留时间相对应的特征峰，其中峰 1~3、峰 5、峰 8 应分别与相应的对照品参照物峰的保留时间相对应。峰 4、峰 6~7 与 S 峰（峰 5）的相对保留时间依次约为：0.79、1.56、1.60。

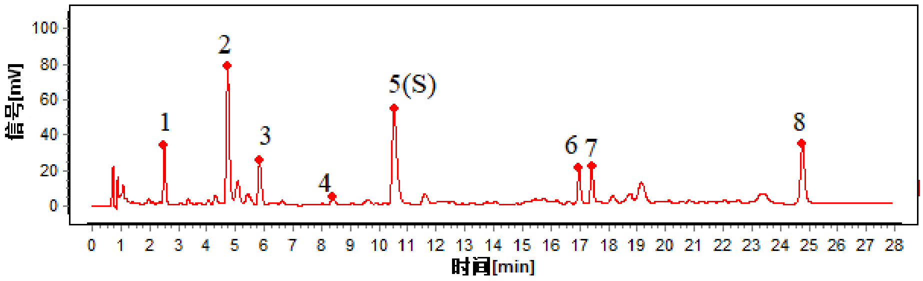

对照特征图谱

峰 1：新绿原酸 峰 2：绿原酸 峰 3：隐绿原酸 峰 5（S）：木兰花碱 峰 8：橙皮苷

色谱柱：BEH C18（100mm×2.1mm，1.7μm）

【检查】 应符合颗粒剂（中国药典 通则 0104）项下有关的各项规定。

【浸出物】 照醇溶性浸出物测定法（中国药典 通则 2201）项下的热浸法测定，不得少于 26.0%。

【含量测定】 照高效液相色谱法（中国药典 通则 0512）测定。

色谱条件与系统适用性试验　同【特征图谱】项。

对照品溶液的制备 同【特征图谱】项下的对照品参照物溶液。

供试品溶液的制备 同【特征图谱】项。

测定法 精密吸取对照品溶液与供试品溶液各 0.5μl，注入液相色谱仪，测定，即得。

本品每 1g 含橙皮苷($C_{28}H_{34}O_{15}$)应为 7.5~20.0mg。

【规格】 每 1g 配方颗粒相当于饮片 8g

【贮藏】 密封。

江苏省药品监督管理局

中药配方颗粒标准

JS-YBZ-2024413

竹叶柴胡配方颗粒

Zhuyechaihu Peifangkeli

【来源】 本品为伞形科植物竹叶柴胡 *Bupleurum marginatum* Wall.*ex* DC.的干燥全草经炮制并按标准汤剂的主要质量指标加工制成的配方颗粒。

【制法】 取竹叶柴胡饮片 3500g，加水煎煮，滤过，滤液浓缩成清膏（出膏率为 15-23%），加入辅料适量，干燥（或干燥，粉碎），再加入辅料适量，混匀，制粒，制成 1000g，即得。

【性状】 本品为棕黄色至黄棕色颗粒，气微，味微苦。

【鉴别】 取本品 2g，研细，加乙醇 20ml，超声处理 30 分钟，滤过，滤液浓缩至 1ml，作为供试品溶液。另取竹叶柴胡对照药材 2g，加水 50ml，煮沸 30 分钟，滤过，滤液蒸干，残渣加乙醇 20ml，同法制成对照药材溶液。照薄层色谱法（中国药典 通则 0502）试验，吸取上述两种溶液各 10μl，分别点于同一硅胶 G 薄层板上，以二氯甲烷-无水乙醇（40:1）为展开剂，展开，取出，晾干，喷以 10%硫酸乙醇溶液，加热至斑点显色清晰，置紫外光灯（365nm）下检视。供试品色谱中，在与对照药材色谱相应的位置上，显相同颜色的荧光斑点。

【特征图谱】 照高效液相色谱法（中国药典 通则 0512）测定。

色谱条件与系统适用性试验 以十八烷基硅烷键合硅胶为填充剂；以甲醇为流动相 A，以 0.4%磷酸溶液为流动相 B，按下表梯度洗脱；检测波长为 266nm；柱温为 30℃。理论板数按芦丁峰计应不低于 3000。

时间（分钟）	流动相 A（%）	流动相 B（%）
0	0	100
10	15	85
40	45	55
53	60	40
70	77.5	22.5

参照物溶液的制备 取竹叶柴胡对照药材 1g，加水 50ml，加热回流 30 分

钟，滤过，蒸干，残渣加 70%甲醇溶液 50ml，密塞，超声处理 30 分钟，放冷，滤过，取续滤液，作为对照药材参照物溶液。另取芦丁对照品适量，精密称定，加 70%甲醇制成每 1ml 含 80μg 的溶液，作为对照品参照物溶液。

供试品溶液的制备 取本品适量，研细，取约 0.2g，精密称定，精密加入 70%甲醇 50ml，密塞，称定重量，超声处理（功率 600W，频率 40kHz）30 分钟，放冷，再称定重量，用 70%甲醇补足减失的重量，摇匀，滤过，取续滤液，即得。

测定法 精密吸取参照物溶液与供试品溶液各 5μl，注入液相色谱仪，测定，即得。

供试品色谱中应呈现与对照药材参照物色谱中 7 个保留时间相对应的特征峰，峰 6 应与对照品参照物峰的保留时间相对应。峰 1~5、峰 7 与 S 峰（峰 6）的相对保留时间依次约为：0.39、0.42、0.57、0.61、0.62、1.23。

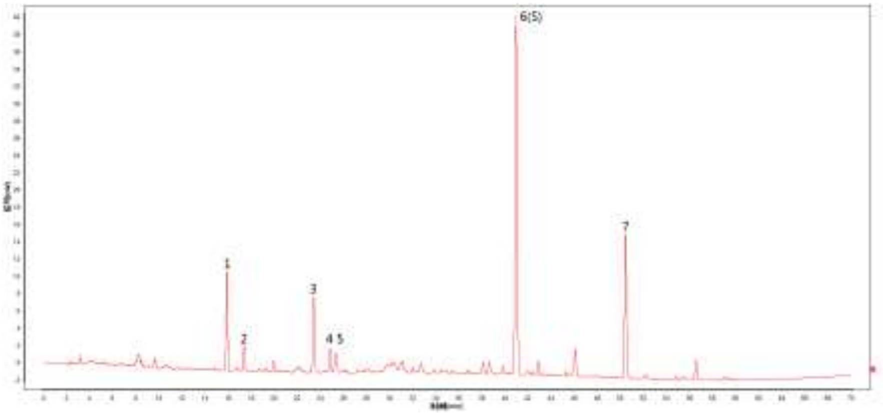

对照特征图谱

峰 2：新绿原酸　峰 3：绿原酸　峰 4：隐绿原酸　峰 6（S）：芦丁　峰 7：槲皮素

色谱柱 Kromasil 100-5-C18（250mm×4.6mm，5μm）

【检查】 应符合颗粒剂（中国药典 通则 0104）项下有关的各项规定。

【浸出物】 照醇溶性浸出物测定法（中国药典 通则 2201）项下的热浸法测定，用乙醇作溶剂，不得少于 18.0%。

【含量测定】 照高效液相色谱法（中国药典 通则 0512）测定。

色谱条件与系统适用性试验 以十八烷基硅烷键合硅胶为填充剂；以甲醇-0.4%磷酸溶液（38：62）为流动相；检测波长为 355nm；柱温 30℃。理论板数按芦丁峰计应不低于 3000。

对照品溶液的制备 同【特征图谱】项下的对照品参照物溶液。

供试品溶液的制备 同【特征图谱】项。

测定法 精密吸取对照品溶液与供试品溶液各 10μl，注入液相色谱仪，测定，

即得。

本品每1g含芦丁（$C_{27}H_{30}O_{16}$）应为8.0~30.0mg。

【规格】 每1g配方颗粒相当于饮片3.5g

【贮藏】 密封。
