# Supplementary material for: Efficacy of Xiaoyao-san preparations in treating Hashimoto’s thyroiditis: a meta-analysis and systematic review
Source: Front Pharmacol. 2025 Jun 13;16:1528506. doi: 10.3389/fphar.2025.1528506 (PMC12202410; doi:10.3389/fphar.2025.1528506)
Supplement: Supplementary file 2 [file Supplementaryfile2.zip › Supplementary Files 2/Supplementary Files 2 formula granules section/Mudanpi_BJ-PFKL-2022067.pdf]

北京市药品监督管理局
北京市中药配方颗粒标准

BJ-PFKL-2022067

牡丹皮配方颗粒

Mudanpi Peifangkeli

【来源】 本品为毛茛科植物牡丹*Paeonia suffruticosa* Andr.的干燥根皮经炮制并按标准汤剂的主要质量指标加工制成的配方颗粒。

【制法】 取牡丹皮饮片3000g，水蒸气蒸馏，收集结晶物（以β-环糊精适量包合，备用），蒸馏后的药渣加水煎煮，滤过，滤液浓缩成清膏（干浸膏出膏率为21%~30%），加入辅料适量及结晶β-环糊精包合物，干燥（或干燥，粉碎），再加入辅料适量，混匀，制粒，制成1000g，即得。

【性状】 本品为棕色至深棕色的颗粒；气芳香，味微苦而涩。

【鉴别】 取本品1g，研细，加水25ml，超声处理30分钟，滤过，滤液用乙酸乙酯振摇提取2次，每次25ml，合并乙酸乙酯液，挥干，残渣加丙酮1ml使溶解，作为供试品溶液。另取牡丹皮对照药材1g，加水30ml，煎煮30分钟，取出，放冷，滤过，滤液自“用乙酸乙酯振摇提取2次”起，同法制成对照药材溶液。再取丹皮酚对照品，加丙酮制成每1ml含2mg的溶液，作为对照品溶液。照薄层色谱法（中国药典2020年版通则0502）试验，吸取上述三种溶液各10μl，分别点于同一硅胶G薄层板上，以环己烷-乙酸乙酯-甲酸（3:1.5:0.2）为展开剂，展开，取出，晾干，喷以5%香草醛硫酸乙醇溶液（1→10），在105℃加热至斑点显色清晰。供试品色谱中，在与对照药材色谱和对照品色谱相应的位置上，显相同颜色的斑点。

【特征图谱】 照高效液相色谱法（中国药典2020年版通则0512）测定。

色谱条件与系统适用性试验 以十八烷基硅烷键合硅胶为填充剂（柱长为100mm，内径为2.1mm，粒径为1.7μm）；以乙腈为流动相A，以0.1%甲酸溶液为流动相B，按下表中的规定进行梯度洗脱；流速为每分钟0.6ml；柱温为35℃；检测波长为254nm。理论板数按丹皮酚峰计算应不低于5000。

时间（分钟）	流动相A（%）	流动相B（%）
0~4	0→12	100→88
4~6	12	88
6~8	12→21	88→79
8~12	21→40	79→60

12~13	40→100	60→0
13~15	100→0	0→100

参照物溶液的制备 取牡丹皮对照药材1g，置圆底烧瓶中，加水50ml，煎煮30分钟，放冷，滤过，取续滤液，作为对照药材参照物溶液。另取没食子酸对照品、芍药苷对照品适量，精密称定，分别加甲醇制成每1ml各含20μg的溶液，作为没食子酸对照品、芍药苷对照品参照物溶液；再取〔含量测定〕项下的对照品溶液，作为丹皮酚对照品参照物溶液。

供试品溶液的制备 同〔含量测定〕项。

测定法 分别精密吸取参照物溶液与供试品溶液各2μl，注入超高效液相色谱仪，测定，即得。

供试品色谱中应呈现9个特征峰，并应与对照药材参照物色谱中的9个特征峰保留时间相对应，其中峰1、峰4、峰9应分别与没食子酸、芍药苷、丹皮酚对照品参照物峰保留时间相对应。与芍药苷参照物峰相应的峰为S1峰，计算峰2、峰3与S1峰的相对保留时间，其相对保留时间应在规定值的±15%范围之内；与丹皮酚参照物峰相应的峰为S2峰，计算峰5~峰8与S2峰的相对保留时间，其相对保留时间应在规定值的±10%范围之内。规定值为：0.56（峰2）、0.71（峰3）、0.64（峰5）、0.78（峰6）、0.90（峰7）、0.93（峰8）。

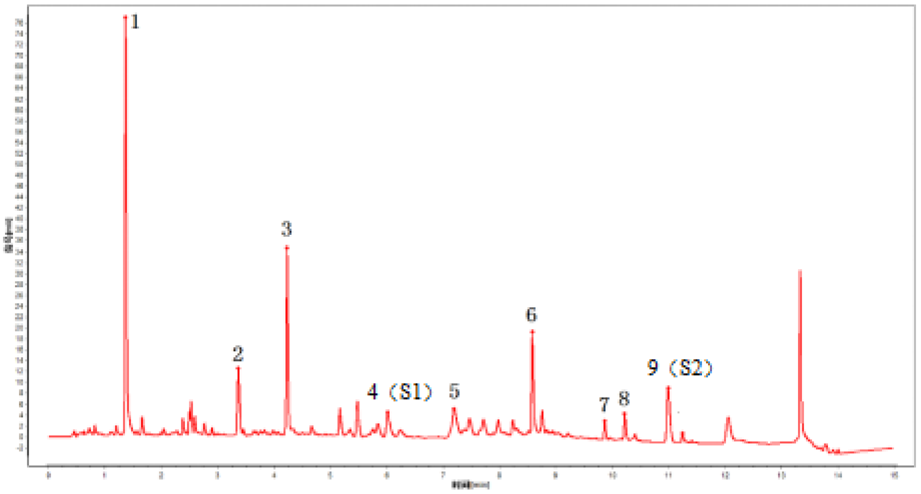

对照特征图谱

峰 1：没食子酸　峰 4（S1）：芍药苷　峰 7：牡丹皮苷 C　峰 9（S2）：丹皮酚

色谱柱：ACQUITY BEH C18，2.1mm×100mm，1.7μm

【检查】 应符合颗粒剂项下有关的各项规定（中国药典2020年版通则0104）。

【浸出物】 照醇溶性浸出物测定法（中国药典2020年版通则2201）项下的热浸法测定，用乙醇作溶剂，不得少于33.0%。

【含量测定】 照高效液相色谱法（中国药典2020年版通则0512）测定。

色谱条件与系统适用性试验 以十八烷基硅烷键合硅胶为填充剂；以甲醇-水（45:55）为流动相；流速为每分钟0.4ml；检测波长为274nm。理论板数按丹皮酚峰计算应不低于5000。

对照品溶液的制备 取丹皮酚对照品适量，精密称定，加甲醇制成每1ml含20μg的溶液，

即得。

供试品溶液的制备 取本品适量，研细，取约0.1g，精密称定，置具塞锥形瓶中，精密加入甲醇20ml，密塞，称定重量，超声处理（功率250W，频率40kHz）20分钟，取出，放冷，再称定重量，用甲醇补足减失的重量，摇匀，滤过，取续滤液，即得。

测定法 分别精密吸取对照品溶液与供试品溶液各2μl，注入超高效液相色谱仪，测定，即得。

本品每1g含丹皮酚（$C_9H_{10}O_3$）应为2.4mg~6.6mg。

【规格】 每1g配方颗粒相当于饮片3.0g

【贮藏】 密封。
