# Supplementary material for: Efficacy of Xiaoyao-san preparations in treating Hashimoto’s thyroiditis: a meta-analysis and systematic review
Source: Front Pharmacol. 2025 Jun 13;16:1528506. doi: 10.3389/fphar.2025.1528506 (PMC12202410; doi:10.3389/fphar.2025.1528506)
Supplement: Supplementary file 2 [file Supplementaryfile2.zip › Supplementary Files 2/Supplementary Files 2 formula granules section/Shanyao_JS-YBZ-2021095 .pdf]

江苏省药品监督管理局
中药配方颗粒标准

JS-YBZ-2021095

山药配方颗粒
Shanyao Peifangkeli

【来源】 本品为薯蓣科植物薯蓣 *Dioscorea opposita* Thunb. 的干燥根茎经炮制并按标准汤剂的主要质量指标加工制成的配方颗粒。

【制法】 取山药饮片 4000g，加水煎煮，滤过，滤液浓缩成清膏（出膏率为 15～25%），加入辅料适量，干燥（或干燥，粉碎），再加入辅料适量，混匀，制粒，制成 1000g，即得。

【性状】 本品为类白色至黄色的颗粒；气微，味淡、微酸。

【鉴别】 取本品 1g，研细，加乙醇 20ml，超声处理 20 分钟，滤过，滤液浓缩至 2ml，作为供试品溶液。另取山药对照药材 2g，同法制成对照药材溶液。照薄层色谱法（中国药典 通则 0502）试验，吸取供试品溶液 2µl、对照药材溶液 5µl，分别点于同一硅胶 G 薄层板上，以水饱和正丁醇-冰醋酸（13：3）为展开剂，展开，取出，晾干，喷以茚三酮试液，在 105℃加热至斑点显色清晰。供试品色谱中，在与对照药材色谱相应的位置上，显相同颜色的斑点。

【特征图谱】 照高效液相色谱法（中国药典 通则 0512）测定。

色谱条件与系统适用性试验 以十八烷基硅烷键合硅胶为填充剂；以乙腈为流动相 A，以 0.2%磷酸溶液为流动相 B，按下表梯度洗脱；流速为 0.2ml/min；柱温为 30℃；检测波长为 258nm。理论板数按腺苷峰计算应不低于 4000。

时间（分钟）	流动相 A（%）	流动相 B（%）
0	1	99
4	1	99
10	3	97
25	20	80
27	1	99
35	1	99

参照物溶液的制备 取山药对照药材约 0.5g，加 10%甲醇 25ml，超声处理（功率 300W，频率 40kHz）30 分钟，取出，放冷，滤过，取续滤液，作为对照药材参照物溶液。取腺苷对照品适量，精密称定，加 10%甲醇制成每 1ml 含 5µg

的溶液，作为对照品参照物溶液。

供试品溶液的制备 取本品适量，研细，取约 0.3g，精密称定，置具塞锥形瓶中，精密加入 10%甲醇 25ml，称定重量，超声处理（功率 300W，频率 40kHz）30 分钟，取出，放冷，再称定重量，用 10%甲醇补足减失的重量，摇匀，滤过，取续滤液，即得。

测定法 分别精密吸取参照物溶液与供试品溶液各 1μl，注入液相色谱仪，测定，即得。

供试品色谱图中应呈现与对照药材参照物色谱中 4 个保留时间相对应的特征峰，峰 1 应与对照品参照物峰的保留时间相对应。峰 2～4 与 S 峰（峰 1）的相对保留时间依次约为：1.61、3.82、4.26。

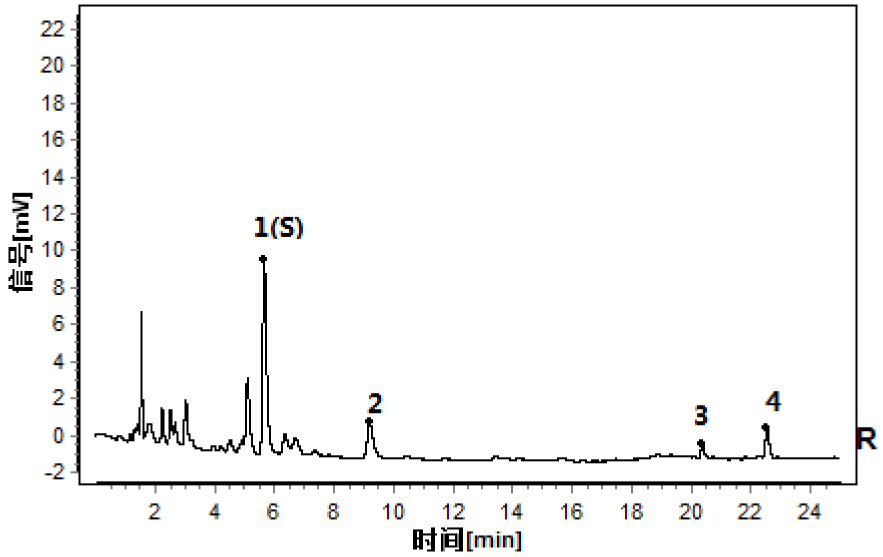

对照特征图谱

峰 1（S）：腺苷

色谱柱： Triart C18 （100mm×2.1mm，1.9μm）

【检查】 溶化性 照颗粒剂溶化性检查方法（中国药典 通则 0104）检查，加热水 200ml，搅拌 5 分钟（必要时加热煮沸 5 分钟），立即观察，应全部溶化或轻微浑浊，不得有焦屑。

其他 应符合颗粒剂项下有关的各项规定（中国药典 通则 0104）。

应符合颗粒剂项下有关的各项规定（中国药典 通则 0104）。

【浸出物】 取本品研细，取约 2g，精密称定，精密加入乙醇 100ml，照醇溶性浸出物测定法（中国药典 通则 2201）项下的热浸法测定，不得少于 7.0%。

【含量测定】 腺苷 照高效液相色谱法（中国药典 通则 0512）测定。

色谱条件与系统适用性试验 同【特征图谱】项。

对照品溶液的制备 同【特征图谱】项下的对照品参照物溶液。

供试品溶液的制备 同【特征图谱】项。

测定法 分别精密吸取对照品溶液与供试品溶液各 1μl，注入液相色谱仪，测定，即得。

本品每 1g 含腺苷（$C_{10}H_{13}N_5O_4$）应为 0.30~1.50mg。

尿囊素 照高效液相色谱法（中国药典 通则 0512）测定。

色谱条件与系统适用性试验 以氨基键合硅胶为填充剂；以乙腈-水（90：10）为流动相；检测波长为 224nm。理论板数按尿囊素峰计算应不低于 2500。

对照品溶液的制备 取尿囊素对照品适量，精密称定，加甲醇制成每 1ml 含 80μg 的溶液，作为对照品溶液。

供试品溶液的制备 取本品适量，研细，取约 0.3g，精密称定，置具塞锥形瓶中，精密加入稀乙醇 25ml，称定重量，超声处理（功率 300W，频率 40kHz）30 分钟，取出，放冷，再称定重量，用稀乙醇补足减失的重量，摇匀，滤过，取续滤液，即得。

测定法 分别精密吸取对照品溶液与供试品溶液各 10μl，注入液相色谱仪，测定，即得。

本品每 1g 含尿囊素（$C_4H_6N_4O_3$）应为 7.0~42.0mg。

【规格】 每 1g 配方颗粒相当于饮片 4g

【贮藏】 密封。
