# Supplementary material for: Efficacy of Xiaoyao-san preparations in treating Hashimoto’s thyroiditis: a meta-analysis and systematic review
Source: Front Pharmacol. 2025 Jun 13;16:1528506. doi: 10.3389/fphar.2025.1528506 (PMC12202410; doi:10.3389/fphar.2025.1528506)
Supplement: Supplementary file 2 [file Supplementaryfile2.zip › Supplementary Files 2/Supplementary Files 2 formula granules section/Shanzhuyu_YBZ-PFKL-2021110 .pdf]

国家药品监督管理局
国家药品标准

YBZ-PFKL-2021110

山萸肉配方颗粒

Shanyurou Peifangkeli

【来源】 本品为山茱萸科植物山茱萸 *Cornus officinalis* Sieb.et Zucc.的干燥成熟果肉经炮制并按标准汤剂的主要质量指标加工制成的配方颗粒。

【制法】 取山萸肉饮片 1200g，加水煎煮，滤过，滤液浓缩成清膏（干浸膏出膏率为 42%～60%），加入辅料适量，干燥（或干燥，粉碎），再加入辅料适量，混匀，制粒，制成 1000g，即得。

【性状】 本品为浅棕色至红棕色的颗粒；气微，味酸、涩、微苦。

【鉴别】 取本品 1g，研细，加无水乙醇 10ml，超声处理 15 分钟，滤过，滤液蒸干，残渣加无水乙醇 1ml 使溶解，作为供试品溶液。另取山茱萸对照药材 1.5g，加水 50ml，煮沸 30 分钟，滤过，滤液蒸干，残渣加无水乙醇 10ml，同法制成对照药材溶液。再取马钱苷对照品，加无水乙醇制成每 1ml 含 1mg 的溶液，作为对照品溶液。照薄层色谱法（中国药典 2020 年版通则 0502）试验，吸取上述三种溶液各 5μl，分别点于同一硅胶 G 薄层板上，以乙酸乙酯-乙醇-冰醋酸（50∶10∶1）为展开剂，展开，取出，晾干，喷以 5%香草醛硫酸溶液，在 105℃加热至斑点显色清晰。供试品色谱中，在与对照品色谱相应的位置上，显相同的紫红色斑点，与对照药材色谱相应的位置上，显相同颜色的斑点。

【特征图谱】 照高效液相色谱法（中国药典 2020 年版通则 0512）测定。

色谱条件与系统适用性试验 以十八烷基硅烷键合硅胶为填充剂（柱长为 150mm，内径为 2.1mm，粒径为 1.7μm）；以乙腈为流动相 A，以 0.2%磷酸溶液为流动相 B，按下表中的规定进行梯度洗脱；流速为每分钟 0.2ml；柱温为 25℃；检测波长为 260nm。理论板数按马钱苷峰计算应不低于 10000。

时间（分钟）	流动相 A（%）	流动相 B（%）
0~3	2→8	98→92
3~9	8→15	92→85
9~13	15→23	85→77
13~19	23→100	77→0
19.1~24	2	98

参照物溶液的制备 取山茱萸对照药材 0.2g，置具塞锥形瓶中，加水 10ml，加热回流 60 分钟，放冷，离心，取上清液，加甲醇定容至 20ml 量瓶中，摇匀，滤过，取续滤液，作为对照药材参照物溶液。另取没食子酸对照品、5-羟甲基糠醛对照品、莫诺苷对照品、马钱苷对照品适量，加 80%甲醇

制成每 1ml 含没食子酸 20μg、5-羟甲基糠醛 20μg、莫诺苷 80μg、马钱苷 100μg 的混合溶液，作为对照品参照物溶液。

供试品溶液的制备 取本品适量，研细，取约 0.1g，置具塞锥形瓶中，加 80%甲醇 20ml，超声处理（功率 500W，频率 40kHz）30 分钟，放冷，摇匀，滤过，取续滤液，即得。

测定法 分别精密吸取参照物溶液与供试品溶液各 1μl，注入液相色谱仪，测定，即得。

供试品色谱中应呈现 5 个特征峰，并应与对照药材参照物色谱中的 5 个特征峰保留时间相对应，其中 4 个峰应分别与相应对照品参照物峰的保留时间相对应。与马钱苷参照物峰相对应的峰为 S 峰，计算峰 5 与 S 峰的相对保留时间，其相对保留时间应在规定值的±10%范围之内，规定值为：1.37（峰 5）；计算峰 1 与 S 峰的相对峰面积，其相对峰面积应在规定范围内，规定范围为：0.35~3.00（峰 1）。

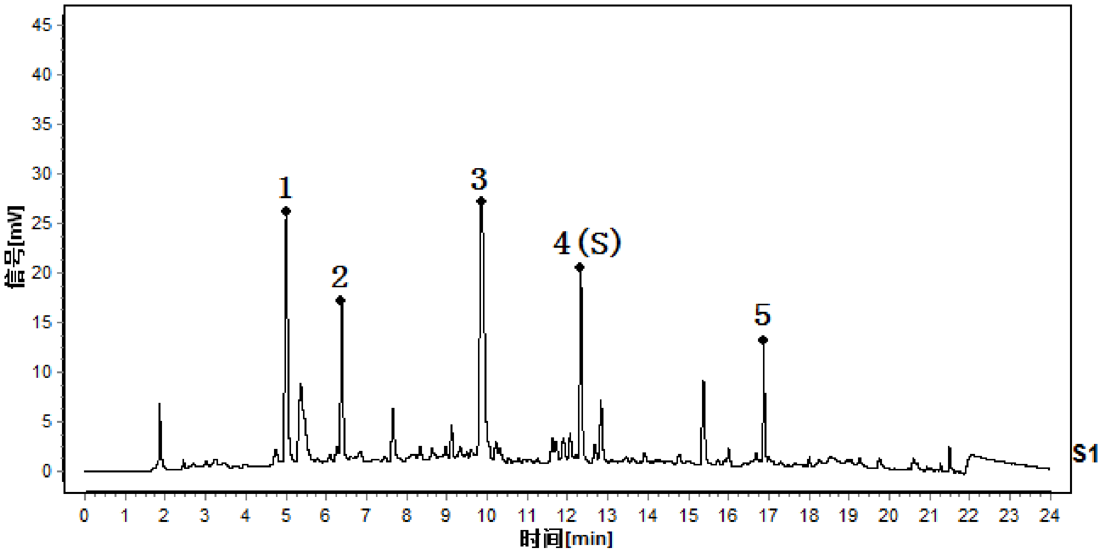

对照特征图谱

峰 1：没食子酸；峰 2：5-羟甲基糠醛；峰 3：莫诺苷；峰 4（S）：马钱苷

色谱柱：BEH C18，2.1mm×150mm，1.7μm

【检查】 **重金属及有害元素** 照铅、镉、砷、汞、铜测定法（中国药典 2020 年版通则 2321）测定，铅不得过 5mg/kg；镉不得过 1mg/kg；砷不得过 2mg/kg；汞不得过 0.2mg/kg；铜不得过 20mg/kg。

其他 应符合颗粒剂项下有关的各项规定（中国药典 2020 年版通则 0104）。

【浸出物】 取本品研细，取约 2g，精密称定，精密加入乙醇 100ml，照醇溶性浸出物测定法（中国药典 2020 年版通则 2201）项下的热浸法测定，不得少于 30.0%。

【含量测定】 照高效液相色谱法（中国药典 2020 年版通则 0512）测定。

色谱条件与系统适用性试验 以十八烷基硅烷键合硅胶为填充剂；以乙腈为流动相 A，以 0.3%磷酸溶液为流动相 B，按下表中的规定进行梯度洗脱；流速为每分钟 0.3ml，柱温为 35℃；检测波长为 240nm。理论板数按马钱苷峰计算应不低于 10000。

时间（分钟）	流动相 A（%）	流动相 B（%）
0~4	7	93
4~13.5	7→20	93→80

13.5~13.6	20→7	80→93
13.6~19	7	93

对照品溶液的制备 取莫诺苷对照品、马钱苷对照品适量，精密称定，加80%甲醇制成每1ml各含50μg的混合溶液，即得。

供试品溶液的制备 取本品适量，研细，取约0.15g，精密称定，置具塞锥形瓶中，精密加入甲醇20ml，称定重量，超声处理（功率500W，频率40kHz）30分钟，放冷，再称定重量，用甲醇补足减失的重量，摇匀，滤过，取续滤液，即得。

测定法 分别精密吸取对照品溶液与供试品溶液各1μl，注入液相色谱仪，测定，即得。

本品每1g含莫诺苷（$C_{17}H_{26}O_{11}$）和马钱苷（$C_{17}H_{26}O_{10}$）的总量应为16.0mg~31.0mg。

【规格】 每1g配方颗粒相当于饮片1.2g

【贮藏】 密封。
