# Supplementary material for: Efficacy of Xiaoyao-san preparations in treating Hashimoto’s thyroiditis: a meta-analysis and systematic review
Source: Front Pharmacol. 2025 Jun 13;16:1528506. doi: 10.3389/fphar.2025.1528506 (PMC12202410; doi:10.3389/fphar.2025.1528506)
Supplement: Supplementary file 2 [file Supplementaryfile2.zip › Supplementary Files 2/Supplementary Files 2 formula granules section/Shaoyao_YBZ-PFKL-2021002.pdf]

国家药品监督管理局
国家药品标准

YBZ-PFKL-2021002

白芍配方颗粒

Baishao Peifangkeli

【来源】 本品为毛茛科植物芍药 *Paeonia lactiflora* Pall.的干燥根经炮制并按标准汤剂的主要质量指标加工制成的配方颗粒。

【制法】 取白芍饮片 4500g，加水煎煮，滤过，滤液浓缩成清膏（干浸膏出膏率为 14%～22%），加入辅料适量，干燥（或干燥，粉碎），再加入辅料适量，混匀，制粒，制成 1000g，即得。

【性状】 本品为黄白色至黄棕色的颗粒；气微，味苦、微酸。

【鉴别】 取本品 0.5g，研细，加乙醇 20ml，超声处理 5 分钟，滤过，滤液浓缩至约 1ml，作为供试品溶液。另取白芍对照药材 2g，同法制成对照药材溶液。再取芍药苷对照品，加乙醇制成每 1ml 含 1mg 的溶液，作为对照品溶液。照薄层色谱法（中国药典 2020 年版通则 0502）试验，吸取供试品溶液与对照药材溶液各 2μl、对照品溶液 5μl，分别点于同一硅胶 G 薄层板上，以三氯甲烷-乙酸乙酯-甲醇-甲酸（40∶5∶10∶0.2）为展开剂，展开，取出，晾干，喷以 5%香草醛硫酸溶液，加热至斑点显色清晰。供试品色谱中，在与对照药材和对照品色谱相应的位置上，显相同颜色的斑点。

【特征图谱】 照高效液相色谱法（中国药典 2020 年版通则 0512）测定。

色谱条件与系统适用性试验 以十八烷基硅烷键合硅胶为填充剂（柱长为 250mm，内径为 4.6mm，粒径为 5μm）；以乙腈为流动相 A，以 0.1%磷酸溶液为流动相 B，按下表中的规定进行梯度洗脱；柱温为 30℃；检测波长为 230nm。理论板数按芍药苷峰计算应不低于 2000。

时间（分钟）	流动相 A（%）	流动相 B（%）
0~25	5→15	95→85
25~37	15	85
37~38	15→20	85→80
38~58	20	80
58~70	20→50	80→50
70~71	50→5	50→95
71~85	5	95

参照物溶液的制备 取白芍对照药材 0.4g，置具塞锥形瓶中，加稀乙醇 50ml，超声处理（功率 250W，频率 40kHz）30 分钟，放冷，摇匀，滤过，取续滤液，作为对照药材参照物溶液。另取没食子酸对照品、儿茶素对照品、芍药苷对照品、1,2,3,4,6-五没食子酰葡萄糖对照品、苯甲酰芍药苷对照品适量，精密称定，加甲醇制成每 1ml 含没食子酸 50μg、儿茶素 30μg、芍药苷 160μg、1,2,3,4,6-五没

食子酰葡萄糖 30μg、苯甲酰芍药苷 30μg 的混合溶液，作为对照品参照物溶液。

供试品溶液的制备　取本品适量，研细，取约 0.1g，置具塞锥形瓶中，加稀乙醇 50ml，超声处理（功率 250W，频率 40kHz）30 分钟，放冷，摇匀，滤过，取续滤液，即得。

测定法　分别精密吸取参照物溶液与供试品溶液各 10μl，注入液相色谱仪，测定，即得。

供试品色谱中应呈现 6 个特征峰，并应与对照药材参照物色谱中的 6 个特征峰保留时间相对应，其中 5 个峰应分别与相应对照品参照物峰的保留时间相对应。与芍药苷参照物峰相对应的峰为 S 峰，计算峰 3 与 S 峰的相对保留时间，其相对保留时间应在规定值的±10%范围之内，规定值为：0.90（峰 3）；计算峰 3、峰 6 与 S 峰的相对峰面积，其相对峰面积应在规定的范围之内，规定范围为：不低于 0.089（峰 3）、不低于 0.020（峰 6）。

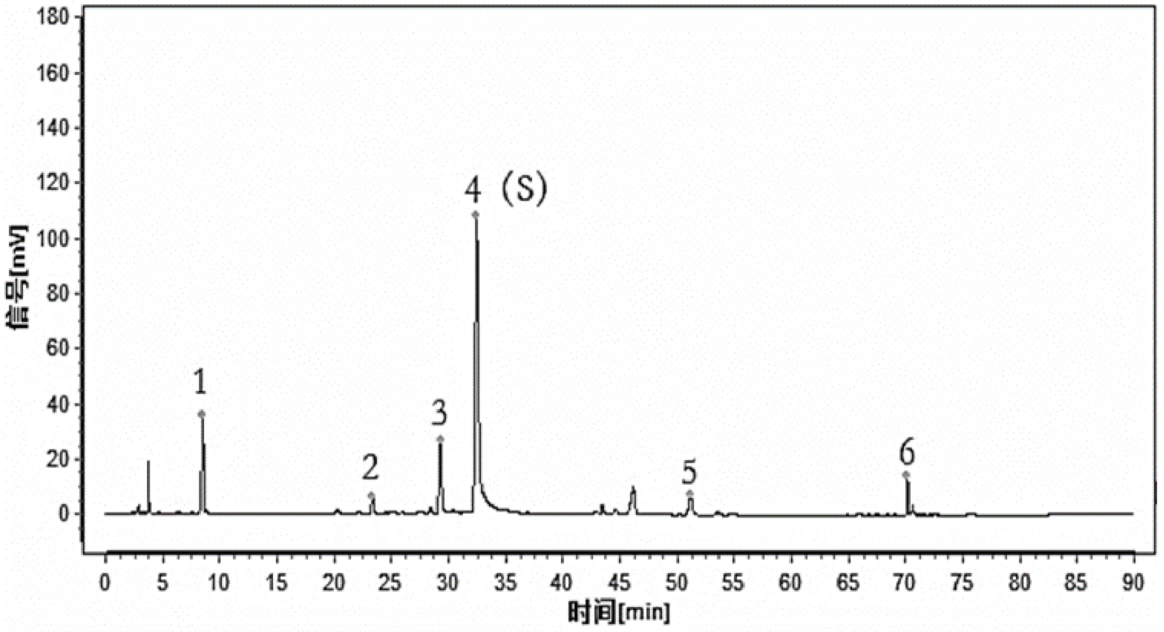

对照特征图谱

峰 1：没食子酸；峰 2：儿茶素；峰 3：芍药内酯苷；峰 4（S）：芍药苷；

峰 5：1,2,3,4,6-五没食子酰葡萄糖；峰 6：苯甲酰芍药苷

色谱柱：Triart C18，4.6mm×250mm，5μm

【检查】　**硫熏检查**　在〔特征图谱〕项下供试品色谱中，与 S 峰相对保留时间 0.59±10%的范围之内不得检出色谱峰，如果检出色谱峰，其与 S 峰的相对峰面积不得大于 0.15。

重金属及有害元素　照铅、镉、砷、汞、铜测定法（中国药典 2020 年版通则 2321 原子吸收分光光度法或电感耦合等离子体质谱法）测定，铅不得过 5mg/kg；镉不得过 1 mg/kg；砷不得过 2mg/kg；汞不得过 0. 2mg/kg；铜不得过 20mg/kg。

其他　应符合颗粒剂项下有关的各项规定（中国药典 2020 年版通则 0104）。

【浸出物】　取本品研细，取约 2g，精密称定，精密加入乙醇 100ml，照醇溶性浸出物测定法（中国药典 2020 年版通则 2201）项下的热浸法测定，不得少于 35.0%。

【含量测定】　照高效液相色谱法（中国药典 2020 年版通则 0512）测定。

色谱条件与系统适用性试验　以十八烷基硅烷键合硅胶为填充剂，以乙腈-0.1%磷酸溶液（14∶86）为流动相；检测波长为 230nm。理论板数按芍药苷峰计算应不低于 2000。

对照品溶液的制备　取芍药苷对照品适量，精密称定，加甲醇制成每 1ml 含 120μg 的溶液，即得。

供试品溶液的制备　取本品适量，研细，取约 0.1g，精密称定，置具塞锥形瓶中，精密加入甲醇 50ml，称定重量，超声处理（功率 250W，频率 40kHz）30 分钟，放冷，再称定重量，用甲醇补足减失的重量，摇匀，滤过，取续滤液，即得。

测定法　分别精密吸取对照品溶液与供试品溶液各 10μl，注入液相色谱仪，测定，即得。

本品每 1g 含芍药苷（$C_{23}H_{28}O_{11}$）应为 65.0mg~137.0mg。

【注意】　不宜与藜芦同用。

【规格】　每 1g 配方颗粒相当于饮片 4.5g

【贮藏】　密封。
