# Supplementary material for: Efficacy of Xiaoyao-san preparations in treating Hashimoto’s thyroiditis: a meta-analysis and systematic review
Source: Front Pharmacol. 2025 Jun 13;16:1528506. doi: 10.3389/fphar.2025.1528506 (PMC12202410; doi:10.3389/fphar.2025.1528506)
Supplement: Supplementary file 2 [file Supplementaryfile2.zip › Supplementary Files 2/Supplementary Files 2 formula granules section/Shudihuang_YBZ-PFKL-2021118 .pdf]

国家药品监督管理局
国家药品标准

YBZ-PFKL-2021118

熟地黄配方颗粒
Shudihuang Peifangkeli

【来源】 本品为玄参科植物地黄 *Rehmannia glutinosa* Libosch.的干燥块根经炮制并按标准汤剂的主要质量指标加工制成的配方颗粒。

【制法】 取熟地黄饮片1300g，加水煎煮，滤过，滤液浓缩成清膏（干浸膏出膏率为44%~76.5%），加辅料适量，干燥（或干燥，粉碎），再加辅料适量，混匀，制粒，制成1000g，即得。

【性状】 本品为灰棕色至棕褐色的颗粒；气微，味甜。

【鉴别】 取本品1g，研细，加水10ml使溶解，用水饱和正丁醇振摇提取3次，每次10ml，合并正丁醇液，蒸干，残渣加甲醇1ml使溶解，作为供试品溶液。另取熟地黄对照药材1g，加水50ml，煎煮30分钟，滤过，滤液浓缩至10ml，同法制成对照药材溶液。再取毛蕊花糖苷对照品，加甲醇制成每1ml含1mg的溶液，作为对照品溶液。照薄层色谱法（中国药典2020年版通则0502）试验，吸取供试品溶液和对照药材溶液各2~5μl、对照品溶液2μl，分别点于同一聚酰胺薄膜上，以甲醇-冰醋酸-水（2∶1∶7）为展开剂，取出，晾干，置紫外光灯（365nm）下检视。供试品色谱中，在与对照药材色谱和对照品色谱相应的位置上，显相同颜色的荧光斑点。

【特征图谱】 照高效液相色谱法（中国药典2020年版通则0512）测定。

色谱条件与系统适用性试验 以十八烷基硅烷键合硅胶为填充剂（柱长为100mm，内径为2.1mm，粒径为1.8μm）；以乙腈为流动相A，以0.1%醋酸溶液为流动相B，按下表中的规定进行梯度洗脱；流速为每分钟0.3ml；柱温为35℃；检测波长为330nm。理论板数按毛蕊花糖苷峰计算应不低于5000。

时间（分钟）	流动相A（%）	流动相B（%）
0～5	0→14	100→86
5～15	14→22	86→78
15～22	22→30	78→70
22～28	30→100	70→0
28～30	100→0	0→100

参照物溶液的制备 取熟地黄对照药材1g，置具塞锥形瓶中，加入甲醇100ml，加热回流1.5小时，放冷，摇匀，滤过，精密量取续滤液50ml，回收溶剂至近干，残渣用乙腈-0.1%醋酸溶液（16∶84）混合溶液溶解，转移至5ml量瓶中，加乙腈-0.1%醋酸溶液（16∶84）混合溶液至刻度，摇匀，滤过，取续滤液，作为对照药材参照物溶液。另取毛蕊花糖苷对照品适量，精密称定，加甲醇制成每1ml含10μg的溶液，作为对照品参照物溶液。

供试品溶液的制备 取本品适量，研细，取约0.2g，精密称定，置具塞锥形瓶中，精密加入30%

甲醇20ml，密塞，称定重量，超声处理（功率250W，频率40kHz）20分钟，放冷，再称定重量，用30%甲醇补足减失的重量，摇匀，滤过，取续滤液，即得。

测定法　分别精密吸取参照物溶液 1μl 与供试品溶液 4μl，注入液相色谱仪，测定，即得。

供试品色谱中应呈现 9 个特征峰，并应与对照药材参照物色谱中 9 个特征峰保留时间相对应，其中峰 4 应与毛蕊花糖苷对照品参照物峰保留时间相对应。与毛蕊花糖苷参照物峰相应的峰为 S 峰，计算峰 2~峰 9 与 S 峰的相对保留时间，其相对保留时间应在规定值的±10%范围之内。规定值为： 0.70（峰 2）、0.83（峰 3）、1.06（峰 5）、1.10（峰 6）、1.23（峰 7）、1.53（峰 8）、1.64（峰 9）。

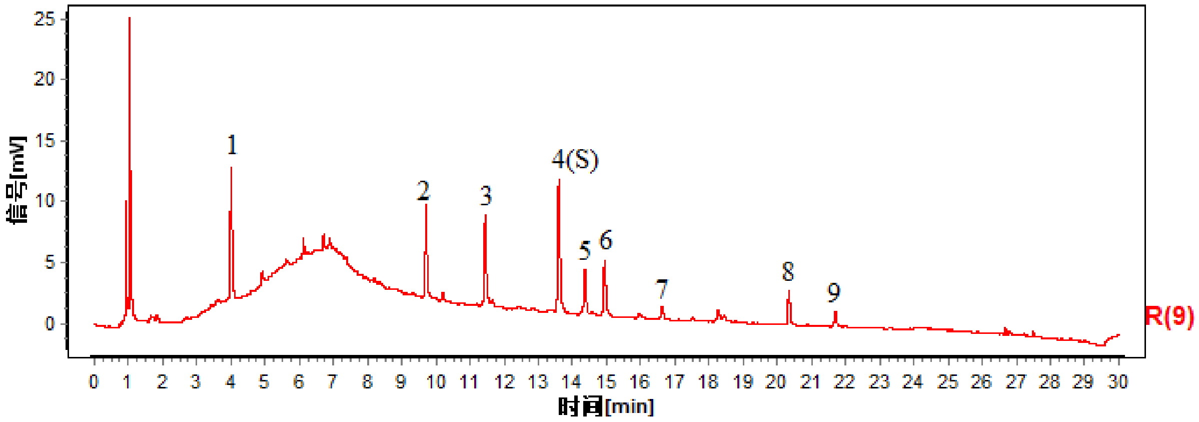

对照特征图谱

峰 2：洋地黄叶苷 C；峰 3：焦地黄苯乙醇苷 A1；峰 4（S）：毛蕊花糖苷；

峰 5：焦地黄苯乙醇苷 B1；峰 6：异毛蕊花糖苷

色谱柱：HSS T3 C18，2.1mm×100mm，1.8μm

【检查】　应符合颗粒剂项下有关的各项规定（中国药典 2020 年版通则 0104）。

【浸出物】　照醇溶性浸出物测定法（中国药典 2020 年版通则 2201）项下的热浸法测定，用乙醇作溶剂，不得少于 12.0%。

【含量测定】　照高效液相色谱法（中国药典 2020 年版通则 0512）测定。

色谱条件与系统适用性试验　以十八烷基硅烷键合硅胶为填充剂；以甲醇-0.1%磷酸（5∶95）为流动相；检测波长为 203nm。理论板数按地黄苷 D 峰计算应不低于 5000。

对照品溶液的制备　取地黄苷 D 对照品适量，精密称定，加 25%甲醇制成每 1ml 含 70μg 的溶液，即得。

供试品溶液的制备　取本品适量，研细，取约 0.7g，精密称定，置具塞锥形瓶中，精密加入水 25ml，密塞，称定重量，充分振摇 30 分钟，再称定重量，用水补足减失的重量，摇匀，滤过，取续滤液，即得。

测定法　分别精密吸取对照品溶液 1μl 与供试品溶液 2μl，注入液相色谱仪，测定，即得。

本品每 1g 含地黄苷 D（$C_{27}H_{42}O_{20}$）应为 0.70mg~2.70mg。

【规格】　每 1g 配方颗粒相当于饮片 1.3g

【贮藏】　密封。
