# Supplementary material for: Efficacy of Xiaoyao-san preparations in treating Hashimoto’s thyroiditis: a meta-analysis and systematic review
Source: Front Pharmacol. 2025 Jun 13;16:1528506. doi: 10.3389/fphar.2025.1528506 (PMC12202410; doi:10.3389/fphar.2025.1528506)
Supplement: Supplementary file 2 [file Supplementaryfile2.zip › Supplementary Files 2/Supplementary Files 2 formula granules section/Taizishen_YBZ-PFKL-2022046.pdf]

国家药品监督管理局
国家药品标准

YBZ-PFKL-2022046

太子参配方颗粒

Taizishen Peifangkeli

【来源】 本品为石竹科植物孩儿参 *Pseudostellaria heterophylla*（Miq.）Pax ex Pax et Hoffm. 的干燥块根经炮制并按标准汤剂的主要质量指标加工制成的配方颗粒。

【制法】 取太子参饮片 2500g，加水煎煮，滤过，滤液浓缩成清膏（干浸膏出膏率为 24%～39%），加入辅料适量，干燥（或干燥，粉碎），再加入辅料适量，混匀，制粒，制成 1000g，即得。

【性状】 本品为浅黄色至棕黄色的颗粒；气微，味微甘。

【鉴别】 （1）取本品 0.2g，研细，加甲醇 10ml，超声处理 30 分钟，滤过，滤液浓缩至 1ml，作为供试品溶液。另取太子参对照药材 1g，加水 50ml，加热回流 30 分钟，滤过，滤液蒸干，残渣加甲醇 10ml，同法制成对照药材溶液。照薄层色谱法（中国药典 2020 年版通则 0502）试验，吸取上述两种溶液各 5μl，分别点于同一硅胶 G 薄层板上，以正丁醇-冰醋酸-水（3∶1∶1）为展开剂，展开，取出，晾干，喷以 1%茚三酮乙醇溶液，在 105℃加热至斑点显色清晰。供试品色谱中，在与对照药材色谱相应的位置上显相同颜色的斑点。

（2）取本品 2g，研细，加甲醇 20ml，超声处理 30 分钟，滤过，滤液蒸干，残渣加水 20ml 使溶解，用水饱和的正丁醇振摇提取 2 次，每次 20ml，合并正丁醇液，蒸干，残渣加甲醇 1ml 使溶解，作为供试品溶液。另取太子参对照药材 1g，加水 50ml，煮沸 30 分钟，滤过，滤液蒸干，残渣加甲醇 20ml，同法制成对照药材溶液。照薄层色谱法（中国药典 2020 年版通则 0502）试验，吸取供试品溶液 1μl、对照药材溶液 5μl，分别点于同一硅胶 G 薄层板上，以正丁醇-乙酸乙酯-甲酸-水（4∶1∶0.2∶1）为展开剂，展开，取出，晾干，喷以 10%硫酸乙醇溶液，在 105℃加热至斑点显色清晰，置紫外光灯（365nm）下检视，供试品色谱中，在与对照药材色谱相应的位置上，显相同颜色的荧光斑点。

【特征图谱】 照高效液相色谱法（中国药典 2020 年版通则 0512）测定。

色谱条件与系统适用性试验 以十八烷基硅烷键合硅胶为填充剂（柱长为 250mm，内径为 4.6mm，粒径为 5μm）；以乙腈为流动相 A，以 0.1%磷酸溶液为流动相 B，按下表中的规定进行梯度洗脱；流速为每分钟 0.8ml；柱温为 35℃；检测波长为 220nm。理论板数按色氨酸峰计算应不低于 1500。

时间（分钟）	流动相 A（%）	流动相 B（%）
0~15	4	96
15~26	4→11	96→89
26~30	11→12	89→88
30~45	12	88
45~55	12→25	88→75
55~65	25→55	75→45

参照物溶液的制备　取太子参对照药材 1g，加水 50ml，加热回流 30 分钟，放冷，滤过，滤液蒸干，残渣加甲醇 50ml，超声处理（功率 300W，频率 40kHz）30 分钟，滤过，滤液蒸干，残渣加乙腈-0.1%磷酸溶液（4∶96）适量使溶解并转移至 5ml 量瓶中，加乙腈-0.1%磷酸溶液（4∶96）至刻度，摇匀，滤过，取续滤液，作为对照药材参照物溶液。另取色氨酸对照品适量，精密称定，加甲醇制成每 1ml 含 4μg 的溶液，作为对照品参照物溶液。

供试品溶液的制备　取本品适量，研细，取 0.5g，置具塞锥形瓶中，加甲醇 50ml，同对照药材参照物溶液制备方法制成供试品溶液。

测定法　分别精密吸取参照物溶液与供试品溶液各 10μl，注入液相色谱仪，测定，即得。

供试品色谱中应呈现 7 个特征峰，并应与对照药材参照物色谱中的 7 个特征峰的保留时间相对应，其中峰 4 应与对照品参照物峰的保留时间相对应。与色氨酸对照品参照物峰相对应的峰为 S 峰，计算各特征峰与 S 峰的相对保留时间，其相对保留时间应在规定值的±10%范围之内，规定值为：0.37（峰 1）、0.47（峰 2）、0.80（峰 3）、1.39（峰 5）、1.76（峰 6）、1.87（峰 7）；计算峰 6 与 S 峰的相对峰面积，其相对峰面积应在规定值的范围之内，规定值为：不得小于 0.65（峰 6）。

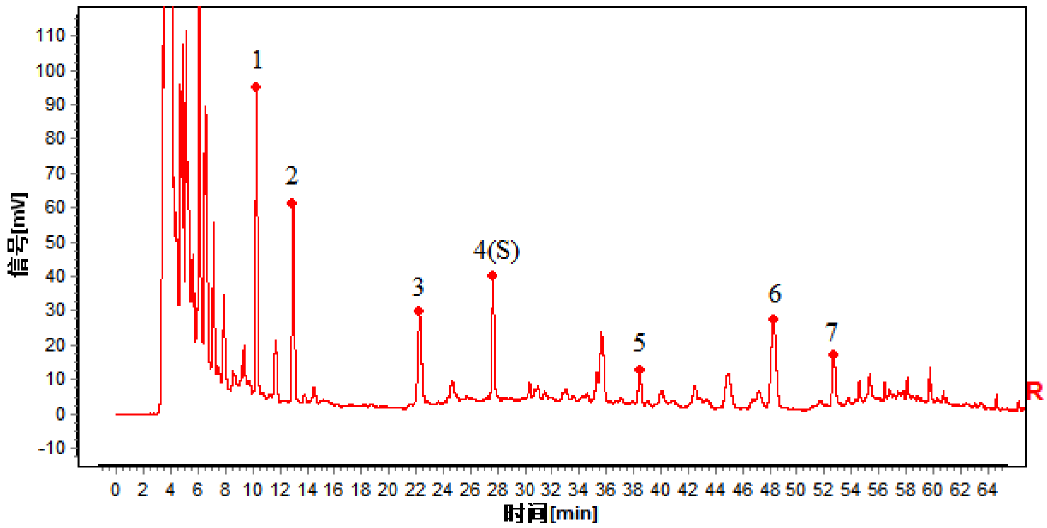

对照特征图谱

峰 3：2-吡咯甲酸；峰 4（S）：色氨酸

色谱柱：Diamonsil Plus C18，4.6mm×250mm，5μm

【检查】 应符合颗粒剂项下有关的各项规定（中国药典 2020 年版通则 0104）。

【浸出物】 取本品适量，研细，取约 2g，精密称定，精密加入乙醇 100ml，照醇溶性浸出物测定法（中国药典 2020 年版通则 2201）项下的热浸法测定，不得少于 11.0%。

【含量测定】 **总皂苷** 照紫外-可见分光光度法（中国药典 2020 年版通则 0401）测定。

对照品溶液的制备 取人参皂苷 Rg_1 对照品适量，精密称定，加甲醇制成每 1ml 含 0.15mg 的溶液，即得。

标准曲线的制备 精密移取对照品溶液 0.4ml、0.6ml、0.8ml、1.0ml、1.2ml、1.4ml，分别置 25ml 具塞试管中，水浴挥去溶剂，残渣精密加入 5%香草醛-冰醋酸溶液 0.2ml 使溶解，另加入 60%硫酸溶液 2ml，摇匀，置 60℃恒温水浴上加热 30 分钟，取出，放冷，再加冰醋酸 5ml，摇匀；以相应的试剂为空白，照紫外-可见分光光度法（中国药典 2020 年版通则 0401），在 483nm 的波长处分别测定吸光度，以吸光度为纵坐标，浓度为横坐标绘制标准曲线。

测定法 取本品适量，研细，取约 0.6g，精密称定，置具塞锥形瓶中，精密加入甲醇 50ml，称定重量，超声处理（功率 250W，频率 40kHz）30 分钟，取出，放冷，再称定重量，用甲醇补足减失的重量，摇匀，滤过，精密移取续滤液 20ml，蒸干，残渣加 10ml 水使溶解，用水饱和的正丁醇振摇提取 3 次，每次 20ml，合并正丁醇液，蒸干，残渣加甲醇溶解，转移至 25ml 量瓶中，加甲醇至刻度，摇匀，精密吸取 1ml，置 25ml 具塞试管中，照标准曲线制备项下的方法，自“水浴挥去溶剂”起，依法测定吸光度，从标准曲线上读出供试品溶液中人参皂苷 Rg_1 的重量，计算，即得。

本品每 1g 含总皂苷以人参皂苷 Rg_1（$C_{42}H_{72}O_{14}$）计，应为 6.0mg～21.0mg。

太子参环肽 B 照高效液相色谱法（中国药典 2020 年版通则 0512）测定。

色谱条件与系统适用性试验 以十八烷基硅烷键合硅胶为填充剂（柱长为 50mm，内径为 2.1mm，粒径为 1.6μm）；以乙腈为流动相 A，以水为流动相 B，按下表中的规定进行梯度洗脱；流速为每分钟 0.4ml；柱温为 30℃；检测波长为 210nm。理论板数按太子参环肽 B 峰计算应不低于 5000。

时间（分钟）	流动相 A（%）	流动相 B（%）
0～4	15	85
4～14	15→40	85→60
14～16	40→45	60→55

16～17	45→85	55→15

对照品溶液的制备　取太子参环肽B对照品适量，精密称定，加甲醇制成每1ml含4μg的溶液，即得。

供试品溶液的制备　取本品适量，研细，取约0.2g，精密称定，置具塞锥形瓶中，精密加入稀乙醇25ml，称定重量，超声处理（功率250W，频率40kHz）30分钟，放冷，再称定重量，用稀乙醇补足减失的重量，摇匀，滤过，取续滤液，即得。

测定法　分别精密吸取对照品溶液与供试品溶液各2μl，注入液相色谱仪，测定，即得。

本品每1g含太子参环肽B（$C_{40}H_{58}N_8O_8$）应为0.23mg～0.65mg。

【规格】　每1g配方颗粒相当于饮片2.5g

【贮藏】　密封。
