# Supplementary material for: Efficacy of Xiaoyao-san preparations in treating Hashimoto’s thyroiditis: a meta-analysis and systematic review
Source: Front Pharmacol. 2025 Jun 13;16:1528506. doi: 10.3389/fphar.2025.1528506 (PMC12202410; doi:10.3389/fphar.2025.1528506)
Supplement: Supplementary file 2 [file Supplementaryfile2.zip › Supplementary Files 2/Supplementary Files 2 formula granules section/Xiangyuan_YBZ-PFKL-2021129.pdf]

国家药品监督管理局
国家药品标准

YBZ-PFKL-2021129

香橼（香圆）配方颗粒

Xiangyuan（Xiangyuan） Peifangkeli

【来源】 本品为芸香科植物香圆 *Citrus wilsonii* Tanaka 的干燥成熟果实经炮制并按标准汤剂的主要质量指标加工制成的配方颗粒。

【制法】 取香橼饮片 2000g，加水煎煮，滤过，滤液浓缩成清膏（干浸膏出膏率为 25%～38%），加辅料适量，干燥（或干燥，粉碎），再加辅料适量，混匀，制粒，制成 1000g，即得。

【性状】 本品为淡黄色至黄棕色的颗粒；气微，味酸而苦。

【鉴别】 取本品适量，研细，取 1g，加甲醇 30ml，超声处理 30 分钟，滤过，滤液蒸干，残渣加甲醇 2ml 使溶解，作为供试品溶液。另取香橼（香圆）对照药材 1g，加水 50ml，煮沸 30 分钟，滤过，滤液蒸干，残渣加甲醇 30ml，同法制成对照药材溶液。照薄层色谱法（中国药典 2020 版通则 0502）试验，吸取上述两种溶液各 5μl，分别点于同一硅胶 G 薄层板上，以甲苯-乙酸乙酯-甲酸（8∶2∶0.5）为展开剂，展开，取出，晾干，喷以 3%三氯化铝乙醇溶液，置紫外光灯（365nm）下检视。供试品色谱中，在与对照药材色谱相应的位置上，显相同颜色的荧光主斑点。

【特征图谱】 照高效液相色谱法（中国药典 2020 年版通则 0512）测定。

色谱条件与系统适用性试验 以十八烷基硅烷键合硅胶为填充剂（柱长为 250mm，内径为 4.6mm，粒径为 5μm）；以甲醇-乙腈（1:1）为流动相 A，以 1%冰醋酸溶液为流动相 B，按下表中的规定进行梯度洗脱；柱温为 35℃；检测波长为 320nm。理论板数按柚皮苷峰计算应不低于 4000。

时间（分钟）	流动相 A（%）	流动相 B（%）
0～15	17→30	83→70
15～30	30	70
30～60	30→60	70→40

参照物溶液的制备 取香橼（香圆）对照药材 0.5g，置具塞锥形瓶中，加 50%甲醇 40ml，密塞，超声处理（功率 600W，频率 40kHz）30 分钟，放冷，摇匀，滤过，取续滤液，作为对照药材参照物溶液。另取柚皮苷对照品适量，加 50%甲醇制成每 1ml 含 30μg 的溶液，作为对照品参照物溶液。

供试品溶液的制备 取本品适量，研细，取约 0.2g，同对照药材参照物溶液制备方法制成供试品溶液。

测定法 分别精密吸取参照物溶液和供试品溶液各 10μl，注入液相色谱仪，测定，即得。

供试品色谱中应呈现 8 个特征峰，并应与对照药材参照物色谱中的 8 个特征峰保留时间相对应，与柚皮苷参照物峰相对应的峰为 S 峰，计算各特征峰与 S 峰的相对保留时间，其相对保留时间应在规定值的±10%范围之内。规定值为：0.449（峰 1）、0.694（峰 2）、0.774（峰 3）、1.057（峰 5）、

1.886（峰 6）、2.546（峰 7）、2.622（峰 8）。

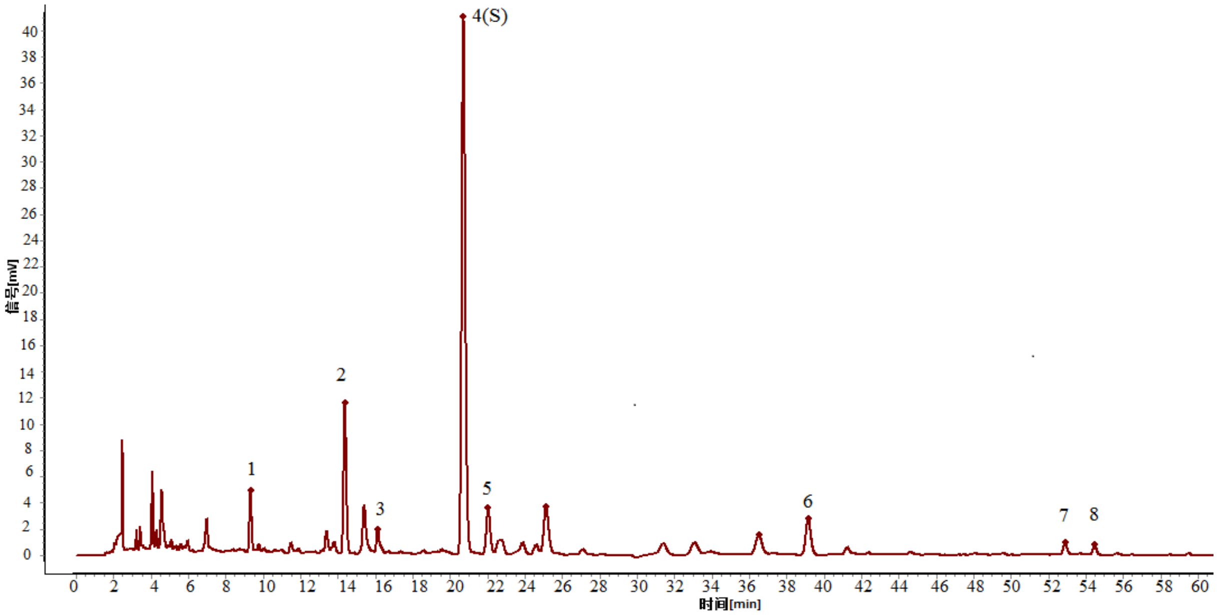

对照特征图谱

峰 3：异牡荆苷；峰 4（S）：柚皮苷

色谱柱：100Å C18，4.6mm×250mm，5μm

【检查】 应符合颗粒剂项下有关的各项规定（中国药典 2020 年版通则 0104）。

【浸出物】 照醇溶性浸出物测定法（中国药典 2020 年版通则 2201）项下的热浸法测定，用乙醇作溶剂，不得少于 12.0%。

【含量测定】 照高效液相色谱法（中国药典 2020 年版通则 0512）测定。

色谱条件与系统适用性试验 以十八烷基硅烷键合硅胶为填充剂；以甲醇-水-冰醋酸（30∶63∶3）为流动相；检测波长为 284nm。理论板数按柚皮苷峰计算应不低于 4000。

对照品溶液的制备 取柚皮苷对照品适量，精密称定，加 50%甲醇制成每 1ml 含 30μg 的溶液，即得。

供试品溶液的制备 取本品适量，研细，取约 0.1g，精密称定，置具塞锥形瓶中，精密加入 50%甲醇 50ml，称定重量，超声处理（功率 600W，频率 40kHz）20 分钟，放冷，再称定重量，用 50%甲醇补足减失的重量，摇匀，滤过，取续滤液，即得。

测定法 分别精密吸取对照品溶液与供试品溶液各 5μl，注入液相色谱仪，测定，即得。

本品每 1g 含柚皮苷（$C_{27}H_{32}O_{14}$）应为 30.0mg～90.0mg。

【规格】 每 1g 配方颗粒相当于饮片 2g

【贮藏】 密封。
