# Supplementary material for: Efficacy of Xiaoyao-san preparations in treating Hashimoto’s thyroiditis: a meta-analysis and systematic review
Source: Front Pharmacol. 2025 Jun 13;16:1528506. doi: 10.3389/fphar.2025.1528506 (PMC12202410; doi:10.3389/fphar.2025.1528506)
Supplement: Supplementary file 2 [file Supplementaryfile2.zip › Supplementary Files 2/Supplementary Files 2 formula granules section/Zhizi_YBZ-PFKL-2021150 .pdf]

国家药品监督管理局
国家药品标准

YBZ-PFKL-2021150

栀子配方颗粒

Zhizi Peifangkeli

【来源】 本品为茜草科植物栀子 *Gardenia jasminoides* Ellis 的干燥成熟果实经炮制并按标准汤剂的主要质量指标加工制成的配方颗粒。

【制法】 取栀子饮片 3000g，加水煎煮，滤过，滤液浓缩成清膏（干浸膏出膏率为 20%~28%），加辅料适量，干燥（或干燥，粉碎），再加辅料适量，混匀，制粒，制成 1000g，即得。

【性状】 本品为浅黄色至棕黄色的颗粒；气微，味微酸而苦。

【鉴别】 取本品适量，研细，取 0.2g，加 75%乙醇 10ml，超声处理 20 分钟，滤过，滤液蒸干，残渣加甲醇 1ml 使溶解，作为供试品溶液。另取栀子对照药材 1g，同法制成对照药材溶液。再取栀子苷对照品，加乙醇制成每 1ml 含 4mg 的溶液，作为对照品溶液。照薄层色谱法（中国药典 2020 年版通则 0502）试验，吸取上述三种溶液各 5μl，分别点于同一硅胶 G 薄层板上，以乙酸乙酯-丙酮-甲酸-水（5 : 5 : 1 : 1）为展开剂，展开，取出，晾干，喷以 10%硫酸乙醇溶液，在 105℃加热至斑点显色清晰。供试品色谱中，在与对照药材色谱和对照品色谱相应的位置上，显相同颜色的斑点。

【特征图谱】 照高效液相色谱法（中国药典 2020 年版通则 0512）测定。

色谱条件与系统适用性试验 以十八烷基硅烷键合硅胶为填充剂(柱长为 250mm，内径为 4.6mm，粒径为 5μm)；以乙腈为流动相 A，以 0.4%磷酸溶液为流动相 B，按下表中的规定进行梯度洗脱；检测波长：0~23 分钟为 238nm，23~40 分钟为 440nm。理论板数按栀子苷峰计算应不低于 3000。

时间（分钟）	流动相 A（%）	流动相 B（%）
0～10	8→15	92→85
10～15	15→20	85→80
15～20	20→25	80→75
20～40	25→30	75→70

参照物溶液的制备 取栀子对照药材 0.1g，置具塞锥形瓶中，加 50%乙醇 50ml，超声处理（功率 250W，频率 50kHz）20 分钟，放冷，滤过，取续滤液，作为对照药材参照物溶液。另取栀子苷对照品适量，精密称定，加甲醇制成每 1ml 含 30μg 的溶液，作为对照品参照物溶液。

供试品溶液的制备 同〔含量测定〕项。

测定法 分别精密吸取参照物溶液与供试品溶液各 10μl，注入液相色谱仪，测定，即得。

供试品色谱中应呈现 6 个特征峰，并应与对照药材参照物色谱中的 6 个特征峰保留时间相对应，与栀子苷参照物峰相对应的峰为 S 峰，计算各特征峰与 S 峰的相对保留时间，其相对保留时间应在规

定值的±8%范围之内。规定值为：0.409（峰 1）、0.704（峰 2）、0.806（峰 3）、1.455（峰 5）、1.709（峰 6）。

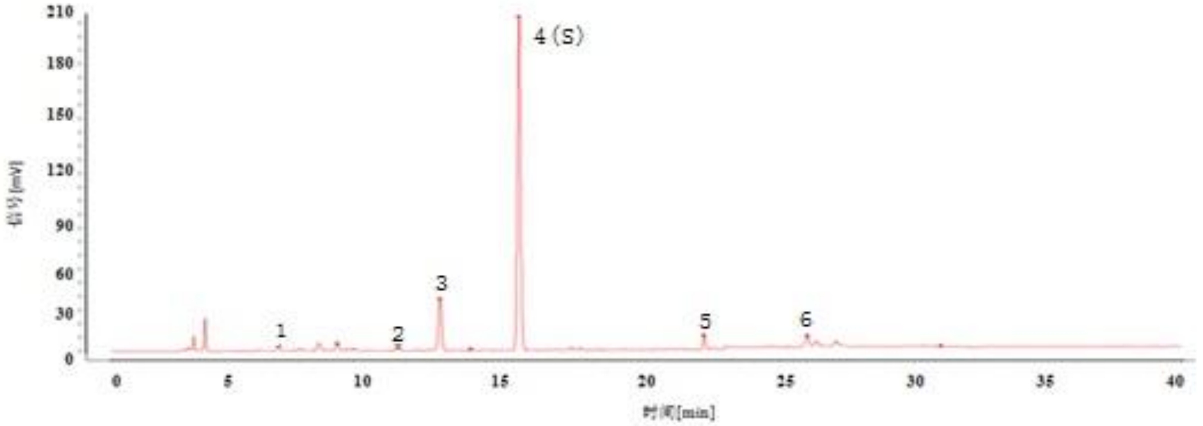

对照特征图谱

峰 3：京尼平 1-β-D 龙胆双糖苷；峰 4（S）：栀子苷；峰 6：西红花苷 I

色谱柱：TC C18，4.6mm×250mm，5μm

【检查】 **重金属及有害元素** 照铅、镉、砷、汞、铜测定法（中国药典 2020 年版通则 2321 原子吸收分光光度法或电感耦合等离子体质谱法）测定，铅不得过 5mg/kg；镉不得过 1 mg/kg；砷不得过 2mg/kg；汞不得过 0. 2mg/kg；铜不得过 20mg/kg。

其他 应符合颗粒剂项下有关的各项规定（中国药典 2020 年版通则 0104）。

【浸出物】 照醇溶性浸出物测定法（中国药典 2020 年版通则 2201）项下的热浸法测定，用乙醇作溶剂，不得少于 32.0%。

【含量测定】 照高效液相色谱法（中国药典 2020 年版通则 0512）测定。

色谱条件与系统适用性试验 以十八烷基硅烷键合硅胶为填充剂（柱长为 250mm，内径为 4.6mm，粒径为 5μm）；以乙腈-水（15 : 85）为流动相；检测波长为 238nm。理论板数按栀子苷峰计算应不低于 1500。

对照品溶液的制备 取栀子苷对照品适量，精密称定，加甲醇制成每 1ml 含 30μg 的溶液，即得。

供试品溶液的制备 取本品适量，研细，取约 0.1g，精密称定，置具塞锥形瓶中，加 50%乙醇 50ml，密塞，称定重量，超声处理（功率 250W，频率 50kHz）20 分钟，放冷，再称定重量，用 50%乙醇补足减失的重量，摇匀，滤过，取续滤液，即得。

测定法 分别精密吸取对照品溶液与供试品溶液各 10μl，注入液相色谱仪，测定，即得。

本品每 1g 含栀子苷（$C_{17}H_{24}O_{10}$）应为 64.0mg ~ 184.0mg。

【规格】 每 1g 配方颗粒相当于饮片 3g

【贮藏】 密封。
