# Supplementary material for: Efficacy of Xiaoyao-san preparations in treating Hashimoto’s thyroiditis: a meta-analysis and systematic review
Source: Front Pharmacol. 2025 Jun 13;16:1528506. doi: 10.3389/fphar.2025.1528506 (PMC12202410; doi:10.3389/fphar.2025.1528506)
Supplement: Supplementary file 2 [file Supplementaryfile2.zip › Supplementary Files 2/Supplementary Files 2 general rules section.docx]

**Efficacy of Xiao-yao San preparations in treating Hashimoto's thyroiditis: a meta-analysis and systematic review**

**Supplementary Files 2 (General Rules Section)**

[Part 1 General Rules for Preparation of Tablets (0101) 2](#_Toc187757887)

[Part 2 General Rules for Preparation of Granules (0104) 5](#_Toc187757888)

[Part 3 General Rules for Preparation of Pills (0108) 7](#_Toc187757889)

[Part 4 General Rules for Preparation of Mixtures (0181) 10](#_Toc187757890)

[Part 5 General Rules for Preparation of Soft extract (0183) 11](#_Toc187757891)

[Part 5 General Rules for Thin layer chromatography (0502) 12](#_Toc187757892)

# Part 1 General Rules for Preparation of Tablets (0101)

**0101 片剂**

片剂系指原料药物或与适宜的辅料制成的圆形或异形的片状固体制剂。

　　中药还有浸膏片、半浸膏片和全粉片等。

　　片剂以口服普通片为主，另有含片、舌下片、口腔贴片、咀嚼片、分散片、可溶片、泡腾片、阴道片、阴道泡腾片、缓释片、控释片、肠溶片与口崩片等。

　　含片 系指含于口腔中缓慢溶化产生局部或全身作用的片剂。

　　含片中的原料药物一般是易溶性的，主要起局部消炎、杀菌、收敛、止痛或局部麻醉等作用。

　　舌下片 系指置于舌下能迅速溶化，药物经舌下黏膜吸收发挥全身作用的片剂。

　　舌下片中的原料药物应易于直接吸收，主要适用于急症的治疗。

　　口腔贴片 系指粘贴于口腔，经黏膜吸收后起局部或全身作用的片剂。

　　口腔贴片应进行溶出度或释放度（通则0931）检查。

　　咀嚼片 系指于口腔中咀嚼后吞服的片剂。

　　咀嚼片一般应选择甘露醇、山梨醇、蔗糖等水溶性辅料作填充剂和黏合剂。咀嚼片的硬度应适宜。

　　分散片 系指在水中能迅速崩解并均匀分散的片剂。

　　分散片中的原料药物应是难溶性的。分散片可加水分散后口服，也可将分散片含于口中吮服或吞服。

　　分散片应进行溶出度（通则0931）和分散均匀性检查。

　　可溶片 系指临用前能溶解于水的非包衣片或薄膜包衣片剂。

　　可溶片应溶解于水中，溶液可呈轻微乳光。可供口服、外用、含漱等用。

　　泡腾片 系指含有碳酸氢钠和有机酸，遇水可产生气体而呈泡腾状的片剂。泡腾片不得直接吞服。

　　泡腾片中的原料药物应是易溶性的，加水产生气泡后应能溶解。有机酸一般用枸椽酸、酒石酸、富马酸等。

　　阴道片与阴道泡腾片 系指置于阴道内使用的片剂。阴道片和阴道泡腾片的形状应易置于阴道内，可借助器具将其送入阴道。阴道片在阴道内应易溶化、溶散或融化、崩解并释放药物，主要起局部消炎杀菌作用，也可给予性激素类药物。具有局部刺激性的药物，不得制成阴道片。

　　阴道片应进行融变时限检查（通则0922）。阴道泡腾片还应进行发泡量检查。

　　缓释片 系指在规定的释放介质中缓慢地非恒速释放药物的片剂。缓释片应符合缓释制剂的有关要求（指导原则9013）并应进行释放度（通则0931）检查。除说明书标注可掰开服用外，一般应整片吞服。

　　控释片 系指在规定的释放介质中缓慢地恒速释放药物的片剂。控释片应符合控释制剂的有关要求（指导原则9013）并应进行释放度（通则0931）检查。除说明书标注可掰开服用外，一般应整片吞服。

　　肠溶片 系指用肠溶性包衣材料进行包衣的片剂。

　　为防止原料药物在胃内分解失效、对胃的刺激或控制原料药物在肠道内定位释放，可对片剂包肠溶衣；为治疗结肠部位疾病等，可对片剂包结肠定位肠溶衣。除说明书标注可掰开服用外，一般不得掰开服用。

　　肠溶片除另有规定外，应符合迟释制剂（指导原则9013）的有关要求，并进行释放度（通则0931）检查。

　　口崩片 系指在口腔内不需要用水即能迅速崩解或溶解的片剂。

　　一般适合于小剂量原料药物，常用于吞咽困难或不配合服药的患者。可采用直接压片和冷冻干燥法制备。

　　口崩片应在口腔内迅速崩解或溶解、口感良好、容易吞咽，对口腔黏膜无刺激性。

　　除冷冻干燥法制备的口崩片外，口崩片应进行崩解时限检查（通则0921）。对于难溶性原料药物制成的口崩片，还应进行溶出度检查（通则0931）。对于经肠溶材料包衣的颗粒制成的口崩片，还应进行释放度检查（通则0931）。

　　釆用冷冻干燥法制备的口崩片可不进行脆碎度检查。

　　片剂在生产与贮藏期间应符合下列规定。

　　一、原料药物与辅料应混合均匀。含药量小或含毒、剧药的片剂，应根据原料药物的性质采用适宜方法使其分散均匀。

　　二、凡属挥发性或对光、热不稳定的原料药物，在制片过程中应釆取遮光、避热等适宜方法，以避免成分损失或失效。

　　三、压片前的物料、颗粒或半成品应控制水分，以适应制片工艺的需要，防止片剂在贮存期间发霉、变质。

　　四、片剂通常采用湿法制粒压片、干法制粒压片和粉末直接压片。干法制粒压片和粉末直接压片可避免引入水分，适合对湿热不稳定的药物的片剂制备。

　　五、根据依从性需要，片剂中可加入矫味剂、芳香剂和着色剂等，一般指含片、口腔贴片、咀嚼片、分散片、泡腾片、口崩片等。

　　六、为增加稳定性、掩盖原料药物不良臭味、改善片剂外观等，可对制成的药片包糖衣或薄膜衣。对一些遇胃液易破坏、刺激胃黏膜或需要在肠道内释放的口服药片，可包肠溶衣。必要时，薄膜包衣片剂应检查残留溶剂。

　　七、片剂外观应完整光洁，色泽均匀，有适宜的硬度和耐磨性，以免包装、运输过程中发生磨损或破碎，除另有规定外，非包衣片应符合片剂脆碎度检查法（通则0923）的要求。

　　八、片剂的微生物限度应符合要求。

　　九、根据原料药物和制剂的特性，除来源于动、植物多组分且难以建立测定方法的片剂外，溶出度、释放度、含量均匀度等应符合要求。

　　十、片剂应注意贮存环境中温度、湿度以及光照的影响，除另有规定外，片剂应密封贮存。生物制品原液、半成品和成品的生产及质量控制应符合相关品种要求。

　　除另有规定外，片剂应进行以下相应检查。

**【重量差异】**照下述方法检查，应符合规定。

　　检查法 取供试品20片，精密称定总重量，求得平均片重后，再分别精密称定每片的重量，每片重量与平均片重比较（凡无含量测定的片剂或有标示片重的中药片剂，每片重量应与标示片重比较），按表中的规定，超出重量差异限度的不得多于2片，并不得有1片超出限度1倍。

　　
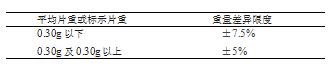


　　糖衣片的片芯应检查重量差异并符合规定，包糖衣后不再检查重量差异。薄膜衣片应在包薄膜衣后检查重量差异并符合规定。

　　凡规定检查含量均匀度的片剂，一般不再进行重量差异检查。

**【崩解时限】**除另有规定外，照崩解时限检查法（通则0921）检查，应符合规定。

　　阴道片照融变时限检查法（通则0922）检查，应符合规定。

　　咀嚼片不进行崩解时限检查。

　　凡规定检查溶出度、释放度的片剂，一般不再进行崩解时限检查。

**【发泡量】**阴道泡腾片照下述方法检查，应符合规定。

　　检查法 除另有规定外，取25ml具塞刻度试管（内径1.5cm，若片剂直径较大，可改为内径2.0cm）10支，按表中规定加水一定量，置37℃±1℃水浴中5分钟，各管中分别投入供试品1片，20分钟内观察最大发泡量的体积，平均发泡体积不得少于6ml，且少于4ml的不得超过2片。

　　
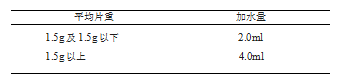


**【分散均匀性】**分散片照下述方法检查，应符合规定。

　　检查法 照崩解时限检查法（通则0921）检查，不锈钢丝网的筛孔内径为710μm，水温为15～25℃；取供试品6片，应在3分钟内全部崩解并通过筛网，如有少量不能通过筛网，但已软化成轻质上漂且无硬心者，符合要求。

**【微生物限度】**以动物、植物、矿物来源的非单体成分制成的片剂，生物制品片剂，以及黏膜或皮肤炎症或腔道等局部用片剂（如口腔贴片、外用可溶片、阴道片、阴道泡腾片等），照非无菌产品微生物限度检查：微生物计数法（通则1105）和控制菌检查法（通则1106）及非无菌药品微生物限度标准（通则1107）检查，应符合规定。规定检查杂菌的生物制品片剂，可不进行微生物限度检查。

# Part 2 General Rules for Preparation of Granules (0104)

**0104 颗粒剂**

　　颗粒剂系指原料药物与适宜的辅料混合制成具有一定粒度的干燥颗粒状制剂。

　　颗粒剂可分为可溶颗粒（通称为颗粒）、混悬颗粒、泡腾颗粒、肠溶颗粒，根据释放特性不同还有缓释颗粒等。

　　混悬颗粒 系指难溶性原料药物与适宜辅料混合制成的颗粒剂。临用前加水或其他适宜的液体振摇即可分散成混悬液。除另有规定外，混悬颗粒剂应进行溶出度（通则0931）检查。

　　泡腾颗粒 系指含有碳酸氢钠和有机酸，遇水可放出大量气体而呈泡腾状的颗粒剂。

　　泡腾颗粒中的原料药物应是易溶性的，加水产生气泡后应能溶解。有机酸一般用枸椽酸、酒石酸等。泡腾颗粒一般不得直接吞服。

　　肠溶颗粒 系指采用肠溶材料包裹颗粒或其他适宜方法制成的颗粒剂。肠溶颗粒耐胃酸而在肠液中释放活性成分或控制药物在肠道内定位释放，可防止药物在胃内分解失效，避免对胃的刺激。肠溶颗粒应进行释放度（通则0931）检查。肠溶颗粒不得咀嚼。

　　缓释颗粒 系指在规定的释放介质中缓慢地非恒速释放药物的颗粒剂。

　　缓释颗粒应符合缓释制剂（指导原则9013）的有关要求，并应进行释放度（通则0931）检查。缓释颗粒不得咀嚼。

　　颗粒剂在生产与贮藏期间应符合下列规定。

　　一、原料药物与辅料应均匀混合。含药量小或含毒、剧药物的颗粒剂，应根据原料药物的性质釆用适宜方法使其分散均匀。

　　二、除另有规定外，中药饮片应按各品种项下规定的方法进行提取、纯化、浓缩成规定的清膏，釆用适宜的方法干燥并制成细粉，加适量辅料或饮片细粉，混匀并制成颗粒；也可将清膏加适量辅料或饮片细粉，混匀并制成颗粒。

　　三、凡属挥发性原料药物或遇热不稳定的药物在制备过程应注意控制适宜的温度条件，凡遇光不稳定的原料药物应遮光操作。

　　四、颗粒剂通常釆用干法制粒、湿法制粒等方法制备。干法制粒可避免引入水分，尤其适合对湿热不稳定药物的颗粒剂的制备。

　　五、根据需要颗粒剂可加入适宜的辅料，如稀释剂、黏合剂、分散剂、着色剂以及矫味剂等。

　　六、除另有规定外，挥发油应均匀喷入干燥颗粒中，密闭至规定时间或用包合等技术处理后加入。

　　七、为了防潮、掩盖原料药物的不良气味，也可对颗粒进行包衣。必要时，包衣颗粒应检查残留溶剂。

　　八、颗粒剂应干燥，颗粒均匀，色泽一致，无吸潮、软化、结块、潮解等现象。

　　九、颗粒剂的微生物限度应符合要求。

　　十、根据原料药物和制剂的特性，除来源于动、植物多组分且难以建立测定方法的颗粒剂外，溶出度、释放度、含量均匀度等应符合要求。

　　十一、除另有规定外，颗粒剂应密封，置干燥处贮存，防止受潮。生物制品原液、半成品和成品的生产及质量控制应符合相关品种要求。

　　除另有规定外，颗粒剂应进行以下相应检查。

**【粒度】**除另有规定外，照粒度和粒度分布测定法（通则0982第二法双筛分法）测定，不能通过一号筛与能通过五号筛的总和不得超过15%。

**【水分】**中药颗粒剂照水分测定法（通则0832）测定，除另有规定外，水分不得超过8.0%。

**【干燥失重】**除另有规定外，化学药品和生物制品颗粒剂照干燥失重测定法（通则0831）测定，于105℃干燥（含糖颗粒应在80℃减压干燥）至恒重，减失重量不得超过2.0%。

**【溶化性】**除另有规定外，颗粒剂照下述方法检查，溶化性应符合规定。含中药原粉的颗粒剂不进行溶化性检查。

　　可溶颗粒检查法 取供试品10g（中药单剂量包装取1袋），加热水200ml，搅拌5分钟，立即观察，可溶颗粒应全部溶化或轻微浑浊。

　　泡腾颗粒检查法 取供试品3袋，将内容物分别转移至盛有200ml水的烧杯中，水温为15～25℃，应迅速产生气体而呈泡腾状，5分钟内颗粒均应完全分散或溶解在水中。

　　颗粒剂按上述方法检查，均不得有异物，中药颗粒还不得有焦屑。

　　混悬颗粒以及已规定检查溶出度或释放度的颗粒剂可不进行溶化性检查。

**【装量差异】**单剂量包装的颗粒剂按下述方法检查，应符合规定。

　　检查法　取供试品10袋（瓶），除去包装，分别精密称定每袋（瓶）内容物的重量，求出每袋（瓶）内容物的装量与平均装量。每袋（瓶）装量与平均装量相比较［凡无含量测定的颗粒剂或有标示装量的颗粒剂，每袋（瓶）装量应与标示装量比较］，超出装量差异限度的颗粒剂不得多于2袋（瓶），并不得有1袋（瓶）超出装量差异限度1倍。

　　
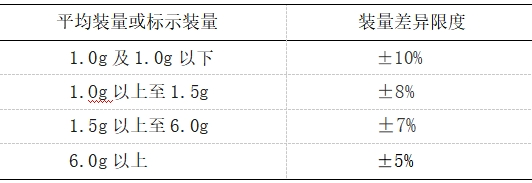


　　凡规定检查含量均匀度的颗粒剂，一般不再进行装量差异检查。

**【装量】**多剂量包装的颗粒剂，照最低装量检查法（通则0942）检查，应符合规定。

**【微生物限度】**以动物、植物、矿物质来源的非单体成分制成的颗粒剂，生物制品颗粒剂，照非无菌产品微生物限度检查：微生物计数法（通则1105）和控制菌检查法（通则1106）及非无菌药品微生物限度标准（通则1107）检查，应符合规定。规定检查杂菌的生物制品颗粒剂，可不进行微生物限度检查。

# Part 3 General Rules for Preparation of Pills (0108)

**0108 丸剂**

　　丸剂系指原料药物与适宜的辅料制成的球形或类球形固体制剂。

　　丸剂包括蜜丸、水蜜丸、水丸、糊丸、蜡丸、浓缩丸、滴丸和糖丸等。

　　蜜丸 系指饮片细粉以炼蜜为黏合剂制成的丸剂。其中每丸重量在0.5g（含0.5g）以上的称大蜜丸，每丸重量在0.5g以下的称小蜜丸。

　　水蜜丸 系指饮片细粉以炼蜜和水为黏合剂制成的丸剂。

　　水丸 系指饮片细粉以水（或根据制法用黄酒、醋、稀药汁、糖液、含5%以下炼蜜的水溶液等）为黏合剂制成的丸剂。

　　糊丸 系指饮片细粉以米粉、米糊或面糊等为黏合剂制成的丸剂。

　　蜡丸 系指饮片细粉以蜂蜡为黏合剂制成的丸剂。

　　浓缩丸 系指饮片或部分饮片提取浓缩后，与适宜的辅料或其余饮片细粉，以水、炼蜜或炼蜜和水等为黏合剂制成的丸剂。根据所用黏合剂的不同，分为浓缩水丸、浓缩蜜丸和浓缩水蜜丸等。

　　滴丸 系指原料药物与适宜的基质加热熔融混匀，滴入不相混溶、互不作用的冷凝介质中制成的球形或类球形制剂。

　　糖丸 系指以适宜大小的糖粒或基丸为核心，用糖粉和其他辅料的混合物作为撒粉材料，选用适宜的黏合剂或润湿剂制丸，并将原料药物以适宜的方法分次包裹在糖丸中而制成的制剂。

　　丸剂在生产与贮藏期间应符合下列有关规定。

　　一、除另有规定外，供制丸剂用的药粉应为细粉或最细粉。

　　二、炼蜜按炼蜜程度分为嫩蜜、中蜜和老蜜，制备时可根据品种、气候等具体情况选用。蜜丸应细腻滋润，软硬适中。

　　三、滴丸基质包括水溶性基质和非水溶性基质，常用的有聚乙二醇类（如聚乙二醇6000、聚乙二醇4000等）、泊洛沙姆、硬脂酸聚烃氧（40）酯、明胶、硬脂酸、单硬脂酸甘油酯、氢化植物油等。

　　四、丸剂通常采用泛制、塑制和滴制等方法制备。

　　五、浓缩丸所用饮片提取物应按制法规定，采用一定的方法提取浓缩制成。

　　六、蜡丸制备时，将蜂蜡加热熔化，待冷却至适宜温度后按比例加入药粉，混合均匀。

　　七、除另有规定外，水蜜丸、水丸、浓缩水蜜丸和浓缩水丸均应在80℃以下干燥；含挥发性成分或淀粉较多的丸剂（包括糊丸）应在60℃以下干燥；不宜加热干燥的应采用其他适宜的方法干燥。

　　八、滴丸冷凝介质必须安全无害，且与原料药物不发生作用。常用的冷凝介质有液状石蜡、植物油、甲基硅油和水等。

　　九、除另有规定外，糖丸在包装前应在适宜条件下干燥，并按丸重大小要求用适宜筛号的药筛过筛处理。

　　十、根据原料药物的性质、使用与贮藏的要求，凡需包衣和打光的丸剂，应使用各品种制法项下规定的包衣材料进行包衣和打光。

　　十一、除另有规定外，丸剂外观应圆整，大小、色泽应均匀，无粘连现象。蜡丸表面应光滑无裂纹，丸内不得有蜡点和颗粒。滴丸表面应无冷凝介质黏附。

　　十二、根据原料药物的性质与使用、贮藏的要求，供口服的滴丸可包糖衣或薄膜衣。必要时，薄膜衣包衣滴丸应检查残留溶剂。

　　十三、丸剂的微生物限度应符合要求。

　　十四、根据原料药物和制剂的特性，除来源于动、植物多组分且难以建立测定方法的丸剂外，溶出度、释放度、含量均匀度等应符合要求。

　　十五、除另有规定外，丸剂应密封贮存，防止受潮、发霉、虫蛀、变质。

　　除另有规定外，丸剂应进行以下相应检查。

**【水分】**照水分测定法（通则0832）测定。除另有规定外，蜜丸和浓缩蜜丸中所含水分不得过15.0%；水蜜丸和浓缩水蜜丸不得过12.0%；水丸、糊丸、浓缩水丸不得过9.0%。

　　蜡丸不检查水分。

**【重量差异】**（1）除另有规定外，滴丸照下述方法检查，应符合规定。

　　检查法 取供试品20丸，精密称定总重量，求得平均丸重后，再分别精密称定每丸的重量。每丸重量与标示丸重相比较（无标示丸重的，与平均丸重比较），按下表中的规定，超出重量差异限度的不得多于2丸，并不得有1丸超岀限度1倍。

　　
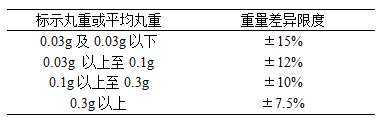


　　（2）除另有规定外，糖丸照下述方法检查，应符合规定。

　　检查法 取供试品20丸，精密称定总重量，求得平均丸重后，再分别精密称定每丸的重量。每丸重量与标示丸重相比较（无标示丸重的，与平均丸重比较），按下表中的规定，超出重量差异限度的不得多于2丸，并不得有1丸超出限度1倍。

　　
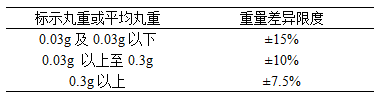


　　（3）除另有规定外，其他丸剂照下述方法检查，应符合规定。

　　检查法 以10丸为1份（丸重1.5g及1.5g以上的以1丸为1份），取供试品10份，分别称定重量，再与每份标示重量（每丸标示量×称取丸数）相比较（无标示重量的丸剂，与平均重量比较），按下表规定，超出重量差异限度的不得多于2份，并不得有1份超出限度1倍。

　　
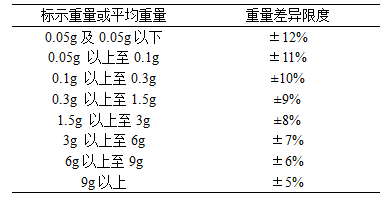


　　包糖衣丸剂应检查丸芯的重量差异并符合规定，包糖衣后不再检查重量差异，其他包衣丸剂应在包衣后检查重量差异并符合规定；凡进行装量差异检查的单剂量包装丸剂及进行含量均匀度检查的丸剂，一般不再进行重量差异检查。

**【装量差异】**除糖丸外，单剂量包装的丸剂，照下述方法检查应符合规定。

　　检查法 取供试品10袋（瓶），分别称定每袋（瓶）内容物的重量，每袋（瓶）装量与标示装量相比较，按下表规定，超出装量差异限度的不得多于2袋（瓶），并不得有1袋（瓶）超出限度1倍。

　　
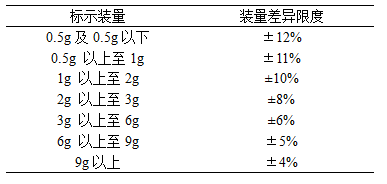


**【装量】**装量以重量标示的多剂量包装丸剂，照最低装量检查法（通则0942）检查，应符合规定。

　　以丸数标示的多剂量包装丸剂，不检查装量。

**【溶散时限】**除另有规定外，取供试品6丸，选择适当孔径筛网的吊篮（丸剂直径在2.5mm以下的用孔径约0.42mm的筛网；在2.5～3.5mm之间的用孔径约1.0mm的筛网；在3.5mm以上的用孔径约2.0mm的筛网），照崩解时限检查法（通则0921）片剂项下的方法加挡板进行检查。除另有规定外，小蜜丸、水蜜丸和水丸应在1小时内全部溶散；浓缩水丸、浓缩蜜丸、浓缩水蜜丸和糊丸应在2小时内全部溶散。滴丸不加挡板检查，应在30分钟内全部溶散，包衣滴丸应在1小时内全部溶散。操作过程中如供试品黏附挡板妨碍检查时，应另取供试品6丸，以不加挡板进行检查。上述检查，应在规定时间内全部通过筛网。如有细小颗粒状物未通过筛网，但已软化且无硬心者可按符合规定论。

　　蜡丸照崩解时限检查法（通则0921）片剂项下的肠溶衣片检查法检查，应符合规定。

　　除另有规定外，大蜜丸及研碎、嚼碎后或用开水、黄酒等分散后服用的丸剂不检查溶散时限。

**【微生物限度】**以动物、植物、矿物质来源的非单体成分制成的丸剂，生物制品丸剂，照非无菌产品微生物限度检查：微生物计数法（通则1105）和控制菌检查法（通则1106）及非无菌药品微生物限度标准（通则1107）检查，应符合规定。生物制品规定检查杂菌的，可不进行微生物限度检查。

# Part 4 General Rules for Preparation of Mixtures (0181)

**0181 合剂**

　　合剂系指饮片用水或其他溶剂，采用适宜的方法提取制成的口服液体制剂（单剂量灌装者也可称“口服液”）。

　　合剂在生产与贮藏期间应符合下列规定。

　　一、饮片应按各品种项下规定的方法提取、纯化、浓缩制成口服液体制剂。

　　二、根据需要可加入适宜的附加剂。除另有规定外，在制剂确定处方时，如需加入抑菌剂，该处方的抑菌效力应符合抑菌效力检查法（通则1121）的规定。山梨酸和苯甲酸的用量不得超过0.3%（其钾盐、钠盐的用量分别按酸计），羟苯酯类的用量不得超过0.05%，如加入其他附加剂，其品种与用量应符合国家标准的有关规定，不影响成品的稳定性，并应避免对检验产生干扰。必要时可加入适量的乙醇。

　　三、合剂若加蔗糖，除另有规定外，含蔗糖量一般不高于20%（g/ml）。

　　四、除另有规定外，合剂应澄清。在贮存期间不得有发霉、酸败、异物、变色、产生气体或其他变质现象，允许有少量摇之易散的沉淀。

　　五、一般应检查相对密度、pH值等。

　　六、除另有规定外，合剂应密封，置阴凉处贮存。

　　除另有规定外，合剂应进行以下相应检查。

**【装量】**单剂量灌装的合剂，照下述方法检查，应符合规定。

　　检查法 取供试品5支，将内容物分别倒入经标化的量入式量筒内，在室温下检视，每支装量与标示装量相比较，少于标示装量的不得多于1支，并不得少于标示装量的95%。

　　多剂量灌装的合剂，照最低装量检查法（通则0942）检查，应符合规定。

**【微生物限度】**除另有规定外，照非无菌产品微生物限度检查：微生物计数法（通则1105）和控制菌检查法（通则1106）及非无菌药品微生物限度标准（通则1107）检查，应符合规定。

# Part 5 General Rules for Preparation of Soft extract (0183)

**0183 煎膏剂（膏滋）**

　　煎膏剂系指饮片用水煎煮，取煎煮液浓缩，加炼蜜或糖（或转化糖）制成的半流体制剂。

　　煎膏剂在生产与贮藏期间应符合下列有关规定。

　　一、饮片按各品种项下规定的方法煎煮，滤过，滤液浓缩至规定的相对密度，即得清膏。

　　二、如需加入饮片原粉，除另有规定外，一般应加入细粉。

　　三、清膏按规定量加入炼蜜或糖（或转化糖）收膏；若需加饮片细粉，待冷却后加入，搅拌混匀。除另有规定外，加炼蜜或糖（或转化糖）的量，一般不超过清膏量的3倍。

　　四、煎膏剂应无焦臭、异味，无糖的结晶析出。

　　五、除另有规定外，煎膏剂应密封，置阴凉处贮存。

　　除另有规定外，煎膏剂应进行以下相应检查。

**【相对密度】**除另有规定外，取供试品适量，精密称定，加水约2倍，精密称定，混匀，作为供试品溶液。照相对密度测定法（通则0601）测定，按下式计算，应符合各品种项下的有关规定。

　　
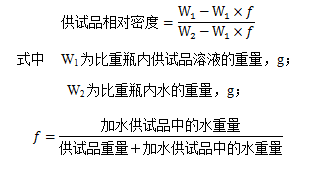


　　凡加饮片细粉的煎膏剂，不检查相对密度。

**【不溶物】**取供试品5g，加热水200ml，搅拌使溶化，放置3分钟后观察，不得有焦屑等异物。

　　加饮片细粉的煎膏剂，应在未加入细粉前检查，符合规定后方可加入细粉。加入药粉后不再检查不溶物。

**【装量】**照最低装量检查法（通则0942）检查，应符合规定。

**【微生物限度】**照非无菌产品微生物限度检查：微生物计数法（通则1105）和控制菌检查法（通则1106）及非无菌药品微生物限度标准（通则1107）检查，应符合规定。

# Part 5 General Rules for Thin layer chromatography (0502)

**0502 薄层色谱法**

　　薄层色谱法系将供试品溶液点于薄层板上，在展开容器内用展开剂展开，使供试品所含成分分离，所得色谱图与适宜的标准物质按同法所得的色谱图对比，亦可用薄层色谱扫描仪进行扫描，用于鉴别、检查或含量测定。

　　1.仪器与材料

　　（1）薄层板 按支持物的材质分为玻璃板、塑料板或铝板等；按固定相种类分为硅胶薄层板、键合硅胶板、微晶纤维素薄层板、聚酰胺薄层板、氧化铝薄层板等。固定相中可加入黏合剂、荧光剂。硅胶薄层板常用的有硅胶 G、硅胶 GF_254_、硅胶 H、硅胶 HF_254_，G、H 表示含或不含石膏黏合剂，F_254_ 为在紫外光 254nm 波长下显绿色背景的荧光剂。按固定相粒径大小分为普通薄层板（10~40µm）和高效薄层板（5~10µm）。

　　在保证色谱质量的前提下，可对薄层板进行特别处理和化学改性以适应分离的要求，可用实验室自制的薄层板。固定相颗粒大小一般要求粒径为 10~40μm。玻板应光滑、平整，洗净后不附水珠。

　　（2）点样器 一般釆用微升毛细管或手动、半自动、全自动点样器材。

　　（3）展开容器 上行展开一般可用适合薄层板大小的专用平底或双槽展开缸，展开时须能密闭。水平展开用专用的水平展开槽。

　　（4）显色装置 喷雾显色应使用玻璃喷雾瓶或专用喷雾器，要求用压缩气体使显色剂呈均匀细雾状喷出；浸渍显色可用专用玻璃器械或用适宜的展开缸代用；蒸气熏蒸显色可用双槽展开缸或适宜大小的干燥器代替。

　　（5）检视装置 为装有可见光、254nm 及 365nm 紫外光光源及相应的滤光片的暗箱，可附加摄像设备供拍摄图像用。暗箱内光源应有足够的光照度。

　　（6）薄层色谱扫描仪 系指用一定波长的光对薄层板上有吸收的斑点，或经激发后能发射出荧光的斑点，进行扫描，将扫描得到的谱图和积分数据用于物质定性或定量的分析仪器。

　　2.操作方法

　　（1）薄层板制备

　　市售薄层板 临用前一般应在 110℃ 活化 30 分钟。聚酰胺薄膜不需活化。铝基片薄层板、塑料薄层板可根据需要剪裁，但须注意剪裁后的薄层板底边的固定相层不得有破损。如在存放期间被空气中杂质污染，使用前可用三氯甲烷、甲醇或二者的混合溶剂在展开缸中上行展开预洗，晾干，110℃ 活化，置干燥器中备用。

　　自制薄层板 除另有规定外，将 1 份固定相和 3 份水（或加有黏合剂的水溶液，如 0.2%~0.5%羟甲基纤维素钠水溶液，或为规定浓度的改性剂溶液）在研钵中按同一方向研磨混合，去除表面的气泡后，倒入涂布器中，在玻板上平稳地移动涂布器进行涂布（厚度为 0.2~0.3mm），取下涂好薄层的玻板，置水平台上于室温下晾干后，在 110℃ 烘 30 分钟，随即置于有干燥剂的干燥箱中备用。使用前检查其均匀度，在反射光及透视光下检视，表面应均匀、平整、光滑，并且无麻点、无气泡、无破损及污染。

　　（2）点样 除另有规定外，在洁净干燥的环境中，用专用毛细管或配合相应的半自动、自动点样器械点样于薄层板上。一般为圆点状或窄细的条带状，点样基线距底边 10~15mm，高效板一般基线离底边 8~10mm。圆点状直径一般不大于 4mm，高效板一般不大于 2mm。接触点样时注意勿损伤薄层表面。条带状宽度一般为 5~10mm，高效板条带宽度一般为 4~8mm，可用专用半自动或自动点样器械喷雾法点样。点间距离可视斑点扩散情况以相邻斑点互不干扰为宜，一般不少于 8mm，高效板供试品间隔不少于 5mm。

　　（3）展开 将点好供试品的薄层板放入展开缸中，浸入展开剂的深度为距原点 5mm 为宜，密闭。除另有规定外，一般上行展开 8~15cm，高效薄层板上行展开 5~8cm。溶剂前沿达到规定的展距，取出薄层板，晾干，待检测。

　　展开前如需要溶剂蒸气预平衡，可在展开缸中加入适量的展开剂，密闭，一般保持 15~30 分钟。溶剂蒸气预平衡后，应迅速放入载有供试品的薄层板，立即密闭，展开。如需使展开缸达到溶剂蒸气饱和的状态，则须在展开缸的内壁贴与展开缸高、宽同样大小的滤纸，一端浸入展开剂中，密闭一定时间，使溶剂蒸气达到饱和再如法展开。

　　必要时，可进行二次展开或双向展开，进行第二次展开前，应使薄层板残留的展开剂完全挥干。

　　（4）显色与检视 有颜色的物质可在可见光下直接检视，无色物质可用喷雾法或浸渍法以适宜的显色剂显色，或加热显色，在可见光下检视。有荧光的物质或显色后可激发产生荧光的物质可在紫外光灯（365nm 或 254nm）下观察荧光斑点。对于在紫外光下有吸收的成分，可用带有荧光剂的薄层板（如硅胶 GF_254_ 板），在紫外光灯（254nm）下观察荧光板面上的荧光物质淬灭形成的斑点。

　　（5）记录 薄层色谱图像一般可采用摄像设备拍摄，以光学照片或电子图像的形式保存。也可用薄层色谱扫描仪扫描或其他适宜的方式记录相应的色谱图。

　　3.系统适用性试验

　　按各品种项下要求对实验条件进行系统适用性试验，即用供试品和标准物质对实验条件进行试验和调整，应符合规定的要求。

　　（1）比移值（R_f_） 系指从基线至展开斑点中心的距离与从基线至展开剂前沿的距离的比值。

　　
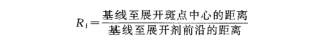


　　除另有规定外，杂质检查时，各杂质斑点的比移值 R_f_ 以在 0.2~0.8 之间为宜。

　　（2）检出限 系指限量检查或杂质检查时，供试品溶液中被测物质能被检出的最低浓度或量。一般采用已知浓度的供试品溶液或对照标准溶液，与稀释若干倍的自身对照标准溶液在规定的色谱条件下，在同一薄层板上点样、展开、检视，后者显清晰可辨斑点的浓度或量作为检出限。

　　（3）分离度（或称分离效能） 鉴别时，供试品与标准物质色谱中的斑点均应清晰分离。当薄层色谱扫描法用于限量检查和含量测定时，要求定量峰与相邻峰之间有较好的分离度，分离度(R)的计算公式为：

　　R=2(d_2_-d_1_)/(W_1_+W_2_)

　　式中 d_2_ 为相邻两峰中后一峰与原点的距离；

　　d_1_ 为相邻两峰中前一峰与原点的距离；

　　W_1_ 及 W_2_ 为相邻两峰各自的峰宽。

　　除另有规定外，分离度应大于 1.0。

　　在化学药品杂质检查的方法选择时，可将杂质对照品用供试品自身稀释的对照溶液溶解制成混合对照溶液，也可将杂质对照品用待测组分的对照品溶液溶解制成混合对照标准溶液，还可釆用供试品以适当的降解方法获得的溶液，上述溶液点样展开后的色谱图中，应显示清晰分离的斑点。

　　（4）相对标准偏差 薄层扫描含量测定时，同一供试品溶液在同一薄层板上平行点样的待测成分的峰面积测量值的相对标准偏差应不大于 5.0%；需显色后测定的或者异板的相对标准偏差应不大于 10.0%。

　　4.测定法

　　（1）鉴别 按各品种项下规定的方法，制备供试品溶液和对照标准溶液，在同一薄层板上点样、展开与检视，供试品色谱图中所显斑点的位置和颜色（或荧光）应与标准物质色谱图的斑点一致。必要时化学药品可采用供试品溶液与标准溶液混合点样、展开，与标准物质相应斑点应为单一、紧密斑点。

　　（2）限量检查与杂质检查 按各品种项下规定的方法，制备供试品溶液和对照标准溶液，并按规定的色谱条件点样、展开和检视。供试品溶液色谱图中待检查的斑点与相应的标准物质斑点比较，颜色（或荧光）不得更深；或照薄层色谱扫描法操作，测定峰面积值，供试品色谱图中相应斑点的峰面积值不得大于标准物质的峰面积值。含量限度检查应按规定测定限量。

　　化学药品杂质检查可釆用杂质对照法、供试品溶液的自身稀释对照法或两法并用。供试品溶液除主斑点外的其他斑点与相应的杂质对照标准溶液或系列浓度杂质对照标准溶液的相应主斑点比较，不得更深，或与供试品溶液自身稀释对照溶液或系列浓度自身稀释对照溶液的相应主斑点比较，不得更深。通常应规定杂质的斑点数和单一杂质量，当釆用系列自身稀释对照溶液时，也可规定估计的杂质总量。

　　（3）含量测定 照薄层色谱扫描法，按各品种项下规定的方法，制备供试品溶液和对照标准溶液，并按规定的色谱条件点样、展开、扫描测定。或将待测色谱斑点刮下经洗脱后，再用适宜的方法测定。

　　5.薄层色谱扫描法

　　系指用一定波长的光照射在薄层板上，对薄层色谱中可吸收紫外光或可见光的斑点，或经激发后能发射出荧光的斑点进行扫描，将扫描得到的图谱及积分数据用于鉴别、检查或含量测定。可根据不同薄层色谱扫描仪的结构特点，按照规定方式扫描测定，一般选择反射方式，采用吸收法或荧光法。除另有规定外，含量测定应使用市售薄层板。

　　扫描方法可采用单波长扫描或双波长扫描。如采用双波长扫描，应选用待测斑点无吸收或最小吸收的波长为参比波长，供试品色谱图中待测斑点的比移值（R_f_ 值）、光谱扫描得到的吸收光谱图或测得的光谱最大吸收和最小吸收应与对照标准溶液相符，以保证测定结果的准确性。薄层色谱扫描定量测定应保证供试品斑点的量在线性范围内，必要时可适当调整供试品溶液的点样量，供试品与标准物质同板点样、展开、扫描、测定和计算。

　　薄层色谱扫描用于含量测定时，通常采用线性回归二点法计算，如线性范围很窄时，可用多点法校正多项式回归计算。供试品溶液和对照标准溶液应交叉点于同一薄层板上，供试品点样不得少于 2 个，标准物质每一浓度不得少于 2 个。扫描时，应沿展开方向扫描，不可横向扫描。
